# Supplementary material for: Stochastic parametric skeletal dosimetry model for humans: Anatomical-morphological basis and parameter evaluation
Source: PLoS One. 2025 Jul 2;20(7):e0327156. doi: 10.1371/journal.pone.0327156 (PMC12306906; doi:10.1371/journal.pone.0327156)
Supplement: S1 Femur — (DOCX) [file pone.0327156.s001.docx]

**Femur**

**Pre-adults, analysis of published data on femur macro-parameters and cortical thickness**

The shape and size of the femur are significantly dependent on age, Fig. F1 illustrates the age-changes in the period 0–5 years.


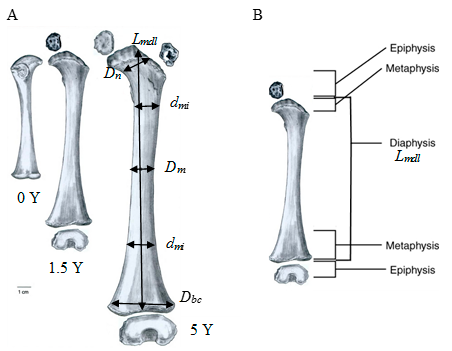


**Fig. F1.** Age-changes in femur bone: A-Femur in natural proportion (based on Baker et al. 2005) for newborn, infant and children of about 5 years; epiphyseal centers of ossification are shown; B- diagram of the divisions of pre-adult femur into diaphysis, metaphysis, and epiphysis; note that the diaphysis length (syn. maximal diaphysis length) includes the length of metaphysis (Haas J et al. 1994, Meresh 1970). Letter designations are deciphered in the text.

Main measured parameters described in literature which were collected:

- Maximal diaphysis length *(L_mdl_)-* distance between the proximal and distal growth zone (epiphyseal lines), does not include the epiphysis ossified from separate centers (Fig. 1);
- Outside diameter (*D_m_*) in the mid-point of diaphysis (Fig. 1);
- Cortical thickness (Ct.Th) in the mid-point of diaphysis;
- Bicondylar diameter (*D_bc_*) in distal part of femur (Fig. 1);
- Femur neck width (*D_n_*) (Fig. 1);
- Relative outside diameters (relative to mid-point) at different distances from the distal end of the diaphysis; they were estimated by us with use the images from Gosman et al. (2013), and used for derivation of absolute values of *d_mi,_* i.e. outside diameters in different point *i* of diaphysis (Fig. 2);
- Relative cortical thickness (relative to mid-point) at different distances from the distal end of the diaphysis; they were estimated by us with use of images from Gosman et al. (2013) (Fig. 2) and used for derivation of absolute values of Ct.Th.

Tables F1–F4 present the measured data on femur macro-parameters and cortical thickness of pre-adults; Table 5 shows the averaged values assumed for modeling.

**F1.** Published data on maximal femur diaphyseal length (*L_mdl_*), mm.

| Author | Age | n | M | SD |
| --- | --- | --- | --- | --- |
| Florence et al. 2007 | 0 | 34 | 69.8 | 8.2 |
| Medvedev et al 1999* | 0 | n/a | 75.0 | 4.5 |
| Jeanty et al 1983* | 0 | >100 | 74.0 | 3.9 |
| Demidov et al 1990* | 0 | n/a | 76.0 | 5.5 |
| Blinov et al. 2002* | 0 | >100 | 75.8 | 4.7 |
| Maresh et al. 1970 | 0.1 | 127 | 86.6 | 4.9 |
| Miles 1994 | 0.1 | 36 | 74.7 | - |
| Florence et al. 2007 | 0.25 | 38 | 80,3 | 5.1 |
| Maresh et al. 1970 | 0.25 | 74 | 100.7 | 4.2 |
| Florence et al. 2007 | 0.4 | 9 | 89.2 | 7.2 |
| Maresh et al. 1970 | 0.5 | 145 | 111.7 | 4.8 |
| Florence et al. 2007 | 0.8 | 5 | 120,4 | 7.9 |
| Miles 1994 | 0.8 | 3 | 90.0 | 9.0 |
| Maresh et al. 1970 | 1 | 153 | 135.6 | 5.4 |
| Maresh et al. 1970 | 1.5 | 152 | 154.7 | 6.6 |
| Maresh et al. 1970 | 1.5 | 1 | 136 | - |
| Florence et al. 2007 | 2 | 4 | 157.5 | 30.3 |
| Maresh et al. 1970 | 2 | 72 | 171.6 | 7.2 |
| Maresh et al. 1970 | 2.5 | 154 | 186 | 7.8 |
| Maresh et al. 1970 | 3 | 150 | 199.4 | 8.6 |
| Maresh et al. 1970 | 3.5 | 151 | 211.6 | 10.7 |
| Maresh et al. 1970 | 4 | 152 | 223.7 | 10 |
| Florence et al. 2007 | 4.5 | 3 | 212,2 | 8.2 |
| Maresh et al. 1970 | 4.5 | 74 | 235.6 | 10.9 |
| Maresh et al. 1970 | 5 | 157 | 247.3 | 11.3 |
| Miles 1994 | 5 | 4 | 192.3 | 6.8 |
| Maresh et al. 1970 | 5.5 | 147 | 257.6 | 12.0 |
| Maresh et al. 1970 | 6 | 146 | 269.3 | 12.8 |
| Maresh et al. 1970 | 6.5 | 153 | 279.7 | 13.2 |
| Maresh et al. 1970 | 7 | 157 | 290.0 | 13.5 |
| Maresh et al. 1970 | 7.5 | 159 | 300.5 | 14.4 |
| Maresh et al. 1970 | 8 | 155 | 311.0 | 15.1 |
| Maresh et al. 1970 | 8.5 | 154 | 320.0 | 15.2 |
| Maresh et al. 1970 | 9 | 159 | 329.6 | 15.7 |
| Maresh et al. 1970 | 9.5 | 161 | 339.4 | 17.2 |
| Florence et al. 2007 | 10 | 2 | 315.0 | 10.0 |
| Maresh et al. 1970 | 10 | 161 | 348.6 | 17.4 |
| Miles 1994 | 10 | 7 | 286.4 | 21.2 |
| Maresh et al. 1970 | 10.5 | 78 | 357.0 | 18.8 |
| Maresh et al. 1970 | 11 | 151 | 367 | 19.5 |
| Maresh et al. 1970 | 11.5 | 151 | 376.9 | 20.8 |
| Maresh et al. 1970 | 12 | 145 | 386.9 | 21.0 |

*Data was taken from Medvedev et al. 2009;

For children older than 12 years, measurements of maximal diaphyseal length were not found but was found data of maximal bone length (including epiphysis). For calculation of maximal diaphyseal length, maximal bone length was multiplied by conversion factor *k_l_*=0.91. This value was calculated as average relation of *L_mdl_* to maximal bone length, measured for the bone samples of 10-12y old children (Maresh et al. 1970). Diaphyseal lengths for children older than 12 are shown in table 13.

**Table F2.** Published data on maximal femur diaphyseal length (*L_mdl_*), mm according to Maresh et al. 1970.

| Sex | Age | n | M | SD |
| --- | --- | --- | --- | --- |
| Male | 13 | 73 | 406.0 | 19.5 |
|  | 13.5 | 73 | 416.0 | 21.8 |
|  | 14 | 75 | 427.2 | 21.9 |
|  | 14.5 | 69 | 434.6 | 22.9 |
|  | 15 | 61 | 443.8 | 21.3 |
|  | 15.5 | 52 | 452.4 | 21.2 |
|  | 18 | 28 | 464.4 | 22.1 |
| Female | 13 | 69 | 405.8 | 21.9 |
|  | 13.5 | 63 | 411.2 | 20.0 |
|  | 14 | 64 | 417.3 | 20.4 |
|  | 14.5 | 41 | 421.5 | 18.9 |
|  | 15 | 57 | 421.4 | 19.4 |
|  | 15.5 | 12 | 427.9 | 23.6 |

**Table F3.** Published data on femur outside diameter (*D_m_*) and cortical thickness (Ct.Th) in the mid-point of diaphysis, mm

| Author | Age | n | *D_m_* | | Ct.Th | |
| --- | --- | --- | --- | --- | --- | --- |
|  |  |  | M | SD | M | SD |
| Svadkovsky 1961 | 0 | 10 | 6.8 | 0.6 | 2.3 | - |
| Florence et al. 2007 | 0 | 10 | 6.2 | 0.8 | 2.0 | 0.3 |
| Dhavale 2016 | 0 | 5 | 7.5 | 1 | 2.5 | 0.3 |
| Florence et al. 2007 | 0.2 | 17 | 6.8 | 0,6 | 2.2 | 0.3 |
| Florence et al. 2007 | 0.4 | 9 | 7.4 | 0,9 | 2.0 | 0.9 |
| Florence et al. 2007 | 0.75 | 5 | 9.2 | 1.2 | 2.4 | 0.6 |
| Dhavale 2016 | 1 | 10 | 11 | 0,6 | 3.2 | 0.4 |
| Svadkovsky 1961 | 2 | 20 | 12.4 | 5.2 | 2.3 | - |
| Florence et al. 2007 | 2 |  | 11.1 | 2.4 | 3 | 1.1 |
| Svadkovsky 1961 | 4 | 5 | 14.2 | 0.0 | 2.7 | - |
| Florence et al. 2007 | 4.5 | 3 | 14.1 | 0.8 | 3.6 | 0.3 |
| Dhavale 2016 | 5 | 4 | 14.8 | 0,9 | 4.2 | 0.3 |
| Svadkovsky 1961 | 6 | 5 | 16.2 | 2.2 | 3.4 | - |
| Svadkovsky 1961 | 8 | 5 | 18.6 | 0.0 | 4.1 | - |
| Svadkovsky 1961 | 10 | 5 | 19.5 | 1.7 | 4.5 | - |
| Petit et al. 2002 | 10 | 90 | - | - | 4.4 | 0.9 |
| Svadkovsky 1961 | 12 | 6 | 20.6 | 2.0 | 4.6 | - |
| Svadkovsky 1961 | 14 | 6 | 23.3 | 1.7 | 5.4 | - |
| Svadkovsky 1961 | 16 | 6 | 24.6 | 1.8 | 7.1 | - |
| Svadkovsky 1961 | 18 | 6 | 27.5 | 2.1 | 7.3 | - |

**Table F4.** Published data on femur neck width (*D_n_*), mm (male and female).

| Author | Age | n | M | SD |
| --- | --- | --- | --- | --- |
| Djuric et al. 2012 | 7 (0–14) | 29 | 23 | 7 |
| Petit et al. 2002 | 10 | 90 | 24.9 | 1.7 |
| Kanellopoulos et al. 2007 | 15 | 11 | 32.0 | 4.3 |
| Takale and Bagal 2016 | adults | 50 | 25.9 | - |
| Chowdhury et al. 2012 | adults | 89 | 46.7 | 1.7 |
| Clavero et al. 2015 | adults | 56 | 28.4 | 2.9 |
| Asha et al. 2014 | adults | 70 | 25.9 | 2.9 |

**Table F5.** Published data on femur bicondylar diameter (*D_bc_*), mm.

| Author | Age | n | M | SD |
| --- | --- | --- | --- | --- |
| Nemec et al. 2013 | 0 | 13 | 26.4 | 2.5 |
| Zivicnjak et al. 2008 | 2 | 70 | 62.55 | 3.35 |
| Zivicnjak et al. 2008 | 3 | 115 | 65.9 | 3.5 |
| Zivicnjak et al. 2008 | 4 | 153 | 68 | 3.15 |
| Singh et al. 2007 | 5 | 14 | 65.7 | 4.6 |
| Zivicnjak et al. 2008 | 5 | 218 | 70.8 | 3.9 |
| Singh et al. 2007 | 6 | 17 | 66.5 | 3.9 |
| Zivicnjak et al. 2008 | 6 | 227 | 72.9 | 4.15 |
| Singh et al. 2007 | 7 | 37 | 66.7 | 6.8 |
| Zivicnjak et al. 2008 | 7 | 368 | 75.95 | 4.35 |
| Singh et al. 2007 | 8 | 37 | 69.8 | 5.2 |
| Zivicnjak et al. 2008 | 8 | 295 | 77.3 | 4.05 |
| Singh et al. 2007 | 9 | 40 | 73.2 | 4 |
| Zivicnjak et al. 2008 | 9 | 358 | 80.3 | 4.4 |
| Singh et al. 2007 | 10 | 40 | 74.5 | 6.1 |
| Zivicnjak et al. 2008 | 10 | 343 | 82.3 | 4.4 |
| Singh et al. 2007 | 11 | 36 | 77.5 | 5.5 |
| Zivicnjak et al. 2008 | 11 | 433 | 85.35 | 4.6 |
| Singh et al. 2007 | 12 | 36 | 77.7 | 8.5 |
| Zivicnjak et al. 2008 | 12 | 394 | 87.05 | 5 |
| Singh et al. 2007 | 13 | 46 | 80.3 | 7.2 |
| Zivicnjak et al. 2008 | 13 | 406 | 88.25 | 4.6 |
| Singh et al. 2007 | 14 | 70 | 82.5 | 8.9 |
| Zivicnjak et al. 2008 | 14 | 434 | 89.65 | 4.3 |
| Singh et al. 2007 | 15 | 37 | 85.1 | 6.8 |
| Zivicnjak et al. 2008 | 15 | 457 | 90.45 | 4.2 |

**Table F6.** Averaged values of main femur-parameters assumed for reference ages, mm.

| Age | *L_mdl_* | | Ct.Th in mid-point of diaphysis | | *D_m_* | | *D_n_* | | *D_bc_* | |
| --- | --- | --- | --- | --- | --- | --- | --- | --- | --- | --- |
|  | M | SD | M | SD | M | SD | M | SD | M | SD |
| 0 | 79 | 4.1 | 2.2 | 0.5 | 6.8 | 0,8 | - | - | 26.4 | 2.5 |
| 1 | 145 | 5 | 2.9 | 0.5 | 10.6 | 0.8 | - | - | 34 | 4 |
| 5 | 250 | 10.2 | 3.9 | 0.3 | 14.6 | 0.9 | 23 | 7 | 68.2 | 4.2 |
| 10 | 352 | 19.1 | 4.4 | 0.9 | 19.5 | 1.7 | 24.9 | 1.7 | 78.4 | 5.3 |
| 15 m | 450 | 21.8 | 6.3 | 1.2 ^a^ | 24 | 1.8 | 32.0 | 4.3 | 87.8 | 5.5 |
| 15 f | 424 | 20.6 | 6.3 | 1.2 ^a^ | 24 | 1.8 | 32.0 | 4.3 | 87.8 | 5.5 |
| 18 m | 464 | 22.1 | - | - | - | - | - | - | - | - |

As seen, we have data on Ct.Th and outside- diameter only for mid-point of diaphysis. An exception is the data on the femur neck in children of 10 years measured by Petit et al. (2002, n=90; Ct.Th=1.77±0.26 mm). For modeling, the estimates in other diaphysis points for reference ages are necessary. For this purpose, we have used the data of Gosman et al. (2013) who evaluated the diameters of the diaphysis and the Ct.Th in different points of growing femur. Fig. F2 presents the scheme of femur cross-sections in reference points located from distal to proximal end.


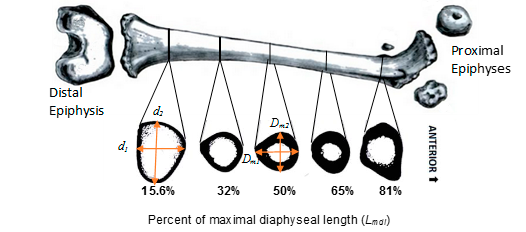


**Fig. F2.** Schematic view on femur-diaphysis cross-sections from distal to proximal end illustrating the changes in femur diameter and cortical thickness (Ct.Th). Numbers indicate the position of cross-sections relative to maximal diaphyseal length (diaphyseal/metaphyseal length). According to Gosman et al. (2013) with modifications.

Since the bone increases in length with age, the position of the points was determined relative to the total length of the diaphysis. Based on Gosman et al. (2013) data, we calculated the relative values (relative to the central point of 50%) of *d_mi_* and *Ct.Th_i_* for persons of studied ages (Table F7, F8) and absolute value of the parameters (Table F9, F10).

**Table F7.** Relative values (relative mid-point 50%) of femur outside-diameters (*d_mi_*) in reference points from Fig. F2 (relative units, based on Gosman et al. 2013).

| Age | 15.6% | | 32% | | **Mid-point 50%** | 65% | | 81% | |
| --- | --- | --- | --- | --- | --- | --- | --- | --- | --- |
|  | *d_1_* | *d_2_* | *d_1_* | *d_2_* | ***D_m_*** | *d_1_* | *d_2_* | *d_1_* | *d_2_* |
| 0–1.9 | 1.1 | 1.7 | 1.0 | 1.2 | **1.0** | 1.1 | 1.0 | 1.3 | 1.2 |
| 2–4.9 | 1.2 | 1.8 | 1.0 | 1.3 | **1.0** | 1.1 | 1.0 | 1.6 | 1.3 |
| 5–8.9 | 1.0 | 1.8 | 1.0 | 1.2 | **1.0** | 1.0 | 1.0 | 1.3 | 1.3 |
| 9–13.7 | 1.0 | 1.5 | 1.0 | 1.1 | **1.0** | 1.0 | 1.1 | 1.1 | 1.3 |
| 14–17.9 | 1.0 | 1.4 | 1.1 | 1.2 | **1.0** | 0.9 | 1.0 | 1.0 | 1.0 |

*d_1_* – anterior-posterior direction; *d_2_* – lateral-medial direction; in mid-point *Dm_1_= Dm_2_*

**Table F8.** Relative values (relative mid-point 50%) of femur- diaphysis *Ct.Th_i_* in reference points from Fig. F2 (relative units, based on Gosman et al. 2013)

| Age | 15.6% | 32% | **Mid-point 50%** | 65% | 81% |
| --- | --- | --- | --- | --- | --- |
| 0–1.9 | 0.4 | 0.6 | **1.0** | 0.7 | 0.5 |
| 2–4.9 | 0.6 | 1.0 | **1.0** | 1.4 | 0.8 |
| 5–8.9 | 0.5 | 0.6 | **1.0** | 1.1 | 0.5 |
| 9–13.7 | 0.5 | 0.9 | **1.0** | 1.0 | 1.0 |
| 14–17.9 | 0.4 | 0.9 | **1.0** | 1.1 | 0.8 |

**Table F9.** Calculated values of femur outside-diameters (*d_mi_* mm) in reference points from Fig. F2 assumed for reference ages.

| Age | 15.6% | | 32% | | **Mid-point 50%** | 65% | | 81% | |
| --- | --- | --- | --- | --- | --- | --- | --- | --- | --- |
|  | *d_1_* | *d_2_* | *d_1_* | *d_2_* | ***D_m_*** | *d_1_* | *d_2_* | *d_1_* | *d_2_* |
| 0 | 7.7 | 11.6 | 6.8 | 8.2 | 6.8 | 7.5 | 7.1 | 8.5 | 8.0 |
| 1 | 12.0 | 18.0 | 10.6 | 12.7 | 10.6 | 11.7 | 11.0 | 13.3 | 12.5 |
| 5 | 17.2 | 25.6 | 15.3 | 18.4 | 14.6 | 16.3 | 15.2 | 24.0 | 18.4 |
| 10 | 14.6 | 26.8 | 15.0 | 17.9 | 14.6 | 14.7 | 15.3 | 18.4 | 18.9 |
| 15 | 20.4 | 30.2 | 20.3 | 20.6 | 19.5 | 19.3 | 21.0 | 21.2 | 24.9 |

*d_1_* – anterior-posterior direction; *d_2_* – lateral-medial direction.

**Table F10.** Calculated values of femur cortical thickness (*Ct.Th_i_* mm) in reference points from Fig. F2 assumed for reference ages.

| Age | 15.6% | 32% | **Mid-point 50%** | 65% | 81% |
| --- | --- | --- | --- | --- | --- |
| 0 | 0.8 | 1.4 | 2.2 | 1.6 | 1.0 |
| 1 | 1.1 | 1.8 | 2.9 | 2.1 | 1.4 |
| 5 | 2.4 | 3.9 | 3.9 | 5.6 | 3.2 |
| 10 | 1.9 | 2.5 | 3.9 | 4.4 | 2.0 |
| 15 | 2.2 | 4.1 | 4.4 | 4.5 | 4.4 |

Uncertainty values of *Ct.Th_i_* and *d_mi_* estimates were taken the same as for mid-point of diaphysis (in terms of CV).

**Femur 0–1 Y, segmentation and estimation of model parameters**

Femur has the shape of a complex tube; at the ends, the diameters are much larger than in the middle. Three BPSs were used for description of infant femur (Fig. F3), Table F11 summarizes the approaches to BPS parameter derivation:

**Fig. F3.** Infant femur: (a, b) radiograph (x-rays) images (Normal pediatric bone X-ray), (a) inferior view; (b) lateral view; (c) stylized models (BPSs).

BPS 1 (body) was described by round cylinder of height *h_m_* and diameter *d_m_*; cortical layer is located on the walls of the cylinder;

BPS 2 (proximal end) was described by the truncated cone of height *h_e_*; round base of diameter *d_m_* and elliptical base of diameters *d_1d_* and d*_2d;_* cortical layer is located on the walls of the cylinder;

BPS 3 (distal end) was described by the truncated cone of height *h_e_*; round base of diameter *d_m_* and elliptical base of diameters *d_1d_* and d*_2d;_* cortical layer is located on the walls of the cylinder although thinner than in BPS2.

**Table F11.** BPS parameter assumed for femur of 0–1 Y (mm).

| BPS | Para-meter | Rationale | 0 Y | | 1 Y | |
| --- | --- | --- | --- | --- | --- | --- |
|  |  |  | M | SD | M | SD |
| #1 | *Ct.Th* | Three point average (32%, 50%, 65%)^a^ | 1.7 | 0.4 | 2.3 | 0.4 |
| #2 | *Ct.Th* | Two times thinner than at reference points 81% | 0.5 | 0.12 | 0.7 | 0.12 |
| #3 | *Ct.Th* | Two times thinner than at reference points 15.6% | 0.4 | 0.1 | 0.6 | 0.1 |
| #1 | *h_m_* | 1/2 maximal diaphysis length *L_mdl_* | 37.8 | 2.05 | 71.5 | 2.5 |
| #1, #2, #3 | *d_m_* | Three-point average (32%, 50%, 65%) | 7.2 | 0.8 | 11.2 | 0.8 |
| #2, #3 | *h_e_* | 1/4 maximal diaphysis length *L_mdl_* | 18.9 | 1 | 35.8 | 1.3 |
| #2, #3 | *d_1d_* | = *D_bc_* | 26.4 | 2.5 | 34 | 4 |
| #2, #3 | d*_2d_* | = *k×d_m_*; k=1.7^b^ | 11.6 | 1.4 | 18.0 | 1.4 |

a- *d_m_* and Ct.Th values in reference-point are presented in the Table F8 and Table F9; b- *k*-value was derived from analysis of infant-bone images (Normal pediatric bone X-ray).

**Femur 5 Y, segmentation and estimation of model parameters**

The shape of the femur diaphysis is complicated with age; in the proximal end, the femoral neck region is distinguished (extended). The femur is modeled by three BPSs (Fig. F4); Table F12 summarizes the approaches to parameter derivation:

**Fig. F4.** Femur of 5-Y-children: (a, b) radiograph (x-rays) images (Normal pediatric bone X-ray); measured parameters are indicated, (a) inferior view; (b) lateral view; (с) stylized models (BPSs).

BPS 1 (body) was described by round cylinder of height *h_m_* and diameter *d_m_*; cortical layer is located on the walls of the cylinder;

BPS2 (proximal end) was described by round cylinder of height *h_e_* and diameter *d_n_*, cortical layer is located on the walls of the cylinder,

BPS3 (distal end) was described by the truncated cone of height *h_e_*; round base of diameter *d_m_* and elliptical base of diameters *d_1d_* and d*_2d_*; cortical layer is located on the walls of the cylinder,

**Table F12.** BPS-parameter estimation for femur of 5-Y (mm).

| BPS | Parameter | Rationale | Assumed value, | SD, mm |
| --- | --- | --- | --- | --- |
| #1 | Ct.Th | 4-point average (32%, 50%, 65%, 81%) ^a^ | 3.7 | 0.3 |
| #2 | Ct.Th | Two times thinner than at reference points 81% ^a^ | 1.3 | 0.18 |
| #3 | Ct.Th | Two times thinner than at reference points 15.6% | 1.1 | 0.08 |
| #1 | *h_m_* | 60% maximal diaphysis length *L_mdl_* | 148.2^b^ | 6.1 |
| #1, #3 | *d_m_* | 4-point average (32%, 50%, 65%, 81%) | 16.6 | 1.0 |
| #2, #3 | *h_e_* | 20% maximal diaphysis length *L_mdl_* | 49.4 | 2.0 |
| #2 | *d_n_* | Measured data | 23 | 7 |
| #3 | *d_1d_* | = *D_bc_* | 68.2 | 4.2 |
| #4 | d*_2d_* | = *k×d_m_*; k=1.7^c^ | 25.0 | 1.8 |

1. *d_m_* and Ct.Th values in reference point are presented in the Table 8 and Table 9; b-Value will be used for weighting procedures, for calculation, the value 30 mm will be used; c- value was derived from analysis of femur images for 5-Y-children (Normal pediatric bone X-ray).

**Femur 10 Y, segmentation and estimation of model parameters**

Three segments were highlighted to describe the proximal and distal parts of femur diaphysis (Fig. F5). The middle part of diaphysis is not modeled, since it does not contain the active marrow (AM). Table F13 summarizes the approaches to parameter derivation.

**Fig. F5.** Femur of 10-Y children: (a – c) radiograph (x-rays) images (Normal pediatric bone X-ray): (a) lateral view; (b) proximal part anterior view; (с) distal part anterior view; (d) stylized models (BPSs).

BPS1 (upper proximal end) was described by round cylinder of height *h_p_* and diameter *d_n,_* cortical layer is located on the walls of the cylinder,

BPS1 (lower proximal end) was described by round cylinder of height *h_p_* and diameter *d_n,_* cortical layer is located on the walls of the cylinder, it is thinner than in BPS1

BPS3 (distal end) was described by the truncated cone of height *h_d_*; round base of diameter *d_m_* and elliptical base of diameters *d_1d_* and d*_2d;_* cortical layer is located on the walls of the cylinder,

**Table F13.** BPS-parameter estimation for femur of 10-Y, mm.

| BPS | Parameter | Rationale | Assumed value | SD |
| --- | --- | --- | --- | --- |
| #1 | *Ct.Th* | Measured data of Petit et al. (2002) | 1.77 | 0.26 |
| #2 | *Ct.Th* | Two times thinner than at reference points 81% ^a^ | 2.2 | 0.3 |
| #3 | *Ct.Th* | Two times thinner than at reference points 15.6% | 1.1 | 0.15 |
| #1, #2 | *h_p_* | 10% maximal diaphysis length *L_mdl_* | 34.6 | 1.9 |
| #1, #2 | *d_n_* | Measured data | 24.9 | 1.7 |
| #3 | *d_m_* | 4-point average (32%, 50%, 65%, 81%) | 21.0 | 1.8 |
| #3 | *h_d_* | 20% maximal diaphysis length *L_mdl_* | 69.2 | 3.8 |
| #3 | *d_1d_* | = *D_bc_* | 78.4 | 5.3 |
| #4 | d*_2d_* | = *k×d_m_*; k=1.7^b^ | 33.2 | 3.2 |

1. *d_m_* and Ct.Th values in reference point are presented in the Table 8 and Table 9; b-value was derived from analysis of femur images children (Normal pediatric bone X-ray).

**Adults, and 15-y analysis of published data on femur macro-parameters and cortical thickness**

Two segments were highlighted to describe the proximal parts of femur diaphysis (Fig F6). The distal part of diaphysis is not modeled, since it does not contain the AM. Tables F14-F22 summarizes the published data on adult femur measurements.


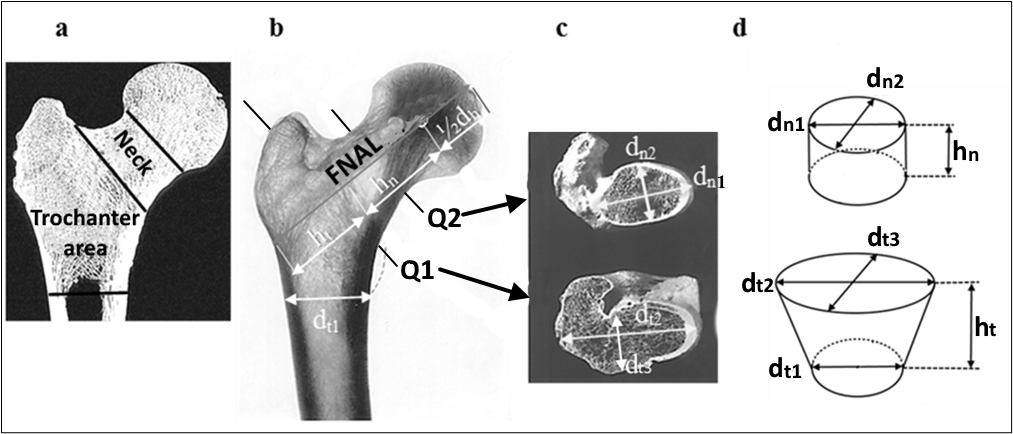


**Fig. F6.** Adult proximal femur: (а) cross-section view and identification of modeled segments; (b) general view and main macro-parameters measured in morphometric study: Q1 – cross-section through the trochanter area; Q2- cross-section through the femoral neck; (c) transverse section of femur neck (upper) trochanter area (lower); (d) stylized models for neck (upper) and trochanter area (lower) (BPSs).

Main measured parameters described in literature which were collected:

- Vertical diameter of the femoral neck (*d_n1_*)
- Transverse diameter of the femoral neck (*d_n2_*)
- Femoral head diameter (*d_h_*)
- Femur neck height (*h_n_*)
- Femur neck axis length (FNAL)- the distance from the base of the greater trochanter to the apex of femoral head through the neck-axis
- Subtrochanter diameter (*d_t1_*)
- Maximal trochanter diameter (*d_t2_*) – distance from the most prominent point of the minor trochanter to the most distant point of the greater trochanter
- Minor trochanter diameter (*d_t3_*) was calculated as *d_t3=_ d_t2_*,/1,5
- Height of trochanter area (*h_t_*) was calculated as *h_t_=*FNAL*-1/2d_h_-h_n_*

BPS1 (neck) was described by elliptical cylinder of height *h_n_* and of diameter *d_n1,_ d_n2;_* cortical layer is located on the walls of the cylinder;

BPS2 (trochanter area) was described by truncated cone of height *ht*; round base of diameter *d_tl_* and elliptical base of diameters *d_1d_* and d*_2d;_* cortical layer is located on the walls of the cylinder,

**All BPS parameters in a 15-year-old man and a 15-year-old woman were considered the same as in adults, with the exception of the height of the segments. Heights of trochanter and neck were calculated as height of segment for adult multiplied by the ratio *L_mdl(15-Y)_/L_mdl(adult);_* values of *L_mdl(adult)_* and *L_mdl(15-Y)_* are shown in the table F6.**

**Table F14.** Published data on femur head diameter (*d_h_*, mm) for adult male

| Author | Age | n | *d_h_* | SD |
| --- | --- | --- | --- | --- |
| Baharuddin et al. 2011 | 25±5 | 30 | 43.62 | 3.05 |
| Mostafa et al. 2012 | 39,8±10 | 24 | 59.12 | 2.45 |
| Takale and Bagal 2016 | Adults | 50 | 45.0 | 0.0 |
| Lv et al. 2012 | 70,5 (66-75) | 51 | 48.26 | 1.961 |
| Chowdhury et al. 2012 | Adults | 89 | 58.3 | 3.0 |
| Clavero et al. 2015 | Adults | 56 | 47.0 | 2.75 |
| Asha et al. 2014 | Adults | 70 | 37.3 | 2.83 |
| Sikka and Jain 2016 | Adults | 115 | 43.9 | 2.55 |
| Timonov et al. 2015 | Adults | 82 | 47.9 | 2.47 |
| Lee et al. 2014 | 53 (21-62) | 50 | 48 | 2.7 |
| Prasad et al. 1996 | 40 (25-55) | 94 | 43 | 3.8 |
| Cho et al. 2015 | 50 | 88 | 48.5 | 2.23 |
| Deshwal et al. 2013 | Adults | 84 | 45.83 | 1.88 |
| Dewo et al. 2016 | 20-70 | 50 | 47.22 | 3.24 |
| Chauhan et al. 2002 | 50-70 | 18 | 45.64 | 3.13 |
| Monisha and Karpagam 2016 | Adults | 20 | 43.2 | 1.47 |
| Yang et al. 2014 | 31,4 (20-45) | 40 | 48.06 | 1.97 |
| Howale et al. 2016 | Adults | 50 | 44.45 | 2.83 |
| Prasath and Ismail 2014 | Adults | 70 | 44.18 | 2.98 |
| Kukadiya et al. 2014 | Adults | 60 | 44.15 | 2.61 |
| **Average values assumed for BPS (CV%)** | | | ***d_h_*=51.2 (4)** | |

**Table F15.** Published data on femur neck width (*d_n_*), mm and length (*h_n_*) for adult male

| Author | Age | n | *d_n_*_1_ | SD | *d_n_*_2_ | SD | *h_n_* | SD |
| --- | --- | --- | --- | --- | --- | --- | --- | --- |
| Asha et al. 2014 | Adults | 70 | 43.05 | 4.59 | 25.9 | 2.9 | - | - |
| Baharuddin at al. 2011 | 25±5 | 30 | 28.88 | 3.38 | - | - | - | - |
| Chowdhury et al. 2012 | Adults | 89 | 46.9 | 1.9 | 46.7 | 1.7 | - |  |
| Clavero et al. 2015 | Adults | 56 | 37.99 | 2.34 | 28.4 | 2.9 | - |  |
| Lee et al. 2013 | 53 (21-62) | 50 | 35.4 | 2.5 | - | - | - | - |
| Linwei et al. 2011 | 70.5 (66-75) | 51 | 35.7 | 2.97 | - | - | - |  |
| Mustafa et al. 2012 | 39.8±10 | 24 | 41.88 | 2.69 | - |  | - |  |
| Nissen et al. 2005 | 44 (19-78) | 94 | 38 | 3 | - |  | - |  |
| Prasad et al. 1996 | 40 (25-55) | 94 | - |  | - | - | 30.5 | 4.1 |
| Ripamonti et al. 2014 | 68.4±9.5 | 228 | 37 | 3 | - | - | - |  |
| Sikka and Jain 2016 | Adults | 115 | 31.3 | 2.1 | - | - | - |  |
| Takale and Bagal 2016 | Adults | 50 | 31.96 | - | 25.98 | - | - |  |
| Timonov et al. 2015 | Adults | 82 | 34.12 | 2.51 | - | - | - |  |
| **Average values assumed for BPS (CV%)** | | | ***d_n1_*=36.0 (6); *d_n2_*=32.0(4); *h_n_*=30.5(13)** | | | | | |

**Table F16.** Published data on femur trochanter for adult male

| Author | Age | n | *d_t1_* | SD | *d_t2_* | SD |
| --- | --- | --- | --- | --- | --- | --- |
| Brown 2006 | Adults | 168 | 26.17 | 1.96 | - |  |
| Lee et al. 2013 | 53 (21-62) | 50 | - |  | 69.6 | 4.6 |
| Prasad et al. 1996 | 40 (25-55) | 94 | - |  | 60.5 | 5.9 |
| Slaus 1997 | Adults | 80 | 29.55 | 2.29 | - |  |
| Young et al. 2013 | 32±6 | 40 | 37 | 3.8 | - |  |
| **Average values assumed for BPS (CV%)** | | | ***d_t1_*=30.0 (7); *d_t2_*=66.0 (6)** | | | |

*d_t3_* was calculated as *d_t3=_ d_t2_,/1,5=44 (6)*

**Table F17.** Published data on FNAL for adult male, mm

| Author | Age | n | M | SD |
| --- | --- | --- | --- | --- |
| Christiensen et al. 2014 | Adults | 4236 | 99.4 | 5.7 |
| Meeusen et al. 2015 | Adults | 54 | 99.72 | 5.79 |
| Nissen et al. 2009 | 44 (19-78) | 94 | 109.0 | 7.0 |
| Pick et al. 1941 | Adults | 75 | 98.23 | 77-13 |
| Pires et al. 2012 | Adults | 177 | 115.3 | 9.3 |
| Steppacher et al. 2017 | Adults | na | 93.0 | 4.0 |
| **Average value (CV%)** | | | **FNAL=99.0 (8)** | |

**Average values assumed for BPS (CV%):** *h_t_=*FNAL*-1/2d_h_-h_n_;* ***h_t_=43.0 (8)***

**Table F18.** Published data on femur head diameter (*d_h_*), mm for adult female

| Author | Age | n | *d_h_* | SD |
| --- | --- | --- | --- | --- |
| Chowdhury et al. 2012 | Adults | 30 | 43.62 | 3.05 |
| Clavero et al. 2015 | 20-58 | 24 | 59.12 | 2.45 |
| Asha et al. 2014 | Adults | 50 | 45.0 | 0.0 |
| Brown 2006 | Adults | 51 | 48.26 | 1.961 |
| Baharuddin et al. 2011 | 25±5 | 89 | 58.3 | 3.0 |
| Chauhan et al. 2002 | 50 | 56 | 47.0 | 2.75 |
| Sikka, Jain 2016 | Adults | 70 | 37.3 | 2.83 |
| Timonov et al. 2015 | Adults | 115 | 43.9 | 2.55 |
| Lee et al. 2013 | 53(21-62) | 82 | 47.9 | 2.47 |
| Srivastava et al. 2012 | 25-67 | 50 | 48 | 2.7 |
| Nidugala et al. 2013 | 25-65 | 94 | 43 | 3.8 |
| Cho et al. 2015 | 54 | 88 | 48.5 | 2.23 |
| Deshwal et al. 2013 | Adults | 84 | 45.83 | 1.88 |
| Dewo et al. 2016 | 20-70 | 50 | 47.22 | 3.24 |
| Monisha, Karpagam 2016 | Adults | 18 | 45.64 | 3.13 |
| Yang et al. 2014 | 31(20-45) | 20 | 43.2 | 1.47 |
| Howale et al. 2016 | Adults | 40 | 48.06 | 1.97 |
| Prasath and Ismail 2014 | Adults | 50 | 44.45 | 2.83 |
| Chandran 2010 | Adults | 70 | 44.18 | 2.98 |
| Kukadiya et al. 2014 | Adults | 60 | 44.15 | 2.61 |
| Varma et al. 2013 | 40-70 | 20 | 39.95 | 1.44 |
| Kamdi et al. 2016 | 30-70 | 20 | 42.3 | 2.39 |
| Pandey and Gaikwad 2016 | Adults | 19 | 41.57 | 2.31 |
| Soni et al. 2010 | Adults | 40 | 39.89 | 2.37 |
| Nirmale 2017 | Adults | 26 | 37.44 | 1.94 |
| Prasad et al. 1996 | 40(20-55) | 77 | 39.1 | 2.6 |
| **Average values assumed for BPS (CV%)** | | | ***d_h_*=40.4 (6)** | |

**Table F19.** Published data on femur trochanter for adult female

| Author | Age | n | *d_t1_* | SD | *d_t2_* | SD |
| --- | --- | --- | --- | --- | --- | --- |
| Brown 2006 | Adults | 65 | 27.4 | 2.25 | - | - |
| Lee et al. 2013 | 53(21-62) | 50 | - | - | 62.1 | 3.7 |
| Prasad et al. 1996 | 25-55 | 77 | - | - | 54.7 | 6.3 |
| Slaus 1997 | Adults | 80 | 25.95 | 2.0 | - | - |
| **Average values assumed for BPS (CV%)** | | | **d_t1_=26.7 (6); d_t2_=58.0 (7)** | | | |

*d_t3_* was calculated as *d_t3=_ d_t2_,/1,5=39 (7)*

**Table F20.** Published data on femur neck width (*d_n_*), mm and length (*h_n_*) for adult female

| Author | Age | n | *d_n_*_1_ | SD | *d_n_*_2_ | SD | *h_n_* | SD |
| --- | --- | --- | --- | --- | --- | --- | --- | --- |
| Asha et al. 2014 | Adults | 70 | 38.2 | 3.35 | 22.9 | 2.9 | - | - |
| Baharuddin at al. 2011 | 25±5 | 30 | 25.95 | 4.31 | - | - | - | - |
| Brown 2006 | Adults | 25 | 31.05 | 3.73 | 24.47 | 1.94 | - | - |
| Chauhan et al. 2002 | 50 | 70 | 26 | 2 | - | - | - | - |
| Chowdhury et al. 2012 | Adults | 110 | 28.3 | 2.1 | 25.5 | 1.1 | - | - |
| Clavero et al. 2015 | 20-58 | 58 | 32.57 | 1.92 | 24.76 | 2.29 | - | - |
| Lee et al. 2013 | 53(21-62) | 50 | 30.5 | 2.1 | - | - | - | - |
| Maeda et al. 2011 | 66-93 | 14 | - | - | - | - | 29.3 | 3.18 |
| Maske et al. 2012 | Adults | 179 | - | - | 21.1 | 2 | - | - |
| Nidugala et al. 2013 | 25-65 | 35 | 30.3 | 3.76 | - | - | - | - |
| Nissen et al. 2005 | 47(21-79) | 155 | 33 | 3 | - | - | - | - |
| Prasad et al. 1996 | 40(20-55) | 77 | - | - |  | - | 26.4 | 4 |
| Ravi et al. 2016 | Adults | 592 | - | - |  | - | 36.3 | 5.4 |
| Sikka and Jain 2016 | Adults | 65 | 26.4 | 2.8 | - | - | - | - |
| Srivastava et al. 2012 | 25-67 | 28 | 26.5 | 1.6 | - | - | - | - |
| Takale and Bagal 2016 | Adults | 50 | 29.5 | - | 24.79 | - | - | - |
| Timonov et al. 2015 | Adults | 58 | 28.98 | 3.36 | - | - | - | - |
| Vaghefi et al. 2015 | 20-40 | 60 | 24.9 | 2.6 | - | - | - | - |
| **Average values assumed for BPS (CV%)** | | | ***d_n1_*=29.4 (10); *d_n2_*=23.9 (9); *h_n_*=30.9 (14)** | | | | | |

**Table F21.** Published data on FNAL for adult female, mm

| Author | Age | n | M | SD |
| --- | --- | --- | --- | --- |
| Christiensen et al. 2014 | Adults | 46134 | 88.2 | 5.1 |
| Meeusen et al. 2015 | Adults | 54 | 88.02 | 5.4 |
| Nidugala et al. 2013 | 25-65 | 35 | 79.78 | 6.7 |
| Nissen et al. 2009 | 47(21-79) | 155 | 95.0 | 6.0 |
| Srivastava et al. 2012 | 25-67 | 28 | 75.29 | 5.8 |
| **Average value (CV%)** | | | **FNAL=85.6 (7)** | |

**Average values assumed for BPS (CV%):** *h_t_=*FNAL*-1/2d_h_-h_n_;* ***h_t_=34.5 (7)***

**Table F22.** Published data on Ct.Th. for adults, mm

| Author | Age | n | Neck | | Trochanter | |
| --- | --- | --- | --- | --- | --- | --- |
|  |  |  | M | SD | M | SD |
| Malo et al. 2013 | 47.1 (17-82) | 1 | 1.85 | 0.18 | - | - |
| Johannesdottir et al. 2013 | 74 (69-90) | 100 | 2.05 | 0.65 | - | - |
| Spencer and Scholar 2015(m+f) | 50 (20-80) | 24 | 1.95 | 0.3 | - | - |
| Yang et al. 2012 | >65 | 250 | - | - | 2.28 | 1.2 |
| **Average values assumed for BPS (CV%)** | | | **Ct.th (neck)=1.95 (5);**  **Ct.th (trochanter)=2.3 (52)** | | | |

**Analysis of published data on femur microstructures**

**Table F23.** Published individual data on microstructure of proximal femur for pre-adults.

| Author | Individual cod* | Age | BV/TV, r.u. | SD BV/TV | Tb.Th, mm | SD Tb.Th |
| --- | --- | --- | --- | --- | --- | --- |
| Salle et al. 2002 | - | 0 | 0.336 | 0.051 | 0.093 | 0.009 |
| Ryan et al. 2006 | 73 | 0 | 0.526 | 0.094 | 0.114 | 0.029 |
| Ryan et al. 2006 | 152 | 0 | 0.502 | 0.066 | 0.110 | 0.018 |
| Ryan et al. 2006 | 12 | 0.04 | 0.483 | 0.045 | 0.120 | 0.020 |
| Ryan et al. 2006 | 99 | 0.08 | 0.514 | 0.083 | 0.137 | 0.035 |
| Milovanovic et al. 2017 | - | 0.1 | 0.348 | 0.103 | 0.126 | 0.026 |
| Ryan et al. 2006 | 126 | 0.25 | 0.569 | - | 0.143 | - |
| Milovanovic et al. 2017 | - | 0.3 | 0.189 | 0.025 | 0.118 | 0.017 |
| Ryan et al. 2006 | 133 | 0.38 | 0.413 | 0.141 | 0.130 | 0.035 |
| Ryan et al. 2006 | 201 | 0.38 | 0.348 | 0.046 | 0.120 | 0.013 |
| Milovanovic et al. 2017 | - | 0.4 | 0.096 | 0.023 | 0.090 | 0.011 |
| Ryan et al. 2006 | 172 | 0.5 | 0.257 | 0.079 | 0.171 | 0.001 |
| Ryan et al. 2006 | 202 | 0.5 | 0.223 | - | 0.162 | - |
| Ryan et al. 2006 | 85 | 0.63 | 0.313 | - | 0.170 | - |
| Milovanovic et al. 2017 | - | 0.7 | 0.174 | 0.047 | 0.131 | 0.021 |
| Ryan et al. 2006 | 57 | 0.75 | 0.227 | - | 0.168 | - |
| Ryan et al. 2006 | 140 | 0.75 | 0.287 | 0.212 | 0.135 | 0.058 |
| Ryan et al. 2006 | 142 | 0.75 | 0.303 | 0.038 | 0.154 | 0.033 |
| Ryan et al. 2006 | 167 | 0.75 | 0.320 | - | 0.188 | - |
| Ryan et al. 2006 | 247 | 0.75 | 0.180 | - | 0.165 | - |
| Milovanovic et al. 2017 | - | 0.8 | 0.168 | 0.050 | 0.154 | 0.034 |
| Milovanovic et al. 2017 | - | 0.9 | 0.167 | 0.035 | 0.141 | 0.024 |
| Ryan et al. 2006 | 58 | 1 | 0.202 | - | 0.161 | - |
| Ryan et al. 2006 | 117 | 1.5 | 0.151 | 0.009 | 0.124 | 0.044 |
| Ryan et al. 2006 | 131 | 1.5 | 0.203 | - | 0.172 | - |
| Ryan et al. 2006 | 135 | 1.5 | 0.210 | 0.044 | 0.136 | 0.030 |
| Ryan et al. 2006 | 162 | 1.5 | 0.169 | - | 0.204 | - |
| Ryan et al. 2006 | 221 | 1.5 | 0.203 | - | 0.202 | - |
| Milovanovic et al. 2017 | - | 1.6 | 0.275 | 0.067 | 0.175 | 0.028 |
| Ryan et al. 2006 | 79 | 1.75 | 0.308 | - | 0.194 | - |
| Ryan et al. 2006 | 88 | 2 | 0.431 | - | 0.295 | - |
| Ryan et al. 2006 | 124 | 2 | 0.331 | - | 0.328 | - |
| Ryan et al. 2006 | 171 | 2 | 0.370 | - | 0.285 | - |
| Milovanovic et al. 2017 | - | 2 | 0.182 | 0.024 | 0.127 | 0.008 |
| Ryan et al. 2006 | 65 | 2.3 | 0.217 | 0.043 | 0.149 | 0.026 |
| Ryan et al. 2006 | 153 | 2.5 | 0.349 | 0.092 | 0.212 | 0.052 |
| Ryan et al. 2006 | 160 | 2.5 | 0.228 | - | 0.212 | - |
| Ryan et al. 2006 | 177 | 2.5 | 0.339 | - | 0.266 | - |
| Milovanovic et al. 2017 | - | 2.8 | 0.243 | 0.071 | 0.164 | 0.028 |
| Ryan et al. 2006 | 25 | 3 | 0.348 | - | 0.275 | - |
| Ryan et al. 2006 | 76 | 3 | 0.339 | - | 0.259 | - |
| Ryan et al. 2006 | 161 | 3 | 0.232 | - | 0.245 | - |
| Milovanovic et al. 2017 | - | 3 | 0.358 | 0.049 | 0.185 | 0.027 |
| Ryan et al. 2006 | 183 | 3.5 | 0.426 | - | 0.344 | - |
| Milovanovic et al. 2017 | - | 4.3 | 0.361 | 0.041 | 0.185 | 0.018 |
| Continuation | | | | | | |
| Author | Individual cod* | Age | BV/TV, r.u | SD BV/TV | Tb.Th, mm | SD Tb.Th |
| Ryan et al. 2006 | 115 | 5 | 0.290 | 0.075 | 0.159 | 0.050 |
| Ryan et al. 2006 | 30 | 5.5 | 0.364 | - | 0.283 | - |
| Milovanovic et al. 2017 | - | 5.7 | 0.232 | 0.040 | 0.147 | 0.019 |
| Ryan et al. 2006 | 29 | 6.5 | 0.368 | - | 0.296 | - |
| Milovanovic et al. 2017 | - | 6.5 | 0.390 | 0.040 | 0.223 | 0.028 |
| Milovanovic et al. 2017 | - | 7.5 | 0.356 | 0.076 | 0.227 | 0.026 |
| Milovanovic et al. 2017 | - | 7.6 | 0.339 | 0.044 | 0.223 | 0.020 |
| Ryan et al. 2006 | 260 | 8 | 0.375 | - | 0.280 | - |
| Milovanovic et al. 2017 | - | 8.7 | 0.424 | 0.110 | 0.246 | 0.044 |
| Milovanovic et al. 2017 | - | 9.7 | 0.358 | 0.066 | 0.246 | 0.036 |
| Ryan et al. 2006 | 89 | 10.5 | 0.403 | - | 0.326 | - |
| Milovanovic et al. 2017 | - | 12 | 0.416 | 0.067 | 0.262 | 0.060 |
| Ryan et al. 2006 | 113 | 15 | 0.396 | 0.028 | 0.201 | 0.021 |
| Pafundi 2009 | - | 18 | 0.244 | - | - | - |

* - Original burial number from Ryan et al. (2006, 2017);

Ryan et al. 2006, Milovanovic et al. 2017, Pafundi 2009 shows the individual data, SD of individual data reflects the inter-specimen variability within individual bone.

**Table F24.** Published data on microstructure of proximal femur for adults.

| Author | n | Age | BV/TV | SD BV/TV | Tb.Th | SD Tb.Th | Tb.Sp | SD Tb.Sp |
| --- | --- | --- | --- | --- | --- | --- | --- | --- |
| Femoral neck | | | | | | | | |
| Djuric et al. 2010 | 10 | 26-39 | 0.1522 | 0.04 | 0.215 | 0.03 | 0.745 | 0.09 |
| Turunen et al. 2013 | 27 | 17-82 | 0.18 | 0.07 | 0.18 | 0.03 | 0.789 | 0.11 |
| Trochanter area | | | | | | | | |
| Djuric et al. 2010 | 10 | 26-39 | 0.101 | 0.06 | 0.195 | 0.04 | 0.83 | 0.05 |
| Fazzalari et al. 2001 | 13 | 20-85 | 0.102 | 0.05 | 0.087 | 0.024 | 1.02 | 0.31 |
| Turunen et al. 2013 | 27 | 17-82 | 0.12 | 0.03 | 0.147 | 0.018 | 0.701 | 0.1 |
| Truong et al. 2006 | 13 | 44-71 | 0.076 | 0.03 | 0.111 | 0.03 | 1.4 | 0.36 |
| Tsangari et al. 2006 | 13 | 18-50 | 0.139 | 0.04 | 0.14 | 0.05 | - | - |

**Table F25.** Measured data on trabecular space, mm (Milovanovic et al. 2007, Ryan et al. 2017).

| Age range | Average | Tb.Sp_SD | Min | Max | n |
| --- | --- | --- | --- | --- | --- |
| 0.0–0.5 | 0.388 | 0.103 | 0.2498 | 0.529 | 5 |
| 0.6–1.5 | 0.538 | 0.110 | 0.337 | 0.813 | 16 |
| 1.6–2.9 | 0.547 | 0.078 | 0.43 | 0.691 | 11 |
| 3.0–12.0 | 0.533 | 0.078 | 0.4108 | 0.691 | 18 |

**Parameters for the distal part**

Data for the distal femur satisfying the analysis conditions were found only for adults: Saers et al. (2016) presented a comparison of microparameters of the distal and proximal femur in three human populations. In all cases, within the same population, significant differences were found between BV/TV of proximal and distal ends. Differences amounted to 25–30% and were revealed both on data averaged over three populations and within individual populations. There were no significant differences in Tb.Th. Thus, for the distal section, the same Tb.Th values are accepted as for the proximal. As for BV/TV, for people 5 years and older it was accepted that in the distal part, BV/TV is 25% lower than in the proximal part. It is assumed that the variability of BV/TV and Tb.Th (SD value) is equal to that for the proximal.

**Table F26.** Ratio BV/TV, Tb.Th and Tb.Sp assumed for femur in SPSD-model.

| Age | Subsite | BV/TV r.u (min–max) | SD | Tb.Th, mm  (min–max) | SD, mm | Tb.Sp, mm  (min–max) | SD, mm |
| --- | --- | --- | --- | --- | --- | --- | --- |
| 0 | Femur | 0.37  (0.096–0.529) | 0.16 | 0.11  (0.09–0.143) | 0.017 | 0.388  (0.25–0.529) | 0.103 |
| 1 | Femur | 0.22  (0.151–0.32) | 0.072 | 0.16  (0.124–0.204) | 0.06 | 0.538  (0.337–0.813 | 0.110 |
| 5 | Proximal | 0.35  (0.23–0.426) | 0.059 | 0.24  (0.147–0.344) | 0.053 | 0.538  (0.411–0.631) | 0.077 |
|  | Distal | 0.26  (0.174–0.32) | 0.059 | 0.24  (0.147–0.344) | 0.053 | 0.538  (0.411–0.631) | 0.077 |
| 10 | Proximal | 0.35  (0.23–0.426) | 0.059 | 0.24  (0.147–0.344) | 0.053 | 0.538  (0.411–0.631) | 0.077 |
|  | Distal | 0.26  (0.174–0.32) | 0.059 | 0.24  (0.147–0.344) | 0.053 | 0.538  (0.411–0.631) | 0.077 |
| 15 | Neck | 0.35  (0.23–0.426) | 0.059 | 0.24  (0.147–0.344) | 0.053 | 0.538  (0.411–0.631) | 0.077 |
|  | Trochanter area | 0.26  (0.174–0.32) | 0.059 | 0.24  (0.147–0.344) | 0.053 | 0.538  (0.411–0.631) | 0.077 |
| Adults | Neck | 0.17  (0.12-0.24) | 0.06 | 0.136  (0.09-0.19) | 0.03 | 0.99  (0.66-1.32) | 0.2 |
|  | Trochanter area | 0.11  (0.06-0.16) | 0.04 | 0.19  (0.16-0.2) | 0.02 | 0.78  (0.62-0.94) | 0.1 |

**Reference for femur**

Asha KR, Vinaykumar K, Bindurani MK, Kavyashree AN, Lakshmiprabha S. Reconstruction of Femoral Length from Its Proximal Fragments and Diaphyseal Segments in South Indian Population. Research Journal of Pharmaceutical, Biological and Chemical Sciences. 2014; 5(5): 920.

Baker BJ, Dupras TL, Tocheri MW, Wheeler SM. Osteology of infants and children. Texas A&M University Press. 2005. <https://muse.jhu.edu/book/2769>

Haas J, Buikstra JE, Ubelaker DH, Aftandilian D, History FMoN, Survey AA. Standards for Data Collection from Human Skeletal Remains: Proceedings of a Seminar at the Field Museum of Natural History, Organized by Jonathan Haas: Arkansas Archeological Survey; 1994.

Baharuddin MY, Kadir MRA, Zulkifly AH, Saat A, Aziz AA, Lee MH. Morphology Study of the Proximal Femur in Malay Population. Int. J. Morphol. 2011; 29(4): 1321-1325.

Brown JL Morphological variation of the proximal femur in selected skeletal remains. Thesis. B.S. Michigan State University. 2006.

Chandran M. Reconstruction of Femur Length from Its Fragments in South Indian Females. International Journal of Medical Toxicology and Forensic Medicine. 2011; 1(2): 45-53.

Chauhan R, Paul S, Dhaon B.K. Anatomical Parameters of North Indian Hip Joints – Cadaveric Study. J. Anat. Soc. India. 2002; 51(1): 39-42.

Cho Ho-Jung, Kwak Dai-Soon, Kim In-Beom. Morphometric Evaluation of Korean Femurs by Geometric Computation: Comparisons of the Sex and the Population. Biomed Res Int. 2015. 730538.

Chowdhury Sh, Naushaba H, Chowdhury MM, Khan LF, Ara JG. Morphometric study of fully ossified head and neck diameter of the human left femur. Journal of Dhaka National Medical College & Hospital, 2012; 18(02): 9-13.

Christensen AM, Leslie WD, Baim S. Ancestral differences in femoral neck axis length: possible implications for forensic anthropological analyses. Forensic Sci Int. 2014;236:193.e1-193.e1934. doi:10.1016/j.forsciint.2013.12.027

Clavero A, Salicrú M, Turbón D. Sex prediction from the femur and hip bone using a sample of CT images from a Spanish population. International Journal of Legal Medicine. 2015; 129(2): 373–383.

Demidov VN, Bychkov PA, Logvinenko AV, Voevodin SM. Ul'trazvukovaja biometrija. Spravochnye tablicy i uravnenija. V knige: Medvedeva M. V., Zykina B. I., redaktory. Klinicheskie lekcii po UZ-diagnostike v perinatologii. M. 1990. 83–92.

Deshwal AKr, Tripathi A, Bhatnagar S. Comparative measurement of diameter of femoral head in population of western uttar paradesh. IJSR. 2013; 2(12): 437-439.

Dewo P, Suyitno, Dharmastiti R, Salim UA, Hidayat L, Wibowo PA, Lanodiyu ZA, Magetsari R. Three Dimensional Morphometry of Proximal Femur to Design Best-Fit Femoral Stem for Indonesian Population. Int. J. Morphol. 2016; 34(2): 436-442.

Dhavale N, Halcrow SE, Buckley HR, Tayles N, Domett KM, Gray AR. Linear and appositional growth in infants and children from the prehistoric settlement of Ban Non Wat, Northeast Thailand: Evaluating biological responses to agricultural intensification in Southeast Asia, Journal of Archaeological Science: Reports, 2017; V11: 435–446. ISSN 2352-409.

Djuric M, Djonic D, Milovanovic P, Nikolic S, Marshall R, Marinkovic J, Hahn M. Region-specific sex-dependent pattern of age-related changes of proximal femoral cancellous bone and its implications on differential bone fragility. Calcif Tissue Int. 2010; 86(3):192–201.

Djurić M, Milovanović P, Djonić D, Minić A, Hahn M. Morphological characteristics of the developing proximal femur: a biomechanical perspective. Srp Arh Celok Lek. 2012 Nov-Dec; 140(11–12):738–745. PubMed PMID: 23350248

[Fazzalari NL](https://www.ncbi.nlm.nih.gov/pubmed/?term=Fazzalari%20NL%5BAuthor%5D&cauthor=true&cauthor_uid=11393778), [Kuliwaba JS](https://www.ncbi.nlm.nih.gov/pubmed/?term=Kuliwaba%20JS%5BAuthor%5D&cauthor=true&cauthor_uid=11393778), [Atkins GJ](https://www.ncbi.nlm.nih.gov/pubmed/?term=Atkins%20GJ%5BAuthor%5D&cauthor=true&cauthor_uid=11393778), [Forwood MR](https://www.ncbi.nlm.nih.gov/pubmed/?term=Forwood%20MR%5BAuthor%5D&cauthor=true&cauthor_uid=11393778), [Findlay DM](https://www.ncbi.nlm.nih.gov/pubmed/?term=Findlay%20DM%5BAuthor%5D&cauthor=true&cauthor_uid=11393778). The ratio of messenger RNA levels of receptor activator of nuclear factor kappaB ligand to osteoprotegerin correlates with bone remodeling indices in normal human cancellous bone but not in osteoarthritis. [J Bone Miner Res.](https://www.ncbi.nlm.nih.gov/pubmed/?term=Fazzalari+NL+RNA+level+2001+bone) 2001; 16(6):1015–1027.

Florence JL. Linear and cortical bone dimensions as indicators of health status in subadults from the Milwaukee County Poor Farm Cemetery. M.A., University of Colorado at Denver. 2007.

Gosman JH, Ketcham RA. Patterns in ontogeny of human trabecular bone from SunWatch Village in the Prehistoric Ohio Valley: general features of microarchitectural change. Am J Phys Anthropol. 2009 Mar;138(3):318–32. doi:10.1002/ajpa.20931. PubMed PMID: 18785633.

Howale DS, Tandel MR, Ramawat MR, Pandit DP, Madole MB. Determination of Sex from adult human femur from South Gujarat region. International Journal of Anatomy and Research. 2016; 4(4): 3044-3047.

Jeanty P. Fetal limb biometry. Radiology. 1983 May;147(2):601-2. doi: 10.1148/radiology.147.2.6836145. PMID: 6836145.

Johannesdottir F, Aspelund T, Reeve J, Poole KE, Sigurdsson S, Harris TB, Gudnason VG, Sigurdsson G. Similarities and differences between sexes in regional loss of cortical and trabecular bone in the mid-femoral neck: the AGES-Reykjavik longitudinal study. J Bone Miner Res. 2013; 28(10):2165-76.

Kamdi A, Kaore A, Saritha S. Study of vertical diameter of head of femur in South Indian cadavets. Int J Anat Res 2016; 4(3):2730-33.

Kanellopoulos AD, Yiannakopoulos CK, Soucacos PN. Closed, locked intramedullary nailing of pediatric femoral shaft fractures through the tip of the greater trochanter. J Trauma. 2006; Jan;60(1):217–22; discussion 222-3. PubMed PMID: 16456459.

Kukadiya UC, Singel TC, Trivedi PN, Rathava JK, Satapara VK, Gohil DV, Patel MM. Sex determination from vertical diameter of femoral head in Gujarati population. International Journal of Advanced Research. 2014; 2(6):859-863.

Lee JH, Kim YS, Jeong YG, Lee NS, Han SY, Tubbs RS, Han SH. Sex determination from partial segments and maximum femur lengths in Koreans using computed tomography. Folia Morphol (Warsz). 2014; 73(3):353-358.

Lv L, Meng G, Gong H, Zhu D, Zhu W. A new method for the measurement and analysis of three-dimensional morphological parameters of proximal Male femur. Biomedical Research. 2012; 23(2).

Maeda Y, Sugano N, Saito M, Yonenobu K. Comparison of femoral morphology and bone mineral density between femoral neck fractures and trochanteric fractures. Clin Orthop Relat Res. 2011; 469(3):884-889. doi:10.1007/s11999-010-1529-8

Malo MK, Rohrbach D, Isaksson H, Töyräs J, Jurvelin JS, Tamminen IS, Kröger H, Raum K. Longitudinal elastic properties and porosity of cortical bone tissue vary with age in human proximal femur. Bone. 2013; 53(2):451-458.

Maresh MM. Measurements from roentgenograms. In: Human Growth and Development (R.W. McCammon, Ed.), 1970; 157–200. Springfield, IL: Charles C. Thomas.

Maske SS, Kamble P, Joshi DS. Sexing the femora from Marathwada region using demarcating point method. International J. of Healthcare & Biomedical Research. 2012; 1(1):13-16

Medvedev MV Ed. Ultrasonic Fetometry: Reference Tables and Nomograms Ed. 8th, rev. Moscow: Real time Publisher. 2009: 19–24 (in Russian).

Meeusen RA, Christensen AM, Hefner JT. The Use of Femoral Neck Axis Length to Estimate Sex and Ancestry. J Forensic Sci. 2015; 60(5):1300-1304.

Miles AEW. Growth Curves of Immature Bones from a Scottish Island Population of Sixteenth to mid-Nineteenth Century: Limb-bone Diaphyses and Some Bones of the Hand and Foot. International Journal of Osteoarcheology. 1994; 4:121–136.

[Milovanovic P](https://www.ncbi.nlm.nih.gov/pubmed/?term=Milovanovic%20P%5BAuthor%5D&cauthor=true&cauthor_uid=28631293), [Djonic D](https://www.ncbi.nlm.nih.gov/pubmed/?term=Djonic%20D%5BAuthor%5D&cauthor=true&cauthor_uid=28631293), [Hahn M](https://www.ncbi.nlm.nih.gov/pubmed/?term=Hahn%20M%5BAuthor%5D&cauthor=true&cauthor_uid=28631293), [Amling M](https://www.ncbi.nlm.nih.gov/pubmed/?term=Amling%20M%5BAuthor%5D&cauthor=true&cauthor_uid=28631293), [Busse B](https://www.ncbi.nlm.nih.gov/pubmed/?term=Busse%20B%5BAuthor%5D&cauthor=true&cauthor_uid=28631293), [Djuric M](https://www.ncbi.nlm.nih.gov/pubmed/?term=Djuric%20M%5BAuthor%5D&cauthor=true&cauthor_uid=28631293). Region-dependent patterns of trabecular bone growth in the human proximal femur: A study of 3D bone microarchitecture from early postnatal to late childhood period. [Am J Phys Anthropol.](https://www.ncbi.nlm.nih.gov/pubmed/?term=Petar+Milovanovic+Region%E2%80%90dependent+patterns) 2017 Oct;164(2):281–291. doi: 10.1002/ajpa.23268. Epub 2017 Jun 20.

Monisha K, Karpagam K. Study of Determination of Sex from Femur Bone. International Journal of Advanced Research. 2016; 4(6):1182-1183.

Mostafa EM, El-Elemi AH, El-Beblawy MA, Dawood Abd El-W. Adult sex identification using digital radiographs of the proximal epiphysis of the femur at Suez Canal University Hospital in Ismailia, Egypt. Egyptian Journal of Forensic Sciences. 2012; 2(3):81–88.

Mustafa MS, Mahmoud OM, El Raouf HH, Atef HM. Morphometric study of sacral hiatus in adult human Egyptian sacra: Their significance in caudal epidural anesthesia. Saudi J Anaesth. 2012 Oct–Dec;6(4):350–7.

Nemec U, Nemec SF, Weber M, Brugger PC, Kasprian G, Bettelheim D, Rimoin DL, Niu J, Feng G, Kong X, Wang J, Han P. Age-related marrow conversion and developing epiphysis in the proximal femur: evaluation with STIR MR imaging. J Huazhong Univ Sci Technolog Med Sci. Oct;27(5):617–21; PubMed PMID: 18060651; 2007.

Nidugala H, Bhaskar B, Suresh S, Avadhani R. Metric assessment of femur using discriminant function analysis in South Indian population. Int J Anat Res, 2013; 02:29 -32.

Nirmale VK, Laeeque M, Diwan CV. Assessment of reliability of various criteria used in adult hip bone sex differentiation. Int J Anat Res 2016;4(4):3185-3189. DOI: 10.16965/ijar.2016.435

Nissen N, Hauge EM, Abrahamsen B, Jensen JEB, Mosekilde L, Brixen K. Geometry of the Proximal Femur in Relation to Age and Sex: A Cross-Sectional Study in Healthy Adult Danes. Acta Radiologica. 2009; 46(5): 514-518.

Normal pediatric bone X-ray, available in: <https://bonexray.com/>; <http://bones.getthediagnosis.org/>; <http://bonepit.com/>

Pafundi D. Image-based skeletal tissues and electron dosimetry models for the ICRP reference pediatric age series. A dissertation presented to the graduate schools of the University of Florida in partial fulfillment of the requirements for the degree of doctor of the philosophy. University of Florida. 2009.

Pandey R and Gaikwad H. Sex determination by discriminant function analysis of femoral heads of a North Indian population. Indian Journal of Forensic and Community Medicine, July-September 2016;3(3):172-17

Petit MA, McKay HA, MacKelvie KJ, Heinonen A, Khan KM, Beck TJ. A randomized school-based jumping intervention confers site and maturity-specific benefits on bone structural properties in girls: a hip structural analysis study. J Bone Miner Res. 2002 Mar;17(3):363–72. PubMed PMID: 11874228.

Pick JW, Stack JK, Anson BJ. Measurements on the human femur - I. lengths, diameters and angles. Q Bull Northwest Univ Med Sch. 1941;15(4):281-290.

Pires RE, Prata EF, Gibram AV, Santos LE, Lourenço PR, Belloti JC. Radiographic anatomy of the proximal femur: correlation with the occurrence of fractures. Acta Ortop Bras. 2012; 20(2):79-83. doi:10.1590/S1413-78522012000200004

Prasad R, Vettivel S, Jeyaseelan L, Isaac B, Chandi G. Reconstruction of Femur Length from Markers of Its Proximal End. Clinical Anatomy. 1996; 9: 28-33.

Prasath RA, Ismail BM. A correlative study of morphometric analysis of acetabulum and femoral head in male and female south Indian human cadavers. Journal of Science. 2014; 4(1): 4-8.

Ripamonti C, Lisi L, Avella M. Femoral neck shaft angle width is associated with hip-fracture risk in males but not independently of femoral neck bone density. The British Journal of Radiology. 2014; 87(1037). PubMed PMID: 20130358. doi:10.1259/bjr.20130358.

Ryan TM, Krovitz GE. [Trabecular bone ontogeny in the human proximal femur.](https://www.ncbi.nlm.nih.gov/pubmed/16963108) J Hum Evol. 2006 Dec;51(6):591–602. Epub 2006 Aug 5.

[Ryan](https://www.cambridge.org/core/search?filters%5BauthorTerms%5D=Timothy%20M.%20Ryan&eventCode=SE-AU) TM, [Raichlen](https://www.cambridge.org/core/search?filters%5BauthorTerms%5D=David%20A.%20Raichlen&eventCode=SE-AU) DA, [Gosman](https://www.cambridge.org/core/search?filters%5BauthorTerms%5D=James%20H.%20Gosman&eventCode=SE-AU) JH. Structural and Mechanical Changes in Trabecular Bone during Early Development in the Human Femur and Humerus. Chapter 12. In: [Building Bones: Bone Formation and Development in Anthropology](https://www.cambridge.org/core/books/building-bones-bone-formation-and-development-in-anthropology/FAA70C0A5554F14B613C13A5F3A1A891). Cambridge University Press 2017; 281–302. <https://doi.org/10.1017/9781316388907.013>

Ravi GO, Shaik Hussain Saheb, Abraham Ratna Joseph N. A Morphometric Study of Femur and Its Clinical Importance. International Journal of Integrative Medical Sciences, Int J Intg Med Sci 2016, 3(7):341-44. ISSN 2394 – 4137 DOI: <http://dx.doi.org/10.16965/ijims.2016.135>

Saers JP, Cazorla-Bak Y, Shaw CN, Stock JT, Ryan TM. Trabecular bone structural variation throughout the human lower limb. J Hum Evol. 2016;97:97-108. doi:10.1016/j.jhevol.2016.05.012

[Salle BL](https://www.ncbi.nlm.nih.gov/pubmed/?term=Salle%20BL%5BAuthor%5D&cauthor=true&cauthor_uid=12052448), [Rauch F](https://www.ncbi.nlm.nih.gov/pubmed/?term=Rauch%20F%5BAuthor%5D&cauthor=true&cauthor_uid=12052448), [Travers R](https://www.ncbi.nlm.nih.gov/pubmed/?term=Travers%20R%5BAuthor%5D&cauthor=true&cauthor_uid=12052448), [Bouvier R](https://www.ncbi.nlm.nih.gov/pubmed/?term=Bouvier%20R%5BAuthor%5D&cauthor=true&cauthor_uid=12052448), [Glorieux FH](https://www.ncbi.nlm.nih.gov/pubmed/?term=Glorieux%20FH%5BAuthor%5D&cauthor=true&cauthor_uid=12052448). Human fetal bone development: histomorphometric evaluation of the proximal femoral metaphysis. [Bone.](https://www.ncbi.nlm.nih.gov/pubmed/?term=Human+fetal+bone+development%3A+histomorphometric+evaluation+of+the+proximal+femoral+metaphysis) 2002 Jun;30(6):823–8.

Sikka A, Jain A. Sex determination of femur: a morphometric analysis in the North NORTH Indian Population. Journal of Evolution of Medical and Dental Sciences. 2016; 5(59): 4056-4059.

Singh SP, Malhotra P, Sidhu LS, Singh PP. (2007). Skeletal Frame Size of Spitian Children. Journal of Human Ecology. 21(3); 227–230

Slaus M. Discriminant function sexing of fragmentary and complete femora from medieval sites in continental Croatia. Opvscvla archaeologica. 1997; 21: 167-175.

Soni G, Dhall U, Chhabra S. Determination of Sex from Femur: Discriminant Analysis. Journal of Anatomical Society of India. 2010; 216-221.

Spencer T, Scholar M. Quantitative Analysis of Cortical and Trabecular Bone in Three Human Populations. McNair Research Journal. 2015; 68–81. [https://www.semanticscholar.org/paper/Quantitative-Analysis-of-Cortical-and-Trabecular-in-Spencer Ryan/b9c6f68f93a60030aeda62879770291bb2088631](https://www.semanticscholar.org/paper/Quantitative-Analysis-of-Cortical-and-Trabecular-in-Spencer%20Ryan/b9c6f68f93a60030aeda62879770291bb2088631)

Srivastava R, Saini V, Rai R.K, Pandey S, Tripathi SK. A study of sexual dimorphism in the femur among North Indians. J Forensic Sci. 2012 Jan; 57(1):19-23.

Steppacher SD, Anwander H, Schwab JM, Siebenrock KA, Tannast M. Femoral Dysplasia. Available at 03.10.2017, https://musculoskeletalkey.com/femoral-dysplasia/

Svadovsky VS. Age-related bone remodeling. Moscow. 1961.

Takale S. and Bagal G. Sex Determination from The Upper End And Length Of The Femur: A Morphometric Study. Journal of Medical Sciences and Clinical Research. 2016; 4(02): 9257-9261.

Timonov P, Fasova A, Badiani K, Radoinova D, Alexandrov A. Sex determination from the femur in a Bulgarian modern population. Anil Aggrawal's Internet Journal of Forensic Medicine and Toxicology. 2015. 16(2): p3-3.

Truong LH, Kuliwaba JS, Tsangari H, Fazzalari NL. Differential gene expression of bone anabolic factors and trabecular bone architectural changes in the proximal femoral shaft of primary hip osteoarthritis patients. Arthritis Res Ther. 2006; 8(6): R188.

Tsangari H, Kuliwaba JS, Fazzalari NL. Trabecular bone modeling and subcapital femoral fracture. J Musculoskelet Neuronal Interact. 2007 Jan-Mar;7(1):69–73.

Turunen MJ, Prantner V, Jurvelin JS, Kröger H, Isaksson H. Composition and microarchitecture of human trabecular bone change with age and differ between anatomical locations. Bone. 2013; 54(1):118–25.

Vaghefi SHE, Elyasi L, Amirian SR, Raigan P, Akbari H, Sheikhshoaiee M, Borbor A. Evaluating Anthropometric Dimensions of the Femur Using Direct and Indirect Methods. 2015; 12(2).

Varma CL, Raju P, Rajeshwari T. Parameters of Hip Joint on Human Cadavers. 1995.

Yang Z, Jian W, Li ZH, Jun X, Liang Z, Ge Y, Shi ZJ. The geometry of the bone structure associated with total hip arthroplasty. PLoS One. 2014; 7;9(3): e91058

Young EY, Gebhart J, Cooperman D, Ahn NU. Are the left and right proximal femurs symmetric? Clin Orthop Relat Res. 2013;471(5):1593-1601. doi:10.1007/s11999-012-2704-x

Zivicnjak M, Smolej Narancić N, Szirovicza L, Franke D, Hrenović J, Bisof V, Tomas Z, Skarić-Jurić T. Gender-specific growth patterns of transversal body dimensions in Croatian children and youth (2 to 18 years of age). Coll Antropol. 2008 Jun;32(2):419–31. PubMed PMID: 18756891
