# Supplementary material for: Stochastic parametric skeletal dosimetry model for humans: Anatomical-morphological basis and parameter evaluation
Source: PLoS One. 2025 Jul 2;20(7):e0327156. doi: 10.1371/journal.pone.0327156 (PMC12306906; doi:10.1371/journal.pone.0327156)
Supplement: S2 Humeri — (DOCX) [file pone.0327156.s002.docx]

**humerus**

**Pre-adults, analysis of published data on humerus macro-parameters and cortical thickness**

The shape and size of the humerus are significantly dependent on age, Fig. H1 illustrates the age-changes in the period 0–5 years.


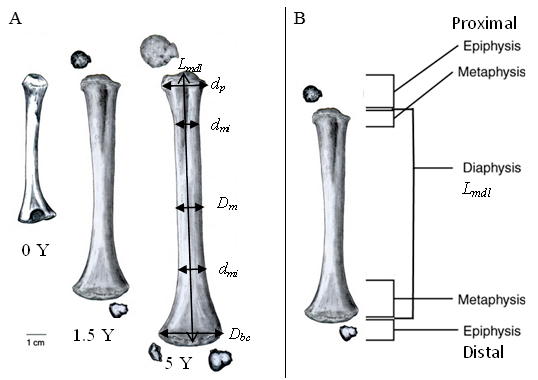


**Fig. H1.** Age-changes in humerus: A-humerus in natural proportion (Baker et al. 2005) for newborn, infant and children of about 5 years; epiphyseal centers of ossification are shown; B- diagram of the divisions of pre-adult humerus into diaphysis, metaphysis, and epiphysis; note that the diaphysis length (syn. maximal diaphysis length) includes the length of metaphysis (Buikstra and Ubelaker 1994; Meresh 1970). Letter designations are deciphered in the text.

Main measured parameters described in literature which were collected:

- Maximal diaphysis length *(L_mdl_)-* distance between the proximal and distal growth zone (epiphyseal lines), does not include the epiphysis ossified from separate centers (Fig. H1);
- Outside diameter (*D_m_*) in the mid-point of diaphysis (Fig. H1);
- Cortical thickness (Ct.Th) in the mid-point of diaphysis;
- Bicondylar diameter (*D_bc_*) in distal part of humerus (Fig. H1);

We have not found in published data on the values of the following parameters required in modeling:

- *Ct.Th* at diaphysis points (*i*) other than mid-point;
- Outside diameters (*d_mi_, d_p_*) at different diaphysis points (*i*) other than mid-point.

Values these parameters (*d_mi_, d_p_, Ct.Th* in different diaphysis points) were derived from the analysis of bone images (Normal pediatric bone X-ray; e.g., Baker et al. 2005; Cunningham et al. 2016; Scheuer and Black 2004; Gosman et al. 2013). Approaches to parameter assessment are described below.

**Table H1.** Published data on maximal diaphyseal length (*L_mdl_*), mm.

| Author | Age | N | M | SD |
| --- | --- | --- | --- | --- |
| Danforth et al. 2009 | 0 | 6 | 61.3 | 7.5 |
| Demidov et al. 1990* | 0 | n/a | 65.0 | 3.5 |
| Jeanty et al. 1983* | 0 | >100 | 66.0 | 3.0 |
| Medvedev et al. 1999* | 0 | n/a | 66 | 2.5 |
| Florence 2007 | 0.04 | 18 | 63.5 | 7.8 |
| Maresh 1970 | 0.1 | 128 | 71.8 | 3.6 |
| Florence 2007 | 0.2 | 40 | 72.4 | 4.5 |
| Maresh 1970 | 0.3 | 124 | 69.2 | 4.4 |
| Florence 2007 | 0.4 | 12 | 80.2 | 3.8 |
| Maresh 1970 | 0.5 | 145 | 80.6 | 4.8 |
| Florence 2007 | 0.7 | 6 | 75.0 | 5.2 |
| Maresh 1970 | 1.0 | 153 | 86.8 | 4.6 |
| Danforth et al. 2009 | 1.0 | 2 | 78 | 21.2 |
| Maresh 1970 | 1.5 | 152 | 88.4 | 5.0 |
| Florence 2007 | 1.9 | 4 | 97.7 | 6.8 |
| Maresh 1970 | 2.0 | 152 | 103.6 | 4.8 |
| Danforth et al. 2009 | 2.0 | 1 | 115 |  |
| Maresh 1970 | 2.5 | 154 | 105.5 | 5.2 |
| Maresh 1970 | 3.0 | 150 | 117.0 | 5.1 |
| Danforth et al. 2009 | 3.0 | 7 | 115.7 | 6.1 |
| Maresh 1970 | 3.5 | 151 | 118.8 | 5.4 |
| Maresh 1970 | 4.0 | 152 | 124.6 | 22.4 |
| Danforth et al. 2009 | 4.0 | 2 | 137.5 | 5 |
| Florence 2007 | 4.4 | 2 | 127.7 | 5.8 |
| Maresh 1970 | 4.5 | 149 | 130.0 | 5.5 |
| Maresh 1970 | 5.0 | 157 | 136.9 | 6.1 |
| Danforth et al. 2009 | 5.0 | 1 | 146 |  |
| Maresh 1970 | 5.5 | 147 | 139.0 | 5.9 |
| Maresh 1970 | 6.0 | 146 | 145.3 | 6.7 |
| Danforth et al. 2009 | 6.0 | 5.0 | 158.6 | 15.5 |
| Maresh 1970 | 6.5 | 153 | 147.5 | 6.7 |
| Maresh 1970 | 7.0 | 157 | 153.4 | 7.1 |
| Danforth et al. 2009 | 7.0 | 5 | 166 | 10.9 |
| Florence 2007 | 7.4 | 1 | 155.0 | 7.8 |
| Maresh 1970 | 7.5 | 159 | 160.9 | 7.7 |
| Maresh 1970 | 8.0 | 155 | 162.7 | 6.9 |
| Danforth et al. 2009 | 8.0 | 1.0 | 178 |  |
| Maresh 1970 | 8.5 | 154 | 154.5 | 9.6 |
| Maresh 1970 | 9.0 | 159 | 169.1 | 8.3 |
| Danforth et al. 2009 | 9.0 | 2.0 | 191 | 1.4 |
| Maresh 1970 | 9.5 | 161 | 169.8 | 7.4 |
| Maresh 1970 | 10.0 | 161 | 176.3 | 8.7 |
| Danforth et al. 2009 | 10.0 | 4.0 | 202.3 | 6.1 |
| Florence 2007 | 10.4 | 2 | 177.4 | 8.2 |
| Maresh 1970 | 10.5 | 151 | 182.6 | 9.0 |
| Maresh 1970 | 11.0 | 151 | 184.6 | 8.1 |
| Danforth et al. 2009 | 11.0 | 2.0 | 214 | 7.1 |
| Maresh 1970 | 11.5 | 151 | 190.0 | 9.6 |
| Continuation | | | | |
| Author | Age | N | M | SD |
| Maresh 1970 | 12.0 | 144 | 190.9 | 7.6 |
| Danforth et al. 2009 | 12.0 | 2.0 | 214.5 | 10.6 |
| Maresh 1970 m | 14.5 | 64 | 299.6 | 16.4 |
| Maresh 1970 m | 15.0 | 60 | 306.7 | 15.6 |
| Maresh 1970 m | 15.5 | 52 | 313.7 | 15.4 |
| Maresh 1970 f | 14.5 | 42 | 293.5 | 15.9 |
| Maresh 1970 f | 15.0 | 57 | 294.2 | 15.8 |
| Maresh 1970 f | 15.5 | 12 | 301.3 | 18.3 |

*- cited from Medvedev et al. 2009

**Table H2.** Published data on humeral outside diameter (*D_m_*) and cortical thickness (*Ct.Th*) in the mid-point of diaphysis, mm.

| Author | Age | N | *D_m_* | | Ct.Th | |
| --- | --- | --- | --- | --- | --- | --- |
|  |  |  | M | SD | M | SD |
| Svadkovsky 1961 | 0 | 10 | 5.4 | 0.3 | 1.63 | 0.3 |
| Florence 2007 | 0 | 10 | 5.3 | 0.8 | 1.6 | 0.2 |
| Florence 2007 | 0.2 | 17 | 5.6 | 0.7 | 1.7 | 0.3 |
| Florence 2007 | 0.4 | 9 | 6.4 | 0.9 | 1.8 | 0.3 |
| Florence 2007 | 0.7 | 5 | 8.6 | 1.1 | 2.1 | 0.4 |
| Svadkovsky 1961 | 2 | 20 | 11.6 | 0.43 | 2.0 | 0.3 |
| Florence 2007 | 2 |  | 9.9 | 2.6 | 2.1 | 0.5 |
| Svadkovsky 1961 | 4 | 5 | 12.7 | 0.52 | 2.2 | 0.4 |
| Florence 2007 | 4.5 | 3 | 12.1 | 0.42 | 2.9 | 0.5 |
| Svadkovsky 1961 | 6 | 5 | 13.6 | 0.49 | 2.4 | 0.4 |
| Florence 2007 | 7.5 | 1 | 12.4 |  | 3.0 |  |
| Svadkovsky 1961 | 8 | 5 | 14.7 | 0.30 | 2.9 | 0.12 |
| Svadkovsky 1961 | 10 | 5 | 15.3 | 0.40 | 3.1 | 0.3 |
| Florence 2007 | 10.5 | 2 | 17.8 | 1.0 | 3.1 | 0.3 |
| Svadkovsky 1961 | 12 | 6 | 16.9 | 0.55 | 3.3 | 0.3 |
| Svadkovsky 1961 | 14 | 6 | 17.6 | 0.55 | 3.5 | 0.6 |
| Svadkovsky 1961 | 16 | 6 | 19.7 | 1.07 | 4.0 | 0.5 |
| Svadkovsky 1961 | 18 | 6 | 21.3 | 1.71 | 4.9 | 0.4 |

**Table H3.** Averaged values of measured humeral-parameters used in modeling, mm.

| Age | *L_mdl_* | | Ct.Th in mid-point of diaphysis | | *D_m_* | | *D_bc_* | |
| --- | --- | --- | --- | --- | --- | --- | --- | --- |
|  | M | SD | M | SD | M | SD | M | SD |
| 0 | 67.2 | 6.3 | 1.7 | 0.3 | 5.7 | 0.7 | - | - |
| 1 | 79.9 | 10.3 | 2.1 | 0.4 | 8.6 | 1.1 | - | - |
| 5 | 135.9 | 5.8 | 2.7 | 0.5 | 12.9 | 0.46 | 45.6 | 3.1 |
| 10 | 181.7 | 7.9 | 3.1 | 0.3 | 16.6 | 0.7 | 54.0 | 4.9 |
| 15 m | 306.7 | 15.8 | 3.8 | 0.6 | 18.7 | 0.8 | 63.0 | 5.0 |
| 15 f | 296.3 | 16.7 | 3.8 | 0.6 | 18.7 | 0.8 | 63.0 | 5.0 |

**Table H4.** Published data on humeral bicondylar diameter (*D_bc_*), mm.

| Author | Ager | n | M | SD |
| --- | --- | --- | --- | --- |
| Zivicnjak et al. 2007 | 2 | 70 | 43.8 | 2.3 |
| Zivicnjak et al. 2007 | 3 | 115 | 45.9 | 2.5 |
| Zivicnjak et al. 2007 | 4 | 153 | 46.9 | 2.6 |
| Singh 2007 | 5 | 37 | 43.1 | 3 |
| Zivicnjak et al. 2007 | 5 | 218 | 48.6 | 3.1 |
| Singh 2007 | 6 | 37 | 43.2 | 2.5 |
| Zivicnjak et al. 2007 | 6 | 227 | 49.9 | 3.3 |
| Singh 2007 | 7 | 40 | 45.8 | 5 |
| Zivicnjak et al. 2007 | 7 | 368 | 51.6 | 3.2 |
| Singh 2007 | 8 | 40 | 45.9 | 2.7 |
| Zivicnjak et al. 2007 | 8 | 295 | 52.8 | 3.05 |
| Singh 2007 | 9 | 36 | 48.8 | 3.8 |
| Zivicnjak et al. 2007 | 9 | 358 | 55 | 3.5 |
| Singh 2007 | 10 | 36 | 51.1 | 6.2 |
| Zivicnjak et al. 2007 | 10 | 343 | 56.8 | 3.6 |
| Singh 2007 | 11 | 46 | 52.7 | 5.3 |
| Zivicnjak et al. 2007 | 11 | 433 | 58.9 | 3.9 |
| Singh 2007 | 12 | 70 | 56.4 | 14.2 |
| Zivicnjak et al. 2007 | 12 | 394 | 61.3 | 4.0 |
| Singh 2007 | 13 | 37 | 56.2 | 5.2 |
| Zivicnjak et al. 2007 | 13 | 406 | 62.2 | 3.6 |
| Singh 2007 | 14 | 33 | 57.1 | 4.6 |
| Zivicnjak et al. 2007 | 14 | 434 | 64.3 | 3.6 |
| Singh 2007 | 15 | 38 | 59.7 | 6.7 |
| Zivicnjak et al. 2007 | 15 | 457 | 65 | 3.3 |
| Singh 2007 | 16 | 45 | 62.1 | 4.3 |

In order to move from the D_m_ and Ct.Th at mid-point of diaphysis to the values at other points, we suggested that the relative change in these parameters with diaphysis length corresponds to that for femur (Appendix Femur). Locations of reference points in humeral diaphysis were also adopted the same as for the femur. The distance from the distal end to the reference points is evaluated in percentages of the maximum diaphysis length (L_mdl_): 15.6%; 32%; 50% (mid-point); 65%, 81%; 95–99 (proximal end; for humerus only).

Table H5 and H6 present the relative values (relative mid-point 50%) of humerus outside-diameters (*d_mi_*) and Ct.Th in reference points. Estimates are based on femur data of Gosman et al. 2013 (Table F7 and F8 in Appendix Femur) and image analysis. Table H7 and Table H8 present estimated values assumed for modeling.

**Table H5.** Relative values (relative mid-point 50%) of outside-diameters (*d_mi_*) in reference points of humerus (relative units).

| Age | 15.6% | 32% | **Mid-point 50%** | 65% | 81% | 95–99% proximal end |
| --- | --- | --- | --- | --- | --- | --- |
| 0 | 1.4 | 1.1 | 1 | 1.05 | 1.25 | 2.3 |
| 1 | 1.4 | 1.1 | 1 | 1.05 | 1.25 | 2.3 |
| 5 | 1.5 | 1.1 | 1 | 1.0 | 1.4 | 2.2 |
| 10 | 1.25 | 1.05 | 1 | 1.0 | 1.2 | 2.2 |
| 15 | 1.2 | 1.15 | 1 | 1.0 | 1 | 2.2 |

**Table H6.** Relative values (relative mid-point 50%) of diaphysis *Ct.Th_i_* in reference points of humerus (relative units).

| Age | 15.6% | 32% | **Mid-point 50%** | 65% | 81% |
| --- | --- | --- | --- | --- | --- |
| 0 | 0.4 | 0.6 | 1.0 | 0.7 | 0.5 |
| 1 | 0.4 | 0.6 | 1.0 | 0.7 | 0.5 |
| 5 | 0.6 | 0.8 | 1.0 | 1.3 | 0.7 |
| 10 | 0.5 | 0.9 | 1.0 | 1.0 | 1.0 |
| 15 | 0.4 | 0.9 | 1.0 | 1.1 | 0.8 |

**Table H7.** Calculated values of humerus outside diameters (*d_mi,_* mm) in reference points.

| Age | 15.6% | 32% | **Mid-point 50%** | 65% | 81% | 95–99% proximal end *(d_pe_)* |
| --- | --- | --- | --- | --- | --- | --- |
| 0 | 8.1 | 6.25 | 5.7 | 6.1 | 6.8 | 13.1 |
| 1 | 12.2 | 9.45 | 8.6 | 9.2 | 10.3 | 19.8 |
| 5 | 18.6 | 14.7 | 12.9 | 13.6 | 16.4 | 28.4 |
| 10 | 21.55 | 17.4 | 16.6 | 17.2 | 17.75 | 36.5 |
| 15 | 22 | 21.3 | 18.7 | 18.4 | 18.9 | 41.1 |

**Table H8.** Calculated values of humerus cortical thickness (*Ct.Th_i_* mm) in reference points.

| Age | 15.6% | 32% | **Mid-point 50%** | 65% | 81% |
| --- | --- | --- | --- | --- | --- |
| 0 | 0.65 | 1.05 | 1.70 | 1.21 | 0.81 |
| 1 | 0.80 | 1.3 | 2.10 | 1.50 | 1.00 |
| 5 | 1.49 | 2.21 | 2.70 | 3.45 | 1.80 |
| 10 | 1.6 | 2.9 | 3.1 | 3.2 | 3.1 |
| 15 | 1.58 | 3.43 | 3.80 | 4.08 | 3.06 |

Uncertainty values of *Ct.Th_i_* and *d_mi_* estimates were taken the same as for mid-point of diaphysis (in terms of CV)

**Humerus 0–1 Y, segmentation and estimation of model parameters**

Humerus has the shape of a complex tube; at the ends, the diameters are much larger than in the middle. Three BPSs were used for description of femur of newborn and 1-Y old (Figs. H1, H2), Table H9 summarizes the approaches to BPS parameter derivation:


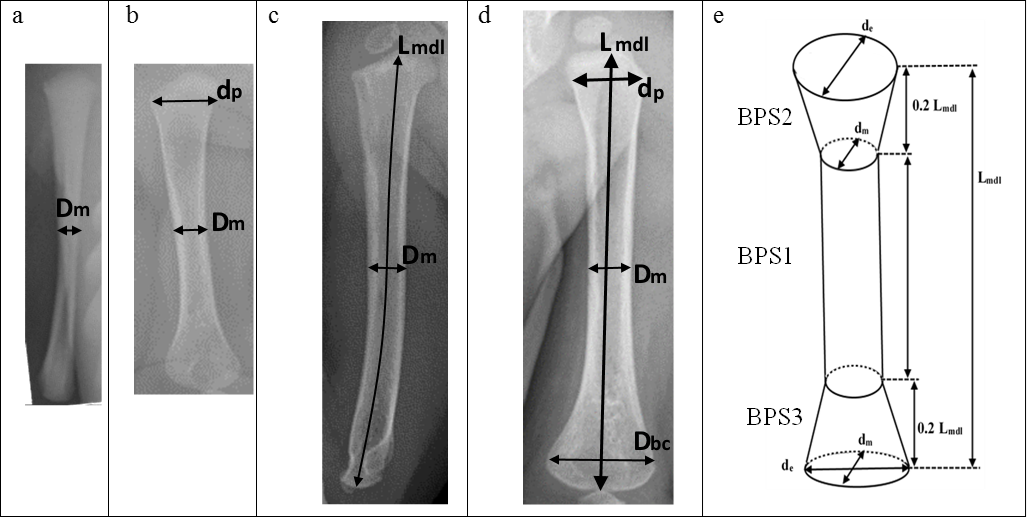


**Fig. H2.** Humeri, (a – d) radiograph (x-rays) images (Normal pediatric bone X-ray) : (a) 0-Y lateral view; (b) 0-Y anterior view; (с) 1-Y lateral view; (d) 1-Y anterior view; (e) stylized models (BPSs).

BPS 1 (shaft) was described by round cylinder of height h_m_ and diameter d_m_; cortical layer is located on the walls of the cylinder;

BPS 2 (proximal end) was described by the truncated cone of height *h_e_*; round base of diameter *d_m_* and lager round base of d_e_; cortical layer is located on the walls of the cylinder;

BPS 3 (distal end) was described by the truncated cone of height *h_e_*; round base of diameter *d_m_* and elliptical base of diameters *d_m_* and d*_e;_* cortical layer is located on the walls of the cylinder.

**Table H9.** BPS parameter assumed for humerus of 0–1 Y (mm).

| BPS | Parameter | Rationale | 0 Y | | 1 Y | |
| --- | --- | --- | --- | --- | --- | --- |
|  |  |  | M | SD | M | SD |
| #1 | *Ct.Th* | Three point average (32%, 50%, 65%)^a^ | 1.3 | 0.2 | 1.6 | 0.3 |
| #2 | *Ct.Th* | Two times thinner than at reference points 65% | 0.4 | 0.07 | 0.5 | 0.1 |
| #3 | *Ct.Th* | Two times thinner than at reference points 32% | 0.32 | 0.06 | 0.4 | 0.08 |
| #1 | *h_m_* | 60% maximal diaphysis length *L_mdl_* | 40.3 | 3.8 | 47.9 | 6.2 |
| #1, #2, #3 | *d_m_* | Three-point average (32%, 50%, 65%) a | 6.0 | 0.7 | 9.1 | 1.2 |
| #2, #3 | *h_e_* | 20% maximal diaphysis length *L_mdl_* | 13.4 | 1.3 | 16.0 | 2.1 |
| #2, #3 | *d_e_* | =*d_pe_* in point 95–99% ^b^ | 13.1 | 1.6 | 19.8 | 2.5 |

a- *d_m_* and Ct.Th values in reference-points are presented in the Table H7 and Table H8; b- data from Table H7

**Humerus 5-Y, segmentation and estimation of model parameters**

Three BPSs were used for description of 5-Y child (Fig. H3), Table H10 summarizes the approaches to BPS parameter derivation:

**Fig. H3.** Humeri of 5-Y child: a,b - radiograph (x-rays) images from (Normal pediatric bone X-ray) a- lateral view; b- anterior view; (с) stylized models (BPSs).

BPS 1 (shaft) was described by round cylinder of height h_m_ and diameter d_m_; cortical layer is located on the walls of the cylinder;

BPS 2 (proximal end) was described by the truncated cone of height *h_pe_*; round base of diameter *d_m_* and lager round base of d_pe_; cortical layer is located on the walls of the cylinder;

BPS 3 (distal end) was described by the truncated cone of height *h_de_*; round base of diameter *d_m_* and elliptical base of diameters *d_m_* and *D_bc_*; cortical layer is located on the walls of the cylinder.

**Table H10.** BPS parameter assumed for humerus of 5-Y (mm).

| BPS | Parameter | Rationale | 5 Y | |
| --- | --- | --- | --- | --- |
|  |  |  | M | SD |
| #1 | *Ct.Th* | Four point average (32%, 50%, 65%, 82%)^a^ | 2.5 | 0.5 |
| #2 | *Ct.Th* | Two times thinner than at reference points 65% | 0.9 | 0.16 |
| #3 | *Ct.Th* | Two times thinner than at reference points 32% | 0.75 | 0.14 |
| #1 | *h_m_* | 65% maximal diaphysis length *L_mdl_* | 88.3 | 3.8 |
| #1, #2, #3 | *d_m_* | Four point average (32%, 50%, 65%, 82%)^a^ | 14.7 | 0.5 |
| #2 | *h_pe_* | 15% maximal diaphysis length *L_mdl_* | 20.4 | 0.9 |
| #3 | *h_de_* | 20% maximal diaphysis length *L_mdl_* | 27.2 | 1.2 |
| #2, | *d_pe_* | point 95–99% b | 32.4 | 1.2 |
| #3 | *d_de_* | =*D_bc_* | 45.6 | 3.1 |

1. *d_m_* and Ct.Th values in reference-points are presented in the Table H7 and Table H8; b- data from Table H7

**Humerus 10-Y, segmentation and estimation of model parameters**

Two segments were highlighted to describe the proximal and distal ends of humerus diaphysis (Fig. F5). The middle part of diaphysis is not modeled, since it does not contain the AM. Table H11 summarizes the approaches to parameter derivation.

**Fig. H4.** Humeri of 10-Y children: (a – c) radiograph (x-rays) images (Normal pediatric bone X-ray): (a) anterior view; (b) proximal part anterior view; (c) distal part lateral view; (d) stylized models (BPSs).

BPS 1 (proximal end) was described by the truncated cone of height *h_e_*; round base of diameter *d_m_* and lager round base of d_pe_; cortical layer is located on the walls of the cylinder;

BPS 2 (distal end) was described by the truncated cone of height *h_e_*; round base of diameter *d_m_* and elliptical base of diameters *d_m_* and *D_bc;_* cortical layer is located on the walls of the cylinder.

**Table H11.** BPS parameter assumed for humerus of 10 Y (mm).

| BPS | Parameter | Rationale | 10 Y | |
| --- | --- | --- | --- | --- |
|  |  |  | M | SD |
| #1 | *Ct.Th* | equal to that for adults | 1.1 | 0.18 |
| #2 | *Ct.Th* | Two times thinner than at reference points 15% | 0.78 | 0.08 |
| #1, #2 | *d_m_* | Four point average (32%, 50%, 65%, 82%)^a^ | 17.7 | 0.7 |
| #1, #2 | *h_e_* | 15% maximal diaphysis length *L_mdl_* | 27.3 | 1.2 |
| #1 | *d_pe_* | point 95–99% b | 38.9 | 1.6 |
| #2 | *d_de_* | =*D_bc_* | 54.0 | 4.9 |

a- *d_m_* and Ct.Th values in reference-points are presented in the Table H7 and Table H8; b- data from Table H7.

**Adults, and 15-y analysis of published data on humerus macro-parameters and cortical thickness**

One segment was highlighted to describe the proximal end of humerus diaphysis for adults and 15 Y (Fig. H5).


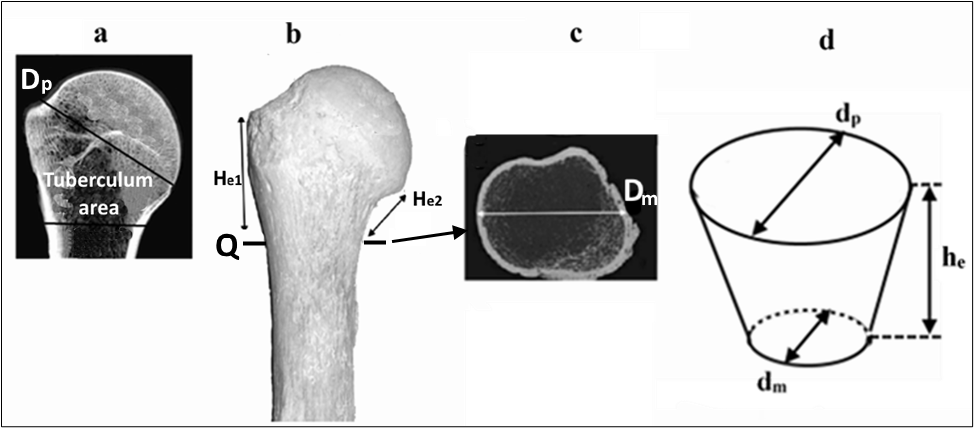


**Fig. H5.** Humeri of adults: a - radiograph (x-rays) slice, anterior view on proximal humerus; (b) proximal humeri tuberculum area measurements; (c) radiograph slice of tuberculum area of humeri (d) stylized models (BPS), Q – cross-section through the surgical neck.

Measured values:

*Dm* – diameter of the humeri at surgical neck

*Dp* – the distance from the most proximal point of the greater tuberosity to the most inferior point on the margin of the articular surface on the humeral head

*He1* – the distance between the most proximal point of the greater tuberosity of the humeral head and surgical neck;

*He2* –the distance between the most inferior point on the margin of the articular surface on the humeral head and surgical neck

BPS 1 (proximal end) was described by the truncated cone of height *h_e_*; round base of diameter *d_m =_Dm* and lager round base of *d_p_=Dp*; cortical layer is located on the walls of the cylinder;

**Table H12.** Published data of proximal humeri cortical thickness for adults, mm

| Author | Age | n | Head | | Neck | |
| --- | --- | --- | --- | --- | --- | --- |
|  |  |  | M | SD | M | SD |
| Sprecher et al. 2015 | 58.7±29.0 | 6 | 0.33 | - | 2.2 | 0.5 |
| Helfen et al. 2017 | 18-44 | 8 | - | - | 1.85 | 0.28 |
|  | 45-64 | 10 | - | - | 1.54 | 0.21 |
| **Average values assumed for BPS (CV%)** | | | **Ct.Th.(head) =0.33**  **Ct.Th.(neck)=1.86 (18)** | | | |

Cortical thickness of tuberculum area of humerus was calculated as mean value between Ct.Th.(head) and Ct.Th.(neck**)** =1.1 (CV=18%)

**Table H13.** Published data of humeri tuberculum area for adult male, mm

| Author | Age | Sex | n | *D_p_* | SD | *D_m_* | SD | *H_e1_* | SD | *H_e2_* | SD |  |
| --- | --- | --- | --- | --- | --- | --- | --- | --- | --- | --- | --- | --- |
| Atamtürk et al. 2010 | 20-79 | m | 36 | 59.4 | - | - | - | - | - | - | - |  |
| Shehri et al. 2015 | 20-62 | m | 216 | 55 | 3.0 | - | - | - | - | - | - |  |
| Iyem et al. 2017 | 18-65 | m | 101 | - | - | 21.5 | 3.2 | - | - | - | - |  |
| Mohanty et al. 2012 | Adults | M | 100 | - | - | - | - | 35 | 4 | - | - |  |
| Kabakci et al. 2017 | Adults | m+f | 31 | - | - | 28.5 | 2.9 | 32.9 | 3.4 | - | - |  |
| Akman et al. 2006 | 30-60 | m | 120 | - | - | - | - | 34.4 | - | - | - |  |
| Ali and Elbaky 2016 | 20-60 | M | 75 | - | - | - | - | - | - | 21.2 | 2.8 |  |
| **Average values assumed for BPS (CV%)** | | | | ***D_p_=56 (5); D_m_=25.1 (15;) H_e1_=34.1 (11); H_e2_=21.2 (13)*** | | | | | | | | |

**Table H14.** Published data of humeri tuberculum area for adult female, mm

| Author | Age | Sex | n | *Dp* | SD | *Dm* | SD | *He1* | SD | *He2* | SD |  |
| --- | --- | --- | --- | --- | --- | --- | --- | --- | --- | --- | --- | --- |
| Atamtürk et al. 2010 | 20-79 | f | 39 | 51.3 | 3.2 | - | - | - | - | - | - |  |
| Iyem et al. 2017 | 26-58 | f | 94 | - | - | 19.2 | 2.3 | - | - | - | - |  |
| Mohanty et al. 2012 | Adults | f | 100 | - | - | - | - | 33.4 | 1.4 | - | - |  |
| Kabakci et al. 2017 | Adults | m+f | 31 | - | - | 28.5 | 2.9 | 32.9 | 3.4 | - | - |  |
| Srimani et al. 2017 | Adults | f | 30 | - | - | - | - | 27.8 | - | - | - |  |
| Ali and Elbaky 2016 | 20-60 | f | 75 | - | - | - | - | - | - | 18.4 | 2.2 |  |
| **Average values assumed for BPS (CV%)** | | | | ***D_p_=51.3 (6); D_m_=23.9 (11) H_e1_=31.4 (8); H_e2_=18.4 (12)*** | | | | | | | | |

*h_e_* was calculated as average value between *H_e1_* and *H_e2_*

For male *h_e_*=28 (12)

For female *h_e_*=24.9 (10)

**Analysis of published data on humerus microstructures**

**Table H15.** Published data on microstructure of individual proximal humerus for pre-adults.

| Author | Individual code^*^ | Age | BV/TV, r.u. | Tb.Th, mm |
| --- | --- | --- | --- | --- |
| Gosman et al. 2011 | - | 0–0.5 | 0.3462 | 0.075 |
| Gosman et al. 2011 | - | 0–0.5 | 0.3231 | 0.085 |
| Gosman et al. 2011 | - | 0–0.5 | 0.2885 | 0.08 |
| Gosman et al. 2011 | - | 0–0.5 | 0.3231 | 0.071 |
| Gosman et al. 2011 | - | 0–0.5 | 0.2692 | 0.069 |
| Gosman et al. 2011 | - | 0–0.5 | 0.2731 | 0.075 |
| Gosman et al. 2011 | - | 0–0.5 | 0.1654 | 0.065 |
| Gosman et al. 2011 | - | 0–0.5 | 0.1615 | 0.065 |
| Ryan et al. 2017 | 172 | 0.5 | 0.387 | 0.208 |
| Ryan et al. 2017 | 202 | 0.5 | 0.251 | 0.172 |
| Ryan et al. 2017 | 85 | 0.63 | 0.294 | 0.161 |
| Ryan et al. 2017 | 57 | 0.75 | 0.191 | 0.157 |
| Ryan et al. 2017 | 140 | 0.75 | 0.206 | 0.14 |
| Ryan et al. 2017 | 142 | 0.75 | 0.249 | 0.171 |
| Ryan et al. 2017 | 167 | 0.75 | 0.195 | 0.166 |
| Ryan et al. 2017 | 173 | 0.75 | 0.318 | 0.235 |
| Ryan et al. 2017 | 247 | 0.75 | 0.149 | 0.118 |
| Ryan et al. 2017 | 58 | 1 | 0.213 | 0.21 |
| Ryan et al. 2017 | 117 | 1.5 | 0.297 | 0.211 |
| Continuation | | | | |
| Author | Individual code^*^ | Age | BV/TV, r.u. | Tb.Th, mm |
| Ryan et al. 2017 | 131 | 1.5 | 0.209 | 0.173 |
| Ryan et al. 2017 | 135 | 1.5 | 0.176 | 0.166 |
| Ryan et al. 2017 | 162 | 1.5 | 0.115 | 0.15 |
| Ryan et al. 2017 | 221 | 1.5 | 0.114 | 0.195 |
| Ryan et al. 2017 | 88 | 2 | 0.279 | 0.234 |
| Ryan et al. 2017 | 65 | 2.3 | 0.17 | 0.17 |
| Ryan et al. 2017 | 153 | 2.5 | 0.232 | 0.195 |
| Ryan et al. 2017 | 25 | 3 | 0.307 | 0.231 |
| Ryan et al. 2017 | 76 | 3 | 0.306 | 0.203 |
| Ryan et al. 2017 | 161 | 3 | 0.123 | 0.217 |
| Ryan et al. 2017 | 183 | 3.5 | 0.3 | 0.189 |
| Ryan et al. 2017 | 115 | 5 | 0.143 | 0.163 |
| Ryan et al. 2017 | 29 | 6.5 | 0.307 | 0.215 |
| Ryan et al. 2017 | 260 | 8 | 0.267 | 0.201 |
| Ryan et al. 2017 | 113 | 9 | 0.238 | 0.207 |
| Ryan et al. 2017 | 89 | 10.5 | 0.216 | 0.267 |
| Pafundi 2009 | - | 18 | 0.1552 | - |

^*^ original burial number from Ryan et al. 2017;

**Table H16**. Measured data on trabecular space, mm (Ryan et al. 2017; total n=27).

| Age | n | Average | SD | Min | Max |
| --- | --- | --- | --- | --- | --- |
| 0.0–0.6 | 3 | 0.360 | 0.041 | 0.325 | 0.406 |
| 0.75–1.75 | 12 | 0.577 | 0.270 | 0.416 | 1.34 |
| 2.0–10.5 | 12 | 0.579 | 0.188 | 0.386 | 1.05 |

**Table H17.** Published data on microstructure of proximal humerus for adults, mm

| Author | n | Age | BV/TV | SD | Tb.Th, | SD | Tb.Sp | SD |
| --- | --- | --- | --- | --- | --- | --- | --- | --- |
| Head | | | | | | | | |
| Scherf et al. 2013 | 8 | Adults | 0.15 | 0.01 | 0.184 | 0.03 | 0.955 | 0.09 |
| Barvencik et al. 2010 | 15 | 30 (20–40) | 0.13 | 0.01 | 0.114 | 0.003 | 0.702 | 0.001 |
| Barvencik et al. 2010 | 15 | 50 (40–60) | 0.13 | 0.01 | 0.112 | 0.003 | 0.802 | 0.001 |
| Yakacki et al. 2010 | 1 | 36 | - | - | 0.128 | - | 0.48 | - |
| Tuberculum area | | | | | | | | |
| Barvencik et al. 2010 | 15 | 20-40 | 0.06 | 0.02 | 0.094 | - | 2.23 | - |
|  | 15 | 40-60 | 0.06 | 0.02 | 0.095 | - | 2.55 | - |

The variability of the microstructure parameters for the tuberculum area (in terms of CV) was assumed to be the same as the variability in the humeral head (Table 17)

**Table H18.** Ratio BV/TV, Tb.Th and Tb.Sp assumed for humerus in SPSD-model

| Age | BV/TV r.u.  (min–max) | SD | Tb.Th, mm  (min–max) | SD, mm | Tb.Sp, mm  (min–max) | SD, mm |
| --- | --- | --- | --- | --- | --- | --- |
| 0 | 0.280  (0.162–0.387) | 0.069 | 0.102  (0.065–0.208) | 0.052 | 0.360  (0.325–0.529) | 0.041 |
| 1 | 0.220  (0.114–0.318) | 0.066 | 0.174  (0.118–0.267) | 0.033 | 0.580  (0.416–0.813) | 0.270 |
| 5 | 0.220  (0.114–0.318) | 0.066 | 0.208  (0.118–0.267) | 0.028 | 0.580  (0.386–1.05) | 0.188 |
| 10 | 0.220  (0.114–0.318) | 0.066 | 0.208  (0.118–0.267) | 0.028 | 0.580  (0.386–1.05) | 0.188 |
| 15 | 0.220  (0.114–0.318) | 0.066 | 0.208  (0.118–0.267) | 0.028 | 0.580  (0.386–1.05) | 0.188 |
| Adults | 0.06  (1-13) | 0.02 | 0.1  (0.7-0.13) | 0.018 | 2.37  (1.4-3.3) | 0.590 |

**Reference for humerus**

Akman D, Karakab P, Bozkir. The Morphometric Measurements of Humerus Segments, Turk J Med Sci. 2006; 36:81–85.

Ali DM, Elbaky F. Sex identification and reconstruction of length of humerus from its fragments: An Egyptian study. Egyptian Journal of Forensic Sciences. 2016; 6(2):48–55.

Atamtürk D, Akçal A, Duyar I, Mas N. Sex estimation from the radiographic measurements of the humerus. Eurasian J. Anthropol. 2010; 1:99–108.

Baker B, Dupras TL, Tocheri MW, Wheeler SM. The osteology of infants and children. Texas A and M University Press. 2005.

[Barvencik F](https://www.ncbi.nlm.nih.gov/pubmed/?term=Barvencik%20F%5BAuthor%5D&cauthor=true&cauthor_uid=19630001), [Gebauer M](https://www.ncbi.nlm.nih.gov/pubmed/?term=Gebauer%20M%5BAuthor%5D&cauthor=true&cauthor_uid=19630001), [Beil FT](https://www.ncbi.nlm.nih.gov/pubmed/?term=Beil%20FT%5BAuthor%5D&cauthor=true&cauthor_uid=19630001), [Vettorazzi E](https://www.ncbi.nlm.nih.gov/pubmed/?term=Vettorazzi%20E%5BAuthor%5D&cauthor=true&cauthor_uid=19630001), [Mumme M](https://www.ncbi.nlm.nih.gov/pubmed/?term=Mumme%20M%5BAuthor%5D&cauthor=true&cauthor_uid=19630001), [Rupprecht M](https://www.ncbi.nlm.nih.gov/pubmed/?term=Rupprecht%20M%5BAuthor%5D&cauthor=true&cauthor_uid=19630001), [Pogoda P](https://www.ncbi.nlm.nih.gov/pubmed/?term=Pogoda%20P%5BAuthor%5D&cauthor=true&cauthor_uid=19630001), [Wegscheider K](https://www.ncbi.nlm.nih.gov/pubmed/?term=Wegscheider%20K%5BAuthor%5D&cauthor=true&cauthor_uid=19630001), [Rueger JM](https://www.ncbi.nlm.nih.gov/pubmed/?term=Rueger%20JM%5BAuthor%5D&cauthor=true&cauthor_uid=19630001), [Pueschel K](https://www.ncbi.nlm.nih.gov/pubmed/?term=Pueschel%20K%5BAuthor%5D&cauthor=true&cauthor_uid=19630001), [Amling M](https://www.ncbi.nlm.nih.gov/pubmed/?term=Amling%20M%5BAuthor%5D&cauthor=true&cauthor_uid=19630001). Age- and sex-related changes of humeral head microarchitecture: histomorphometric analysis of 60 human specimens. Journal of Orthopaedic Research. 2010; 28(1): 18–26.

Buikstra JE, Ubelaker D. Standards for data collection from human skeletal remains. Fayetteville, Arkansas: Arkansas archeological survey research series. 1994; 44.

Cunningham C, Scheuer L, Black S. Developmental Juvenile Osteology. Second Edition. Elsevier Academic Press. 2016.

Danforth ME, Wrobel GD, Armstrong CW, Swanson D. Juvenile age estimation using diaphyseal long bone lengths among ancient Maya populations. Latin American Antiquity. 2017; 20(1): 3–13.

Florence JL. Linear and cortical bone dimensions as indicators of health status in subadults from the Milwaukee County Poor Farm Cemetery. M.A., University of Colorado at Denver. 2007.

Gosman JH, Ketcham RA. Patterns in ontogeny of human trabecular bone from SunWatch Village in the Prehistoric Ohio Valley: general features of microarchitectural change. Am J Phys Anthropol. 2009 Mar;138(3):318–32. doi:10.1002/ajpa.20931. PubMed PMID: 18785633.

[Gosman JH](https://www.ncbi.nlm.nih.gov/pubmed/?term=Gosman%20JH%5BAuthor%5D&cauthor=true&cauthor_uid=22101688), [Stout SD](https://www.ncbi.nlm.nih.gov/pubmed/?term=Stout%20SD%5BAuthor%5D&cauthor=true&cauthor_uid=22101688), [Larsen CS](https://www.ncbi.nlm.nih.gov/pubmed/?term=Larsen%20CS%5BAuthor%5D&cauthor=true&cauthor_uid=22101688). Skeletal biology over the life span: a view from the surfaces. [Am J Phys Anthropol.](https://www.ncbi.nlm.nih.gov/pubmed/22101688) 2011;146 Suppl 53:86–98. doi: 10.1002/ajpa.21612.

Helfen T, Sprecher CM, Eberli U, Gueorguiev B, Müller PE, Richards RG, Schmidutz F. High-Resolution Tomography-Based Quantification of Cortical Porosity and Cortical Thickness at the Surgical Neck of the Humerus During Aging. Calcif Tissue Int. 2017; 101(3):271–279.

Iyem C, Serbest S, Inal M, Burulday V, Kaya A, Kultur T, Tiftikci U A morphometric evaluation of the humeral component in shoulder arthroplasty. Biomedical Research. 2017; 28(6): 2666–2672.

Jeanty P. Fetal limb biometry. Radiology. 1983 May;147(2):601-2. doi: 10.1148/radiology.147.2.6836145. PMID: 6836145.

Kabakci AAD, Buyukmumcu M, Yilmaz MT, Cicekcibasi AE, Akin D, Cihan E. An osteometric study on humerus bones. Int. J. Morphol. 2017; 35(1):219-226.

Maresh MM. Measurements from roentgenograms. In: Human Growth and Development (R.W. McCammon, Ed.) Springfield, IL: Charles C. Thomas. 1970; 157–200.

Medvedev MV Ed. Ultrasonic Fetometry: Reference Tables and Nomograms Ed. 8th, rev. Moscow: Real time Publisher. 2009; 19–24 (in Russian).

Mohanty S, Sahu G, Das S. Estimation of length of humerus from its fragmentary portions, J Forensic Leg Med. 2012; 19(6):316–320.

Normal pediatric bone X-ray, available in: <https://bonexray.com/>; <http://bones.getthediagnosis.org/>; <http://bonepit.com/>;

Pafundi D. Image-based skeletal tissues and electron dosimetry models for the ICRP reference pediatric age series. A dissertation presented to the graduate schools of the University of Florida in partial fulfillment of the requirements for the degree of doctor of the philosophy. University of Florida. 2009.

[Ryan](https://www.cambridge.org/core/search?filters%5BauthorTerms%5D=Timothy%20M.%20Ryan&eventCode=SE-AU) TM, [Raichlen](https://www.cambridge.org/core/search?filters%5BauthorTerms%5D=David%20A.%20Raichlen&eventCode=SE-AU) DA, [Gosman](https://www.cambridge.org/core/search?filters%5BauthorTerms%5D=James%20H.%20Gosman&eventCode=SE-AU) JH. Structural and Mechanical Changes in Trabecular Bone during Early Development in the Human Femur and Humerus. Chapter 12. In: [Building Bones: Bone Formation and Development in Anthropology](https://www.cambridge.org/core/books/building-bones-bone-formation-and-development-in-anthropology/FAA70C0A5554F14B613C13A5F3A1A891). Cambridge University Press 2017; 281–302. <https://doi.org/10.1017/9781316388907.013>

Scherf H, Harvati K, Hublin JJ. A comparison of proximal humeral cancellous bone of great apes and humans. J Hum Evol. 2013. 65(1):29–38.

Scheuer L, Black S. The juvenile Skeleton. Elsevier Academic Press London WC1X 8RR, UK 2004.

Shehri FA, Soliman KE Determination of sex from radiographic measurements of the humerus by discriminant function analysis in Saudi population, Qassim region, KSA. Forensic Sci Int. 2015; 253:138. e1–6.

Singh SP, Malhotra P, Sidhu LS & Singh Prit Pal. Skeletal Frame Size of Spitian Children, Journal of Human Ecology. 2007; 21:3, 227-230, DOI: 10.1080/09709274.2007.11905977

Sprecher CM, Schmidutz F, Helfen T, Richards RG, Blauth M, Milz S. Histomorphometric Assessment of Cancellous and Cortical Bone Material Distribution in the Proximal Humerus of Normal and Osteoporotic Individuals. [Medicine (Baltimore).](https://www.ncbi.nlm.nih.gov/pubmed/26705200) 2015; 94(51): e2043.

Srimani P, Datta M, Saha A, Mazumdar S. Prediction of Total Length of Humerus from its Fragments in West Bengal Population. International Journal of Anatomy, Radiology and Surgery. 2017 Jan; 6(1): 1-6.

Svadovsky VS. Age-related bone remodeling. Moscow, 1961. (In Russian)

Yakacki CM, Poukalova M, Guldberg RE. The effect of the trabecular microstructure on the pullout strength of suture anchors. J Biomech. 2010. 43(10):1953–1959.

Zivicnjak M, Smolej Narancić N, Szirovicza L, et al. Gender-specific growth patterns of transversal body dimensions in Croatian children and youth (2 to 18 years of age). Coll Antropol. 2008;32(2):419-431.
