# Supplementary material for: Stochastic parametric skeletal dosimetry model for humans: Anatomical-morphological basis and parameter evaluation
Source: PLoS One. 2025 Jul 2;20(7):e0327156. doi: 10.1371/journal.pone.0327156 (PMC12306906; doi:10.1371/journal.pone.0327156)
Supplement: S3 Pelvis — (DOCX) [file pone.0327156.s003.docx]

**Pelvis (Os Coxae)**

**Pre-adults of 0, 1, 5, and 10 years**

In children, the pelvic bone consists of three separate bones: ilium, ischium, and pubis (Fig. P1; Scheuer and Black 2004; Cunningham et al. 2016). They are connected to each other by cartilaginous tissue, forming a single structure. The bones are modeled separately up to the moment of fusion in the area of the acetabulum (about age 12 years), that is, the BPS parameters for individual bones are determined for reference ages 0, 1, 5, and 10 years. For 15-year-old men and women, segmentation and BPS-parameters are done as for an adult.


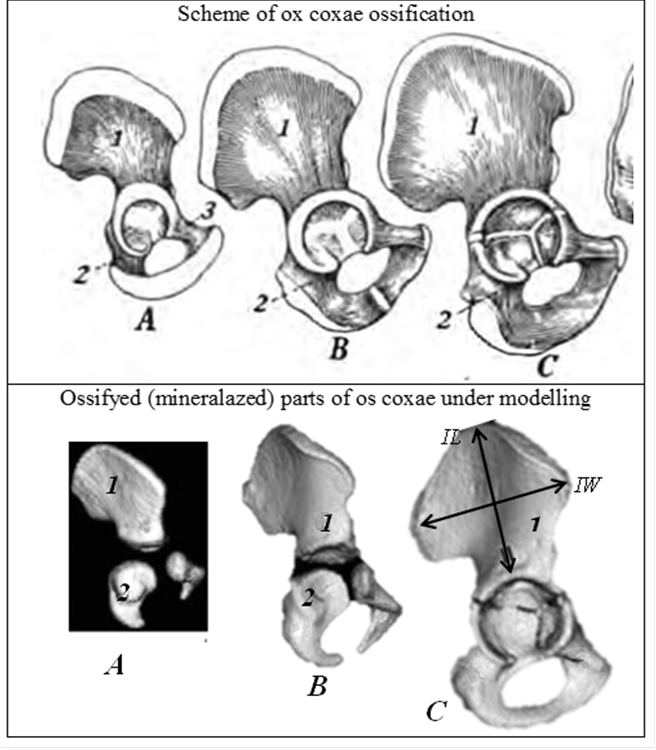


**Fig. P1.** Lateral view of os coxae of approximate ages. (A) 1–4 months; (B) 4–5 years; and (C) 8–10 years; 1-ilium; 2-ischium; 3-pubis. Upper panel after Quain's anatomy; lower panel - according to Scheuer and Black (2004); Cunningham et al. (2016); *IW*- ilium weidth; *IL*- ilium length.

Three bones are variously embedded in the formation of the acetabulum, which determines the features of bone segmentation for modeling (Fig. P1). Pubis forms the upper and anterior one-fifth of acetabulum, the ischium forms the floor of the acetabular fossa and the lower and posterior two-fifths, while the ilium forms the remaining upper two-fifths of the articular surface.

**Ilium**

**Analysis of published data on ilium macro-parameters and cortical thickness**

Typically, morphometric studies evaluate the following parameters important for modeling. (Tables P1–P4): *IW*-Ilium width: maximum distance between the anterior to the posterior superior iliac spines; *IL* - Ilium length: maximum distance between the middle point of the iliac crest and the convexity of the acetabular extremity; *S_il_* - total area of ilium; *C.W_i_*- ilium core width or ilium thickness, it was estimated by biopsy in the area of iliac crest; *Ct.Th* – thickness of cortical layer on gluteal side, pelvic side and average.

**Table P1.** Published data on Ilium length and Ilium width, mm.

| Author | Age | n | *IL* | | *IW* | |
| --- | --- | --- | --- | --- | --- | --- |
|  |  |  | Mean | SD | Mean | SD |
| Blake 2011 | 1 mo | 192* | 32.64 | 5.01 | - | - |
| Cunningham et al. 2016 | 0–6 mo | 12 | 33.15 | - | 35.3 | - |
| Corron 2017 | 0–12 mo | 22 | 37.12 | 6.69 | 42.33 | 8.06 |
| Cunningham et al. 2016 | 10–18 mo | 8 | 47.55 | - | 53.9 | - |
| Corron 2017 | 1–2 y. | 14 | 57.97 | 5.54 | 65.12 | 5.89 |
| Corron 2017 | 5–6 y | 19 | 81.11 | 6.15 | 86.24 | 8.86 |
| Corron 2017 | 10–11 y. | 24 | 108.56 | 4.85 | 119.73 | 6.51 |

***-** number of measured bones including right and left ilium from the same person

Extensive studies by Rissech et al. (2003, 2005, 2007) regarding morphometric measurements of pelvis should be noted. Unfortunately, these publications contain averaged parameter values; averaging was performed in increments of 5 years, which makes their use difficult. (The data of Rissech et al. for ilium are not given here). Similarly, data from Yusof et al. 2013 on estimates of ilium areas, which were averaged over 5-year ranges, were not used to estimate model parameters, except for data for newborns (Table P2).

**Table P2.** Published data of iliac surface area S_il_ (mm^2^).

| Author | Age | n | S_il_, | SD |
| --- | --- | --- | --- | --- |
| Yusof et al. 2013 | Neonate | 5 | 739.5 | 27.8 |
| Corron et al. 2017 | 0–12 mo | 22 | 1166.3 | 416.6 |
| Corron et al. 2017 | 1–2 y. | 14 | 2719.6 | 426.2 |
| Corron et al. 2017 | 5–6 y | 19 | 4981.3 | 708.8 |
| Corron et al. 2017 | 10–11 y. | 24 | 9189.6 | 954.5 |

**Table P3.** Published data on ilium core width (mm).

| Author | Age | n | C.Wi | SD |
| --- | --- | --- | --- | --- |
| Schnitzler et al. 2009 | 0.1 | 5 | 4.0 | 0.9 |
| Schnitzler et al. 2009 | 0.5–1.5 | 6 | 5.0 | 0.9 |
| Schnitzler et al. 2009 | 4–7 | 4 | 7.88 | 1.03 |
| Parfitt et al. 2000 | 5 | 2 | 7.0 | 2.0 |
| Schnitzler et al. 2009 | 9–11 | 9 | 7.83 | 1.52 |
| Parfitt et al. 2000 | 9–11 | 8 | 8.19 | 1.75 |

Сortical thickness has been most extensively studied for newborns (Cunningham and Black 2009). Cunningham and Black (2009) divided the bone area into 23 squares and studied the cortical thickness on the gluteal side and pelvic side. Two areas on the gluteal side can be distinguished: areas with a large Ct.Th of about 1 mm (14 squares in the center) and a small thickness of about 0.2–0.3 mm (9 squares located on the edge of the bone, in the peripheral part). On the pelvic side, the variability of the cortical thickness is not so high. We averaged the data over two regions and obtained the values of the following parameters (Table P4): region 1 (in the center of the bone) - gluteal side 1, pelvic side 1; region 2 (peripheral part) —gluteal side 2, pelvic side 2. We used these values estimates of BPS- parameters (described below). For other ages (1-Y, 5-Y, 10-Y), the results of measurements of bone biopsies in the iliac crest (Table P3), i.e. in the peripheral part, were used.

**Table P4.** Published data on ilium cortical thickness (mm).

| Author | Age | n | Ct.Th | SD |
| --- | --- | --- | --- | --- |
| Cunningham et al. 2009 | 0 | 28 | 1.20 (gluteal side 1)  0.47 (pelvic side 1)  0.24 (gluteal side 2)  0.21 (pelvic side 2) | 0.398  0.228  0.074  0.032 |
| Parfitt et al. 2000 | 1 | -^a b^ | 0.4 | - |
| Schnitzler et al. 2009 | 1 | -^a b^ | 0.4 | - |
| Glorieux et al. 2000 | 1.5–6.9 | 10^b^ | 0.70 | 0.28 |
| Parfitt et al. 2000 | 4–6 | 2 ^b^ | 1.0 | - |
| Schnitzler et al. 2009 | 5 | 7 ^b^ | 0.757 | 0.172 |
| Glorieux et al. 2000 | 7.0–10.9 | 10 ^b^ | 0.97 | 0.37 |
| Parfitt et al. 2000 | 8.0–11.0 | 6 ^b^ | 0.92 | 0.29 |
| Schnitzler et al. 2009 | 8.0–11.0 | 10 ^b^ | 0.69 | 0.22 |

a- calculated according to equation describing age dependencies; b - average cortical thickness in the iliac crest area (in a bone biopsy)

**Table P5.** Averaged values of measured data on ilium in mm (*S_il_* in mm^2^).

| Age | IL | SD | IW | SD | S_il_ | SD | Ct.Th ^b^ | SD | C.Wi | SD |
| --- | --- | --- | --- | --- | --- | --- | --- | --- | --- | --- |
| 0 | - ^a^ | - ^a^ | - ^a^ | - ^a^ | 952.9 | 95 | 1.20 ^b^  0.47 ^c^  0.23 ^d^ | 0.40  0.23  0.06 | 4.0 | 0.9 |
| 1 | 57.97 | 5.54 | 65.12 | 5.89 | 2719.6 | 426.2 | 0.40 | 0.12 | 5.0 | 0.9 |
| 5 | 81.11 | 6.15 | 86.24 | 8.86 | 4981.3 | 708.8 | 0.82 | 0.16 | 7.88 | 1.03 |
| 10 | 102.5 | 7.54 | 110.6 | 7.3 | 9189.6 | 954.5 | 0.86 | 0.15 | 8.0 | 1.75 |

a- not used in modeling; b- gluteal side in iliac central area; c- pelvic side in iliac central area; d- average for gluteal and pelvic sides for peripheral area.

**Ilium 0-Y, segmentation and estimation of model parameters**

The bone is assumed to be flat with the same core width (C.Wi). Two box-segments of 30 mm × 30 mm × C.Wi with different Ct.Th were taken. The cortical layer covers the upper and lower sides. The external size of the segments is the same, C.Wi = 4±0.9 mm

BPS1 describes 60% of the total ilium area (S_il_):

Ct.Th of gluteal side=1.2±0. 39 mm; Ct.Th of pelvic side =0.47±0.22 mm

BPS2 describes 40% of the total ilium area (S_il_):

Ct.Th is the same for gluteal and pelvic sides = 0.23±0.058 mm

Total area of ilium S_il_= 953 ±90 mm^2^

**Ilium 1-Y, segmentation and estimation of model parameters**

Three BPSs were used for description of 1-Y ilium because the acetabulum part begins to form (Fig. P2):

BPS1, BPS2 describe flat part of ilium by boxes of the same external size (30mm×30mm×C.Wi) with different Ct.Th; they describe (in equal parts) 85% of the total ilium area, the cortical layer covers the upper and lower sides.

BPS3 (acetabular part) was described by a truncated cone with elliptical bases (Fig. P2), the cortical layer covers the walls of the cone. It is assumed that the acetabular part accounts for 15% of the total ilium area.

Total ilium area *S_il_*= 2719.56±426.21 mm^2^


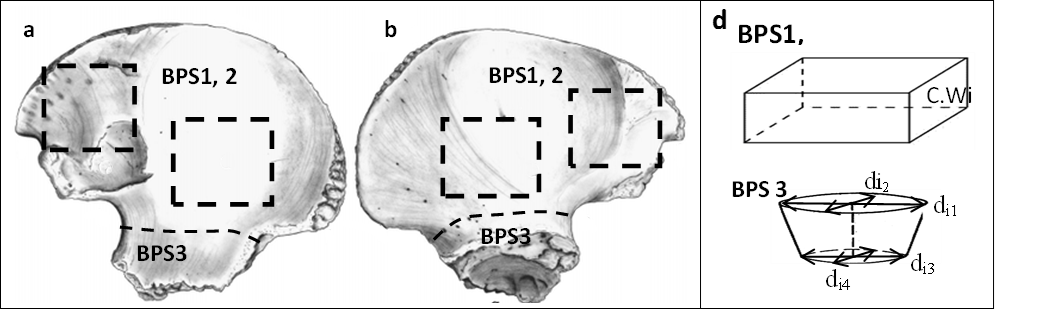


**Fig. P2.** Ilium of children; a- gluteal side; b- pelvic side; d- stylized models (BPSs).

**Table P6.** BPS parameter assumed for ilium of 1-Y (mm).

| BPS | Parameter | Rationale | M | SD |
| --- | --- | --- | --- | --- |
| #1,#2 | *C.Wi* | Measured data | 5.0 | 0.9 |
| #1 | *Ct.Th* gl. side | Same as in 0-Y | 1.2 | 0.39 |
| #1 | *Ct.Th* pel. side | Same as in 0-Y | 0.47 | 0.22 |
| #2 | *Ct.Th* | Measurements for iliac crest | 0.4 | 0.12 |
| #3 | *d_i1_* | = 0.4 × *IW* | 26.05 | 2.35 |
| #3 | *d_i2_* | Double ilium core width *C.Wi* | 10 | 3.0 |
| #3 | *d_i3_* | Image analysis (Cunningham et al. 2016,  Figure 11-13) | 23.6 | 5.3 |
| #3 | *d_i4_* |  | 17.8 | 7.2 |
| #3 | *h_i_* | = 0.25 × *IL* | 14.5 | 1.4 |
| #3 | *Ct.Th* | Equal to that for BPS2 | 0.4 | 0.12 |

Note: gl. side - gluteal side; pel. side – pelvic side

**Ilium 5-Y, segmentation and estimation of model parameters**

Three BPSs were used for description of 5-Y ilium; they are similar to those of 1-Y child (Fig. P2). Table P7 describes the BPS-parameters.

BPS1, BPS2 describe flat part of ilium by boxes of the same external size (30mm×30mm×C.Wi) with different Ct.Th; they describe (in equal parts) 85% of the total ilium area, the cortical layer covers the upper and lower sides.

BPS3 (acetabular part) was described by a truncated cone with elliptical bases (Fig. P2), the cortical layer covers the walls of the cone. It is assumed that the acetabular part accounts for 15% of the total ilium area.

Total ilium area *S_il_* = 4981±709 mm^2^

**Table P7.** BPS parameter assumed for ilium of 5-Y, mm

| BPS | Parameter | Rationale | M | SD |
| --- | --- | --- | --- | --- |
| #1,#2 | *C.Wi* | Measured data | 7.88 | 1.03 |
| #1 | *Ct.Th* gl. side | 2 times thicker than on pelvic side | 1.64 | 0.54 |
| #1 | *Ct.Th* pel. side | Measurements for iliac crest | 0.82 | 0.16 |
| #2 | *Ct.Th* | Measurements for iliac crest | 0.82 | 0.16 |
| #3 | *d_i1_* | = 0.4 × *IW* | 34.5 | 3.54 |
| #3 | *d_i2_* | Double ilium *C.Wi* | 15.75 | 4.7 |
| #3 | *d_i3_* | Image analysis (Cunningham et al. 2016  Figure 11-13) | 33.55 | 10.1 |
| #3 | *d_i4_* |  | 27.1 | 8.1 |
| #3 | *h_i_* | = 0.25 × *IL* | 20.28 | 1.54 |
| #3 | *Ct.Th* | Equal to that for BPS2 | 0.82 | 0.16 |

Note: gl. side - gluteal side; pel. side – pelvic side.

**Ilium 10-Y, segmentation and estimation of model parameters**

Three BPSs were used for description of 10-Y ilium (two boxes and truncated cone with elliptical bases); they are similar to those of 1-Y and 5-Y child (Fig. P2). Table P8 describes the BPS-parameters. Total ilium area *S_il_* = 9189.6±954.5mm^2^

**Table P8.** BPS parameter assumed for ilium of 5-Y, mm

| BPS | Parameter | Rationale | M | SD |
| --- | --- | --- | --- | --- |
| #1,#2 | *C.Wi* | Measured data | 8.01 | 1.75 |
| #1 | *Ct.Th* gl. side | 2 times thicker than on pelvic side | 1.71 | 0.57 |
| #1 | *Ct.Th* pel. side | Measurements for iliac crest | 0.86 | 0.15 |
| #2 | *Ct.Th* | Measurements for iliac crest | 0.86 | 0.15 |
| #3 | *d_i1_* | = 0.4 × *IW* | 44.23 | 2.9 |
| #3 | *d_i2_* | = 2.5×*C.Wi* | 20.03 | 4.38 |
| #3 | *d_i3_* | Image analysis (Cunningham et al. 2016,  Figure 11-13) | 37.1 | 11.13 |
| #3 | *d_i4_* |  | 27.8 | 8.34 |
| #3 | *h_i_* | = 0.25 × *IL* | 25.6 | 1.9 |
| #3 | *Ct.Th* | Equal to that for BPS2 | 0.86 | 0.15 |

Note: gl. side - gluteal side; pel. side – pelvic side.

**Pubis**

**Analysis of published data on pubis macro-parameters and cortical thickness for pre-adults**

Typically, morphometric studies evaluate the following parameters important for modeling. (Tables P9–P11): *PL*-pubic length, maximum length between acetabular articular end and symphyseal surface; *PW*- pubic width, minimum width of the pubic body from the pubic symphyseal face, usually near the base of the pubic symphysis, to the obturator foramen; *L-I*PR- length of ischial-pubis ramus, we did not find the results of its direct measurements, the value is calculated based on measured lengths of pubis and ischium and ischial-pubic angle.

**Table P9**. Pubic length and width, mm

| Author | Age | n | PL | SD | n | PW | SD |
| --- | --- | --- | --- | --- | --- | --- | --- |
| Blake 2011 | 1 mo | 144 | 14.82 | 1.99 | 89 | 5.02 | 0.81 |
| Scheuer and Black 2009 | 0 | 10 | 16.6 | 1.4 | - | - | - |
| Blake 2011 | 1 | 21 | 24.16 | 2.85 | 19 | 7.67 | 0.85 |
| Blake 2011 | 5 | 26 | 36.26 | 5.88 | 24 | 13.12 | 1.50 |
| Blake 2011 | 10 | 22 | 48.73 | 2.97 | 22 | 16.01 | 1.52 |
| Blake 2011 | 15 | 2 | 62.12 | 5.19 | 2 | 16.68 | 1.24 |

**Table P10**. Parameters of acetabular surface of pubis, mm

| Age | Maximal (vertical) diameter | Minimal (horizontal) diameter |
| --- | --- | --- |
| 6 month | 15.5 | 10.9 |
| 4 year | 21.8 | 16.8 |
| 6 year | 22.7 | 18.2 |

Note: Pictures with measuring scales were measured by us in Cunningham et al. 2016, Figure 11-13.

**Table P11.** Parameters of acetabular surface of pubis according to Rissech (2007), mm

| Age range | n | Vertical diameter | | Horizontal diameter | |
| --- | --- | --- | --- | --- | --- |
|  |  | M | DS | M | DS |
| 0–4 | 15 | 17.25 | 4.93 | 11.95 | 2.92 |
| 5–9 | 15 | 24.85 | 2.41 | 17.7 | 1.74 |
| 10–12 | 12 | 28.1 | 2.385 | 20.9 | 2.14 |
| 13–16 | 5 | 35 | 4.18 | 23 | 1.22 |

**Pubis 0-Y, segmentation and estimation of model parameters**

Pubis of newborn is described by cylindrical BPS (Fig. P3) of height *h_p_*= Pubic Length (*PL*); and diameter of round base *d_p_* = Pubic Width (*PW*).

PL=15.71±2.0 mm

PW=5.02±0.81 mm.

The cortical layer covers the wall of the cylinder. Cortical thickness corresponds to the averaged value for pelvic side of ilium (averaging over the entire pelvic surface of the ilium):

Ct.Th= 0.37±0.033 mm.

**Fig. P3.** Segmentation of newborn pubis; a- pelvic side; b- gluteal side; stylized model (BPS).

**Pubis 1-Y, segmentation and estimation of model parameters**

Bone is modeled by two BPSs (Fig. P4, Table P12):

BPS1 acetabular part - truncated cone (wide part to the acetabulum) of height *h_p_*_1_; with elliptical base of diameters *d_p1_, d_p2_,* and round base of diameter *d_p3_,*

BPS2 pubic ramus superior- round-base cylinder of height *h_p_*_2_ and of diameter *d_p4_*

The cortical layer covers the wall of the cylinder and the wall of the truncated cone.

**Fig. P4.** Segmentation of pubis of 1-year old.

**Table P12.** BPS parameter assumed for pubis of 1-Y (mm).

| BPS | Parameter | Rationale | M | SD |
| --- | --- | --- | --- | --- |
| #1 | *dp1* | Based on Cunningham et al. 2016, Figure 11-13 | 15.5 | 5.3 |
| #1 | *dp2* |  | 10.9 | 7.2 |
| #1 | *dp3* | =PW | 7.67 | 0.85 |
| #1 | *hp1* | = 0.2 of PL | 4.83 | 0.72 |
| #2 | *hp2* | =0.8 of PL | 19.33 | 2.90 |
| #2 | *dp4* | = PW | 7.67 | 0.85 |
| #1, #2 | *Ct.Th* | Equal to average value for iliac crest | 0.4 | 0.12 |

**Pubis 5-Y, segmentation and estimation of model parameters**

Pubis is modeled by three BPSs (Fig. P5, Table P13):

**Fig. P5.** Segmentation of pubis for children of 5- and 10-years old.

BPS1 acetabular part - truncated cone (wide part to the acetabulum) of height *h_p_*_1_; with two elliptical bases of diameters *d_p1_, d_p2_, d_p3_, d_p4_*

BPS2 pubic-ramus-superior- cylinder of height *h_p_*_2_ with elliptical base of diameters *d_p5_, d_p6_*

BPS2 pubic-ramus-inferior - a small cylinder with round base of diameter *d_7_* and of height *h*_3_ that is 1/3 part of total ischial-pubic-ramus length *L-IPR* (1/3 is pubis part; 1/3 is ischial part; 1/3 is not mineralized cartilage between them). All parameters are equal to those for ischium-ramus-inferior

The cortical layer covers the wall of the cylinders and the wall of the truncated cone.

**Table P13.** BPS parameter assumed for pubis of 5-Y, mm

| BPS | Parameter | Rationale | M | SD |
| --- | --- | --- | --- | --- |
| #1 | *d_p1_* | Based on Cunningham et al. 2016, Figure 11-13 | 22.25 | 4.45 |
| #1 | *d_p2_* |  | 17.5 | 3.5 |
| #1 | *d_p3_* | *PW* | 13.12 | 1.5 |
| #1 | *d_p4_* | =1/2 *d_p2_* | 8.75 | 1.75 |
| #1 | *h_p1_* | 0.2 of *PL* | 7.25 | 1.09 |
| #2 | *h_p2_* | 0.8 *PL* | 29.00 | 4.35 |
| #2 | *d_p5_* | *PW* | 13.12 | 1.50 |
| #2 | *d_p6_* | 1/2 *d_p2_* | 8.75 | 1.75 |
| #3 | *h_p3_* | 1/3 of *L-IPR_1_*^a^ | 18.8 | 2.82 ^b^ |
| #3 | *d_p7_* | = *d_p6_* | 8.75 | 1.75 |
| All | *Ct.Th* | The same as for adult | 0.5 | 0.15 |

a - the length of the ischial-pubic-ramus (L-IPR) is calculated from the assumption that it is the side of the triangle, where the 2 other sides are pubic length (PL) and ischium length (IsL). Knowing the ischial-pubic angle (*α*), the L-IPR is calculated by the formula √ (PL^2^ + PL ^2^ - 2 PL IsL *cos(α)*). Thus, L-IPR = √ (46.2^2^ + 36.3^2^ - 2 46.2 36.3 cos (112.6º)) = 68.9 (mm). Subtract ½ PW and ½ of the width of ischium (*l_is_*) from this value and obtain L-IPR1 = 68.9-6.56-5.85 = 56.85 (mm). In turn, IPR1 conditionally consists of 3 parts: 1/3 - pubic ramus inferior; 1/3 (middle) - the cartilaginous part; 1/3 - ischial ramus inferior. The angle (α = 112.6º) was taken from Sachdeva et al. 2019 (data for adults of both sexes).

b - SD corresponds to CV = 15%

**Pubis 10-Y, segmentation and estimation of model parameters**

The approaches to bone segmentation and determination of BPS parameters are the same as for 5-Y child. Pubis is modeled by three BPSs: truncated cone and two cylinders (Fig. P5, Table P14). The cortical layer covers the wall of the truncated cone and the wall of cylinders.

**Table P14.** BPS parameter assumed for pubis of 10-Y (mm)

| BPS | Parameter | Rationale | Assumed value, mm | SD, mm |
| --- | --- | --- | --- | --- |
| #1 | *d_p1_* | Based on Cunningham et al. 2016, Figure 11-13 | 28.1 | 2.39 |
| #1 | *d_p2_* |  | 20.9 | 2.14 |
| #1 | *d_p3_* | *PW* | 16.01 | 1.52 |
| #1 | *d_p4_* | =1/2 *d_p2_* | 10.45 | 1.07 |
| #1 | *h_p1_* | 0.2 of *PL* | 9.75 | 1.46 |
| #2 | *h_p2_* | 0.8 *PL* | 38.98 | 5.85 |
| #2 | *d_p5_* | *PW* | 16.01 | 1.52 |
| #2 | *d_p6_* | 1/2 *d_p2_* | 10.45 | 1.07 |
| #3 | *h3* | 0.4×*L-IPR_1_*^a^ | 31.82 | 4.77 ^b^ |
| #3 | *d_p_7* | = *d_p6_* | 10.45 | 1.07 |
| All | Ct.Th | The same as for adult | 0.5 | 0.15 |

a- similar to Table P13: IPR = √(PL^2^ + PL ^2^ − 2 PL IsL cos α) =√(63.1^2^ + 48.7 ^2^ − 2 63.1 48.7 cos(112.6))=93.4 (mm); L-IPR_1_ =93.4-8-5.9= 79.6 (mm). At the age of 10, the cartilaginous part of IPR is largely replaced by the bone, both from the side of ischium and from the side of pubis. Thus, *h_3_* is assumed to be 0.4 of L-IPR1.

b- SD corresponds with CV=15%

**Ischium**

**Analysis of published data on ischium macro-parameters and cortical thickness**

The main measured parameters (Fig. P6, Tables P15 and P14): *IsL* - Ischium length: Maximum distance from the acetabular point (acetabular articular end) to the ischiatic tuberosity (ramus base); *Vd*- Vertical diameter of ischium acetabular surface: Distance between the acetabular point and the end of the posterior horn of the lunate surface. *Hd*- Horizontal diameter of ischium acetabular surface: Distance between the anteroinferior and posterosuperior angle of the ischium.

**Fig. P6.** 1-Ischium length; 2- Vertical diameter; 3- Horizontal diameter (based on Maclean et al. 2014, Blake 2011, Rissech et al. 2003).

**Table P15**. Published data on ischium length (*IsL*), mm

| Author | n | Age | Age range | IsL | SD |
| --- | --- | --- | --- | --- | --- |
| Molleson and Cox 1993 | 10 | 0 | - | 18.5 | 2.0 |
| Blake 2011 | 178 | 0.08 | 0–1 mo | 18.01 | 2.93 |
| Blake 2011 | 22 | 1 | - | 30.46 | 3.25 |
| Rissech et al. 2003 | 18 | 2 | 0–4 | 38.25 | 7.44 |
| Blake 2011 | 26 | 5 | 4–6 | 46.23 | 5.37 |
| Rissech et al. 2003 | 19 | 7 | 5–9 | 54.56 | 6.67 |
| Blake 2011 | 22 | 10 | 10–11 | 63.12 | 6.44 |
| Rissech et al. 2003 | 19 | 12 | 10–14 | 64.82 | 8.25 |
| Blake 2011 | 2 | 15 | 14–16 | 76.63 | 4.99 |
| Rissech et al. 2003 | 39 | 17 | 15–19 | 83.74 | 5.92 |

Molleson and Cox (1993) provide data not only on the length but also on the width of the ischium for the newborn *IsW* = 12 (11.5–13.5) mm, since the newborn os ischium looks like a “flattened oval” (Fig. P6).

**Table P16**. Published data on Ischium vertical and horizontal diameters (Rissech et al. 2003), mm

| Age | Age range | n (*Vd*) | *Vd* | SD | n (*Hd*) | *Hd* | SD |
| --- | --- | --- | --- | --- | --- | --- | --- |
| 2 | 0–4 | 18 | 23.5 | 3.66 | 17 | 25.85 | 5.64 |
| 7 | 5–9 | 19 | 32.44 | 4.24 | 18 | 37.86 | 4.57 |
| 11 | 10–12 | 12 | 36.5 | 2.32 | 12 | 45.06 | 4.58 |
| 15 | 13–16 | 6 | 44.5 | 4.09 | 5 | 56.8 | 6.38 |

**Ischium 0-Y, segmentation and estimation of model parameters**

Ischium is modeled by a flattened cylinder with an elliptical base of diameters *d_1_* = *IsL* = 18.2±2 mm; *d_2_* = IsW = 12±1.5 mm and a height corresponding to the ischium thickness, which is taken to be equal to the pubis width at this age *h* = 5.02 ± 0.81 mm. The cortical layer covers the base of the cylinder; its thickness corresponds to the average Ct.Th value over the pelvic side of ilium = 0.37 ± 0.033 mm.

**Ischium 1-Y, segmentation and estimation of model parameters**

The acetabulum begins to form, a slight extension of the ischial ramus towards the pubic bone is observed (not modeled) (Fig. P7, Table P17). Ischium is modeled by two BPSs:

BPS1 acetabular (upper) part is modeled by an isosceles triangular prism, where *h_is1_* - height; *w_is_* - thickness (bone thickness from posterior view) or the base of the triangle; *l_is1_* - side length of the triangle (bone length in the horizontal plane);

BPS2 tuberosity (lower) part - box with parameters *w_is2_* - bone thickness, *h_is2_* - height (in the vertical plane); *l_is2_* is the length in the horizontal plane.

The cortical layer covers the rectangular sides of the triangular prism (BPS1) and the sides of BPS2 excluding top and bottom sides.

| 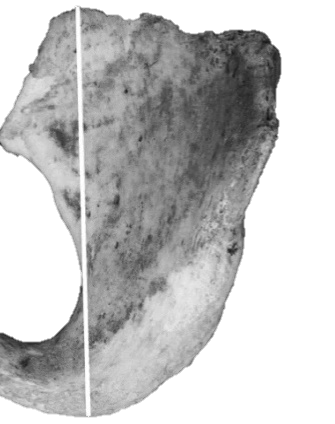  **BPS 2**  **BPS 1**  **Ramus (Tuberosity part)**  **Acetabular part** |   h_is1_  **BPS 1**  **BPS 2** |
| --- | --- |

**Fig. P7.** Segmentation of pubis for children of 1-Y; letter designations are deciphered in the text.

**Table P17.** BPS parameter assumed for ischium of 1-Y (mm).

| BPS | Parameter | Rationale | M | SD ^a^ |
| --- | --- | --- | --- | --- |
| #1 | *h_is1_* | Shape of the acetabular surface is close to the square^b^, where the diagonal (*d*) is the average value of *V_d_* and *H_d_*; then the side *h_is1_*= d/√2 = 24.7/1.14 | 17.5 | 2.63 |
| #1 | *l_is1_* | = *h_is1_* | 17.5 | 2.63 |
| #1 | *w_is1_* | Corresponds to the thickness of ilium in the acetabulum part | 17.8 | 5.3 |
| #2 | *h_is2_* | *IsL-h_is1_*=30.46-17.5 | 13.0 | 1.95 |
| #2 | *l_is2_* | =2/3 *l_is1_* | 11.7 | 1.76 |
| #2 | *w_is2_* | =2/3 *w_is1_* | 11.9 | 1.79 |
| All | *Ct.Th* | Average Ct.Th for iliac crest (and for pubis) | 0.4 | 0.12 |

a- For the calculated values, CV=15% was used to calculate SD;

b- Cunningham et al. 2016; Chapter 11 p. 381 “By year 1, the superior border of the ischium is square …”

**Ischium 5-Y, segmentation and estimation of model parameters**

The acetabulum continues to form, the ischial ramus grows towards the pubis. In general, the bone is modeled by 3 BPSs: two BPSs are similar to the age of 1-Y (triangular prism and box), plus an additional cylindrical BPS-ischial-ramus-inferior, which subsequently (at age 12–15) connects (fuses) with pubic ramus inferior (Fig. P8; Table P18).

BPS1 acetabular (upper) part is modeled by an isosceles triangular prism, where *h_is1_* - height; *w_is_* - thickness (bone thickness from posterior view) or the base of the triangle; *l_is1_* - side length of the triangle (bone length in the horizontal plane);

BPS2 tuberosity (lower) part - box with parameters *w_is2_* - bone thickness, *h_is2_* - height (in the vertical plane); *l_is2_* is the length in the horizontal plane.

BPS3 ischial ramus inferior - cylinder of height *h* and diameter of round base *d* (parameters are equal to those for pubic-ramus-inferior).

The cortical layer covers the rectangular sides of the triangular prism (BPS1); the sides of BPS2 excluding top and bottom; and the wall of cylinder (BPS3).

**Fig. P8.** Segmentation of pubis for children of 5 and 10 years old.

**Table P18.** BPS parameter assumed for ischium of 5-Y, mm

| BPS | Parameter | Rationale | M | SD ^a^ |
| --- | --- | --- | --- | --- |
| #1 | *h_is1_* | Shape of the acetabular surface is close to the square^b^, where the diagonal (*d*) is the average value of *V_d_* and *H_d_*; then the side *h_is1_* = d/√2= 29.9 /1.141 (mm) | 21.15 | 3.17 |
| #1 | *l_is1_* | =his1 | 21.15 | 3.17 |
| #1 | *w_is1_* | Corresponds to the thickness of ilium in the acetabulum part | 27.1 | 4.07 |
| #2 | *h_is2_* | *IsL*-*h_is_1*=46.23mm-21.15mm | 25.08 | 3.76 |
| #2 | *l_is2_* | =2/3 *l_is1_* | 14.10 | 2.12 |
| #2 | *w_is2_* | =2/3 *w_is1_* | 18.07 | 2.71 |
| #3 | *h* | Equal to those for pubic-ramus-inferior | 18.8 | 2.82 |
| #3 | *d* | Eequal to those for pubic-ramus-inferior | 8.75 | 1.75 |
| All | *Ct.Th* | The same as for adult | 0.5 | 0.15 |

a- For the calculated values, CV=15% was used to calculate SD

**Ischium 10-Y, segmentation and estimation of model parameters**

The approaches to bone segmentation and determination of BPS parameters are the same as for 5-Y child. Ischium is modeled by three BPSs: triangular prism, box, and cylinder (Fig. P8, Table P19). The cortical layer covers the rectangular sides of the triangular prism (BPS1); the sides of box BPS2 excluding top and bottom; and the wall of cylinder (BPS3).

BPS1 acetabular (upper) part is modeled by an isosceles triangular prism, where *h_is1_* - height; *w_is_* - thickness (bone thickness from posterior view) or the base of the triangle; *l_is1_* - side length of the triangle (bone length in the horizontal plane);

BPS2 tuberosity (lower) part - box with parameters *w_is2_* - bone thickness, *h_is2_* - height (in the vertical plane); *l_is2_* is the length in the horizontal plane.

BPS3 ischial ramus inferior - cylinder of height *h* and diameter of round base *d* (parameters are equal to those for pubic-ramus-inferior).

**Table P19.** BPS parameter assumed for ischium of 10-Y, mm

| BPS | Parameter | Rationale | M | SD ^a^ |
| --- | --- | --- | --- | --- |
| #1 | *h_is1_* | Shape of the acetabular surface is close to the square, where the diagonal (*d*) is the average value of *V_d_* and *H_d_*; then the side *h_is1_* = d/√2= 40.8 /1.141 | 28.8 | 4.32 |
| #1 | *l_is1_* | =*h_is1_* | 28.8 | 4.32 |
| #1 | *w_is1_* | Corresponds to the thickness of ilium in the acetabulum part | 27.8 | 4.17 |
| #2 | *h_is2_* | *IsL*−*h_is1_*=63.12−28.8 | 34.32 | 5.15 |
| #2 | *l_is2_* | =2/3 *l_is1_* | 19.2 | 2.88 |
| #2 | *w_is2_* | =2/3 *w_is1_* | 18.53 | 2.78 |
| #3 | *H* | equal to those for pubic-ramus-inferior | 31.82 | 4.77 |
| #3 | *D* | equal to those for pubic-ramus-inferior | 10.45 | 1.07 |
| All | *Ct.Th* | The same as for adult | 0.5 | 0.15 |

a- For the calculated values, CV=15% was used to calculate SD

**Adults and 15 –Y**

[Pelvic](https://en.wikipedia.org/wiki/Hip_bone) bone (os coxae) formed by the fusion of ilium, ischium, and pubis. It was assumed that the size of the pelvic bones in 15-year-olds is the same as in adults. Since the largest number of measurements were performed for adults, these measurements formed the basis for modeling pelvic bones. The following parts were considered in ilium: iliac crest, iliac ala, and [dorsal](http://boneandspine.com/glossary/dorsal/) segment (inner side of the segment is the sacropelvic surface). Ishium consists of body and ramus. Pubis consists of body, [superior](http://boneandspine.com/glossary/anterior/) ramus and [inferior](http://boneandspine.com/glossary/inferior/) ramus. Lower end of ilium is fused with body of ischium and body of pubis at the acetabulum. For modeling, the following segments were allocated (Fig. P9).

1. *Iliac crest* was described by box of length *l=30* mm, height *h_c_* and width w_c_; cortical layer covers the box top and two lateral sides; total length of iliac crest *l_c_* was derived from measured data;
2. *Iliac dorsal segment* was described by box of height *h_id_* length *l_id_* and width *w_id_* (bone thickness); *l_id_=w_id_*= 30 mm; total surface of *iliac* *dorsal segment* was taken form measured data; cortical layer is located on the top and bottom (inner and outer surface of ilium).
3. *Iliac ala* (wing) was approximated by box of height *h_ia ,_* length *l_ia_* and width (bone thickness) *w_ia_* ; *l_ia_=w_ia_*= 30 mm; total surface of *iliac ala* was taken form measured data; cortical layer is located on the top and bottom (inner and outer surface of ilium).

**Fig P9.** Pelvic bones: a-medial view; b-suggital section; c-lateral view; d- stylized models (BPS) described pelvic segments. (1) Iliac crest. (2) Iliac dorsal segment. (3) Iliac ala. (4) Pubic ramus superior (upper part): l_pu_ was derived from the acetabulum diameter (*d_a_* ) and the length of pubic bone (*PL,* greatest distance from the central point of the acetabulum to the symphyseal surface): *l_pu_ =PL- ½d_a_*; (5) Pubic ramus superior (lower part); (6) *Pubic* ramus inferior; (7) Ramus of *ischium:* *h_is_* was derived from ischial length (*IL,* greatest distance between the central point of acetabulum and the farthest point on inferior aspect of ischial tuberosity) and diameter of acetabulum (*d*_a_): *h_is_=IL- ½d_a_ ;* (8) acetabulum.

1. Upper part of *pubic ramus superior* was described by box of length *l_pu_* (maximum distance between the outer border of acetabulum and the articular surface of pubic symphysis), height *h_pu_* and width *w_pu_*; it was assumed that cortical layer covers the three lateral sides and one (symphysial side);.
2. Lower part of *pubic* *ramus* *superior,* that also forms a symphysis surface, was described by box of height *h_pl_* (total height of symphysis minus height of ramus superior *h_pu_*), length *l_pl_* and width *w_pl_*; it was assumed that cortical layer does not cover the two sides adjacent to the neighboring sites (upper part of pubic ramus superior and pubic ramus inferior).
3. *Pubic ramus inferior* was described by truncated elliptic cone of height *h*_pi_; base of cone adjacent to pubic ramus superior has maximal and minimal axes (diameters) *d1_pi_, d2_pi_* ; base adjacent to ischium - *d3_pi_, d4_pi_* ; cortical layer covers the lateral surface of cone.
4. Ramus of *ischium* was described by elliptic cylinder of maximal and minimal axes (diameter) *d1_is_, d2_is_* and height *h_is_* (distance between the outer border of acetabulum and the farthest point on inferior aspect of ischial tuberosity); cortical layer covers the lateral surface and the bottom.
5. *Acetabulum* was described by two nested cylinders (tube-like shape). The inner cylinder is empty. The diameter of inner cylinder *d_a_* corresponds to mean acetabular diameter; *th_aw_* - thickness of acetabulum wall, which consists of outer and inner cortical layers and internal trabecular layer; *h_a_*-height (depth) of acetabulum, *th_b_*- thickness of cortical layer in the bottom.

**Analysis of published data on pelvic macro-parameters and cortical thickness**

**Table P20.** Iliac dorsal segment area for combined samples of male and female, mm^2^

| Author | Age | n | *S_id_*,*mm^2^ | SD | *h_id_* | SD |
| --- | --- | --- | --- | --- | --- | --- |
| SUSHPU collection | Adults | 8 | 2150 | 240 | 19 | 3 |
| **Assumed for BPS (CV%)** | |  | **215 (12)** | | **19 (16)** | |

*Inner surface adjacent to sacrum; *h_id_-*bone thickness (the shortest distance between pelvic and gluteal sides of bone)

**Table P21.** Ilium ala parameters for combined set of male and female (mean± STD, mm), published data and results of measurements.

| Author | Age (range) | n | *h_i_** | *SD* | *S_i_* mm^2 **^ | SD |
| --- | --- | --- | --- | --- | --- | --- |
| Hernigou J. 2014 | 50 (20-80) | 24 | 9.5 | 3 | - |  |
| SUSHPU collection | Adults | 11 | - | - | 3360 | 400 |
| **Assumed for BPS (CV%)** | |  | **9.5(30)** | | **3360 (12)** | |

* - the data on different points of ilium ala were averaged by us;

**S_i_ – surface area of ilium ala as it indicated in Fig P9; rough estimate obtained using graph paper, the value does not include an area in the center of the ilium (about 25% of surface area), which consists only of the cortical bone (i.e. does not contain the spongiosa)

**Table P22.** Iliac crest measurements for combined samples of male and female, mm.

| Author | Age | n | *w_c_* | SD | *h_c_* | SD |
| --- | --- | --- | --- | --- | --- | --- |
| Sönmez et al. 2013 | 50-95 | 28 | 12 | 3 | - | - |
| Khamanarong et al. 2005 | 26-86 | 126 | 13 | 2 | - | - |
| Hernigou et al. 2014 | 20-80 | 24 | 13 | 1 | - | - |
| SUSHPU collection | Adults | 11 | - | - | 11 | 1 |
| **Assumed for BPS (CV%)** | | | **13 (15)** | | **11 (9)** | |

Length of iliac crest for adults is taken according Sachdeva et al. 2014ab:

**For male (n=80) *l_c_*=161±15 mm; for females (n=10) *l_c_*=148±12 mm**.

*h_c_-* was measured as distance between margin of iliac bone and the point from which the thickness of the ilium ceases to change significantly

Thickness of ilium for adult was taken as average value of ilium thickness measured by Hernigou et al. 2014 (age 20-80 y; n=24) at different points; ***h_i_*=9.5±3 mm**.

**Table P23**. Pubic-ramus-superior (upper part) macro-parameters for **adult male** (mean± STD, mm).

| Author | Age (range) | n | *PL* | *h_pu_* |
| --- | --- | --- | --- | --- |
| Memarian et al. 2017 | 54 (18–90) | 100 | 82±7 | 14±3 |
| Sachdeva et al. 2014ab | Adults | 80 | 79±7 | - |
| Kimura 1982 | Adults | 50 | 70±4 | - |
| Okoseimiema and Udoaka 2013 | Adults | 100 | 74 | - |
| **Assumed for BPS (CV%)** | | | **76.2 (8)** | **14 (21)** |

*h_pu_ –* minimum width of pubic ramus superior

**Table P24**. Pubic-ramus-superior (lower part) macro-parameters for **adult male** (mean± STD, mm).

| Author | Age (range) | n | *l_pl_* | *h_symph_* | *w_pu(max)_* |
| --- | --- | --- | --- | --- | --- |
| Memarian et al. 2017 | 54 (18–90) | 100 | 28±4 | 43 ±7 | - |
| Kalenderer et al. 2017 | 18 | 30 | 37±13 | - | - |
| Lottering et al. 2014 | 44 (25–64) | 94 | - | - | 18±1 |
| **Assumed for BPS (CV%)** | | | **32 (18)** | **43* (17)** | **18**(3)** |

* Used for derivation of value *h_pl_* ; *h_pl_*=*h_symph_-h_p_*_u_ = 43 -14 =29 (mm, CV=20%); value of *h_p_*_u_ was taken from Table P23;

** Analysis of SUSHPU collection has shown: value of *w_pu_* (used for BPS) is smaller than *w_pu(max)_* by about 3 mm; it was assumed that *w_pu_* = 18 - 3 =15 (mm, CV=20%)

*l_pl_*– midwidth of pubis body (The shortest distance between the midpoint of symphysis pubis and the inner edge of obturator hole).

*w_pu(max)_–* maximal width of the symphyseal surface.

**Table P25**. Pubic-ramus-superior (upper part) macro-parameters for **adult female** (mean± STD, mm).

| Author | Age (range) | n | *PL* | | *h_pu_* | |
| --- | --- | --- | --- | --- | --- | --- |
|  |  |  | M | SD | M | SD |
| Memarian et al. 2017 | 54 (18–90) | 100 | 87 | 8 | 11 | 2 |
| Sachdeva et al. 2014ab | Adults | 20 | 86 | 5 | - | - |
| Kimura 1982 | Adults | 51 | 74 | 5 | - | - |
| Okoseimiema and Udoaka 2013 | 18-75 | 259 | 84 | 7 | - | - |
| **Assumed for BPS (CV%)** | | | **83 (7)** | | **11 (18)** | |

**Table P26**. Pubic-ramus-superior (lower part) macro-parameters for **adult female** (mean± STD, mm).

| Author | Age (range) | n | *l_pl_* | | *h_symph_* | | *w_pu(max)_* | |
| --- | --- | --- | --- | --- | --- | --- | --- | --- |
|  |  |  | M | SD | M | SD | M | SD |
| Memarian et al. 2017 | 54 (18–90) | 100 | 26 | 7 | 31 | 4 | - | - |
| Kalenderer et al. 2017 | 18 | 30 | 39 | 5 | - | - | - | - |
| Lottering et al. 2014 | 44 (25–64) | 94 | - | - | - | - | 14 | 1 |
| Bogusiewicz M 2011 | 56 (21-81) | 122 | 35 | 5 | - | - | - | - |
| **Assumed for BPS (CV%)** | | | **33 (18)** | | **31* (13)** | | **14**(7)** | |

* Used for derivation of value *h_pl_*; *h_pl_*=*h_symph_-h_p_*_u_ = 33 -14 =19 (мм, CV=20%); value of *h_p_*_u_ was taken from Table 25;

** Analysis of SUSHPU collection has shown: value of *w_pu_* (used for BPS) is smaller than *w_pu(max)_* by about 3 mm; it was assumed that *w_pu_* = 14 - 3 =11 (mm, CV=20%)

**Table P27.** Pubic-ramus-inferior macro-parameters for combined set of male and female (mean± STD, mm), results of measurements.

| Author | Age | n | *d1_pi_* | *d2_pi_* | *d3_pi_* | *d4_pi_* | *h_pi_* |
| --- | --- | --- | --- | --- | --- | --- | --- |
| SUSHPU measurements | Adult | 11 | 16±4 | 22±5 | 26±6 | 14±5 | 47±8 |
| **Assumed for BPS (CV%)** | | | **16 (25)** | **22 (23)** | **26 (23)** | **14 (36)** | **47 (17)** |

*Comments: d1_pi_* **–** thickness measured at the middle of the bone at the level of the ischial side of obturator hole; *d2_pi_* **–** length of the line located perpendicular to *d1_pi_* at the level of the ischial side of obturator hole; *d3_pi_* **–** thickness measured at the middle of the bone at the level of the pubic side of obturator hole; *d4_pi_* **–** length of the line located perpendicular to *d3_pi_* at the level of the ischial side of obturator hole; *h_pi_* **-** distance between the ischial and pubic sides of obturator hole along the surface of the pubic ramus inferior.

**Table P28**. Ischial length (*IL)* for adult **female**, mm

| Author | Age (range) | n | M | SD |
| --- | --- | --- | --- | --- |
| Washburn et al. 1948 | Adults | 100 | 78 | - |
| Sachdeva et al. 2014ab* | Adults | 20 | 73 | 8 |
| Okoseimiema et al. 2013 | Adults | 259 | 79 | 6 |
| Memarian et al. 2017 | Adults | 100 | 81 | 9 |
| **Assumed for BPS (CV%)** | | | **78 (9)** | |

*male+female

**Table P29**. Ischial length (*IL)* for adult **male**, mm

| Author | Age (range) | n | M | SD |
| --- | --- | --- | --- | --- |
| Salim 2012 | 19–101 | 66 | 89 | 5 |
| Sachdeva et al. 2014ab* | Adults | 100 | 80 | 5 |
| Davivong 1963 | Adults | 100 | 88 | - |
| Ekanem et al. 2009 | Adults | 114 | 70 | 11 |
| **Assumed for BPS (CV%)** | |  | **74(8)** | |

*male+female

Diameters of adult ischium were obtained from the measurements of SUSHPU -collection (m+f; n=11): ***d1_is_* =34±3.7; *d2_is_* =25±2.5**

**Table P30.** Published data on acetabulum measurements for adult males and females, mm.

| Author | Age | n | *d_a_* | SD | *h_a_* | SD |
| --- | --- | --- | --- | --- | --- | --- |
| Anisimova et al. 2014 | 21–75 | 98 | 57,4 | 7 | - | - |
| Rajkumar et al. 2012 | Adults | 154 | 48 | 4 | 27 | 4 |
| Aksu 2006 | Adults | 1 | 54,3 | - | 30 | - |
| Dhindsa 2013 | Adults | 50 | 51 | 3 | 27 | 3 |
| Chauhan et al. 2002 | Adults | 1 | 47 | - | 28 | - |
| Mukhopadhaya and Barooah 1967 | Adults | 1 | 46 | - | 25 | - |
| Luna et al. 1998 | Adults | 1 | 50 | - | 26 | - |
| Salamon et al. 2004 | Adults | 30 | 52 | - | 30 | 3 |
| Pratibha et al. 2015 | Adults | 1 | 48 | - | 30 | - |
| Bonnin et al. 2012 | 72±8 | 60 | 57 | 3 | 33 | 2 |
| Parmara et al. 2013 | Adults | 1 | 49 | 4 | 26 | 2 |
| Vyas et al. 2013 | Adults | 152 | 48 | 3 | 27 | 3 |
| **Assumed for BPS (CV%)** | |  | **52 (10)** | | **29 (10)** | |

Comments*: d_a_*– outer diameter of acetabulum; *h_a_*–acetabular depth

*Average thickness of the acetabulum wall* was estimated using the data of Anisimova (2013); data for anterior, posterior, inferior and superior walls were average by us; resulted value of *Th_aw_* = 10±2 mm; bottom wall thickness was taken *Th_ab_* =3.6±1.1 mm (completely cortical layer).

**Table P31.** Cortical thickness of pelvis bones for adult male (mean± STD, mm), published data

| Author | Location | Age | n | Ct.Th, mm |
| --- | --- | --- | --- | --- |
| Rehman et al. 1994 | Iliac crest | 46 (18–75) | 76 | 1.1±0.3 |
| Ostertag et al. 2009 | Iliac crest | 52±8 | 47 | 0.9±0.2 |
| **Average for iliac crest BPS (CV%)** | | |  | **1.0 (15)** |
| Lottering et al. 2014 | Pubic ramus superior (symphysis articular surface) | 44 (25–64) | 94 | 1.49±0.18 |

Illustrative schemes of cortical thickness distribution over the pelvic surface (Anderson et al. 2005; Kunitomi et al. 2017) were used for derivation of Ct,Th values. For pubic ramus inferior, ramus of ischium, and acetabulum walls Ct,Th was taken to be 0.5 mm; for non-articular sides of pubic ramus superior Ct,Th was taken to be 0.7; CV was taken to be the same as for iliac crest (15%).

**Analysis of published data on pelvis microstructures**

**(ilium, ischium and pubis)**

**Table P32**. Published data on ilium BV/TV and Tb.Th for pre-adult, mm.

| Author | Age | n | BV/TV | SD | Tb.Th | SD Tb.Th |
| --- | --- | --- | --- | --- | --- | --- |
| Cunningham 2009* | 0 | 23 | 0.326 | 0.072 | 0.169 | 0.024 |
| Volpato 2008* | 0.12 (0–0.25) | 2 | 0.21 | - | 0.137 | - |
| Volpato 2008* | 1.5(1–2) | 5 | 0.33 | - | 0.169 | 0.024 |
| Gloreiex 2000 | 3 (1.5–6.9) | 10 | 0.177 | 0.026 | 0.1 | - |
| Volpato 2008* | 7.5(5–10) | 5 | 0.26 | - | 0.168 | - |
| Nguen et al. 2015 | 7.4 (5.5–9.4) | 32 | - | - | 0.176 | 0.020 |
| Gloreiex 2000 | 8.5(7.0–10.9) | 10 | 0.224 | 0.042 | 0.129 | 0.017 |
| Gloreiex 2000 | 12.5 (11–13.9) | 14 | 0.244 | 0.043 | 0.148 | 0.023 |
| Pereira et al. 2016 | 15±3.8 | 31 | 0.214 | 0.015 | 0.141 | 0.014 |
| Gloreiex 2000 | 15.5 (14–16.9) | 12 | 0.257 | 0.053 | 0.157 | 0.022 |
| Gloreiex 2000 | 19 (17–22.9) | 12 | 0.278 | 0.045 | 0.153 | 0.02 |

*- average iliac data; in other cases, the data on iliac crest

**Table P33.** Published data on ilium Th.Sp for pre-adult, mm.

| Author | Age | M | SD | Min | Max | N |
| --- | --- | --- | --- | --- | --- | --- |
| Cunningham 2009 | 0 | 0.315 | 0.8 | 0.100* | 0.167* | 23 |
| Gloreiex 2000 | 3 (1.5–6.9) | 0.481 | 0.112 | 0.257 | 0.705 | 10 |
| Gloreiex 2000 | 8.5(7.0–10.9) | 0.453 | 0.062 | 0.329 | 0.577 | 10 |
| Gloreiex 2000 | 12.5 (11–13.9) | 0.464 | 0.078 | 0.308 | 0.62 | 14 |
| Gloreiex 2000 | 15.5 (14–16.9) | 0.461 | 0.07 | 0.321 | 0.601 | 12 |
| Pereira et al. 2016 | 15±14.4 | 0.459 | 0.022 | 0.415 | 0.503 | 14 |

*- according to primary data, for the remaining groups M+2SD

**Table P34**. Published data on pelvic bones microstructure of adults, mm.

| Author | Age | n | BV/TV | SD | Tb.Th | SD | Tb.Sp | SD |  |
| --- | --- | --- | --- | --- | --- | --- | --- | --- | --- |
| Iliac crest | | | | | | | | | |
| Shahtheri 2006 | 20-49 | 28 | 0.24 | 0.05 | 0.112 | 0.025 | 0.29 | 0.07 |  |
| Ostojić et al. 2006 | 41-50 | 7 | 0.20 | 0.04 | 0.12 | 0.03 | 0.51 | 0.09 |  |
| Rehman et al. 1994 | 18-55 | 51 | 0.22 | 0.05 | 0.14 | 0.03 | 0.5 | 0.1 |  |
| Cohen et al. 2010 | 39±10 | 54 | 0.19 | 0.05 | 0.11 | 0.02 | 0.5 | 0.13 |  |
| Ulrich et al. 1999 | 69 | 62 | 0.152 | 0.05 | 0.15 | 0.03 | 0.8 | 0.1 |  |
| Tamminen et al. 2011 | 46 | 36 | 0.221 | 0.05 | 0.104 | 0.03 | 0.45 | 0.14 |  |
| Parfitt et al. 1983 | 32±10 | 19 | 0.24 | 0.07 | - | - | - | - |  |
| Qiu et al. 2006 | 37±7,8 | 43 | 0.245 | 0.07 | 0.14 | 0.02 | - | - |  |
| Ostertag et al. 2009 | 52±8 | 47 | 0.147 | 0.03 | 0.12 | 0,02 | 0.7 | 0.15 |  |
| Ilium (posterior part of the auricular surface) | | | | | | | | | |
| Deguette et al. 2014* | 40 (30–50) | 10 | 0.176 | 0.03 | 0.3 | 0.01 | 1.0 | 0.12 |  |
| Pubis (ramus inferior) | | | | | | | | | |
| Wade et al. 2011 | 30–89 | 65 | 0.25 | 0.08 | 0.31 | 0.023 | 1.0 | 0.44 |  |
| Pubis (ramus superior, symphyseal area) | | | | | | | | |  |
| Jadzic et al. 2021 | <45 | 23 | 0.17 | 0.03 | 0.17 | 0.025 | 0.75 | 0.15 |  |

*values were averaged by us over the three sampled volumes of interest

**Table P35.** Parameters assumed for ilium (all segments); BV/TV in relative units; other parameters in mm.

| Age group | BV/TV  (min–max) | SD BV/TV | Tb.Th  (min–max) | SD Tb.Th | Tb.Sp  (min–max) | SD Tb.Sp |
| --- | --- | --- | --- | --- | --- | --- |
| 0 | 0.317*  (0.128–0.563) | 0.072 | 0.166*  (0.12–0.266) | 0.024 | 0.315*  (0.167–0.8) | 0.1 |
| 1 | 0.228  (0.176–0.28) | 0.026 | 0.123  (0.075–0.171) | 0.024 | 0.481  (0.257–0.705) | 0.112 |
| 5 | 0.246  (0.198–0.294) | 0.024 | 0.153  (0.122–0.188) | 0.016 | 0.481  (0.257–0.705) | 0.112 |
| 10 | 0.246  (0.198–0.294) | 0.024 | 0.155  (0.121–0.185) | 0.016 | 0.459  (0.308–0.620) | 0.070 |
| 15 | 0.246  (0.198–0.294) | 0.024 | 0.155  (0.198–0.294) | 0.016 | 0.459  (0.319–0.599) | 0.070 |
| Adults | 0.19 | 0.050 | 0.130 | 0.020 | 0.60 | 0.120 |

*- minimum and maximum according to primary data of Cunningham (2009) for the remaining age-groups M+2SD

For ischium and pubis (all segments from 0-Y to 15-Y), BV/TV and Th.Th were taken to be the same as for ilium (Table P35). For Tb.Sp a gradual increase from 10-Y was assumed so as to reach a value of 1.0 at 25 years. SD was taken to be equal to ilium values.

**Table P36.** Parameters assumed for ischium and pubis (all segments), BV/TV in relative units; other parameters in mm.

| Age group | BV/TV  (min–max) | SD | Tb.Th  (min–max) | SD | Tb.Sp  (min–max) | SD |
| --- | --- | --- | --- | --- | --- | --- |
| 0 | 0.317  (0.128–0.563) | 0.072 | 0.166  (0.12–0.266) | 0.024 | 0.315  (0.167–0.8) | 0.1 |
| 1 | 0.228  (0.176–0.28) | 0.026 | 0.123  (0.075–0.171) | 0.024 | 0.481  (0.257–0.705) | 0.112 |
| 5 | 0.246  (0.198–0.294) | 0.024 | 0.153  (0.122–0.188) | 0.016 | 0.481  (0.257–0.705) | 0.112 |
| 10 | 0.246  (0.198–0.294) | 0.024 | 0.155  (0.121–0.185) | 0.016 | 0.600  (0.46–0.74) | 0.070 |
| 15 | 0.246  (0.198–0.294) | 0.024 | 0.155  (0.121–0.185) | 0.016 | 0.750  (0.61–0.89) | 0.070 |
| Adults* | 0.25  (0.23-0.27) | 0.01 | 0.300  (0.260-340) | 0.02 | 1.0  (0.2-1.8) | 0.400 |
| Adults** | 0.17  (0.11-0.23) | 0.03 | 0.17  (0.1-0.24) | 0.03 | 0.75  (0.3-1.1) | 0.150 |

*Used for pubic ramus inferior and ischium

**Used for pubic ramus superior

**References for pelvic bones**

Aksu FT, Çeri GN, Arman C, Tetik S Morphology and Morphometry of The Acetabulum. DEÜ Tıp Fakültesi Dergisi. 2006; 20(3): 143–148.

Ekanem TB, Akpan EJ, Mesembe OE. A Study of Ischiopubic Index Using X-Ray Films in Lagos State of Nigeria. Advances in Anatomy. Advances in Anatomy; 2014;2014: 1–4. doi:10.1155/2014/192897

Anderson AE, Peters CL, Tuttle BD, Weiss JA. Subject-specific finite element model of the pelvis: development, validation and sensitivity studies. J Biomech Eng. 2005; 127(3):364–73.

Anisimova EA, Yusupov KS, Anisimov DI, Bondareva EV. Morphology of bone structures of acetabulum and femoral component of hip joint. Saratov Journal of Medical Scientific Research. 2014; 10(1):32–38. (in Russian)

Blake KAS. An investigation of sex determination from the subadult pelvis: A morphometric analysis. Doctoral Dissertation, University of Pittsburgh. 2011.

Bogusiewicz M, Rosińska-Bogusiewicz K, Drop A, Rechberger T. Anatomical variation of bony pelvis from the viewpoint of transobturator sling placement for stress urinary incontinence. International Urogynecology Journal. 2011;22(8):1005-1009.

Bonnin MP, Archbold PH, Basiglini L, Fessy MH, Beverland DE. Do we medialise the hip centre of rotation in total hip arthroplasty? Influence of acetabular offset and surgical technique. Hip Int. 2012; 22(4):371–378.

Chauhan R, Paul S, Dhaon BK. Anatomical parameters of North Indian hip joints- cadaveric study. J Anat Soc India. 2002; 51:39–42.

Cohen A, Dempster DW, Müller R, Guo XE, Nickolas TL, Liu XS, Zhang XH, Wirth AJ, van Lenthe GH, Kohler T, McMahon DJ, Zhou H, Rubin MR, Bilezikian JP, Lappe JM, Recker RR, Shane E. Assessment of trabecular and cortical architecture and mechanical competence of bone by high-resolution peripheral computed tomography: comparison with transiliac bone biopsy. Osteoporos Int. 2010; 21(2):263–273.

Corron L, Marchal F, Condemi S, Chaumoître K, Adalian P. [A New Approach of Juvenile Age Estimation using Measurements of the Ilium and Multivariate Adaptive Regression Splines (MARS) Models for Better Age Prediction.](https://www.ncbi.nlm.nih.gov/pubmed/27792240) Forensic Sci. 2017 Jan;62(1):18–29. doi: 10.1111/1556-4029.13224.

Cunningham CA, Black SM. [Iliac cortical thickness in the neonate - the gradient effect.](https://www.ncbi.nlm.nih.gov/pubmed/19549003) J Anat. 2009a Sep;215(3):364–70. doi: 10.1111/j.1469-7580.2009.01112.x.

Cunningham CA, Black SM. Anticipating bipedalism: trabecular organization in the newborn ilium. J Anat. 2009b Jun;214(6):817–29. doi: 10.1111/j.1469-7580.2009.01073.x

Cunningham C, Scheuer L, Black S. Developmental Juvenile Osteology. Second Edition. Elsevier Academic Press. 2016.

Davivong, V. The pelvic girdle of the Australian aborigines, sex differences, sex determination. Am. J. Phys. Anthropol. 1963; 21:443–455.

Deguette C, Chappard D, Libouban H, Airagnes G, Rouge-maillart C, Telmon N. The contribution of Micro-CT to the evaluation of trabecular bone at the posterior part of the auricular surface in men. Int J Legal Med. 2018; 132(4):1231–1239. doi: 10.1007/s00414-014-1139-1.

Dhindsa GS, Singh P, Singh Z. Morphometry of the adult human dry hip bone. Int J Pharm Pharm Sci. 2013; 5(2): 505-507.

[Glorieux FH](https://www.ncbi.nlm.nih.gov/pubmed/?term=Glorieux%20FH%5BAuthor%5D&cauthor=true&cauthor_uid=10678403), [Travers R](https://www.ncbi.nlm.nih.gov/pubmed/?term=Travers%20R%5BAuthor%5D&cauthor=true&cauthor_uid=10678403), [Taylor A](https://www.ncbi.nlm.nih.gov/pubmed/?term=Taylor%20A%5BAuthor%5D&cauthor=true&cauthor_uid=10678403), [Bowen JR](https://www.ncbi.nlm.nih.gov/pubmed/?term=Bowen%20JR%5BAuthor%5D&cauthor=true&cauthor_uid=10678403), [Rauch F](https://www.ncbi.nlm.nih.gov/pubmed/?term=Rauch%20F%5BAuthor%5D&cauthor=true&cauthor_uid=10678403), [Norman M](https://www.ncbi.nlm.nih.gov/pubmed/?term=Norman%20M%5BAuthor%5D&cauthor=true&cauthor_uid=10678403), [Parfitt AM](https://www.ncbi.nlm.nih.gov/pubmed/?term=Parfitt%20AM%5BAuthor%5D&cauthor=true&cauthor_uid=10678403). Normative data for iliac bone histomorphometry in growing children. [Bone.](https://www.ncbi.nlm.nih.gov/pubmed/?term=Normative+Data+for+Iliac+Bone+Histomorphometry+in+Growing+Children) 2000 Feb;26(2):103–9.

Jadzic J, Mijucic J, Nikolic S, Djuric M, Djonic D. The comparison of age- and sex-specific alteration in pubic bone microstructure: A cross-sectional cadaveric study. Exp Gerontol. 2021 Jul 15;150:111375. doi: 10.1016/j.exger.2021.111375. Epub 2021 Apr 30. PMID: 33940115.

Hernigou J, Picard L, Alves A, Silvera J, Homma Y, Hernigou P. Understanding bone safety zones during bone marrow aspiration from the iliac crest: the sector rule. Int Orthop. 2014; 38(11):2377–2384.

Kalenderer Ö, Turgut A, Bacaksız T, Bilgin E, Kumbaracı M, Akkan HA. Evaluation of symphysis pubis and sacroiliac joint distances in skeletally immature patients: A computerized tomography study of 1020 individuals. Acta Orthop Traumatol Turc. 2017 Mar;51(2):150–154.

Khamanarong K, Kosuwon W, Sirichativapee W, Saejung S, Thepsuthammarat K. Thicknesses of the iliac crest appropriate for anterior cervical interbody fusion grafts J Med Assoc Thai. 2005; 88(12):1892–1895.

Kimura K. Sex differences of the hip bone among several populations. Okajimas Folia Anat Jpn 1982; 58(4-6):265–276

Kunitomi S, Yamamoto Y, Kato R, Antona‐Makoshi, Jacobo; Konosu A, Dokko Y, Yasuki, T. The development of the lower extremity of a human FE model and the influence of anatomical detailed modelling in vehicle‐to‐pedestrian impacts. Proceedings of the 2017 International IRCOBI Conference on the Biomechanics of Injury. 2017.

Lottering N, Reynolds MS, MacGregor DM, Meredith M, Gregory LS. Morphometric modelling of ageing in the human pubic symphysis: sexual dimorphism in an Australian population. Forensic Sci Int. 2014 Mar; 236:195. e1-11.

Luna MP, Desnoyers V, Charissoux JL, Mavit C, Arnaud JP. Morfological study of the human acetabulum: Biometry. Rev Chil Anat. 1998; 16: 5–7.

[Maclean SJ](https://www.ncbi.nlm.nih.gov/pubmed/?term=Maclean%20SJ%5BAuthor%5D&cauthor=true&cauthor_uid=24639178), [Black SM](https://www.ncbi.nlm.nih.gov/pubmed/?term=Black%20SM%5BAuthor%5D&cauthor=true&cauthor_uid=24639178), [Cunningham CA](https://www.ncbi.nlm.nih.gov/pubmed/?term=Cunningham%20CA%5BAuthor%5D&cauthor=true&cauthor_uid=24639178). The developing juvenile ischium: macro-radiographic insights. [Clin Anat.](https://www.ncbi.nlm.nih.gov/pubmed/?term=The+Developing+Juvenile+Ischium%3A+Macro-Radiographic+Insights) 2014 Sep;27(6):906–14. doi: 10.1002/ca.22391.

Memarian A, Aghakhani K, Mehrpisheh S, Fares F. Gender determination from diagnostic factors on anteroposterior pelvic radiographs. J Chin Med Assoc. 2017 Mar;80(3):161–168.

Molleson T. and Cox M. The Spitalfields Project Volume 2 – The Anthropology – The Middling Sort, Research Report 86. London: Council for British Archaeology. 1993.

Mukhopadhaya B, Barooah B. Osteoarthritis of hip in Indians: an anatomical and clinical study. Indian J Orthop. 1967; 1: 55–62.

Nguyen TV, Melville A, Nath S, Story C, Howell S, Sutton R, et al. Bone Marrow Recovery by Morphometry during Induction Chemotherapy for Acute Lymphoblastic Leukemia in Children. PLoS One. 2015;10(5):e0126233.

Okoseimiema SC, Udoaka AI. Radiological Determination of Ischio-pubic Index in South- South Nigerian Population. Asian Journal of med Sci. 2013; 5(5):96–100.

Ostertag A, Cohen-Solal M, Audran M, Legrand E, Marty C, Chappard D, de Vernejoul MC. Vertebral fractures are associated with increased cortical porosity in iliac crest bone biopsy of men with idiopathic osteoporosis. Bone. 2009; 44(3):413–417.

Ostojić Z, Cvijanović O, Bobinac D, Zoricić S, Sosa I, Marić I, Crncević-Orlić Z, Mihelić R, Ostojić L, Petrović P. Age-related and gender-related differences between human vertebral and iliac crest bone–a histomorphometric study on the population of the Mediterranean Coast of Croatia. Coll Antropol. 2006; 30(1):49–54.

Parfitt AM, Mathews CH, Villanueva A R, Kleerekoper M, Frame B, Rao D S. Relationships between surface, volume, and thickness of iliac trabecular bone in aging and in osteoporosis. Implications for the microanatomic and cellular mechanisms of bone loss. J Clin Invest. 1983; 72(4): 1396–1409.

Parfitt AM, Travers R, Rauch F, Glorieux FH. [Structural and cellular changes during bone growth in healthy children.](https://www.ncbi.nlm.nih.gov/pubmed/11033443) Bone. 2000 Oct;27(4):487–94. PMID: 11033443

Parmara G, Rupareliab S, Patelc SV, Patelb SM, Jethvaa N. Morphology and morphometry of acetabulum. Int J Biol Med Res. 2013; 4(1): 2924–2926.

Pereira RC, Bischoff DS, Yamaguchi D, Salusky IB, Wesseling-Perry K. [Micro-CT in the Assessment of Pediatric Renal Osteodystrophy by Bone Histomorphometry](https://www.ncbi.nlm.nih.gov/pubmed/26712809). Clin J Am Soc Nephrol. 2016 Mar 7;11(3):481–7. doi: 10.2215/CJN.04810515.

Pratiibha K, Hema L, Devishankar. Acetabulum of the hip bone: A morphometric study with coastal region. International Journal of Recent Trends in science and Technology. 2015; 17(2):136–139.

[Qiu S](https://www.ncbi.nlm.nih.gov/pubmed/?term=Qiu%20S%5BAuthor%5D&cauthor=true&cauthor_uid=17002584), [Rao DS](https://www.ncbi.nlm.nih.gov/pubmed/?term=Rao%20DS%5BAuthor%5D&cauthor=true&cauthor_uid=17002584), [Palnitkar S](https://www.ncbi.nlm.nih.gov/pubmed/?term=Palnitkar%20S%5BAuthor%5D&cauthor=true&cauthor_uid=17002584), [Parfitt AM](https://www.ncbi.nlm.nih.gov/pubmed/?term=Parfitt%20AM%5BAuthor%5D&cauthor=true&cauthor_uid=17002584). Independent and combined contributions of cancellous and cortical bone deficits to vertebral fracture risk in postmenopausal women. [J Bone Miner Res.](https://www.ncbi.nlm.nih.gov/pubmed/17002584) 2006 Nov;21(11):1791–6.

Quain's anatomy, edited by William Sharpey, Allen Thomson and John Cleland. London: Walton and Maberly, 1864–1867. STORE 115:17–19.

Rajkumar, Sachan A, Meena KR. The Relationship between Depth and Diameter of Human Acetabulum in Dry Hip Bone of Indian Population. International Journal of Science and Research (IJSR). 2012; 3(12): 1574–1576.

Rehman MT, Hoyland JA, Denton J, Freemont AJ. Age related histomorphometric changes in bone in normal British men and women. J Clin Pathol. 1994; 47(6):529–534.

Rissech C, Garcıa M, Malgosa A. Sex and age diagnosis by ischium morphometric analysis. Forensic Science International. 2003; 135: 188–196.

Rissech C, Malgosa A. [Ilium growth study: applicability in sex and age diagnosis.](https://www.ncbi.nlm.nih.gov/pubmed/15567622) Forensic Sci Int. 2005 Jan 29;147(2-3):165–74.

Rissech C, Malgosa A. Pubis growth study: Applicability in sexual and age diagnostic. Forensic Science International. 2007; 173: 137–145.

Sachdeva K, Kumar R, Kalsey G. Role of ilium in sexual dimorphism of hip bone: a morphometric study in north indian population. Int J Anat Res. 2014a; 2(3):524–532.

Sachdeva K, Singla RK, Kalsey G. Role of ischio-pubic index in sex identification from innominate bones in north Indian population. Int J Anat Res. 2014b; 2(3):515–20.

Sachdeva K, Singla R, Kalsey G. Sexual dimorphism in various angles of north Indian hip bone- A rarely explored zone. Indian Journal of Clinical Anatomy and Physiology 2019;6(4):509–514.

Salamon A, Salamon T, Sef D, Jo-Osvatic A. Morphological characteristics of the acetabulum. Coll Anthropol 2004; 28 Suppl 2: 221–226.

Salim Z. Accuracy and reliability in sex determination using the os coxa: a comparison of metric vs. Phenice method. A Thesis Presented to The Faculty of the Department of Anthropology The University of Houston In Partial Fulfillment Of the Requirements for the Degree of Master of Arts. 2012.

Scheuer L, Black S. The juvenile Skeleton. Elsevier Academic Press London WC1X 8RR, UK. 2004.

[Schnitzler CM](https://www.ncbi.nlm.nih.gov/pubmed/?term=Schnitzler%20CM%5BAuthor%5D&cauthor=true&cauthor_uid=19136082), [Mesquita JM](https://www.ncbi.nlm.nih.gov/pubmed/?term=Mesquita%20JM%5BAuthor%5D&cauthor=true&cauthor_uid=19136082), [Pettifor JM](https://www.ncbi.nlm.nih.gov/pubmed/?term=Pettifor%20JM%5BAuthor%5D&cauthor=true&cauthor_uid=19136082). Cortical bone development in black and white South African children: iliac crest histomorphometry. [Bone.](https://www.ncbi.nlm.nih.gov/pubmed/?term=Cortical+bone+development+in+black+and+white+South+African+children%3A+Iliac+crest+histomorphometry) 2009 Apr;44(4):603–11. doi: 10.1016/j.bone.2008.12.009.

Shahtaheri SM. Comparison of Cancellous Bone Histomorphometry Between Young Men and Women. Pakistan Journal of Biological Sciences. 2006; 9: 1338–1341.

Sönmez TT, Prescher A, Salama A, Kanatas A, Zor F, Mitchell D. Zaker Shahrak A, Karaaltin MV, Knobe M, Külahci Y, Altuntas SH, Ghassemi A, Hölzle F. Comparative clinicoanatomical study of ilium and fibula as two commonly used bony donor sites for maxillofacial reconstruction. Br J Oral Maxillofac Surg. 2013; 51:736–741.

Tamminen IS, Isaksson H, Aula AS, Honkanen E, Jurvelin JS, Kröger H. Reproducibility and agreement of micro-CT and histomorphometry in human trabecular bone with different metabolic status. J Bone Miner Metab. 2011;29(4):442‐448.

Ulrich D, van Rietbergen B, Laib A, Rüegsegger P. The ability of three-dimensional structural indices to reflect mechanical aspects of trabecular bone. Bone. 1999;25(1):55‐60.

Volpato V. Bone endostructure morphogenesis of the human ilium. C. R. Palevol 7 (2008) pages 463–471 Doi: 10.1016/j.crpv.2008.06.001

Vyas K, Shroff B, Zanzrukiya K. An osseous study of morphological aspect of acetabulum of hip bone. Int J Res Med. 2013; 2(1):78–82.

Wade A, Nelson A, Garvin G, Holdsworth DW. Preliminary radiological assessment of age-related change in the trabecular structure of the human os pubis. J Forensic Sci. 2011; 56(2):312–319.

Washburn SL. Sex differences in the pubic bone. Am J Phys Anthropol. 1948 Jun;6(2):199-207

[Yusof NA](https://www.ncbi.nlm.nih.gov/pubmed/?term=Yusof%20NA%5BAuthor%5D&cauthor=true&cauthor_uid=24106059), [Soames RW](https://www.ncbi.nlm.nih.gov/pubmed/?term=Soames%20RW%5BAuthor%5D&cauthor=true&cauthor_uid=24106059), [Cunningham CA](https://www.ncbi.nlm.nih.gov/pubmed/?term=Cunningham%20CA%5BAuthor%5D&cauthor=true&cauthor_uid=24106059), [Black SM](https://www.ncbi.nlm.nih.gov/pubmed/?term=Black%20SM%5BAuthor%5D&cauthor=true&cauthor_uid=24106059). [Anat Rec (Hoboken).](https://www.ncbi.nlm.nih.gov/pubmed/?term=Yusof+2013+pelvis) Growth of the human ilium: the anomalous sacroiliac junction 2013 Nov;296(11):1688–94. doi: 10.1002/ar.22785.
