# Supplementary material for: Stochastic parametric skeletal dosimetry model for humans: Anatomical-morphological basis and parameter evaluation
Source: PLoS One. 2025 Jul 2;20(7):e0327156. doi: 10.1371/journal.pone.0327156 (PMC12306906; doi:10.1371/journal.pone.0327156)
Supplement: S4 Rib — (DOCX) [file pone.0327156.s004.docx]

**RIBS**

**Pre-adults, analysis of published data on rib macro-parameters and cortical thickness**

The ribs have a considerable length, so it was decided to simulate a segment that describes a part of the rib 30 mm long and having the shape of a box (Fig. R1). One BPS describes all the ribs. Measured parameters^^[[1]](#footnote-1)^^: *w* - media-lateral (smallest or horizontal) cross-sectional width; *h* - cranio-caudal (largest or vertical) cross-sectional height, average cortical thickness *Ct.Th* for midshaft of middle thoracic ribs. Table R1 presents the results of individual measurements of pre-adult ribs from a well-documented cemetery collection, Spitalfields Christ Church, London, UK, which is widely used to evaluate the parameters of individual skeleton bones (Cunningham et al. 2016; Molleson and Cox 1996).

**Fig. R1.** a- set of infant ribs; b –cross-section of rib (Child Bones); c - stylized models (BPSs); letter designations are deciphered in the text.

**Table R1.** Measured values of sizes and cortical thickness of middle thoracic ribs from pre-adults (mm).

| Individual code* | Sex | Age | Beresheim et al. 2019 | Pfeiffer et al. 2006 | |
| --- | --- | --- | --- | --- | --- |
|  |  |  | *Ct.Th* | *w* | *h* |
| 2250 | F | 0 | 0.532 | 2.92 | 3.73 |
| 2277 | M | 0 | 0.364 | 3.09 | 5.41 |
| 2147 | M | 0 | 0.249 | 3.69 | 8.04 |
| 2282 | F | 1 | 0.747 | 2.96 | 6.75 |
| 2206 | M | 2 | 0.46 | 4.92 | 10.7 |
| 2719 | Unk | 5 | 0.421 | - | - |
| 2264 | M | 7 | 0.726 | 4.73 | 13.9 |
| Continuation | | | | | |
| Individual code* | Sex | Age | *Ct.Th* | *w* | *h* |
| 2139 | M | 11 | 0.631 | 5.74 | 8.43 |
| 2677 | F | 12 | 0.772 | 4.48 | 10.5 |
| 2721 | F | 13 | 0.561 | 4.42 | 8.53 |
| 2175 | F | 15 | 0.992 | 4.8 | 9.93 |
| 2755 | F | 16 | 0.717 | 4.65 | 10.8 |
| 2104 | M | 16 | 0.831 | 5.8 | 10.7 |
| 2752 | F | 17 | 0.544 | 5.05 | 10.6 |
| 2698 | F | 17 | 0.855 | 4.86 | 9.38 |

* - is the original Catalog Number

**Table R2.** Assumed values of rib-BPSs parameters for pre-adults, mm

| Age | Ct.Th | SD | *w* | SD | *h* | SD |
| --- | --- | --- | --- | --- | --- | --- |
| 0 | 0.38 | 0.14 | 3.23 | 0.40 | 5.73 | 2.17 |
| 1 | 0.54 | 0.18 | 3.94 | 1.39 | 8.73 | 2.79 |
| 5 | 0.54 | 0.18 | 3.94 | 1.39 | 8.73 | 2.79 |
| 10 | 0.74 | 0.15 | 4.95 | 0.50 | 10.31 | 1.62 |

**Adults and 15-Y, analysis of published data on rib macro-parameters and cortical thickness**

Similar to children, rib BPS for adult and 15-Y is a fragment described by a box with a length of 30 mm (Fig. 1Rc). All adult ribs were grouped into 4 groups (types) with similar values of *h* and *w*. Rib length data were used to estimate the proportion of bone marrow in different types of ribs (with different BPSs). The cortical layer covers the four sides of the BPS.

1. BPS1- describes edges R1 and R2

2. BPS2 - describes edges R3, R4, R9, R10

3. BPS3 - describes the edges R5 - R8

4. BPS4 - describes edges R11 - R12

For the age of 15 years, parameter values are accepted, as for adults.

Table R3 and R4 presents the published data on rib sizes for female and male.

Table R5 presents the published data on rib cortical thickness.

Table R6 presents average values of *h* and *w* for each BPS which represents the piece of typical rib from the corresponding group of standard length (30 mm).

**Table R3.** Measured values of adult **female** rib sizes, mm.

| Author | Age | Rib | n | *l* | *SD* | *h* | SD | *w* | SD |
| --- | --- | --- | --- | --- | --- | --- | --- | --- | --- |
| Subit et al. 2015 | 20-84 | 1 | 19 | 120 | - | - | - | - | - |
| Gomez-Olivencia et al. 2010 | Adult | 1 | 32 | - | - | 15.4 | 1.8 | 4.1 | 0.6 |
| Gomez-Olivencia et al. 2009 | Adult | 1 | 33 | - | - | 16.9 | 2 | 5.4 | 0.8 |
| Elrod et al. 2012 | 58±12 | 1 | 48 | 130 | 12.5 | - | - | - | - |
| Elrod et al. 2012 | 42±14 | 1 | 28 | 127.5 | 10 | - | - | - | - |
| Subit et al. 2015 | 20-84 | 2 | 19 | 195 | - | - | - | - | - |
| Gomez-Olivencia et al. 2010 | Adult | 2 | 33 | - | - | 10.1 | 1.4 | - | - |
| Gomez-Olivencia et al. 2009 | Adult | 2 | 32 | - | - | 13.7 | 1.6 | 5.3 | 1 |
| Subit et al. 2015 | 20-84 | 3 | 19 | 235 | - | - | - | - | - |
| Chapman et al. 2017 | 30±6 | 3 | 7 | 236.3 | 8.3 | - | - | - | - |
| Cirillo and Henneburg, 2012 | 78±9 | 3 | 5 | 272.4 | 7 | 12.2 | 0.4 | 8.1 | 0.3 |
| Roberts and Chen 1971 | Adult | 3 | 1 | - | - | 11.9 | 4.7 |  |  |
| Gomez-Olivencia et al. 2010 | Adult | 3 | 33 | - | - | 8.2 | 1.2 | 5.2 | 0.8 |
| Gomez-Olivencia et al. 2009 | Adult | 3 | 32 | - | - | 9.3 | 1 | 6.7 | 1.2 |
| Subit et al. 2015 | 20-84 | 4 | 19 | 258.5 | - | - | - | - | - |
| Chapman et al. 2017 | 30±6 | 4 | 7 | 261.6 | 9.2 | - | - | - | - |
| Cirillo and Henneburg, 2012 | 78±9 | 4 | 5 | 302.6 | 5.5 | 13.2 | 0.3 | 9.2 | 0.3 |
| Gomez-Olivencia et al. 2010 | Adult | 4 | 33 | - | - | 9 | 1.2 | 5.2 | 0.7 |
| Gomez-Olivencia et al. 2010 | Adult | 4 | 32 | - | - | 9.9 | 1.4 | 7 | 0.9 |
| Subit et al. 2015 | 20-84 | 5 | 19 | 275 | - | - | - | - | - |
| Chapman et al. 2017 | 30±6 | 5 | 7 | 275.8 | 10.8 | - | - | - | - |
| Cirillo and Henneburg, 2012 | 78±9 | 5 | 5 | 315.5 | 5.2 | 13.9 | 0.4 | 9.7 | 0.4 |
| Roberts and Chen, 1971 | Adult | 5 | 1 | - | - | 11.7 | 5.9 |  |  |
| Gomez-Olivencia et al. 2010 | Adult | 5 | 33 | - | - | 10.1 | 1.3 | 5.2 | 0.6 |
| Gomez-Olivencia et al. 2009 | Adult | 5 | 32 | - | - | 11.2 | 1.9 | 7.2 | 1 |
| Subit et al. 2015 | 20-84 | 6 | 19 | 285 | - | - | - | - | - |
| Chapman et al. 2017 | 30±6 | 6 | 7 | 281.5 | 11.8 | - | - | - | - |
| Cirillo and Henneburg, 2012 | 78±9 | 6 | 5 | 318 | 6.2 | 14.7 | 0.5 | 10.8 | 0.4 |
| Gomez-Olivencia et al. 2010 | Adult | 6 | 33 | - | - | 9.4 | 1.1 | 6.2 | 0.7 |
| Gomez-Olivencia et al. 2009 | Adult | 6 | 32 | - | - | 12.5 | 1.6 | 6.8 | 0.9 |
| Subit et al. 2015 | 20-84 | 7 | 19 | 284.5 | - | - | - | - | - |
| Chapman et al. 2017 | 30±6 | 7 | 7 | 280.4 | 13.6 | - | - | - | - |
| Cirillo and Henneburg, 2012 | 78±9 | 7 | 5 | 317.7 | 6 | 16.1 | 0.7 | 12.2 | 0.3 |
| Roberts and Chen, 1971 | Adult | 7 | 1 | - | - | 13.6 | 6.1 | - | - |
| Gomez-Olivencia et al. 2010 | Adult | 7 | 33 | - | - | 9.7 | 0.9 | 6.1 | 0.7 |
| Gomez-Olivencia et al. 2009 | Adult | 7 | 32 | - | - | 13.6 | 1.7 | 6.6 | 0.9 |
| Subit et al. 2015 | 20-84 | 8 | 19 | 275 | - | - | - | - | - |
| Chapman et al. 2017 | 30±6 | 8 | 7 | 268.1 | 14.7 | - | - | - | - |
| Cirillo and Henneburg, 2012 | 78±9 | 8 | 5 | 304.3 | 5.2 | 14.7 | 0.5 | 12.9 | 0.3 |
| Gomez-Olivencia et al. 2009 | Adult | 8 | 32 | - | - | 13.6 | 1.8 | 5.6 | 1.1 |
| Subit et al. 2015 | 20-84 | 9 | 19 | 252.5 | - | - | - | - | - |
| Chapman et al. 2017 | 30±6 | 9 | 7 | 245.3 | 12 | - | - | - | - |
| Cirillo and Henneburg, 2012 | 76±9 | 9 | 5 | 280.2 | - | 13.6 | 0.4 | 13.4 | 0.3 |
| Gomez-Olivencia et al. 2009 | Adult | 9 | 32 | - | - | 14.4 | 1.9 | 5.4 | 0.9 |
| Subit et al. 2015 | 20-84 | 10 | 19 | 217.5 | - | - | - | - | - |
| Cirillo and Henneburg, 2012 | 76±9 | 10 | 5 | 236.8 | - | 12.2 | 0.4 | - | - |
| Gomez-Olivencia et al. 2010 | Adult | 10 | 33 | - | - | 8.5 | 0.8 | 5.4 | 0.58 |
| Gomez-Olivencia et al. 2009 | Adult | 10 | 32 | 227.9 | 17.4 | 13.6 | 2 | 4.7 | 0.9 |
| Gomez-Olivencia et al. 2009 | Adult | 11 | 32 | 180.9 | 18.7 | - | - | - | - |
| Gomez-Olivencia et al. 2009 | Adult | 12 | 32 | 106.1 | 31.8 | - | - | - | - |

**Table R4.** Measured values of adult **male** rib sizes, mm.

| Author | Age | Rib | n | *l* | SD | *h* | SD | *w* | SD |
| --- | --- | --- | --- | --- | --- | --- | --- | --- | --- |
| Subit et al. 2015 | 20-84 | 1 | 36 | 133 | - | - | - | - | - |
| Gomez-Olivencia et al. 2010 | Adult | 1 | 32 | - | - | 17 | 1.6 | 5 | 0.6 |
| Gomez-Olivencia et al. 2009 | Adult | 1 | 32 | - | - | 19 | 2.7 | 6.8 | 0.9 |
| Elrod et al. 2012 | 53±15 | 1 | 37 | 135 | 16 | - | - | - | - |
| Elrod et al. 2012 | 46±16 | 1 | 28 | 133 | 9.5 | - | - | - | - |
| Subit et al. 2015 | 20-84 | 2 | 36 | 221 | - | - | - | - | - |
| Cirillo and Henneburg, 2012 | 76±9 | 2 | 6 | 245 | 5.3 | 13 | 0.3 | 9.4 | 0.4 |
| Cirillo and Henneburg, 2012 | 78±9 | 2 | 5 | 226 | 5.3 | 11 | 0.4 | 8.4 | 0.4 |
| Gomez-Olivencia et al. 2010 | Adult | 2 | 33 | - | - | 12 | 1.6 |  |  |
| Gomez-Olivencia et al. 2009 | Adult | 2 | 32 | - | - | 15 | 1.5 | 6.8 | 1.3 |
| Subit et al. 2015 | 20-84 | 3 | 36 | 270 | - | - | - | - | - |
| Chapman et al. 2017 | 30±6 | 3 | 7 | 261 | 6 | - | - | - | - |
| Cirillo and Henneburg, 2012 | 76±9 | 3 | 6 | 294 | 4.5 | 15 | 0.3 | 9.8 | 0.3 |
| Gomez-Olivencia et al. 2010 | Adult | 3 | 33 | - | - | 9.3 | 1.2 | 6.4 | 0.9 |
| Gomez-Olivencia et al. 2009 | Adult | 3 | 32 | - | - | 13 | 1.6 | 7.1 | 1 |
| Subit et al. 2015 | 20-84 | 4 | 36 | 298 | - | - | - | - | - |
| Chapman et al. 2017 | 30±6 | 4 | 7 | 289 | 5.2 | - | - | - | - |
| Cirillo and Henneburg, 2012 | 76±9 | 4 | 6 | 333 | 3.9 | 16 | 0.4 | 11 | 0.3 |
| Gomez-Olivencia et al. 2010 | Adult | 4 | 33 | - | - | 10 | 1.3 | 6.5 | 1.1 |
| Gomez-Olivencia et al. 2009 | Adult | 4 | 32 | - | - | 13 | 1.5 | 7.4 | 1.1 |
| Subit et al. 2015 | 20-84 | 5 | 36 | 315 | - | - | - | - | - |
| Chapman et al. 2017 | 30±6 | 5 | 7 | 307 | 8.2 | - | - | - | - |
| Cirillo and Henneburg, 2012 | 76±9 | 5 | 6 | 340 | 3.9 | 17 | 0.5 | 12 | 0.5 |
| Gomez-Olivencia et al. 2010 | Adult | 5 | 33 | - | - | 12 | 1.6 | 6.6 | 0.9 |
| Gomez-Olivencia et al. 2009 | Adult | 5 | 32 | - | - | 13 | 1.6 | 8.2 | 1.2 |
| Subit et al. 2015 | 20-84 | 6 | 36 | 325 | - | - |  | - | - |
| Chapman et al. 2017 | 30±6 | 6 | 7 | 311 | 9.6 | - | - | - | - |
| Cirillo and Henneburg, 2012 | 76±9 | 6 | 6 | 350 | 4.1 | 18 | 0.5 | 13 | 0.4 |
| Gomez-Olivencia et al. 2010 | Adult | 6 | 33 | - | - | 10 | 1.1 | 7.8 | 0.9 |
| Gomez-Olivencia et al. 2009 | Adult | 6 | 32 | - | - | 14 | 1.8 | 86 | 1.1 |
| Subit et al. 2015 | 20-84 | 7 | 36 | 325 | - | - | - | - | - |
| Chapman et al. 2017 | 30±6 | 7 | 7 | 310 | 11 | - | - | - | - |
| Cirillo and Henneburg, 2012 | 76±9 | 7 | 6 | 343 | 4.2 | 19 | 0.4 | 14 | 0.4 |
| Gomez-Olivencia et al. 2010 | Adult | 7 | 33 | - | - | 11 | 1.2 | 8 | 0.9 |
| Gomez-Olivencia et al. 2009 | Adult | 7 | 32 | - | - | 15 | 1.8 | 8.5 | 1.2 |
| Subit et al. 2015 | 20-84 | 8 | 36 | 320 | - | - | - | - | -- |
| Chapman et al. 2017 | 30±6 | 8 | 7 | 299 | 17 | - | - | - |  |
| Cirillo and Henneburg, 2012 | 76±9 | 8 | 6 | 336 | 4.1 | 18 | 0.4 | 14 | 0.5 |
| Gomez-Olivencia et al. 2009 | Adult | 8 | 32 | - | - | 15 | 2.1 | 7.8 | 1.3 |
| Subit et al. 2015 | 20-84 | 9 | 36 | 298 | - | - | - | - | - |
| Chapman et al. 2017 | 30±6 | 9 | 7 | 279 | 22 | - |  | - | - |
| Cirillo and Henneburg, 2012 | 76±9 | 9 | 6 | 311 | 4.3 | 16 | 0.4 | 15 | 0.5 |
| Gomez-Olivencia et al. 2009 | Adult | 9 | 32 | - | - | 14 | 1.7 | 7.3 | 1 |
| Subit et al. 2015 | 20-84 | 10 | 36 | 260 | - | - | - | - | - |
| Cirillo and Henneburg, 2012 | 76±9 | 10 | 6 | 273 | 3.9 | 15 | 0.7 | - | - |
| Gomez-Olivencia et al. 2010 | Adult | 10 | 33 | - | - | 9.6 | 0.9 | 6.5 | 0.8 |
| Gomez-Olivencia et al. 2009 | Adult | 10 | 32 | 253 | 21 | 14 | 1.7 | 6.5 | 1 |
| Continuation | | | | | | | | | |
| Author | Age | Rib | n | *l* | SD | *h* | SD | *w* | SD |
| Gomez-Olivencia et al. 2009 | Adult | 10 | 10 | 249 | 13 | - | - | - | - |
| Gomez-Olivencia et al. 2009 | Adult | 11 | 32 | 198 | 25 | 11 | 1.7 | 5.7 | 1 |
| Gomez-Olivencia et al. 2009 | Adult | 11 | 10 | 199 | 13 | - | - | - | - |
| Gomez-Olivencia et al. 2009 | Adult | 12 | 32 | 113 | 31 | 9.5 | 2 | 4 | 1 |
| Gomez-Olivencia et al. 2009 | Adult | 12 | 10 | 116 | 22 | - | - | - | - |

**Table R5.** Published data on adult ribs cortical thickness for male and female, mm.

| Author | Age | N | M | SD |
| --- | --- | --- | --- | --- |
| Murach et al. 2016 | 18-55 | 30 | 0.75 | 0.25 |
| Kemper et al. 2007 | 42-81 | 6 | 0.83 | 0.26 |
| Mohr et al. 2007 | 64±13 | 8 | 0.6 | 0.36 |
| **Assumed for BPS** | |  | **0.74 (3.8)** | |

**Table R6.** Assumed values of rib-BPSs parameters for adults and 15-Y (M±SD, mm).

| BPS# | Assumed for ribs | Male | | | Female | | |
| --- | --- | --- | --- | --- | --- | --- | --- |
|  |  | *w* | *h* | *l** | *w* | *h* | *l** |
| 1 | R1, R2 | 7±0.98 | 17±2.04 | 186.5 | 6±1.0 | 14±1.9 | 163.0 |
| 2 | R3, R4, R9, R10 | 7±0.98 | 13±1.04 | 289.5 | 6±1.0 | 11±0.9 | 244.3 |
| 3 | R5 – R8 | 8±1.04 | 14±1.96 | 319.0 | 7±0.89 | 13±1.0 | 280.3 |
| 4 | R11 – R12 | 6±1.02 | 11±1.98 | 184.5 | 4±1.0 | 9±0.9 | 143.5 |

*-average for group of ribs

**Analysis of published data on rib microstructures**

Table R7 shows the parameters of trabecular bone (secondary spongiosa) as measured by Byers et al. (2000) along the long axis of 5–7 ribs at a distance of up to 5.4 mm from the growth plate. In the original paper, the individual data for studied person were presented and we average the measured data in age-groups. Thus, for children younger than 10 (inclusive), the estimates of Byers et al. (2000) were taken for the SPSD model; for children of 15 and older, the adult parameters described by Mailhot et al. (2017) were accepted.

**Table R7.** Parameters of the microstructure of pre-adult rib according to Byers et al. (2000) (n=46; averaged by age groups by us)

| Age | n | BV/TV, r.u. | SD BV/TV | Tb.Th, mm | SD Tb.Th |
| --- | --- | --- | --- | --- | --- |
| 0–0.5 | 22 | 0.189 | 0.053 | 0.136 | 0.048 |
| 0.5–3 | 16 | 0.286 | 0.100 | 0.233* | 0.083 |
| >3 | 8 | 0.197 | 0.057 | 0.228* | 0.070 |

*Age-groups 0.5–3 and 3 y are not significantly different (p=0.8) and were combined

**Table R8.** Measured data on trabecular space, mm (Bayer et al. 1997; total n=43)

| Age | Average | SD | Min | Max | N |
| --- | --- | --- | --- | --- | --- |
| 0-0.5 | 0.522 | 0.051 | 0.410 | 0.591 | 19 |
| 0.51-13.5 | 0.505 | 0.071 | 0.342 | 0.600 | 24 |

**Table R9.** Parameters of the microstructure of adult rib according to Mailhot et al. 2016 (n=13; age=25)

| BV/TV, % | SD BV/TV | Tb.Th | SD Tb.Th | Tb.Sp | SD Tb.Sp |
| --- | --- | --- | --- | --- | --- |
| 11.6 | 4.1 | 0.147 | 0.018 | 0.817 | 0.1 |

**Table R10**. Assumed values of rib-BPSs micro-parameters

| Age | BV/TV, r.u.  (min-max) | SD BV/TV | Tb.Th, mm  (min-max) | SD  Tb.Th | Tb.Sp, mm  (min-max) | SD  Tb.Sp |
| --- | --- | --- | --- | --- | --- | --- |
| 0 | 0.189  (0.075-0.28) | 0.053 | 0.136  (0.070-0.250) | 0.048 | 0.522  (0.41-0.591) | 0.051 |
| 1 | 0.286  (0.14-0.44) | 0.100 | 0.231  (0.110-0.460) | 0.078 | 0.505  (0.342-0.600) | 0.071 |
| 5 | 0.197  (0.08-0.26) | 0.057 | 0.231  (0.110-0.460) | 0.078 | 0.505  (0.342-0.600) | 0.071 |
| 10 | 0.197  (0.08-0.26) | 0.057 | 0.231  (0.110-0.460) | 0.078 | 0.505  (0.342-0.600) | 0.071 |
| 15 | 0.116 | 0.041 | 0.147 | 0.018 | 0.817 | 0.1 |
| Adults | 0.116 | 0.041 | 0.147 | 0.018 | 0.817 | 0.1 |

**References for ribs**

[Beresheim AC](https://www.ncbi.nlm.nih.gov/pubmed/?term=Beresheim%20AC%5BAuthor%5D&cauthor=true&cauthor_uid=31729033), [Pfeiffer S](https://www.ncbi.nlm.nih.gov/pubmed/?term=Pfeiffer%20S%5BAuthor%5D&cauthor=true&cauthor_uid=31729033), [Grynpas M](https://www.ncbi.nlm.nih.gov/pubmed/?term=Grynpas%20M%5BAuthor%5D&cauthor=true&cauthor_uid=31729033). Ontogenetic changes to bone microstructure in an archaeologically derived sample of human ribs. [J Anat.](https://www.ncbi.nlm.nih.gov/pubmed/?term=Ontogenetic+changes+to+bone+microstructure+in+an+archaeologically+derived+sample+of+human+ribs) 2019 Nov 15. doi: 10.1111/joa.13116

Byers S, Moore AJ, Byard RW, Fazzalari NL. Quantitative histomorphometric analysis of the human growth plate from birth to adolescence. Bone. 2000; 27 (4): 495–501.

Chapman T, Beyer B, Sholukha V, Semal P, Feipel V, Louryan S, Jan VSS. How different are the Kebara 2 ribs to modern humans? J Anthropol Sci. 2017 Mar 10. doi: 10.4436/JASS.95004.

Child Bones-Alamy. Disarticulated skeleton of child. https://Alamy.com. Stock-photo.

Cirillo J. and Henneberg M. Sequencing human ribs into anatomical order by quantitative multivariate methods. Homo. 2012; 63: 182-201.

Cunningham C, Scheuer L, Black S. Developmental Juvenile Osteology. Second Edition. Elsevier Academic Press. 2016.

Elrod, Paige Whitney, "The potential of the angle of the first rib, head to tubercle, in sexing adult individuals in forensic contexts". LSU Master's Theses. 3714. 2012. Available at http://digitalcommons.lsu.edu/gradschool_theses/3714.

Gómez-Olivencia A, Carretero JM, Lorenzo C, Arsuaga JL, Bermúdez de Castro JM, Carbonell E. The costal skeleton of Homo antecessor: preliminary results. J Hum Evol. 2010; 59(6):620-40.

Gómez-Olivencia A, Eaves-Johnson KL, Franciscus RG, Carretero JM, Arsuaga JL. Kebara 2: new insights regarding the most complete Neandertal thorax. J Hum Evol. 2009; 57(1):75-90. doi: 10.1016/j.jhevol.2009.02.009.

Kemper AR, McNally C, Pullins CA, Freeman LJ, Duma SM, Rouhana SM. The biomechanics of human ribs: material and structural properties from dynamic tension and bending tests. Stapp Car Crash J. 2007; 51:235-273.

Mailhot G, Dion N, Farlay D, Rizzo S, Bureau NJ, Jomphe V, Sankhe S, Boivin G, Lands LC, Ferraro P, Ste-Marie LG. Impaired rib bone mass and quality in end-stage cystic fibrosis patients. Bone. 2017 May; 98:9-17. doi: 10.1016/j.bone.2017.02.007. Epub 2017 Feb 22. PMID: 28254466.

Mohr M, Abrams E, Engel C, Long WB, Bottlang M. Geometry of human ribs pertinent to orthopedic chest-wall reconstruction. J Biomech. 2007;40(6):1310-7. doi: 10.1016/j.jbiomech.2006.05.017. Epub 2006 Jul 10. PMID: 16831441.

Molleson, T. and Cox, M. The Spitalfields Project Volume 2 – The Anthropology – The Middling Sort, Research Report 86. London: Council for British Archaeology. 1993.

Murach M, Bazyk A, Misicka E, Kang Y, Moorhouse K, Agnew A. Utilization of a Novel Method for Measuring Cortical Thickness to Investigate Variation with Age in Male Human Ribs. In: 2016 IRCOBI Conference, Malaga, Spain (09.14.2016-09.16.2016). Available at https://trid.trb.org/view.aspx?id=1426732.

Pfeiffer S. Cortical Bone Histology in Juveniles. https://www.researchgate.net/publication/303179375_Cortical_bone_histology_in_Juveniles

Roberts S, Chen P. On some geometric properties of human ribs. J Biomechanics. 1970; 3:527-545.

Streeter M. Four-Stage Method of Age at Death Estimation for Use in the Subadult Rib Cortex. J Forensic Sci, July 2010; 55(4). doi: 10.1111/j.1556-4029.2010.01396

Subit D, Sandoz B, Choisne J, Amabile C, Vergari C, Skalli W, Laporte S. Rib length variation with age and sex - Measurements from high-resolution low-radiation X-ray images of volunteer subjects. 24th International Technical Conference on the Enhanced Safety of Vehicles. 2015; 15-0313.

1. The most commonly measured parameters: cross-sectional area of the rib, the area of the cortical part, the characteristics of cortical-bone osteons (e.g., Streeter et al. 2010). However, these parameters cannot be used for modeling. There is also an extensive pool of rib-length measurements (data are also not used). [↑](#footnote-ref-1)
