# Supplementary material for: Stochastic parametric skeletal dosimetry model for humans: Anatomical-morphological basis and parameter evaluation
Source: PLoS One. 2025 Jul 2;20(7):e0327156. doi: 10.1371/journal.pone.0327156 (PMC12306906; doi:10.1371/journal.pone.0327156)
Supplement: S5 Tibia — (DOCX) [file pone.0327156.s005.docx]

**TIBIA**

**Pre-adults, analysis of published data on tibia macro-parameters and cortical thickness**

The shape and size of the tibia are significantly dependent on age, Fig. T1 illustrates the age-changes in the period 0–5 years. Tibia segments for children (0-10 years old) were modeled.


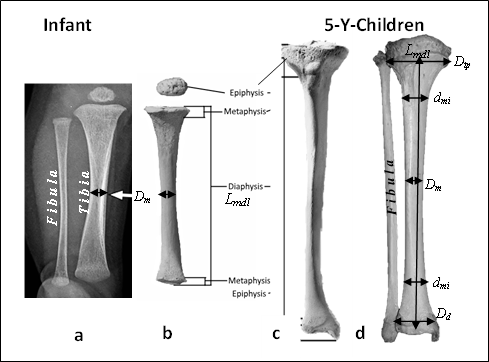


**Fig. T1.** Age-changes in tibia bone: (a) radiograph (x-rays) images (Normal pediatric bone X-ray) of newborn; (b,c) scheme of tibia divisions into diaphysis, metaphysis, and epiphysis of infant and child of 5; note that the diaphysis length (syn. maximal diaphysis length) includes the length of metaphysis (Buikstra and Ubelaker 1994; Meresh 1970); (d) tibia of 5-Y-child based on Schwarz 2007. Letter designations are deciphered in the text.

Main measured parameters described in literature which were collected:

- Maximal diaphysis length *(L_mdl_) -* distance between the proximal and distal growth zone (epiphyseal lines), does not include the epiphysis ossified from separate centers (Fig. T1);
- Outside diameter (*D_m_*) in the mid-point of diaphysis (Fig. T1);
- Cortical thickness (Ct.Th) in the mid-point of diaphysis;
- Sagittal diameter of the proximal epiphysis (*D_sp_*); epiphysis is not modeled but the value of *D_sp_* is close in size to the width of the proximal diaphysis
- Distal epiphyseal breadth (*D_d_*) (Fig. T1); epiphysis is not modeled but the value of *D_sp_* is close in size to the width of the distal diaphysis
- Relative outside diameters (relative to mid-point) at different distances from the distal end of the diaphysis; they were estimated by us with use of the images from Gosman et al. 2013, and used for derivation of absolute values of *d_mi,_* ;i.e., outside diameters in different point *i* of diaphysis (Fig. T2)
- Relative cortical thickness (relative to mid-point) at different distances from the distal end of the diaphysis; they were estimated by us with use of the images from Gosman et al. 2013 (Fig. T2); and used for derivation of absolute values of Ct.Th

Tables F1–F4 present the measured data on tibia macro-parameters and cortical thickness of pre-adults; Table 5 shows the averaged values assumed for modeling.

**Table T1.** Published data on tibia maximal diaphyseal length (*L_mdl_*), mm.

| Author | Age | N | M | SD |
| --- | --- | --- | --- | --- |
| Medvedev et al. 2009 | 0 | 46 | 66.0 | 3.0 |
| Florence et al. 2007 | 0.04 | 13 | 61.6 | 8.7 |
| Miles 1994 | 0 | 36 | 74.7 | - |
| Gindhart et al. 1973 | 0.08 | 264 | 71.7 | 4.7 |
| Maresh et al. 1970 | 0.13 | 128 | 70.6 | 5.0 |
| Florence et al. 2007 | 0.17 | 38 | 69.2 | 4.2 |
| Gindhart et al. 1973 | 0.25 | 216 | 84.9 | 11.2 |
| Maresh et al. 1970 | 0.25 | 123 | 81.4 | 5.0 |
| Florence et al. 2007 | 0.38 | 8 | 75.7 | 6.8 |
| Gindhart et al. 1973 | 0.50 | 308 | 98.2 | 5.2 |
| Maresh et al. 1970 | 0.50 | 145 | 90.0 | 5.3 |
| Florence et al. 2007 | 0.75 | 3 | 104.4 | 5.6 |
| Gindhart et al. 1973 | 0.75 | 217 | 109.8 | 11.2 |
| Gindhart et al. 1973 | 1.00 | 277 | 118.3 | 5.8 |
| Maresh et al. 1970 | 1.00 | 153 | 109.4 | 5.0 |
| Gindhart et al. 1973 | 1.50 | 200 | 134.9 | 6.9 |
| Maresh et al. 1970 | 1.50 | 152 | 125.1 | 5.8 |
| Florence et al. 2007 | 2.00 | 3 | 132.5 | 26.8 |
| Gindhart et al. 1973 | 2.00 | 241 | 149.1 | 7.5 |
| Lopez-Costas et al. 2011 | 2.00 | 13 | 133.9 | 24.5 |
| Maresh et al. 1970 | 2.00 | 152 | 139.2 | 6.5 |
| Gindhart et al. 1973 | 2.50 | 176 | 162.9 | 13.3 |
| Maresh et al. 1970 | 2.50 | 154 | 151.3 | 6.9 |
| Gindhart et al. 1973 | 3.00 | 237 | 173.6 | 9.6 |
| Maresh et al. 1970 | 3.00 | 151 | 162.3 | 8.0 |
| Gindhart et al. 1973 | 3.50 | 168 | 183.9 | 9.8 |
| Maresh et al. 1970 | 3.50 | 151 | 172.0 | 9.3 |
| Gindhart et al. 1973 | 4.00 | 247 | 193.8 | 11.0 |
| Maresh et al. 1970 | 4.00 | 152 | 181.8 | 9.3 |
| Florence et al. 2007 | 4.50 | 2 | 180.0 | 2.9 |
| Gindhart et al. 1973 | 4.50 | 162 | 203.6 | 11.1 |
| Maresh et al. 1970 | 4.50 | 149 | 191.4 | 9.9 |
| Gindhart et al. 1973 | 5.00 | 234 | 212.8 | 12.1 |
| Maresh et al. 1970 | 5.00 | 157 | 200.7 | 10.7 |
| Gindhart et al. 1973 | 5.50 | 149 | 222.8 | 12.8 |
| Maresh et al. 1970 | 5.50 | 147 | 209.1 | 11.6 |
| Gindhart et al. 1973 | 6.00 | 275 | 232.1 | 14.1 |
| Maresh et al. 1970 | 6.00 | 146 | 218.2 | 11.3 |
| Gindhart et al. 1973 | 6.50 | 199 | 241.0 | 15.4 |
| Maresh et al. 1970 | 6.50 | 153 | 227.1 | 12.6 |
| Gindhart et al. 1973 | 7.00 | 263 | 250.4 | 15.6 |
| Lopez-Costas et al. 2011 | 7.00 | 17 | 200.2 | 26.8 |
| Maresh et al. 1970 | 7.00 | 157 | 235.2 | 13.0 |
| Gindhart et al. 1973 | 7.50 | 202 | 260.0 | 17.3 |
| Maresh et al. 1970 | 7.50 | 159 | 243.7 | 13.7 |
| Gindhart et al. 1973 | 8.00 | 256 | 269.4 | 18.0 |
| Continuation | | | | |
| Author | Age | n | M | SD |
| Maresh et al. 1970 | 8.00 | 155 | 252.5 | 14.3 |
| Gindhart et al. 1973 | 8.50 | 193 | 277.7 | 18.5 |
| Maresh et al. 1970 | 8.50 | 154 | 259.9 | 14.0 |
| Gindhart et al. 1973 | 9.00 | 244 | 289.4 | 19.5 |
| Maresh et al. 1970 | 9.00 | 159 | 267.1 | 15.3 |
| Gindhart et al. 1973 | 9.50 | 166 | 297.4 | 19.4 |
| Maresh et al. 1970 | 9.50 | 161 | 276.8 | 16.6 |
| Gindhart et al. 1973 | 10.00 | 225 | 306.8 | 19.2 |
| Maresh et al. 1970 | 10.00 | 161 | 284.6 | 16.8 |
| Florence et al. 2007 | 10.50 | 2 | 252.1 | 1.8 |
| Gindhart et al. 1973 | 10.50 | 29 | 313.1 | 17.1 |
| Maresh et al. 1970 | 10.50 | 151 | 292.2 | 18.3 |
| Gindhart et al. 1973 | 11.00 | 180 | 323.0 | 19.2 |
| Maresh et al. 1970 | 11.00 | 151 | 299.8 | 18.1 |
| Gindhart et al. 1973 | 11.50 | 23 | 326.6 | 18.9 |
| Maresh et al. 1970 | 11.50 | 151 | 308.7 | 19.0 |
| Gindhart et al. 1973 | 12.00 | 128 | 336.6 | 19.8 |
| Lopez-Costas et al. 2011 | 12.00 | 19 | 250.5 | 30.8 |
| Gindhart et al. 1973 | 13.00 | 95 | 302.3 | 23.9 |
| Gindhart et al. 1973 | 14.00 | 64 | 362.5 | 23.8 |
| Gindhart et al. 1973 | 15.00 | 41 | 372.3 | 32.6 |
| Gindhart et al. 1973 | 16.00 | 42 | 384.3 | 24.8 |
| Gindhart et al. 1973 | 17.00 | 33 | 393.0 | 25.9 |
| Lopez-Costas et al. 2011 | 17.00 | 8 | 304.4 | 31.3 |

**Table T2.** Published data on tibia outside diameter (*D_m_*) and cortical thickness (Ct.Th) in the mid-point of diaphysis (mm)

| Author | Age | n | *D_m_* | | Ct.Th | |
| --- | --- | --- | --- | --- | --- | --- |
|  |  |  | M | SD | M | SD |
| Suominen et al. 2015 | 0 | 10 | 7.6 | 0.6 | - | - |
| Dhavale 2016 | 0 | 3 | - | - | 2.5 | 0.3 |
| Florence et al. 2007 | 0.17 | 38 | 6.1 | 0.5 | 1.8 | 0.3 |
| Florence et al. 2007 | 0.38 | 8 | 6.8 | 0.7 | 1.7 | 0.5 |
| Suominen et al. 2015 | 0.5 | 10 | 9.9 | - | - | - |
| Florence et al. 2007 | 0.75 | 3 | 8.8 | 1.0 | 2.7 | 0.2 |
| Dhavale 2016 | 1 | 8 | - | - | 3.3 | 0.5 |
| Florence et al. 2007 | 2 | 3 | 11.5 | 2.4 | 2.8 | 0.15 |
| Suominen et al. 2015 | 3.5 | 10 | 11.6 | 1.0 | - | - |
| Florence et al. 2007 | 4.5 | 2 | 13.4 | 0.5 | 3.7 | 0.47 |
| Dhavale 2016 | 5 | 3 | - | - | 4.1 | 0.8 |
| Florence et al. 2007 | 10.5 | 1 | 19.3 | - | 4.5 |  |
| Kindler et al. 2017 | 11.3 | 630 | - | - | 3.8 | 0.5 |

Farr et al. (2014) describes the Ct.Th measurements performed in distal part of tibia diaphysis (at the level of distal metaphysis); Ct.Th=0.7 ±0.02 (age=12; n=108).

**Table T3.** Published data on tibia distal epiphysis breadth (*D_d_*), and sagittal diameter of the proximal epiphysis (*D_sp_*) according to Lopez-Costas et al. 2011, mm

| Age | *D_d_* | | | *D_sp_* | | |
| --- | --- | --- | --- | --- | --- | --- |
|  | n | M | SD | n | M | SD |
| 2 | 5 | 18.6 | 4.4 | 11 | 16.9 | 3.1 |
| 7 | 12 | 28.3 | 5.4 | 14 | 25.8 | 4.5 |
| 11.3 | 17 | 39.9 | 4.5 | - | - | - |
| 12 | 12 | 38.8 | 4 | 18 | 35.7 | 6.4 |
| 17 | 38 | 43.2 | 2.6 | 39 | 45.3 | 3.2 |
| 22.5 | 72 | 43.5 | 2.3 | 71 | 45.1 | 3.2 |

To assess the transverse (larger) diameter of the proximal tibia end (*D_tp_*, Fig. T1), we used the data on distal end of the femur (bicandilar diameter *D_bc_* Section Femur). These bones form a knee joint. Analysis of the images of the knee joint (posterior views in normal pediatric bone x-ray) showed that the ratio of *D_tp_* and *D_bc_* does not significantly depend on age, and of about 0.8. Table T4 shows the femur measured data described in Section Femur

**Table T4.** Age dependence of femur bicondylar diameter *(D_bc_*).

| Age | Femur *D_bc_* mm | |
| --- | --- | --- |
|  | M | SD |
| 0 | 26.4 | 2.5 |
| 1 | 34 | 4.0 |
| 5 | 68.2 | 4.2 |
| 10 | 78.4 | 5.3 |

**Table T5.** Assumed values of main tibia-parameters used in BPSs modeling, mm.

| Age | *L_mdl_* | | Ct.Th mid-point | | *D_m_* | | *D_d_* | | *D_sp_* | | *D_tp_* | |
| --- | --- | --- | --- | --- | --- | --- | --- | --- | --- | --- | --- | --- |
|  | M | SD | M | SD | M | SD | M | SD | M | SD | M | SD |
| 0 | 74.0 | 6.5 | 1.8 | 0.3 | 6.8 | 0.6 | 14.7 | 3.4 | 13.4 | 2.4 | 21.1 | 2.0 |
| 1 | 111.3 | 7.1 | 3.0 | 0.4 | 8.8 | 1.0 | 16.8 | 3.9 | 15.2 | 2.7 | 27.2 | 3.2 |
| 5 | 204.7 | 10.7 | 3.9 | 0.7 | 13.4 | 0.5 | 24.0 | 5.4 | 22.3 | 4.5 | 54.6 | 3.4 |
| 10 | 291.8 | 16.7 | 4.2 | 0.5 | 19.3 | 1.1 | 34.5 | 4 | 31.5 | 6.4 | 62.7 | 4.2 |

As seen, we have data on Ct.Th and outside- diameter only for mid-point of diaphysis. An exception is the data on distal part of tibia diaphysis in children of 12 years measured by Farr et al. (2014). For modeling, the estimates in other diaphysis points for reference ages are necessary. For this purpose, we have used the data of Gosman et al. (2013) who evaluated the diameters of the diaphysis and the Ct.Th in different points of growing tibia. Fig. T2 presents the scheme of tibia cross-sections in reference points located from distal to proximal end.

**Fig. T2.** Schematic view on tibia-diaphysis cross-sections from distal to proximal end illustrating the changes in tibia diameter and cortical thickness (Ct.Th). Numbers indicate the position of cross-sections relative to maximal diaphyseal length (diaphyseal/metaphyseal length). Based on Gosman et al. (2013) with modifications.

Since the bone increases in length with age, the position of the points was determined relative to the total length of the diaphysis. Based on Gosman et al. (2013) data, we calculated the relative values (relative to the central point of 50%) of *d_mi_* and *Ct.Th_i_* for persons of studied ages (Table T6, T7) and absolute value of the parameters (Table T8, T9). Data of Farr et al. (2014) refer to points located near the epiphyseal line, i.e. more distally than referent point 16.4% (about 3–5%). According to these data, Ct.Th_distal_/Ct.Th_50%_ = 0.16, this ratio also was used to estimate Ct.Th in distal and proximal tibia for other ages (Table T8).

**Table T6.** Relative values (relative mid-point 50%) of tibia outside-diameters (*d_mi_*) in reference points from Fig. T2 (relative units, based on Gosman et al. 2013).

| Age | 16.4% | | 32% | | **Mid-point 50%** | 66.5% | | 83.2% | |
| --- | --- | --- | --- | --- | --- | --- | --- | --- | --- |
|  | *d_1_* | *d_2_* | *d_1_* | *d_2_* | ***D_m_*** | *d_1_* | *d_2_* | *d_1_* | *d_2_* |
| 0–1.9 | 1.3 | 1.2 | 1.0 | 1.0 | **1.0** | 0.8 | 0.9 | 1.3 | 1.3 |
| 2–4.9 | 1.3 | 1.1 | 1.1 | 1.1 | **1.0** | 1.1 | 1.1 | 1.5 | 1.3 |
| 5–8.9 | 1.4 | 1.0 | 1.1 | 0.9 | **1.0** | 1.2 | 1.1 | 1.5 | 1.2 |
| 9–13.7 | 1.3 | 0.9 | 1.0 | 0.9 | **1.0** | 1.2 | 1.0 | 1.3 | 1.1 |
| 14–17.9 | 1.4 | 1.0 | 1.0 | 0.9 | **1.0** | 1.2 | 1.1 | 1.4 | 1.4 |

*d_1_* – anterior-posterior direction; *d_2_* – lateral-medial direction; in mid-point *Dm_1_= Dm_2_*

**Table T7.** Relative values (relative mid-point 50% of corresponding age) of cortical thickness of tibia- diaphysis in position shown in Fig. T2, based on Gosman et al. 2013 (relative units).

| Age | 16.4% | 33.1% | **Mid-point 50%** | 66.5% | 83.2% |
| --- | --- | --- | --- | --- | --- |
| 0–1.9 | 0.5 | 0.8 | **1.0** | 0.9 | 0.6 |
| 2–4.9 | 0.4 | 0.7 | **1.0** | 0.8 | 0.6 |
| 5–8.9 | 0.6 | 0.8 | **1.0** | 0.9 | 0.7 |
| 9–13.7 | 0.6 | 0.9 | **1.0** | 0.8 | 0.7 |
| 14–17.9 | 0.4 | 0.9 | **1.0** | 0.9 | 0.5 |

**Table T8.** Calculated values of tibia outside-diameters (*d_mi_* mm) in reference points from Fig. T2 assumed for reference ages

| Age | 16.4% | | 33.1% | | **Mid-point 50%** | 66.5% | | 83.2% | |
| --- | --- | --- | --- | --- | --- | --- | --- | --- | --- |
|  | *d_1_* | *d_2_* | *d_1_* | *d_2_* | ***D_m_*** | *d_1_* | *d_2_* | *d_1_* | *d_2_* |
| 0 | 8.6 | 8.3 | 6.9 | 6.6 | 6.8 | 5.6 | 5.8 | 9.1 | 8.7 |
| 1 | 11.2 | 10.7 | 8.9 | 8.6 | 8.8 | 7.3 | 7.5 | 11.8 | 11.3 |
| 5 | 17.9 | 14.4 | 14.4 | 14.4 | 13.4 | 15.4 | 14.4 | 20.7 | 17.9 |
| 10 | 18.7 | 13.4 | 14.6 | 12.2 | 13.4 | 16.0 | 14.8 | 19.6 | 16.4 |

*d_1_* – anterior-posterior direction; *d_2_* – lateral-medial direction;

**Table T9**. Calculated values of tibia cortical thickness (*Ct.Th_i_* mm) in reference points from Fig. T2 assumed for reference ages

| Age | Distal and proximal ends* | 16.4% | 33.1% | **Mid-point 50%** | 66.5% | 83.2% |
| --- | --- | --- | --- | --- | --- | --- |
| 0 | 0.3 | 0.9 | 1.4 | **1.8** | 1.5 | 1.1 |
| 1 | 0.51 | 1.6 | 2.3 | **3.0** | 2.6 | 1.8 |
| 5 | 0.70 | 1.7 | 2.8 | **3.9** | 3.0 | 2.3 |
| 10 | 0.70 | 2.3 | 3.1 | **3.9** | 3.4 | 2.6 |

* based on estimated ratio for 12-Y-child (Farr et al. 2014)

Uncertainty values of *Ct.Th_i_* and *d_mi_* estimates were taken the same as for mid-point of diaphysis (in terms of CV).

**Tibia 0**–**1 Y, segmentation and estimation of model parameters**

Tibia has the shape of a complex tube; at the ends, the diameters are much larger than in the middle. Three BPSs were used for description of infant tibia (Fig. T3), Table T10 summarizes the approaches to BPS parameter derivation.

**Fig. T3.** Infant tibia, (a – c) radiograph (x-rays) images from (Normal pediatric bone X-ray): (a) newborn tibia, anterior view; (b) lateral view; (с) posterior view on 1-Y-tibula; (d) stylized models (BPSs).

BPS 1 (shaft) was described by round cylinder of height *h_m_* and diameter *d_m_*; cortical layer is located on the walls of the cylinder;

BPS 2 (proximal end) was described by the truncated cone of height *h_pe_*; round base of diameter *d_m_* and elliptical base of diameters *d_1p_* and d*_2p;_* cortical layer is located on the walls of the cylinder;

BPS 3 (distal end) was described by the truncated cone of height *h_de_*; round base of diameter *d_m_* and larger round base of diameter *d_d_*; cortical layer is located on the walls of the cylinder.

**Table T10.** BPS parameter assumed for femur of 0–1 Y (mm).

| BPS | Para-meter | Rationale | 0 Y | | 1 Y | |
| --- | --- | --- | --- | --- | --- | --- |
|  |  |  | M | SD | M | SD |
| #1 | *Ct.Th* | Four point average (16%, 33%, 50%, 66.5%)^a^ | 1.4 | 0.2 | 2.3 | 0.3 |
| #2, #3 | *Ct.Th* | Based on ratio Ct.Th_dist_/Ct.Th_50%_ | 0.3 | 0.05 | 0.51 | 0.07 |
| #1 | *h_m_* | 45% maximal diaphysis length *L_mdl_* | 33.3 | 2.9 | 50.0 | 3.2 |
| #1, #2, #3 | *d_m_* | Four point average (16%, 33%, 50%, 66.5%)^a^ | 6.9 | 1.9 | 9.0 | 1.2 |
| #2 | *h_p_* | 35% maximal diaphysis length *L_mdl_* | 25.9 | 2.3 | 38.9 | 2.5 |
| #2 | *d_1p_* | *= D_tp_* ^b^ | 21.1 | 2.0 | 27.2 | 3.2 |
| #2 | d*_2p_* | Measured data = *D_sp_* | 13.4 | 2.4 | 15.2 | 2.7 |
| #3 | *h_d_* | 20% maximal diaphysis length *L_mdl_* | 14.8 | 1.3 | 22.3 | 1.4 |
| #3 | *d_d_* | Measured data= *D_d_* | 14.7 | 3.4 | 16.8 | 3.9 |

a- Ct.Th and *d_m_* values in reference-point are presented in the Table T8 and Table T9; b- based on the ratio of transverse (bicondylar) diameter of distal femur and proximal tibia (Table T5).

**Tibia 5-Y, segmentation and estimation of model parameters**

Three BPSs were used for description of 5-Y-tibia (Fig. T4). For the convenience of calculations, the segmentation tibia on the diaphyseal length (L_mdl_) was done according to the reference point from Fig. T2. Table T11 summarizes the approaches to BPS parameter derivation.

**Fig. T4.** Tibia of 5-Y: (a,b) radiograph (x-rays) images from (normal pediatric bone x-ray): (a) posterior view; (b) lateral view; (c) stylized models (BPSs).

BPS 1 (shaft) was described by round cylinder of height *h_m_* and diameter *d_m_*; cortical layer is located on the walls of the cylinder;

BPS 2 (proximal end) was described by the truncated cone of height *h_pe_*; round base of diameter *d_m_* and elliptical base of diameters *d_1p_* and d*_2p_*; cortical layer is located on the walls of the cylinder;

BPS 3 (distal end) was described by the truncated cone of height *h_de_*; round base of diameter *d_m_* and larger round base of diameter *d_d_*; cortical layer is located on the walls of the cylinder.

**Table T11.** BPS parameter assumed for tibia of 5-Y (mm).

| BPS | Parameter | Rationale | M | SD |
| --- | --- | --- | --- | --- |
| #1 | *Ct.Th* | Four point average (16%, 33%, 50%, 66.5%)^a^ | 2.9 | 0.5 |
| #2, #3 | *Ct.Th* | Based on ratio Ct.Th_dist_/Ct.Th_50%_ | 0.66 | 0.12 |
| #1 | *h_m_* | 67% maximal diaphysis length *L_mdl_* ^b^ | 136.7 | 7.1 |
| #1, #2, #3 | *d_m_* | Four point average (16%, 33%, 50%, 66.5%)^a^ | 15.4 | 0.6 |
| #2 | *h_p_* | 17% maximal diaphysis length *L_mdl_* ^b^ | 34.4 | 1.8 |
| #2 | *d_1p_* | *= D_tp_* ^c^ | 54.6 | 3.4 |
| #2 | d*_2p_* | = *D_sp_* | 15.2 | 2.7 |
| #3 | *h_d_* | 16% maximal diaphysis length *L_mdl_* ^b^ | 33.6 | 1.8 |
| #3 | *d_d_* | = *D_d_* | 24.0 | 5.4 |

a- Ct.Th and *d_m_* values in reference-point are presented in the Table T8 and Table T9; b- For the convenience of calculations, segmentation of the tibia on the diaphyseal length (L_mdl_) was done according to the reference point from Fig. 2; c- based on the ratio of transverse (bicondylar) diameter of distal femur and proximal tibia (Table T5).

**Tibia 10-Y, segmentation and estimation of model parameters**

Compared to the age of 5 years, the 10-Y tibia body (shaft) is not modeled (does not contain AM). The remaining two segments are modeled in the same way as for age 5-Y-tibia (Fig. T5). Table T12 summarizes the approaches to BPS parameter derivation.

**Fig. T5.** Tibia of 10-Y: (a,b) radiograph (x-rays) images from (Normal pediatric bone X-ray): (a) posterior view; (b) lateral view; (c) stylized models (BPSs).

BPS 1 (proximal end) was described by the truncated cone of height *h_pe_*; round base of diameter *d_m_* and elliptical base of diameters *d_1p_* and d*_2p_*; cortical layer is located on the walls of the cylinder;

BPS 2 (distal end) was described by the truncated cone of height *h_de_*; round base of diameter *d_m_* and larger round base of diameter *d_d_*; cortical layer is located on the walls of the cylinder.

**Table T12.** BPS parameter assumed for tibia of 10-Y (mm).

| BPS | Parameter | Rationale | M | SD |
| --- | --- | --- | --- | --- |
| #1,#2 | *Ct.Th* | Based on ratio Ct.Th_dist_/Ct.Th_50%_ | 0.71 | 0.08 |
| #1, #2, | *d_m_* | Four point average (16%, 33%, 50%, 66.5%)^a^ | 20.7 | 1.2 |
| #1 | *h_p_* | 17% maximal diaphysis length *L_mdl_* ^b^ | 49.0 | 2.8 |
| #1 | *d_1p_* | *= D_tp_* ^c^ | 62.7 | 4.2 |
| #1 | d*_2p_* | = *D_sp_* | 31.5 | 6.4 |
| #2 | *h_d_* | 16% maximal diaphysis length *L_mdl_* ^b^ | 47.9 | 2.7 |
| #2 | *d_d_* | = *D_d_* | 34.5 | 4.0 |

a- *d_m_* values in reference-point are presented in the Table T8 and Table T9; b- For the convenience of calculations, segmentation of the tibia on the diaphyseal length (*L_mdl_*) was done according to the reference point. from Fig. 2; c- based on the ratio of transverse (bicondylar) diameter of distal femur and proximal tibia (Table T5)

**Analysis of published data on tibia microstructures**

**Table T13.** Published data on microstructure of proximal tibia for pre-adults according to Gosman and Ketcham (2009) and Ding et al. (2012).

| Author | Individual code* | Age | BV/TV | Tb.Th |
| --- | --- | --- | --- | --- |
| G | 10/72 | 0.1 | 0.375 | 0.067 |
| G | 14B/72 | 0.1 | 0.42 | 0.075 |
| G | 9/72 | 0.25 | 0.368 | 0.082 |
| G | 5/71 | 0.25 | 0.399 | 0.083 |
| G | 8/72 | 0.4 | 0.269 | 0.075 |
| G | 15AB/73 | 0.4 | 0.397 | 0.066 |
| G | 15A1B/73 | 0.4 | 0.397 | 0.067 |
| G | 12/73 | 0.4 | 0.271 | 0.083 |
| G | 5/72 | 0.4 | 0.238 | 0.077 |
| G | 7/73 | 0.7 | 0.237 | 0.079 |
| G | 8/73 | 0.9 | 0.22 | 0.089 |
| G | 4/72 | 1.3 | 0.193 | 0.08 |
| G | 9/73 | 1.3 | 0.177 | 0.079 |
| G | 4/73 | 1.3 | 0.16 | 0.085 |
| G | 8/76 | 1.3 | 0.227 | 0.095 |
| G | 7/76 | 1.3 | 0.196 | 0.09 |
| G | 15/72 | 1.3 | 0.231 | 0.104 |
| G | 3/72 | 2.1 | 0.196 | 0.091 |
| G | 6/80 | 2.1 | 0.256 | 0.097 |
| G | 14/74 | 2.75 | 0.142 | 0.088 |
| G | 6/71 | 4.7 | 0.191 | 0.095 |
| G | 13/72 | 6.8 | 0.261 | 0.127 |
| G | 2/73 | 6.8 | 0.222 | 0.121 |
| G | 7/80 | 9.8 | 0.31 | 0.151 |
| G | 1/81 | 9.8 | 0.228 | 0.132 |
| G | SM3 | 9.8 | 0.198 | 0.124 |
| G | 15/74 | 9.8 | 0.239 | 0.132 |
| G | 9/75 | 14.3 | 0,255 | 0,248 |
| G | 7/81 | 14.3 | 0.302 | 0.208 |
| G | 9/77 | 14.3 | 0.262 | 0.201 |
| G | SM18 | 16.9 | 0.278 | 0.212 |
| G | SM16 | 16.9 | 0.258 | 0.253 |
| G | SM9B | 16.9 | 0.212 | 0.179 |
| G | 474 | 19 | 0.244 | 0.202 |
| G | 3A/76 | 21 | 0.235 | 0.257 |
| G | 9/74 | 24 | 0.218 | 0.221 |
| D (n=6) | - | 9–17 | 0.20  (±0.049) | 0.187  (±0.036) |

* original burial number from Gosman and Ketcham (2009) - G; data of Ding et al. (2012) -D, average (±SD)

Parameters for the distal part. Data for the distal portion of tibia that met the analysis conditions were found only for adults (Table T14). A comparison of microparameters of the distal and proximal sections of the tibia in three populations was presented by Saers et al. (2016). In all cases, within the same population, no significant differences were found between the proximal and distal ends in terms of BV/TV and Tb.Th. The averaged data on the distal and proximal end also do not significantly differ. Thus, for the distal part, the same parameters are accepted as for the proximal.

**Table T14.** Parameters of trabecular microstructure of adult tibia.

| Reference | n | Age | BV/TV, r.u. | SD | Tb.Th, mm | SD, mm |
| --- | --- | --- | --- | --- | --- | --- |
| Distal part | | | | | | |
| Saers 2016 (Black) | 16 | 21–41 | 0.346 | 0.07 | 0.469 | 0.104 |
| Saers 2016 (Farm) | 16 | 21–41 | 0.301 | 0.051 | 0.371 | 0.056 |
| Saers 2016 (Kerma) | 20 | 21–41 | 0.173 | 0.041 | 0.207 | 0.025 |
| Kirchir 2015 | 38 | 18–24 | 0.23 | 0.04 | - | - |
| Proximal part | | | | | | |
| Saers 2016 (Farm) | 19 | 21–41 | 0.33 | 0.067 | 0.353 | 0.047 |
| Saers 2016 (Kerma) | 18 | 21–41 | 0.218 | 0.042 | 0.225 | 0.025 |
| Saers 2016 (Black) | 16 | 21–41 | 0.417 | 0.079 | 0.467 | 0.096 |
| Ding 2012 | 9 | 18–24 | 0.212 | 0.061 | 0.169 | 0.03 |
| Ding 2012 | 8 | 25–30 | 0.231 | 0.063 | 0.18 | 0.034 |

Data analysis on trabecular space showed; there is only data for the age group of 9–17 years (Ding et al. 2017; n=6):

Tb.Sp=0.735 mm; SD=0.084 mm; Min (-2*SD) = 0.567; Max(+2*SD)= 0.903

To obtain data for the newborn and children of the younger age group, the dynamics of age-related changes is assumed to be similar to that of the hip and shoulder. For the hip and shoulder, an increase in Tb.Sp was observed in the age from the newborn to 1 year (about 1.5 times) after which the parameter did not change significantly. The maximum scatter of data was observed at the age of 0–1 year, however, at older ages, SD was lower for these bones by only 5–10%. Thus, it is assumed that in a newborn:

Tb.Sp=0.490 mm; SD=0.11 mm; Min (M-2*SD) = 0.272; Max (M+2*SD)= 0.708

For other ages, values are taken to be equal for a group of 9–17 years.

Table T15 shows the values accepted for use in the model for the distal and proximal parts.

**Table T15.** Summary on trabecular microparameters assumed in SPSD-model for tibia.

| Age | BV/TV  (min–max) | SD | Tb.Th, mm  (min–max) | SD, mm | Tb.Sp, mm  (min–max) | SD, mm |
| --- | --- | --- | --- | --- | --- | --- |
| 0 | 0.348  (0.238–0.42) | 0.069 | 0.075  (0.066–0.083) | 0.007 | 0.49  (0.306–0.674) | 0.109 |
| 1 | 0.202  (0.16–0.237) | 0.033 | 0.089  (0.079–0.104) | 0.008 | 0.735  (0.551–0.919) | 0.084 |
| 5 | 0.248  (0.198–0.31) | 0.036 | 0.126  (0.095–0.151) | 0.017 | 0.735  (0.567–0.903) | 0.084 |
| 10 | 0.248  (0.198–0.31) | 0.036 | 0.212  (0.179–0.253) | 0.028 | 0.735  (0.567–0.903) | 0.084 |

**References for tibia**

Buikstra JE, Ubelaker D. Standards for data collection from human skeletal remains. Fayetteville, Arkansas: Arkansas archeological survey research series. 1994; 44.

Dhavale N, Halcrow SE, Buckley HR, Tayles N, Domett KM, Gray AR. Linear and appositional growth in infants and children from the prehistoric settlement of Ban Non Wat, Northeast Thailand: Evaluating biological responses to agricultural intensification in Southeast Asia, Journal of Archaeological Science: Reports. 2017; 11: 435–446, ISSN 2352-409.

Ding M, Lin X, Liu W. [Three-dimensional morphometric properties of rod- and plate-like trabeculae in adolescent cancellous bone.](https://www.ncbi.nlm.nih.gov/pubmed/29662776) J Orthop Translat. 2017 Nov 13; 12:26–35. doi: 10.1016/j.jot.2017.10.001. eCollection 2018 Jan.

Farr JN, Khosla S. Skeletal changes through the lifespan–from growth to senescence. Nat Rev Endocrinol. 2015 Sep;11(9):513–21. doi: 10.1038/nrendo. 2015.89. Epub 2015 Jun 2. Review. PubMed PMID: 26032105; PubMed Central PMCID: PMC4822419.

Florence JL. Linear and cortical bone dimensions as indicators of health status in subadults from the Milwaukee County Poor Farm Cemetery. Dissertation. University of Colorado at Denver. 2007.

Gindhart PS. Growth Standards for the Tibia and Radius in Children Aged One Month through Eighteen Years. Am. J. Phys. Anthrop. 1973; 39: 41–48.

Gosman JH, Ketcham RA. Patterns in ontogeny of human trabecular bone from SunWatch Village in the Prehistoric Ohio Valley: general features of microarchitectural change. Am J Phys Anthropol. 2009 Mar;138(3):318–32. doi:10.1002/ajpa.20931. PubMed PMID: 18785633.

Kindler JM, Pollock NK, Laing EM, et al. Insulin Resistance and the IGF-I-Cortical Bone Relationship in Children Ages 9 to 13 Years. J Bone Miner Res. 2017;32(7):1537-1545. doi:10.1002/jbmr.3132

Lopez-Costas O, Rissech C, Trancho G, Turbón D. Postnatal ontogenesis of the tibia. Implications for age and sex estimation. Forensic Sci Int. 2012 Jan 10;214(1-3): 207.e1–11. doi: 10.1016/j.forsciint.2011.07.038. Epub 2011 Aug 20. PubMed PMID: 21862250.

Maresh MM. Measurements from roentgenograms. In: Human Growth and Development (RW. McCammon, Ed.) Springfield, IL: Charles C. Thomas. 1970. 157–200.

Medvedev MV Ed. Ultrasonic Fetometry: Reference Tables and Nomograms Ed. 8th, rev. Moscow: Real time Publisher. 2009; 19–24 (in Russian).

Miles AEW. Growth Curves of Immature Bones from a Scottish Island Population of Sixteenth to mid-Nineteenth Century: Limb-bone Diaphyses and Some Bones of the Hand and Foot. International Journal of Osteoarcheology. 1994; 4:121–136.

Normal pediatric bone X-ray, available in <https://radiologykey.com/normal-growth-normal-development-and-congenital-disorders/>; <https://bonexray.com/>; <http://bones.getthediagnosis.org/>; <http://bonepit.com/>; https://radiopaedia.org/cases/

[Saers JP](https://www.ncbi.nlm.nih.gov/pubmed/?term=Saers%20JP%5BAuthor%5D&cauthor=true&cauthor_uid=27457548), [Cazorla-Bak Y](https://www.ncbi.nlm.nih.gov/pubmed/?term=Cazorla-Bak%20Y%5BAuthor%5D&cauthor=true&cauthor_uid=27457548), [Shaw CN](https://www.ncbi.nlm.nih.gov/pubmed/?term=Shaw%20CN%5BAuthor%5D&cauthor=true&cauthor_uid=27457548), [Stock JT](https://www.ncbi.nlm.nih.gov/pubmed/?term=Stock%20JT%5BAuthor%5D&cauthor=true&cauthor_uid=27457548), [Ryan TM](https://www.ncbi.nlm.nih.gov/pubmed/?term=Ryan%20TM%5BAuthor%5D&cauthor=true&cauthor_uid=27457548). Trabecular bone structural variation throughout the human lower limb. [J Hum Evol.](https://www.ncbi.nlm.nih.gov/pubmed/?term=Trabecular+bone+structural+variation+throughout+the+human+lower+limb) 2016 Aug;97:97–108. doi: 10.1016/j.jhevol.2016.05.012. Epub 2016 Jul 13

Schwarz JH. Skeleton Keys: An Introduction to Human Skeletal Morphology, Development and Analysis, 2nd Edition. Oxford University Press: Oxford. 2007; 402 pp. <https://global.oup.com/us/companion.websites/9780195188592/student/part_two/Subadult_Specimens/Subadult_B/>

Suominen PK, Nurmi E, Lauerma K. Intraosseous access in neonates and infants: risk of severe complications - a case report. Acta Anaesthesiol Scand. 2015 Nov;59(10):1389–93. doi: 10.1111/aas.12602. Epub 2015 Aug 24. PubMed PMID: 26300243
