# Supplementary material for: Stochastic parametric skeletal dosimetry model for humans: Anatomical-morphological basis and parameter evaluation
Source: PLoS One. 2025 Jul 2;20(7):e0327156. doi: 10.1371/journal.pone.0327156 (PMC12306906; doi:10.1371/journal.pone.0327156)
Supplement: S6 Vertebra — (DOCX) [file pone.0327156.s006.docx]

**vertebrae**

**Adults, analysis of published data on vertebra macro-parameters and cortical thickness**

**Lumbar vertebrae**

The human spine contains five lumbar vertebrae. The typical vertebra (Fig. 1) consists of: *body*; *lamina* attached to the body with *two pedicles*; unpaired *spinous process*, paired *transverse processes*, paired superior and inferior *articular processes* (Fig. 1а).


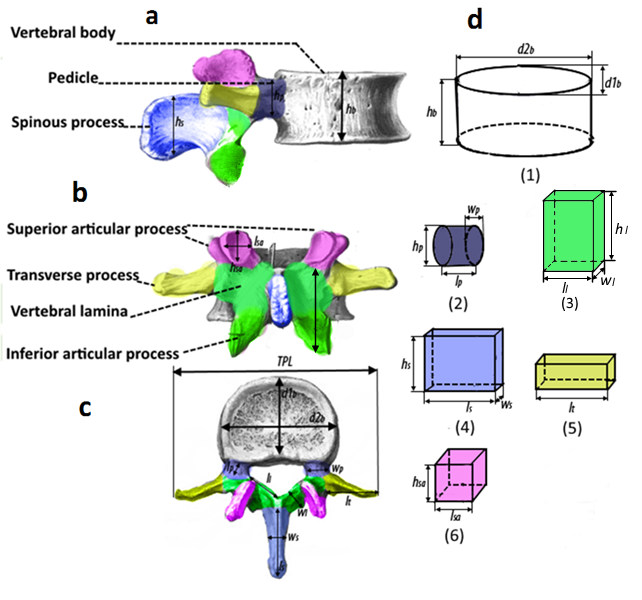


**Fig. V1.** Lumbar vertebra (a) lateral view; (b) posterior view (c) superior view, (d) stylized models (BPS) describing vertebra segments: (1) body; (2) pedicle; (3) lamina and inferior articular process; (4) spinous process, (5) transverse process, (6) superior articular process. In (c), TPL denotes the distance between the ends of the left and right transverse processes (TPLs). Other notations are described in the text that follows.

The following seven segments were allocated for reference lumbar vertebra (see also Fig. 1):

1. *Body* of vertebra was described as elliptic *cylinder* of major axis *d_1b_ (*sagittal body diameter) and *d_2b_* (transverse body diameter) and height *h_b_.* Parameters *d_1b_* and *d_2b_* were fixed based on the measurements at the mid body height; *h_b_* was assigned as a body-average value; cortical layer is located on the walls of the cylinder.
2. *Pedicle* was approximated by elliptic *cylinder* of major axis *d_1b_* and *d_2b_* and height *h_b_.* (distance from the vertebra body to lamina); cortical layer is located on the walls of the cylinder.
3. It was assumed that *Lamina and inferior articular process* consists of two equal boxes of *l_l_-* length of lamina (distance along the upper end of lamina between the pedicolaminar junction and the root of the spinous process); *h_l_* and *w_l_;* cortical layer is located on four lateral sides.
4. *Spinous process* was described by a box of *l_s_* – length; *h_s_* – height; *w_s_* – width; the cortical layer is located on four lateral sides and one end face not adjacent to laminae.
5. *Transverse process* was described by a box of *l_t_* – length; *h_t_* – height; *w_t_* – width; the cortical layer is located on four lateral sides and one end face not adjacent to laminae.
6. *Superior articular process* was described by a box of *l_sa_* – length; *h_sa_*– height; *w_sa_*– width, the cortical layer is located on four lateral sides and one end face not adjacent to laminae.

The measured parameters were averaged by us over the entire set of lumbar vertebrae to receive the BPS-parameters for reference vertebra. Only the vertebral bodies and spinous process have sex-specific dimensions. These parameters were evaluated separately for male and female.

**Table V1.** Body of lumbar vertebra, data for adult **male**, mm

| Author | Age | N | L1 | SD | L2 | SD | L3 | SD | L4 | SD | L5 | SD |  |
| --- | --- | --- | --- | --- | --- | --- | --- | --- | --- | --- | --- | --- | --- |
| Sagittal body diameter *d_1b_* | | | | | | | | | | | | | |
| Alam et al. 2014 | 18-60 | 33 | 31 | - | 33 | - | 33 | - | 34 | - | 33 | - |  |
| Al-Anazi 2009 | Adults | 170 | - | - | - | - | 34 | - | 34 | - | 35 | - |  |
| Gocmen-mas et al. 2010 | 22-49 | 13 | 35 | 2 | 36 | 3 | 37 | 3 | 33 | 3 | 40 | 3 |  |
| Jadhav et al. 2013 | 25-50 | 44 | 30 | 2 | 30 | 2 | 31 | 2 | 32 | 2 | 32 | 2 |  |
| Junno et al. 2009 | 42-54 | 60 | - | - | - | - | - | - | 38 | 3 | - | - |  |
| van Schaik et al. 1985 | 15-75 | 38 | - | - | - | - | 35 | - | 37 | - | 37 | - |  |
| Zhou et al. 2000 | 50±14 | 55 | - | - | - | - | 34 | 3 | 35 | 3 | 36 | 3 |  |
| Transverse body diameter *d_2b_* | | | | | | | | | | | | | |
| Alam et al. 2014 | 18-60 | 33 | 43 | - | 43 | - | 45 | - | 47 | - | 47 | - |  |
| Gocmen-mas et al. 2010 | 22-49 | 13 | 49 | 4 | 52 | 3 | 54 | 4 | 57 | 3 | 57 | 4 |  |
| Jadhav et al. 2013 | 25-50 | 44 | 36 | 2 | 38 | 2 | 40 | 2 | 42 | 2 | 45 | 3 |  |
| Junno et al. 2009 | 42-54 | 60 | - | - | - | - | - | - | 49 | 4 | - | - |  |
| van Schaik et al. 1985 | 15-75 | 38 | - | - | - | - | 46 | - | 47 | - | 50 | - |  |
| Zhou et al. 2000 | 50±14 | 55 | - | - | - | - | 50 | 3 | 53 | 4 | 56 | 5 |  |
| Height *h_b_* | | | | | | | | | | | | | |
| Alam et al. 2014 | 18-60 | 33 | 25 | - | 27 | - | 27 | - | 27 | - | 28 | - |  |
| Gilad and Nissan 1985 | 20-38 | 154 | 25 | 2 | 27 | 2 | 28 | 2 | 27 | 2 | 28 | 2 |  |
| Gocmen-mas et al. 2010 | 22-49 | 13 | 23 | 1 | 24 | 2 | 24 | 2 | 22 | 1 | 23 | 2 |  |
| Hermann et al. 1993 | 22-79 | 40 | 31 | 2 | 34 | 3 | 35 | 2 | 36 | 3 | 36 | 3 |  |
| Junno et al. 2009 | 42-54 | 60 | - | - | - | - | - | - | 31 | 2 | - | - |  |
| Zhou et al. 2000 | 50±14 | 55 | - | - | - | - | 31 | 2 | 30 | 2 | 29 | 2 |  |
| **Average values assumed for BPS (CV%)** | | ***d_1b_*=35.0 (6); *d_2b_*=47.0 (5); *h_b_*=27.0 (7)** | | | | | | | | | | | |

**Table V2.** Body of lumbar vertebra, data for adult **female**, mm.

| Author | Age | N | Vertebra number, M±SD | | | | | | | | | | |
| --- | --- | --- | --- | --- | --- | --- | --- | --- | --- | --- | --- | --- | --- |
|  |  |  | L1 | | SD | L2 | SD | L3 | SD | L4 | SD | L5 | SD |
| Sagittal body diameter *d_1b_* | | | | | | | | | | | | | |
| Alam et al. 2014 | 18-60 | 16 | 29 | | - | 30 | - | 30 | - | 31 | - | 32 | - |
| Gocmen-mas et al. 2010 | 22-49 | 12 | 34 | | 2 | 35 | 3 | 36 | 2 | 38 | 3 | 39 | 2 |
| Jadhav et al. 2013 | 25-50 | 40 | 27 | | 2 | 28 | 3 | 28 | 3 | 29 | 3 | 30 | 3 |
| Junno et al. 2009 | 42-54 | 31 | - | | - | - | - | - | - | 34 | 2 | - | - |
| van Schaik et al. 1985 | 15-75 | 39 | - | | - | - | - | 33 | - | 33 | - | 34 | - |
| Zhou et al. 2000 | 50±14 | 71 | - | | - | - | - | 32 | 3 | 34 | 3 | 34 | 3 |
| Transverse body diameter *d_2b_* | | | | | | | | | | | | | |
| Alam et al. 2014 | 18-60 | 16 | 39 | | - | 40 | - | 42 | - | 43 | - | 45 | - |
| Gocmen-mas et al. 2010 | 22-49 | 12 | 48 | | 5 | 52 | 4 | 54 | 3 | 56 | 4 | 57 | 3 |
| Jadhav et al. 2013 | 25-50 | 40 | 33 | | 3 | 35 | 3 | 37 | 3 | 40 | 3 | 42 | 3 |
| Junno et al. 2009 | 42-54 | 31 | - | | - | - | - | - | - | 44 | 3 | - | - |
| van Schaik et al. 1985 | 15-75 | 39 | - | | - | - | - | 41 | - | 43 | - | 45 | - |
| Zhou et al. 2000 | 50±14 | 71 | - | | - | - | - | 45 | 4 | 49 | 4 | 45 | 5 |
| Height *h_b_* | | | | | | | | | | | | | |
| Alam et al. 2014 | 18-60 | 16 | 24 | | - | 25 | - | 27 | - | 27 | - | 24 | - |
| Gilad and Nissan 1985 | 20-38 | 1154 | 25 | | 2 | 27 | 2 | 28 | 2 | 27 | - | 28 | - |
| Gocmen-mas et al. 2010 | 22-49 | 12 | 23 | | 1 | 24 | 2 | 23 | 3 | 23 | 2 | 23 | 2 |
| Hermann et al. 1993 | 22-80 | 73 | 31 | | 2 | 32 | 2 | 32 | 2 | 33 | 2 | 32 | 3 |
| Junno et al. 2009 | 42-54 | 31 | - | | - | - | - | - | - | 29 | 2 | - | - |
| Zhou et al. 2000 | 50±14 | 71 | - | | - | - | - | 29 | 2 | 28 | 2 | 25 | 2 |
| **Average values assumed for BPS (CV%)** | | | | ***d_1b_=*32 (8)*; d_2b_=*43 (8)*; h_b_=* 27 (7)** | | | | | | | | | |

*d_1b_-*distance at the medial line of the vertebral body from the anterior face to the posterior face

*d_2b_-*distance between the two lateral faces of the vertebral body at the medial portion of the body

*h_b_-*distance between the superior and inferior borders of the vertebral body at the medial line running through the anterior face

**Table V3.** Spinous process of lumbar vertebra, data for adult **male** (M± SD, mm).

| Author | Age (range) | N | P | Vertebra number | | | | |
| --- | --- | --- | --- | --- | --- | --- | --- | --- |
|  |  |  |  | L1 | L2 | L3 | L4 | L5 |
| Cai et al. 2015 | Adults | 30 | *h_s_* | 22±3 | 24±4 | 25±6 | 25±4 | 21±4 |
|  |  |  | *w_s_* |  | 6.0±2 | 6.1±2 | 7.0±3 | 6.5±3 |
| Badr El Dine et al. 2014 | 38 (26-45) | 54 | *h_s_* | 18±2 | - | - | - | - |
| Aylott et al. 2012 | 53 (16-91) | 111 | *h_s_* | 26±4 | 27±3 | 27±3 | 24±3 | 20±4 |
| Wolf et al. 2001 (m+f)* | Adults | 55 | *w_s_* | 5+1.1 | 4.3±1.3 | 4.7±1.2 | 5.3±1.4 | 5.8±1.4 |
|  |  |  | *l_s_* | 30+3.7 | 31.5±4.6 | 33.5±5.7 | 32.8±5.3 | 26±5.7 |
| **Average values assumed for BPS (CV%)** | | | | ***l_s_*=31.0 (6); *h_s_*=24.0 (13); *w_s_*= 6.0 (22)** | | | | |

* data for both sexes, these data were also included in the female sample in addition to data obtained from exclusive female samples

*w_s_*-thickness of spinous process (distance between two lateral sides of the process, measured at the middle)

*l_s_*-length (distance between lamina and most dorsal point of spinous process)

*h_s_*-spinous process height (from inferior to superior point)

**Table V4.** Spinous process of lumbar vertebra, data for adult **female**, mm.

| Author | Age (range) | N | Parameter | | Vertebra | M | SD |
| --- | --- | --- | --- | --- | --- | --- | --- |
| Cai et al. 2015 | Adults | 22 | *h_s_* | | L4 | 19 | 3 |
|  |  |  | *w_s_* | | L4 | 6 | 1 |
| Aylott et al. 2012 | 53 (16-91) | 89 | *h_s_* | | L4 | 21 | 3 |
| Wolf et al. 2001 (m+f) | Adults | 55 | *l_s_* | | L3 | 31 | 5 |
| **Average values assumed for BPS (CV%)** | | | | ***l_s_*=31 (16)4; *h_s_*=20 (15); *w_s_*= 6 (17)** | | | |

**Table V5.** Vertebra lamina and inferior articular process for combined samples of adult male and female, published data (mean±STD, mm).

| Author | Age | n | *h_l_* | | *w_l_* | | *l_l_* | |
| --- | --- | --- | --- | --- | --- | --- | --- | --- |
|  |  |  | M | SD | M | SD | M | SD |
| **Xu et al. 1999a** | **Adults** | **37** | **20.5** | **2.0** | **12.7** | **1.7** | **4.1** | **0.7** |

*h_l_*–median laminar height measured from the superior margin of laminato the inferior margin of inferior articular process

*w_l_*-measured from the interior-anterior aspect at the level equidistant from the superior and inferior laminar margins. Each lamina was measured from a line connecting the medial borders of the superior and inferior articular facets to the midline of the interior aspect of the spinous process

*l_l_*-laminar thickness

**Table V6.** Maximal distance between transverse process ends of lumbar vertebra (TPL), data for adult males and females, mm

| Author | Age (range) | n | L1 | L2 | | L3 | L4 | L5 |
| --- | --- | --- | --- | --- | --- | --- | --- | --- |
| Wolf et al. 2001 | 60 (20-90) | 55 | 82 | 80 | | 89 | 91 | 94 |
| Busscher et al. 2009 | 72 (55-84) | 6 | 78 | 82 | | 90 | 88 | 94 |
| Zhou et al. 2000 | 50±4 | 55 | - | - | | 96 | 94 | 96 |
| **Average values assumed for calculation (CV%)** | | | | | **91 (7)** | | | |
| **Length of transverse process assumed for BPS (CV%)** | | | | | ***l_t_*= *(TPL-d_b2_ )/2=(91-41)/2*=25 (20)** | | | |

Height of transverse process assumed for BPS, mm (CV%) ht=0.3×hb=0.3×27=8.1 (20)

Width of transverse process assumed for BPS (CV%) wt=1.5×h=1.5×8.1=12.1

**Table V7.** Vertebra superior-articular-process macro-parameters for combined samples of adult male and female, published data (mean± STD, mm).

| Author | Age (range) | N | P | Vertebra number | | | | |
| --- | --- | --- | --- | --- | --- | --- | --- | --- |
|  |  |  |  | L1 | L2 | L3 | L4 | L5 |
| Patel et al. 2007 | Adults | 40 | *l_sa_* | 11±2 | 12±2 | 13±2 | 13±2 | 13±2 |
|  |  |  | *h_sa_* | 11±2 | 13±2 | 14±2 | 15±1 | 15±2 |
| Panjabi et al. 1993 | Adults | 8 | *l_sa_* | 12 | 15 | 16 | 17 | 17 |
|  |  |  | *h_sa_* | 10 | 11 | 14 | 15 | 16 |
| Gupta et al. 2015 | Adults | 30 | *l_sa_* | 12±1 | 14±2 | 15±2 | 15±2 | 15±2 |
|  |  |  | *h_sa_* | 12±1 | 13±2 | 14±2 | 15±2 | 15±2 |
| SUSHPU-collection | Adults | 5 | *w_sa_* |  | 11±1 |  | 12±2 | 13±2 |
| **Average values assumed for BPS (CV%)** | | | | ***l_sa_* =15.0 (13); *h_sa_*=14.0 (14); *w_sa_*=12.0 (17)** | | | | |

*l_sa_*-the vertical distance between the most superior point to most inferior point on the facet at their center

*w_sa_-*the horizontal distance in the center of the facet

**Table V8.** Vertebra pedicle parameters for combined samples of adult male and female, published data (mean±STD, mm).

| Author | Age (range) | N | P | | Vertebra number | | | | |
| --- | --- | --- | --- | --- | --- | --- | --- | --- | --- |
|  |  |  |  |  | L1 | L2 | L3 | L4 | L5 |
| Gaivorovsky 2009 | 41(22-60) | 50 | *h_p_* | | 5±1 | 5±1 | 5±1 | 4±1 | 3±1 |
|  |  |  | *d_1p_* | | 16±1 | 16±1 | 16±2 | 15±2 | 16±2 |
|  |  |  | *d_2p_* | | 8±2 | 8±1 | 9±2 | 11±2 | 16±4 |
| Seema et al. 2016 | 35(20-49) | 75 | *d_1p_* | | 18±1 | 19±1 | 18±1 | 18±1 | 18±1 |
|  |  |  | *d_2p_* | | 9±1 | 10±1 | 12±1 | 13±1 | 14±1 |
|  | 55(50-60) | 25 | *d_1p_* | | 17±1 | 18±1 | 17±1 | 18±1 | 18±1 |
|  |  |  | *d_2p_* | | 9±1 | 10±1 | - | 14±1 | 14±1 |
| Badr El Dine et al. 2014 | 38(26-45) | 54 | *d_1p_* | | 22±2 | - | - | - | - |
|  |  |  | *d_2p_* | | 9±1 | - | - | - | - |
| Bhaumik et al. 2013 | 40(20-60) | 500 | *d_1p_* | | 16±4 | 17±4 | 17±5 | 18±6 | 18±8 |
|  |  |  | *d_2p_* | | 9±3 | 10±3 | 12±3 | 14±4 | 18±5 |
| Singel et al. 2004 | Adults | 45 | *d_1p_* | | 15±4 | 15±5 | 15±4 | 14±4 | 13±6 |
|  |  |  | *d_2p_* | | 8±7 | 9±7 | 10±7 | 14±7 | 18±8 |
| Razo et al. 2016 | 33±9 | 50 | *d_1p_* | | 17±1 | 15±2 | 15±2 | 14±1 | 14±1 |
|  |  |  | *d_2p_* | | 9±2 | 10±1 | 10±1 | 12±2 | 16±2 |
|  | 61±1 | 50 | *d_1p_* | | 17±2 | 16±1 | 15±1 | 14±2 | 14±4 |
|  |  |  | *d_2p_* | | 8±1 | 8±1 | 10±1 | 12±2 | 15±3 |
| Zhou et al. 2000 | 50±1 | 55 | *d_1p_* | | - | - | 15±2 | 15±2 | 15±2 |
|  |  |  | *d_2p_* | | - | - | 11±2 | 13±2 | 18±3 |
| Alam et al. 2014 | 37(18-60) | 33 | *d_1p_* | | 13 | 13 | 12 | 12 | 11 |
|  |  |  | *d_2p_* | | 6 | 7 | 11 | 11 | 14 |
| **Average values assumed for BPS (CV%)** | | | | ***h_p_*=5.0 (40); *d_1p_*=18.0 (6); *d_2p_*=12.0 (8)** | | | | | |

*d_1p_-* vertical diameter of the pedicle

*d_2p_-* horizontal diameter of the pedicle

*h_p_-* distance between body and lamina

**Table V9.** Cortical thickness of vertebral body and pedicle for combined samples of adult male and female (mean± STD, mm)

| Author | Age (range) | | N | Vertebra number | | | Ct.Th |
| --- | --- | --- | --- | --- | --- | --- | --- |
| Vertebra body | | | | | | | |
| Ritzel et al. 1997 | 42 | (17-90) | 26 | L1-L5 | | 0.29±0.015 | |
| Fazzalari et al. 2006 | 59 | (20-94) | 26 | L1 | | 0.481±0.285 | |
|  | 59 | (20-94) | 27 | L2 | | 0.2±0.164 | |
|  |  |  |  | L3 | | 0.317±0.19 | |
|  |  |  |  | L4 | | 0.577±0.322 | |
|  |  |  |  | L5 | | 0.515±0.269 | |
| Silva et al. 1994 | 45 | (40-49) | 16 | L1 | | 0.366 | |
| Moussa 2008 | 38 | (30-45) | 4 | L1-l5 | | 0.488±0.053 | |
| **Average for BPS (CV%)** | | | | | | **0.4 (50)** | |
| Vertebra pedicle | | | | | | | |
| Chawla K et al. 2012 | 50 | (30-70) | 14 | L1 –L5 | 1.125±0.325 | | |
| Defino and Vendrame 2007 | 74 | (27-88) | 10 | L1 –L5 | 0.935±0.37 | | |
| **Average used for BPSs: superior process, lamina and inferior articular processes (CV%)** | | | | | | **1.04 (34)** | |

**Thoracic vertebrae**

The human spine contains 12 thoracic vertebrae. The structure of the thoracic vertebra is similar to that of the lumbar. The typical vertebra (Fig. 1) consists of: *body*; *lamina* attached to the body with *two pedicle*; unpaired *spinous process*, paired *transverse processes*, paired superior and inferior *articular processes*. The following seven segments were allocated for thoracic vertebra (see also Fig. 2.2). The difference from the lumbar vertebra is that the superior articular process and the lamina are modeled as one segment:


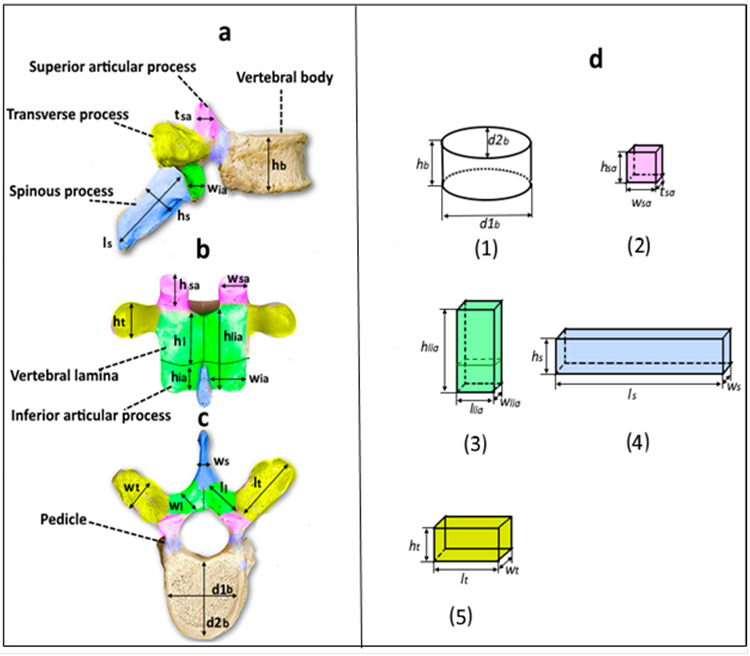


**Fig. V2.** Thoracic vertebra (a) lateral view, (b) posterior view; (c) superior view; (d) stylized models (BPS) described vertebra segments: (1) body; (2) superior articular process; (3) lamina and inferior articular process process; (4) spinous process, (5) transverse process.

1. *Body* of vertebra was described by elliptic *cylinder* of major axis (transverse diameter) *d_1b_*, minor (sagittal) diameter *d_2b_* and height *h_b_.* Parameters *d_1b_* and *d_2b_* were estimated based on the measurements at the mid body height; *h_b_* was assigned as a body-average value; cortical layer is located on the walls of the cylinder.
2. *Superior articular process* was described by a box of length *l_sa_*, height *h_as_* and width *w_sa_*, the cortical layer is located on four lateral sides and one end face not adjacent to laminae.
3. *Lamina* *and inferior articular process* were considered as a single structure consisting of two identical parts (left and right) modeled as two boxes standing one on the other (Fig 2.2 d). Upper (larger) box represents a lamina part (half of lamina), lower (smaller) box represents an inferior articular process. Summary height of BPS *h_lia_*, = *h_l_+ h_ia_*; width *w_lia_* is the average weight of lamina and process; and length *l_lia_* is average length of lamina and process; cortical layer covers the front, back and bottom sides.
4. *Spinous process* was described by a box of length *l_s_*, height *h_s_* and width *w_s_*; the cortical layer is located on four lateral sides and one end face not adjacent to laminae.
5. *Transverse process* was described by a box of length *l_t_*, height *h_t_* and width *w_t_*; the cortical layer is located on four lateral sides and one end face not adjacent to laminae.

The measured parameters were averaged by us over the entire set of thoracic vertebrae to receive the BPS-parameters for reference vertebra. Only the thoracic vertebral bodies have sex-specific dimensions. These parameters were evaluated separately for male and female.

**Table V10.** Body of thoracic vertebra, data for adult **male**, (mean± STD, mm).

| Vertebra number | Hermann et al. 1993; n=40; age =50 (22–79) | Singh et al. 2011; n=81; age= 46 (22–70) | Buck et al. 2002; n=7; Adults |
| --- | --- | --- | --- |
|  | *h_b_* | *d1_b_* | *d2_b_* |
| T1 | - | 25±2 | 29±2 |
| T2 | - | 25±3 | 29±3 |
| T3 | - | 24±2 | 28±4 |
| T4 | 24±2 | 24±2 | 28±4 |
| T5 | 24±2 | 24±2 | 28±4 |
| T6 | 25±2 | 25±2 | 29±3 |
| T7 | 25±2 | 26±2 | 30±2 |
| T8 | 26±2 | 27±2 | 32±3 |
| T9 | 27±2 | 28±3 | 32±3 |
| T10 | 28±3 | 29±3 | 34±2 |
| T11 | 30±2 | 32±4 | 36±3 |
| T12 | 31±2 | 34±3 | 40±5 |
| **Average for BPS (CV%)** | **27.0 (7)** | **28.0 (11)** | **33.0 (9)** |

Comments - *d_1b_-*distance at the medial line of the vertebral body from the anterior face to the posterior face; *d_2b_-*distance between the two lateral faces of the vertebral body at the medial portion of the body; *h_b_-*distance between the superior and inferior borders of the vertebral body at the medial line running through the anterior face

**Table V11.** Body of thoracic vertebra, data for adult **female**, (mean± STD, mm).

| Vertebra number | Hermann et al. 1993 | Singh et al. 2011 | |
| --- | --- | --- | --- |
|  | age =51 (22-80); n=73 | age =46 (22-70); n=46 | |
|  | *h_b_* | *h_b_* | *d1_b_* |
| T1 | - | 14±1 | 23±2 |
| T2 | - | 15±1 | 24±3 |
| T3 | - | 16±1 | 23±3 |
| T4 | 21±1 | 17±1 | 23±1 |
| T5 | 22±1 | 17±1 | 23±2 |
| T6 | 23±1 | 18±1 | 23±2 |
| T7 | 23±2 | 19±1 | 25±2 |
| T8 | 24±2 | 19±1 | 25±2 |
| T9 | 24±2 | 19±1 | 26±2 |
| T10 | 26±2 | 20±1 | 28±2 |
| T11 | 27±2 | 27±1 | 30±2 |
| T12 | 30±2 | 26±1 | 33±3 |

Сomments are the same as for Table V10

**Table V12.** Body of thoracic vertebra, data for adult **female**, (mean± STD, mm)

| Author | Age (range) | N | P | | T6 | T9 | T12 |
| --- | --- | --- | --- | --- | --- | --- | --- |
| Badr El Dine et al. 2014 | Adults | 66 | *d2_b_* | | - | - | 29±1 |
| Taylor et al. 1984 | Adults | 36 | *d2_b_* | | 25 | 29 | - |
|  |  |  | *h_b_* | | 19 | 21 | - |
| **Average for BPS (CV%) according Table 11 and 12** | | | | ***h_b_*=22 (6); *d1_b_*=26 (8); *d2_b_*=29 (6)** | | | |

Сomments are the same as for Table V10

**Table V13.** Lamina measurements for combined samples of adult male and female (T9, mean±STD, mm).

| Author | Age (range) | N | *h_l_* | *w_l_* | *l_l_* |
| --- | --- | --- | --- | --- | --- |
| Been et al. 2010 | Adults | 97 | 22±3 | 3.9±1 | 10.0±2 |
| Xu et al. 1999 | 58 (42–74) | 58 | 22±2 | 3.9±1 | - |
| **Average for BPS (CV%)** | | | **22(11)** | **3.9(25)** | **10.0(20)** |

Comments*: h_l_*–median laminar height measured from the superior margin of laminato the inferior margin of inferior articular process; *w_l_*-measured from the interior-anterior aspect at the level equidistant from the superior and inferior laminar margins. Each lamina was measured from a line connecting the medial borders of the superior and inferior articular facets to the midline of the interior aspect of the spinous process; *l_l_*-laminar thickness

**Table V14.** Inferior articular process measurements for combined samples of adult male and female (T9, mean±STD, mm).

| Author | Age (range) | N | *w_ia_* | *h_ia_* | *l_ia_* |
| --- | --- | --- | --- | --- | --- |
| Ebraheim et al. 1997 | 56 (32–79) | 25 | 11±1 | 11±1 | 4.4±1 |
| Gupta et al. 2015 | Adults | 30 | 12 | 12 | - |
| Panjabi et al. 1993 | 39 (19–59) | 12 | 12±2 | 12±2 | - |
| **Average for BPS (CV%)** | | | **11.3 (14)** | **11.4(12)** | **4.4(11)** |

Comments: *w_ia_-* the horizontal distance on the facet almost in its center on both sides; *h_ia_-* the vertical distance between the most superior to most inferior point on the facet almost at its centre on both sides; *l_ia_-* the horizontal distance between anterior and posterior sides of the facet*,* almost at its center on both sides

**Table V15.** Spinous process measurements averaged by us for T1-T12 for combined samples of adult male and female (mean±STD, mm).

| Author | Age (range) | N | *l_s_* | *h_s_* | *w_s_* |
| --- | --- | --- | --- | --- | --- |
| Tan et al. 2004 | 66 (56–77) | 10 | 50±3 | - | - |
| Panjabi et al. 1991 | 39 (19–59) | 12 | 51±1 | - | - |
| SUSHPU-collection | Adults | 21 | - | 10.3±1.5 | 5.1±1 |
| **Average for BPS (CV%)** | | | **50(4)** | **10.3 (15)** | **5.1(20)** |

Comments: *w_s_*-thickness of spinous process (distance between two lateral sides of the process, measured at the middle); *l_s_*-length (distance between lamina and most dorsal point of spinous process); *h_s_*-spinous process height (from inferior to superior point)

**Table V16.** Transverse process measurements for combined samples of adult male and female, published data (T9, mean±STD, mm).

| Author | Age (range) | N | *w_t_* | *l_t_* | *h_t_* |
| --- | --- | --- | --- | --- | --- |
| Cui et al. 2015 | (38) 24–51 | 45 | 9±1 | 17±1 | 12±1 |
| Singh et al. 2011 | (46) 22–70 | 81 | 12±2 | 18±2 |  |
| **Average for BPS (CV%)** | | | **10.6(13)** | **18.0(11)** | **12.0 (9)** |

Comments: *w_t_-* the distance from the anterior to the posterior edge in the sagittal plane; *l_t_-* the distance from the end of the transverse process to the bottom of the transverse process; *h_t_-* the distance from the superior to the inferior edge in the coronal plane

**Table V17.** Superior articular process measurements for combined samples of adult male and female, published data (T9, mean±STD, mm).

| Author | Age (range) | N | *w_sa_* | *h_sa_* | *l_sa_* |
| --- | --- | --- | --- | --- | --- |
| Ebraheim et al. 1997 | (56) 32–79 | 25 | 11±1 | 11±1 | 4.6±1 |
| Gupta et al. 2013 | Adults | 30 | 10 | 11 | - |
| Panjabi et al. 1993 | (39)19–59 | 12 | 11±1 | 12±2 | - |
| Patel et al. 2007 | Adults | 40 | 9±2 | 9±1 | - |
| **Average for BPS (CV%)** | | | **10.2(11)** | **10.0(15)** | **4.6(10)** |

Comments: *w_sa_-* the horizontal distance on the facet almost in its center on both sides; *h_sa_-* the vertical distance between the most superior to most inferior point on the facet almost at its centre on both sides; *l_sa_-* the horizontal distance between anterior and posterior sides of the facet*,* almost at its center on both sides

**Table V18.** Published data on thoracic vertebra cortical thickness for adult males and females.

| Author | Age | n | Ct.Th | |
| --- | --- | --- | --- | --- |
|  |  |  | M | SD |
| Vertebra body | | | | |
| Fazzalari et al. 2006 | 20–94 | 27 | 0.57 | 0.2 |
| Ritzel et al. 1997 | 17–90 | 26 | 0.24 | 0.014 |
| Fields 2010 | 53–97 | 22 | 0.38 | 0.09 |
| Moussa 2008 | 30–45 | 4 | 0.49 | 0.05 |
| **Average for body-BPS (CV%)** | | | **0.4 (21)** | |
| Pedicle | | | | |
| Zhuang et al. 2012 | 19–59 | 60 | 1.27 | 0.2 |
| **Average used for process- and lamina BPS** | | | **1.27 (16)** | |

**Cervical vertebrae**

Vertebrae C_1_ has unique structures and its lateral masses were modeled instead of the vertebral body (Fig V3). For other vertebrae, the bodies were modeled using only two BPSs: body of C_2_ vertebra and body of C_3_-C_7_ vertebrae (estimated separately for male and female). Parameters of body-BPSs have the same designations as for the lumbar and thoracic vertebrae:

1. *Lateral mass* of C_1_ was described by elliptic cylinder of major axes (diameter) *d1_lm_,* minor diameter *d2_lm_* and height *h_lm_.;* cortical layer covers the lateral surface.
2. *Body* of *C_2_* vertebra was described by elliptic cylinder of major axes (transverse diameter) *d1_b_,* minor (sagittal) diameter *d2_b_* and height *h2_b_.* Parameters *d1_b_* and *d2_b_* are fixed based on the measurements at the mid body height; *h2_b_* was assigned as a body-average value. Cortical layer covers the lateral surface of the cylinder.
3. *Body* of C_3_-C_7_ vertebra was described by elliptic cylinder of major axes (transverse diameter) *d_1b_,* minor (sagittal) diameters *d2_b_* and height *h_b_.* Parameters *d1_b_* and *d2_b_* are fixed based on the measurements at the mid body height; *h_b_* was assigned as a body-average value. Cortical layer is located on the walls of the cylinder.

**
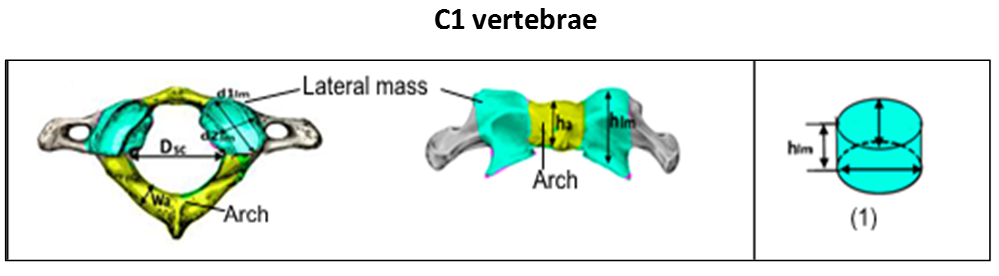
**

**Fig V3.** C1 vertebra (atlas) superior view and posterior view, and BPS describing paired lateral mass

**Table V19.** C1 lateral mass parameters for adult males and females (M±SD, mm).

| Author | Age | N | *d1_lm_* | *d2_lm_* | *h1_lm_* |
| --- | --- | --- | --- | --- | --- |
| Senegul and Kodiglu 2006 | Adults | 40 | 12.0±2.1 | 18.3±3.0 | - |
| Francis 1955 | 25–36 | 244 | 14.0±1.0 | 20.0±2.0 | - |
| Panzer 2006 | Adults | 6 | - | 19.0 | - |
| Buteera and Lukhele 2010 | Adults | 159 | - | - | 12.0±1.0 |
| Gupta et al. 2013 | Adults | 35 | 13.0±2.0 | 19.0 ±2.0 | - |
| Akay et al. 2013 | Adults | 30 | - | - | 18.0±2.0 |
| **Average for BPS (CV%)** | | | **13.3 (13)** | **19 (10)** | **15 (11)** |

Comments: *d1_lm_-* measured as the transverse dimension of articular surface; *d2_lm_-* measured as the A-P dimension of articular surface; *h1_lm_-* the distance between superior and inferior articular surfaces of the vertebra

**Table V20.** Published data on cervical vertebra body C2 sizes for adult males and females.

| Author | Age | N | *d1_b2_* | *d2_b2_* | *h_b2_* |
| --- | --- | --- | --- | --- | --- |
| Gilad and Nissan 1985 | 20–38 | 130 | 17 ±3 | 14±2 | - |
| Panzer 2006 | Adults | 6 | 19 | 14 | 17.5 |
| Senegul and Kodiglu 2006 | Adults | 40 | 22±3 | - | - |
| Panjabi et al. 1991 | 19–59 | 12 | - | 16±1 | 17.5 |
| **Average for BPS (CV%)** | | | **19.2 (13)** | **14.3 (14)** | **17.5 (3)** |

Comments:*d_1b_-*distance at the medial line of the vertebral body from the anterior face to the posterior face; *d_2b_-*distance between the two lateral faces of the vertebral body at the medial portion of the body; *h_b_-*distance between the superior and inferior borders of the vertebral body at the medial line running through the anterior face

**Table V21.** Published data on vertebra body parameters for vertebra C3-C7 for adults, (M±SD, mm).

| Author | Age | N | Sex | P | C3 | C4 | C5 | C6 | C7 |
| --- | --- | --- | --- | --- | --- | --- | --- | --- | --- |
| Bazaldua et al. 2011 | Adults | 30 | M | *h_b_* | 13 | 12 | 11 | 11 | 13 |
|  |  |  | M | *d1_b_* | 15±3 | 16±1 | 17±1 | 17±1 | 17±1 |
|  |  |  | M | *d2_b_* | 19±3 | 21±2 | 21±4 | 22±2 | 23±3 |
| Gilad and Nissan 1985 | 20–38 | 130 | M | *h_b_* | 14±1 | 13±1 | 13±1 | 13±1 | 15±1 |
|  |  |  | M | *d1_b_* | 15±2 | 16±2 | 16±2 | 16±2 | 16±1 |
| Kathole et al. 2012 | 25–40 | 150 | M | *d2_b_* | 17±2 | 17±2 | 17±2 | 17±2 | 17±2 |
|  | 25-40 | 150 | F | *d2_b_* | 16±2 | 15±2 | 15±2 | 15±2 | 16±2 |
| Panjabi et al. 2001 | Adults | 5 | M | *h_b_* | 13±1 | 13±1 | 13±1 | 13±2 | 14±1 |
|  |  |  | M | *d1_b_* | 14±2 | 14±2 | 14±1 | 16±2 | 16±2 |
|  |  |  | M | *d2_b_* | 11±1 | 11±1 | 11±1 | 11±1 | 12±1 |
|  |  | 5 | F | *h_b_* | 13±1 | 13±1 | 13±1 | 13±2 | 14±1 |
|  |  |  | F | *d1_b_* | 16±1 | 16±2 | 18±2 | 17±2 | 20±2 |
|  |  |  | F | *d2_b_* | 15±2 | 15±1 | 16±2 | 16±2 | 15±1 |
| Tan et al. 2004* | 56-77 | 10 | M+F | *d1_b_* | 14±1 | 15±1 | 15±1 | 15±1 | 15±1 |
|  |  |  | M+F | *d2_b_* | 14±1 | 15±1 | 15±1 | 18±1 | 20±1 |
|  |  |  | M+F | *h_b_* | 11±1 | 11±1 | 11±1 | 11±1 | 12±1 |
| Katz et al. 1975 | 18-24 | 31 | F | *h_b_* | 11±1 | 11±1 | 11±1 | 11±1 | 13±1 |
|  |  |  | F | *d1_b_* | 12±1 | 13±1 | 13±1 | 14±1 | 14±1 |
| Zhu et al. 2016 | 19-71 | 68 | F | *d1_b_* | 13±1 | 14±1 | 15±1 | 16±1 | 18±1 |
|  |  |  | F | *d2_b_* | 14±1 | 14±1 | 14±1 | 15±1 | 15±1 |
| **Average for male BPS (CV%)**  **Average for female BPS (CV%)** | | | | ***h_b_* = 13.0 (8); *d1_b_*=16.0 (11); *d2_b_* = 19.0 (14)**  ***h_b_*=12 (6); *d1_b_*=15 (8); *d2_b_*=16 (6)** | | | | | |

Comments are the same as for Table V20

**Table V22**. Published data on cortical thickness of C2-C7 vertebra for adult males and females (mean± SD, mm).

| Author | Age (range) | N | Ct.Th |
| --- | --- | --- | --- |
| Pedicle | | | |
| Chanplakorn et al. 2014 | 49 (18–80) | 74 | 1.3±0.08 |
| **Average used for C1 lateral mass BPS (CV%)** | | | **1.3 (6)** |
| Body | | | |
| Ritzel et al. 1997 | 54 (17–90) | 26 | 0.28±0.02 |
| Amling et al. 1994 | 54 (17–90) | 22 | 0.30 |
| **Average used for body C2 and C3-C7 BPSs (CV%)** | | | **0.29 (7)** |

**Pre-adults, analysis of published data on vertebra macro-parameters and cortical thickness**

The human spine contains seven cervical vertebrae (C_1-7_); 12 thoracic vertebrae (T_1-12_); and five lumbar vertebrae (L_1-5_). The ossification of vertebrae is complex and comes from several centers (Fig. V1). About 30% of the spine is ossified at birth (Dimeglio 1993, 2001). On the roentgenograms, the infant centers of ossification (both vertebral-bodies and vertebral-arches) are in the form of small oval formations (Tager 1983); сartilaginous layers comprise from 30 to 50% of the length of the spine. Further ossification leads to a decrease in the proportion of cartilage.

**Fig. V4.** Ossified parts of typical thoracic vertebra (according to Schaefer et al. 2009 with modification) of 1.5-years child (a): vertebra-body, two half of neural arch and spinous process are indicated; *h_b_* - height of vertebra-body; *d_s_* – sagittal (aterioposterial) diameter of vertebra-body; *d_t_*  - transverse diameter of vertebra-body. (b)- vertebra-body of child of 4 years; (d) – stylized model BPS.

In adults, T- and L- vertebral bodies contain about 8–10 times more AM than other parts of vertebra; for children, this portion is larger. In this regard, for newborne and 1-Y-choldren the only vertebral bodies were modeled (the ossification centers of arches and processes are not modeled), also C1-vertebra (atlant) was not modeled for them. We made models of the transverse and spinous processes of the thoracic and lumbar vertebrae for ages 5-Y, 10-Y, 15-Y.

It should be noted that for 15-year olds the arch (laminae) and superior and inferior processes as well as lateral masses of C1 vertebra were not modeled, but were taken the same as in adults. The corresponding BPSs are described in the section devoted to adults.

**Vertebra body**

The vertebral bodies even within the same part of spine (cervical, thoracic, lumbar) differ from each other. For modeling, the size of the vertebral bodies within one section was averaged (for the cervical region, data on C2–C7 were averaged). Measured parameters are shown in Fig. V1. The bodies of all vertebrae are modeled by a cylinder with an elliptical base of height *h_b_* (height of the vertebral-body), and diameters of *d_t_* (transverse body diameter) and *d_s_* (sagittal or anteroposterior body-diameter) (Fig. V1); cortical layer is located on the walls of the cylinder. Tables V1–V3 present the measured data on vertebra-body macro-parameters of pre-adults.

For V23-V25:

*d_1b_-*distance at the medial line of the vertebral body from the anterior face to the posterior face; *d_2b_-*distance between the two lateral faces of the vertebral body at the medial portion of the body; *h_b_-*distance between the superior and inferior borders of the vertebral body at the medial line running through the anterior face

**Table V23.** Cervical vertebra-body sagittal diameter (*d_s_*) and height (*h_b_*) averaged by us for C2–C7, mm.

| Author | Age | *d_s_* | *SD* | *h_b_* | *SD* |
| --- | --- | --- | --- | --- | --- |
| K | 0 | - | - | 3.7 | 0.3 |
| S | 0 | - | - | 4.5 | 0.4 |
| J | 1 | 9.7 | 0.7 | 5.8 | 1.1 |
| J | 2 | 10.0 | 0.7 | 6.4 | 0.7 |
| J | 3 | 10.6 | 0.7 | 6.7 | 0.7 |
| J | 4 | 11.1 | 0.7 | 7.1 | 1.0 |
| J | 5 | 11.3 | 1.3 | 7.3 | 0.6 |
| J | 6 | 11.6 | 1.0 | 7.6 | 0.6 |
| J | 7 | 13.7 | - | 8.9 | - |
| C | 7 | 12.1 | 0.6 | 7.9 | 0.9 |
| J | 8 | 13.7 | - | 9.0 | - |
| C | 8 | 12.2 | 0.8 | 8.2 | 0.8 |
| J | 9 | 13.8 | - | 9.3 | - |
| C | 9 | 12.6 | 1.0 | 8.2 | 0.9 |
| J | 10 | 14.3 | - | 10.0 | - |
| C | 10 | 13.0 | 0.9 | 8.9 | 1.1 |
| J | 11 | 14.1 | - | 9.9 | - |
| C | 11 | 13.9 | 1.3 | 9.4 | 1.1 |
| J | 12 | 14.5 | - | 10.7 | - |
| C | 12 | 13.9 | 1.1 | 9.9 | 1.2 |
| W | 12.8 | 12.8 | 1.4 | 10.2 | 1.7 |
| J | 13 | 14.7 | - | 11.4 | - |
| C | 13 | 14.7 | 1.3 | 10.8 | 1.2 |
| J | 14 | 14.8 | - | 11.5 | - |
| C | 14 | 14.4 | 1.1 | 11.1 | 1.3 |
| J | 15m | 15.9 | - | 11.7 | - |
| C | 15m | 15.9 | 1.0 | 12.3 | 1.4 |
| J | 15f | 14.6 | - | 12.5 | - |
| C | 15f | 14.4 | 1.3 | 11.0 | 1.3 |

S-Sharma et al. 2017 (n=15); K- Khomutova 2005 (n=32); J- Johnson et al. 2016 (n=128); C- Caldas et al. 2006 (n=1458); W- Wani et al. 2018 (n=86).

Sharma et al. 2017 presents the data on transverse diameter (*d_t_*) for late fetus of third trimester, we combined their data for C3 and C4 for both sexes (n=15) and obtained: *d_t_*= 6.7±0.6 mm

**Table V24.** Thoracic vertebra-body averaged parameters.

| Author | n | Age | *d_s_* | *SD* | *d_t_* | *SD* | *h_b_* | *SD* |
| --- | --- | --- | --- | --- | --- | --- | --- | --- |
| Ponrartana et al. 2015 | 70 | 0 | *-* | *-* | *-* | *-* | 5.1 | 1.1 |
| Comeau et al. 2010 | 1 | 1 | 11.9 | 1.5 | 15.0 | 3.4 | 8.0 | 1.2 |
| Comeau et al. 2010 | 1 | 3 | 15.3 | 2.3 | 17.6 | 3.8 | 10.3 | 1.9 |
| Newman and Gowland 2015 | * | 3 | - | - | - | - | 10.5 | 2 |
| Newman and Gowland 2015 | * | 5 | - | - | - | - | 13 | 4 |
| Comeau et al. 2010 | 1 | 6 | 18.5 | 2.9 | 23.1 | 4.7 | 12.6 | 2.1 |
| Newman and Gowland 2015 | * | 9 | - | - | - | - | 15 | 1 |
| Comeau et al. 2010 | 1 | 10 | 22.0 | 4.7 | 27.3 | 6.5 | 14.3 | 2.4 |
| Newman and Gowland 2015 | * | 16 | - | - | - | - | 18 | 2 |
| Comeau et al. 2010 | 1 | 18 | 26.6 | 5.4 | 29.5 | 5.7 | 21.1 | 3.3 |

* Newman and Gowland 2015 reported total number of thoracic vertebra-body measurements n=62 from two post-medieval sites in England (Bow Baptist, London and Coronation Street, South Shields)

**Table V25.** Lumbar vertebra-body averaged parameters.

| Author | Age  (range) | n | *d_s_* | *SD* | *d_t_* | *SD* | *h_b_* | *SD* |
| --- | --- | --- | --- | --- | --- | --- | --- | --- |
| Ponrartana et al. 2015 | 0 | 70 | *-* | *-* | *-* | *-* | 6.25 | 1.1 |
| Mavrych et al. 2014 | 0 | 23 | 7.7 | 0.4 | 15.0 | 0.7 | 7.1 | 0.4 |
| Mavrych et al. 2014 | 0.5 (0-1) | 12 | 9.3 | 0.7 | 17.6 | 1.3 | 8.6 | 0.5 |
| Mavrych et al. 2014 | 2 (1-3) | 9 | 16.8 | 1.0 | 28.3 | 1.3 | 11.6 | 0.7 |
| Newman and Gowland 2015 | 3 | ^a^ | - | - | - | - | 14 | 2 |
| Mavrych et al. 2014 | 5 (3-7) | 7 | 23.2 | 2.8 | 34.1 | 4.6 | 16.3 | 1.8 |
| Newman and Gowland 2015 | 5 | ^a^ | - | - | - | - | 15 | - |
| Knirsch et al. 2005 | 6-8 | ^b^ | 22.8 | 2.1 |  |  |  |  |
| Newman and Gowland 2015 | 9 | ^a^ | - | - | - | - | 22 | - |
| Knirsch et al. 2005 | 9-11 | ^b^ | 25.5 | 2.7 |  |  |  |  |
| Mavrych et al. 2014 | 10 (8–12) | 6 | 27.3 | 5.7 | 35.9 | 7.7 | 19.2 | 3.4 |
| Knirsch et al. 2005 | 12–14 | ^b^ | 28.0 | 1.1 |  |  |  |  |
| Mavrych et al. 2014 | 14.5 (13–16) | 8 | 30.1 | 2.8 | 43.9 | 4.1 | 24.4 | 2.8 |
| Knirsch et al. 2005 | 15–17 | ^b^ | 28.9 | 0.8 |  |  |  |  |
| Newman and Gowland 2015 | 16 | ^a^ | - | - | - | - | 24 | 3 |
| Mavrych et al. 2014 | 18.5 (17–20) | 14 | 33.6 | 3.0 | 47.7 | 4.1 | 26.7 | 3.1 |

1. Total number of lumbar vertebra-body measurements n=59 from two post-medieval sites in England (Bow Baptist, London and Coronation Street, South Shields);
2. total number of persons measured n=75 of both sexes

As follows from the analysis of published data, a very poor data set concerns the transverse diameter (*d_t_*) of the cervical vertebrae and some parameters for L- T- vertebrae for newborns. In this regard, vertebral images (photographs) were analyzed to determine the ratio *d_s_/d_t_* at different ages (Schaefer and Black 2009; Scheuer and Black 2004; Kosa and Castellana 2004, Schwarz 2007). It was found that the end plate of the C-vertebra-body has an almost round shape. With age, there is not only an increase in all linear-sizes, but also elongation of the C-vertebral body in the transverse direction, the *d_t_/d_s_* ratio increases to 1.3 for 1-Y, and to 1.6 for a 5-Y-old. After this, the C-vertebral body increases to a greater extent in the anteroposterior direction, and in adults this ratio is about 1.2–1.3. As for thoracic vertebra, *d_t_/d_s_* ratio for newborn was estimated as 1.4. Thus, the values of the missing parameters for C- and T-vertebrae were estimated.

Measured data on *vertebra-cortical thickness* were not found for children. Analysis of images has shown that cortical thickness in vertebrae of infants and children is very thin (Kneissel et al. 1997). This thickness is comparable to the thickness of trabeculae. Thus, it is assumed that the entire vertebral body of newborns and 1-Y children is represented by a trabecular bone (spongiosa). For ages 5 and 10 years, Ct.Th corresponds to half the thickness for adults. Therefore:

For L- and T-vertebra of 15-Y, Ct.Th=0.4±0.1 mm (the same as for adults); for 5-Y and 10-Y children, Ct.Th=0.4/2=0.2±0.05 mm;

For C-vertebra of 15-Y, Ct.Th=0.29±0.02 mm (the same as for adults); for children

5-Y and 10-Y children, Ct.Th=0.29/2=0.15±0.01 mm

**Vertebra processes**

*Spinous process* of T- and L-vertebra was described by a box of length *l_s_*, height *h_s_* and width *w_s_*; the cortical layer is located on four lateral sides and one end face not adjacent to laminae.

*Transverse process* of T- and L-vertebra was described by a box of length *l_t_*, height *h_t_* and width *w_t_*; the cortical layer is located on four lateral sides and one end face not adjacent to laminae.

The parameters were derived from Peters et al. (2015, 2021). The authors did not provide the results of dimension measurements, they presented the linear regression equations describing the age dependence of the vertebra sizes. This made it possible to derive the necessary BPS parameters.

**Table V26.** Summary data on vertebra-body parameters and cortical thickness.

| Age | *d_s_* | SD | *d_t_* | SD | *h_b_* | SD | Ct.th | SD |
| --- | --- | --- | --- | --- | --- | --- | --- | --- |
| Cervical- vertebra (C2-C7) | | | | | | | | |
| 0 | 6.9 | 0.5 | 6.5 | 0.5 | 4.1 | 0.3 | 0 | 0 |
| 1 | 9.7 | 0.7 | 12.6 | 0.9 | 5.8 | 0.5 | 0 | 0 |
| 5 | 11.3 | 1.3 | 18.1 | 2.1 | 7.3 | 0.6 | 0.15 | 0.01 |
| 10 | 13.7 | 0.9 | 18.6 | 2.4 | 9.4 | 1.1 | 0.15 | 0.01 |
| 15* | 15.2 | 1.15 | 19.0 | 2.7 | 11.9 | 1.35 | 0.3 | 0.02 |
| Thoracic vertebra body | | | | | | | | |
| 0 | 7.6 | 1.5 | 10.6 | 2.1 | 5.1 | 1 | 0 | 0 |
| 1 | 11.9 | 1.5 | 15 | 3.4 | 8 | 1.2 | 0 | 0 |
| 5 | 17.4 | 3.5 | 21.3 | 4.3 | 11.8 | 2 | 0.2 | 0.05 |
| 10 | 22 | 4.7 | 27.3 | 6.5 | 14.3 | 2.4 | 0.2 | 0.05 |
| 15 | 24.9 | 5 | 28.7 | 5.5 | 18.6 | 2.9 | 0.4 | 0.1 |
| Lumbar vertebra | | | | | | | | |
| 0 | 7.7 | 0.4 | 15 | 0.7 | 7.1 | 0.4 | 0 | 0 |
| 1 | 9.6 | 0.7 | 21 | 1.5 | 9.6 | 0.6 | 0 | 0 |
| 5 | 23.2 | 2.8 | 34.1 | 4.6 | 16.3 | 1.8 | 0.2 | 0.05 |
| 10 | 27.3 | 5.7 | 35.9 | 7.7 | 19.2 | 3.4 | 0.2 | 0.05 |
| 15 | 31.5 | 2.5 | 44.5 | 4.2 | 24.7 | 2.9 | 0.4 | 0.1 |

*- C3-C7, it should be noted that C2 for 15-Y was described by a box; cortical layer covering two sides (ventral and dorsal); see description for adults

*Thoracic vertebra processes*

Table V5 presents the linear regression equations describing age-dependences of T9 vertebra parameters (Peters et al. 2015, 2021). Vertebra T9 was used as reference for all thoracic vertebrae. Table V6 and V7 present the approaches to estimation of model parameters and assumed values for BPSs for T-vertebra processes.

**Table V27.** Equations describing age-dependences of T9 vertebra parameters (Peters et al. 2015, 2021).

|  | Parameter | Sex | Equation |
| --- | --- | --- | --- |
|  | Transverse body diameter *d_t_* | M | 0.981·age+13.834 |
|  |  | F | 0.748·age+14.306 |
|  | Intertransverse process width *ITW (maximal distance between the ends of transverse processes)* | M | 1.806·age+31.507 |
|  |  | F | 1.468·age+32.761 |
|  | Maximal Spinous process length *SPL (distance between the middle point of end-plate of body and the end of spinous process)* | M | 2.022·age+28.96 |
|  |  | F | 1.908·age+30.927 |
|  | Sagittal body diameter *d_s_* | M | 0.893·age+11.7 |
|  |  | F | 0.818·age+10.983 |
|  | Spinal canal depth  *SCD* | M | 0.11·age+14.436 |
|  |  | F | -0.002·age+14.491 |

**Table V28** Approaches to estimation of BPS parameters for T-vertebra processes.

| BPS | Parameter estimates | Rationale |
| --- | --- | --- |
| Transverse process | *l_t_*=(*ITW-d_t_*)/2 | Rough estimate |
|  | *h_t_*=*l_t_**0.7 | Coefficient was derived from the ratio *h_t_/l_t_* for adults |
|  | *w_t_* =*d_t_**0.31 | Coefficient was derived from the ratio *w_t_/d_t_* (determined on the basis of image analysis for children) |
| Spinous process | *l_s_*=*SPL*-1/2**d_s_*-*SCD* | Rough estimate |
|  | *h_s_* =*h_b_**0.5 | Coefficient derived from the ratio *h_s_/h_b_* (determined on the basis of image analysis for children) |
|  | *w_s_* =d_t_*0.181 | Coefficient derived from the ratio *w_s_/d_t_* (determined on the basis of image analysis for children) |

**Table V29** Assumed values for BPS- parameters (T-vertebra processes).

| Transverse process, parameters values, mm | | | | | | |
| --- | --- | --- | --- | --- | --- | --- |
| Age | *l_t_* | SD | *h_t_* | SD | *w_t_* | SD |
| 5-Y | 11 | 2.1 | 7.3 | 1.4 | 5.3 | 1 |
| 10-Y | 12.9 | 2.66 | 8.6 | 1.78 | 7.3 | 1.5 |
| 15-Y | 14.8 | 2.7 | 9.9 | 1.81 | 8.6 | 1.6 |
| Spinous process, parameters values, mm | | | | | | |
| Age | *l_s_* | SD | *h_s_* | SD | *w_s_* | SD |
| 5-Y | 17.4 | 3.3 | 5.9 | 1.12 | 3 | 0.6 |
| 10-Y | 25 | 5.2 | 7.15 | 1.48 | 4.1 | 0.85 |
| 15-Y | 32.5 | 5.9 | 24 | 2.3 | 5.6 | 0.58 |

*Lumbar vertebra processes*

Table V30 presents the linear regression equations describing age-dependences of L3 vertebra parameters (Peters et al. 2015, 2021). Vertebra L3 was used as a reference for all lumbar vertebrae. Approaches for evaluation of model parameters and calculated values are shown in Tables V31 and V32.

**Table V30** Equations describing age-dependence of L3 vertebrae parameters (Peters et al. 2015, 2021).

| Parameter | Sex | Equation |
| --- | --- | --- |
| Transverse body diameter *d_t_* | M | 1.315·age+20.518 |
|  | F | 1.033·age+20.293 |
| Intertransverse process width *ITW (maximal distance between the ends of transverse processes)* | M | 3.128·age+35.03 |
|  | F | 2.531·age+38.269 |
| Maximal Spinous process length *SPL (distance between the middle point of end-plate of body and the end of spinous process)* | M | 2.135·age+34.316 |
|  | F | 1.971·age+35.339 |
| Sagittal body diameter *d_s_* | M | 1.117·age+13.406 |
|  | F | 1.154·age+13.066 |
| Spinal canal depth  *SCD* | M | -0.108·age+17.61 |
|  | F | -0.047·age+16.678 |

**Table V31** Approaches to estimation of BPS parameters for L-vertebra processes.

| BPS | Parameter estimates | Rationale |
| --- | --- | --- |
| Transverse process | *l_t_*=(*ITW-d_t_*)/2 | Rough estimate |
|  | *h_t_*=*l_t_**0.52 | Coefficient was derived from the ratio *h_t_/l_t_* for adults |
|  | *w_t_* =*d_t_**0.15 | Coefficient was derived from the ratio *w_t_/d_t_* (determined on the basis of image analysis for children) |
| Spinous process | *l_s_*=*SPL*-1/2**d_s_*-*SCD* | Rough estimate |
|  | *h_s_* =*h_b_**0.5 | Coefficient derived from the ratio *h_s_/h_b_* (determined on the basis of image analysis for children) |
|  | *w_s_* =d_t_*0.15 | Coefficient derived from the ratio *w_s_/d_t_* (determined on the basis of image analysis for children) |

**Table V32** Assumed values for BPS- parameters (L-vertebra processes).

| Transverse process, parameters values, mm | | | | | | |
| --- | --- | --- | --- | --- | --- | --- |
| Age | *l_t_* | SD | *h_t_* | SD | *w_t_* | SD |
| 5-Y | 12.3 | 1.5 | 8.6 | 1.78 | 7.3 | 1.5 |
| 10-Y | 16.4 | 3.3 | 8.6 | 1.7 | 5.2 | 1.04 |
| 15-Y | 20.5 | 2.2 | 10.7 | 1.03 | 5.6 | 0.58 |
| Spinous process, parameters values, mm | | | | | | |
| Age | *l_s_* | SD | *h_s_* | SD | *w_s_* | SD |
| 5-Y | 13.4 | 1.6 | 14.5 | 1.77 | 5.0 | 0.61 |
| 10-Y | 27.1 | 5.4 | 17.1 | 3.4 | 5.2 | 1.04 |
| 15-Y | 31 | 3 | 24 | 2.3 | 5.6 | 0.58 |

The *thickness of the cortical layer (Ct.Th)* in the transverse and spinous processes is taken to be the same as that of the vertebral body for each specific age

**Analysis of published data on vertebra microstructures**

Tables V33-V34 presents the published data on lumbar and thoracic vertebra. Cervical vertebra parameters for children were derived from data in Table V35-V36. Tb.Sp parameters for cervical vertebrae we take the same as for L- and T- vertebrae.

**Table V33.** Published data on microstructure of thoracic (T) and lumbar (L) vertebrae of pre-adults.

| Author | Age (range) | n | BV/TV | SD | Tb.Th mm | SD mm |
| --- | --- | --- | --- | --- | --- | --- |
| Acquaah et al. 2015 | 0 | 1 | 0.30 | 0.06 | 0.180 | 0.04 |
| Pafundi 2009 | 0* | 1 | 0.455 | 0.053 | - | - |
| Pafundi 2009 | 0** | 1 | 0.598 | 0.088 | - | - |
| Acquaah et al. 2015 | 1,2 | 1 | 0.075 | 0.03 | 0.11 | 0.012 |
| Acquaah et al. 2015 | 2,5 | 1 | 0.075 | 0.029 | 0.130 | 0.012 |
| Kneissel et al. 1997 | 5 (0–9) | 15 | 0.13 | 0.03 | 0.087 | 0.01 |
| Kneissel et al. 1997 | 15 (10–19) | 13 | 0.15 | 0.04 | 0,118 | 0.02 |
| Vijatapalan et al. 2003 | 17 | 1 | 0.101 | - | 0.119 | 0.032 |
| Pafundi 2009 | 18 | 1 | 0.117 | 0.021 | - | - |

* age of 4 days; ** age of 5 days; in the other case 0 means the full-term stillborn.

**Table V34.** Published data on microstructure of thoracic (T) and lumbar (L) vertebrae of adults.

| Author | Vert | Age | n | BV/TV | SD | Tb.Th mm | SD mm | Tb.Sp mm | SD mm |
| --- | --- | --- | --- | --- | --- | --- | --- | --- | --- |
| Cotter et al. 2009 | T | 20–32 | 6 | 0.28 | 0.02 | 0.18 | 0.008 | 0.5 | 0.025 |
| Cotter et al. 2011 | T | 30±7 | 10 | 0.25 | 0.04 | 0.17 | 0.02 | - | - |
| Grote et al. 1995 | T | <45 | 17 | 0.15 | - | - | - | - | - |
| Hazrati et al. 2013 | T | 64-92 | 20 | 0.11 | 0.06 | 0.12 | 0.03 | 0.89 | 0.12 |
| Moussa 2008 | T | 30–45 | 4 | - | - | 0.22 | 0.03 | 0.64 | 0.1 |
| Vijayapalan et al. 2003 | T | 20–49 | 8 | 0.11 | 0.03 | 0.12 | - | - | - |
| Shepelkevich et al. 2011 | L1 | 24 | 1 | 0.07 | - | 0.038 | - | 0.659 | - |
|  | L1 | 33 | 1 | 0.12 | - | 0.045 | - | 0.355 | - |
|  | L1 | 50 | 1 | 0.11 | - | 0.047 | - | 0.423 | - |
| Ostojic et al. 2006 | L1 | 40-59 | 7 | 0.15 | 0.04 | 0.128 | 0.031 | - | - |
| Beuf et al. 2001 | L3 | 22-76 | 14 | 0.21 | 0.05 | 0.143 | 0.01 | 0.574 | 0.123 |
| Chen et al. 2008 | L4 | 57-68 | 9 | 0.16 | 0.02 | 0.119 | 0.014 | 0.722 | 0.076 |
| Fazzalari et al. 2006 | L1 | 20-90 | 26 | - | - | 0.082 | 0.015 | - | - |
|  | L2 |  |  | - | - | 0.08 | 0.016 | - | - |
|  | L3 |  |  | - | - | 0.084 | 0.016 | - | - |
|  | L4 |  |  | - | - | 0.091 | 0.023 | - | - |
|  | L5 |  |  | - | - | 0.09 | 0.03 | - | - |

**Table V35.** Published data on trabecular space for T- and L-vertebra, mm (Kneissel et al. 1997; total n=28).

| Age | Average | SD | Min (-2*SD) | Max(+2*SD) | Count |
| --- | --- | --- | --- | --- | --- |
| 0–9 | 0.60 | 0.120 | 0.36 | 0.84 | 15 |
| 10–19 | 0.65 | 0.154 | 0.34 | 0.96 | 13 |

**Table V36.** Published data on microstructure of cervical vertebrae of pre-adults.

| Author | Age (range) | n | BV/TV | SD | Tb.Th mm | SD mm |
| --- | --- | --- | --- | --- | --- | --- |
| Acquaah et al. 2015^a^ | 0 | 1 | 0.643 | 0.031 | 0.250 | 0.014 |
| Pafundi 2009^b^ | 0 | 1 | 0.538 | 0.054 | - | - |
| Acquaah et al. 2015 ^a^ | 1.5 | 1 | 0.200 | 0.036 | 0.171 | 0.019 |
| Acquaah et al. 2015 ^a^ | 2.5 | 1 | 0.203 | 0.052 | 0.189 | 0.028 |

1. seven vertebrae were measured per person
2. five vertebrae were measured per person

**Table V37.** Published data on microstructure of cervical vertebrae of adults

| Author | Age | n | BV/TV | SD | Tb.Th | SD | Tb.Sp | SD |
| --- | --- | --- | --- | --- | --- | --- | --- | --- |
| Grote et al. 1995 | <45 | 17 | 0.21 | - | - | - | - | - |
| Mulder et al. 2010 | 47–95 | 10 | 0.21 | 0.05 | 0.19 | 0.03 | 0.76 | 0.08 |
| Yan et al. 2011 | 31.8 | 6 | 0.25 | 0.02 | 0.101 | 0.014 | 0.3 | 0.019 |
| Amling et al. 1994 | 17–90 | 22 | 0.2 | - | - | - | - | - |

**Table V38.** Ratio BV/TV and Tb.Th assumed for C - vertebrae in SPSD model.

| Age | BV/TV | SD | Tb.Th mm | SD mm |
| --- | --- | --- | --- | --- |
| 0 | 0.599 | 0.067 | 0.250 | 0.014 |
| 1 | 0.201 | 0.043 | 0.180 | 0.024 |
| 5 | 0.210 | 0.050 | 0.140 | 0.02 |
| 10 | 0.210 | 0.050 | 0.140 | 0.02 |
| 15 | 0.210 | 0.050 | 0.140 | 0.02 |
| Adult | 0.210 | 0.050 | 0.140 | 0.02 |

The value of trabecular separation for adult cervical vertebra vas derived from tablet V37 and equal 0.6±0.06

**Table V39.** Ratio BV/TV, Tb.Th and Tb.Sp assumed for L- and T- vertebrae in SPSD-model.

| Age | BV/TV  (min–max) | SD | Tb.Th, mm  (min–max) | SD, mm | Tb.Sp, mm  (min–max) | SD, mm |
| --- | --- | --- | --- | --- | --- | --- |
| 0 | 0.451  (0.056–0.215) | 0.149 | 0.096  (0.017–0.175) | 0.04 | 0.60  (0.36–0.84) | 0.120 |
| 1 | 0.136  (0.067–0.255) | 0.040 | 0.096  (0.017–0.175) | 0.04 | 0.60  (0.36–0.84) | 0.120 |
| 5 | 0.136  (0.067–0.255) | 0.040 | 0.096  (0.017–0.175) | 0.04 | 0.60  (0.36–0.84) | 0.120 |
| 10 | 0.136  (0.067–0.255) | 0.040 | 0.118  (0.078–0.158) | 0.02 | 0.65  (0.34–0.96) | 0.154 |
| 15 | 0.136  (0.067–0.255) | 0.040 | 0.118  (0.078–0.158) | 0.02 | 0.65  (0.34–0.96) | 0.154 |
| Adult  T-vert | 0.160  (0.03–0.19) | 0.050 | 0.150 | 0.03 | 0.6 | 0.09 |
| Adult  L-vert | 0.150  (0.09-0.22) | 0.030 | 0.100 | 0.013 | 0.6 | 0.09 |

**Reference for vertebrae**

[Acquaah F](https://www.ncbi.nlm.nih.gov/pubmed/?term=Acquaah%20F%5BAuthor%5D&cauthor=true&cauthor_uid=26106365), [Robson Brown KA](https://www.ncbi.nlm.nih.gov/pubmed/?term=Robson%20Brown%20KA%5BAuthor%5D&cauthor=true&cauthor_uid=26106365), [Ahmed F](https://www.ncbi.nlm.nih.gov/pubmed/?term=Ahmed%20F%5BAuthor%5D&cauthor=true&cauthor_uid=26106365), [Jeffery N](https://www.ncbi.nlm.nih.gov/pubmed/?term=Jeffery%20N%5BAuthor%5D&cauthor=true&cauthor_uid=26106365), [Abel RL](https://www.ncbi.nlm.nih.gov/pubmed/?term=Abel%20RL%5BAuthor%5D&cauthor=true&cauthor_uid=26106365). Early Trabecular Development in Human Vertebrae: Overproduction, Constructive Regression, and Refinement. [Front Endocrinol (Lausanne).](https://www.ncbi.nlm.nih.gov/pubmed/?term=Acquaah+vertebra) 2015 May 1;6:67. doi: 10.3389/fendo.2015.00067. eCollection 2015.

Akay A, Rükşen M, Çaǧli MS, Kitiş Ö, Ertürk, M, Zileli, M. An Anatomical and Radiological Study for C1 Lateral Mass Screw Fixation. Journal of Neurological Sciences. 2013; 30: 328–336.

Alam MM, Waqas M, Shallwani H, Javed G. Lumbar Morphometry: A Study of Lumbar Vertebrae from a Pakistani Population Using Computed Tomography Scans. Asian Spine J. 2014; 8(4): 421-426.

Al-Anazi. Sagittal diameter of the lumbar spinal canal and canal/body ratio in normal adult Saudis. Pan Arab J Neurosur. 2009; 13:53-56.

Amling M, Hahn M, Wening VJ, Grote HJ, Delling G. The microarchitecture of the axis as the predisposing factor for fracture of the base of the odontoid process. A histomorphometric analysis of twenty-two autopsy specimens. J Bone Joint Surg Am. 1994;76(12):1840-1846. doi:10.2106/00004623-199412000-00011

Aylott CE, Puna R, Robertson PA, Walker C. Spinous process morphology: the effect of ageing through adulthood on spinous process size and relationship to sagittal alignment. Eur Spine J. 2012; 21(5):1007-1012.

Badr El Dine F, El Shafei MM. Sex determination using anthropometric measurements from multi-slice computed tomography of the 12th thoracic and the first lumbar vertebrae among adult Egyptians. 2014. Egyptian Journal of Forensic Sciences. 2015; 5(3): 82-89. http://dx.doi.org/10.1016/j.ejfs.2014.07.005

Bazaldua CJJ, Gonzalez LA, Gomez SA, Villarreal SA, Velazquez GSE, Sanchez UA, et al. Morphometric study of cervical vertebrae C3–C7 in a population from Northeastern mexico. Int J Morphol. 2011; 29(2):325–330.

Been E, Peleg S, Marom A, Barash A. Morphology and Function of the Lumbar Spine of the Kebara 2 Neandertal. Am J Phys Anthropol. 2010; 142(4):549-557.

Beuf O, Newitt DC, Mosekilde L, Majumdar S. Trabecular structure assessment in lumbar vertebrae specimens using quantitative magnetic resonance imaging and relationship with mechanical competence. J Bone Miner Res. 2001;16(8):1511-1519. doi:10.1359/jbmr.2001.16.8.1511

Bhaumik M, Bapna N, Bhaumik U, Prabhakaran K. Study of transverse and sagittal diameters of lumbar pedicles in relation to trans-pedicular screw fixation using MRI in Rajasthan population. Int J Cur Res Rev. Nov 2013; 05 (21):50-55.

Buck A, Price R, Sweetman I, Oxnard C. An investigation of thoracic and lumbar cancellous vertebral architecture using power-spectral analysis of plain radiographs. Journal of Anatomy. 2002; 200(5):445–456.

Busscher I, Ploegmakers JJ, Verkerke GJ, Veldhuizen AG. Comparative anatomical dimensions of the complete human and porcine spine. Eur Spine J. 2010; 19(7):1104-1114. doi: 10.1007/s00586-010-1326-9. Epub 2010 Feb 26.

Buteera A, Lukhele M. Anatomic study of the atlas for surgical planning of lateral mass screw fixation: is it safe in our population? SA Orthop. J. 2010; 9(4):38–44.

Cai B, Ran B, Li Q, Li ZH, Li FN, Li M, Yan WJ. A morphometric study of the lumbar spinous process in the Chinese population. Brazilian Journal of Medical and Biological Research. 2015; 48(1): 91-95.

Caldas Md P, Ambrosano GM, Haiter Neto F. New formula to objectively evaluate skeletal maturation using lateral cephalometric radiographs. Braz Oral Res. 2007 Oct–Dec;21(4):330–5. PubMed PMID: 18060260.

Chanplakorn P, Kraiwattanapong C, Aroonjarattham K, et al. Morphometric evaluation of subaxial cervical spine using multi-detector computerized tomography (MD-CT) scan: the consideration for cervical pedicle screws fixation. BMC Musculoskelet Disord. 2014;15:125. Published 2014 Apr 11. doi:10.1186/1471-2474-15-125

Chawla K, Sharma M, Abhaya A, Kumar R, Singh J. Importance of microstructure of lumbar pedicle in screw placement. Natl J Clin Anat 2012; 1:86-90

Chawla K, Sharma M, Abhaya A, Kumar R, Singh J. Importance of microstructure of lumbar pedicle in screw placement. National Journal of Clinical Anatomy. 2012;1(2):86-90.

Chen H, Shoumura S, Emura S, Bunai Y. Regional variations of vertebral trabecular bone microstructure with age and gender. Osteoporos Int. 2008; 19(10):1473–1483. doi: 10.1007/s00198-008-0593-3.

Comeau A. Age-related Changes in Geometric Characteristics of the Pediatric Thoracic Cage and Comparison of Thorax Shape with a Pediatric CPR Manikin. PhD thesis. 2010.

Cotter MM, Simpson SW, Latimer BM, Hernandez CJ. Trabecular microarchitecture of hominoid thoracic vertebrae. Anat Rec (Hoboken). 2009;292(8):1098-1106. doi:10.1002/ar.20932

Cui XG, Cai JF, Sun JM, Jiang ZS. Morphology study of thoracic transverse processes and its significance in pedicle-rib unit screw fixation. J Spinal Disord Tech. 2015;28(2):E74-E77. doi:10.1097/BSD.0000000000000163

Defino HLA, Vendrame JRB. Morphometric study of lumbar vertebrae’s pedicle. Acta Ortop Bras. [serial on the Internet]. 2007; 15(4): 183-186. Available from URL: http://www.scielo.br/aob.

Dimeglio A. Growth in pediatric orthopaedics. J Pediatr Orthop. 2001; 21:549–555.

Dimeglio A. Growth of the spine before age 5 years. J Pediatr Orthop. 1993; 1:102.

Ebraheim NA, Xu R, Knight T, Yeasting RA. Morphometric evaluation of lower cervical pedicle and its projection. Spine (Phila Pa 1976). 1997; 22(1):1–6.

Fazzalari NL, Parkinson IH, Fogg QA, Sutton-Smith P. Antero-postero differences in cortical thickness and cortical porosity of T12 to L5 vertebral bodies. Joint Bone Spine. 2006; 73(3):293–297.

Fields A. Trabecular Microarchitecture, Endplate Failure, and the Biomechanics of Human Vertebral Fractures, University of California, Berkeley, Dissertation, 2010. https://escholarship.org/uc/item/2tz8d834

Francis CC. Variations in the articular facets of the cervical vertebrae. Anat. Rec. 1955; 122: 589–602.

Gaivoronskiy IV, Manukovskiy VA, Katz AV. Morphometric characteristics of lumbar vertebra of an adult person and the feasibility of their body volume prediction in percutaneous vertebroplasty. Morphology. 2009;5 (136): 67-72. (in Russian).

Gilad I, Nissan M. Sagittal evaluation of elemental geometrical dimensions of human vertebrae. Journal of Anatomy. 1985; 143: 115-120.

Gocmen-Mas N, Karabekir H, Ertekin T, Edizer M, Canan Y, Duyar I. Evaluation of lumbarvertebral body and disc: a stereological morphometric study. Int. J. Morphol. 2010; 28(3):841-847.

Grote HJ, Amling M, Vogel M, Hahn M, Pösl M, Delling G. Intervertebral variation in trabecular microarchitecture throughout the normal spine in relation to age. Bone. 1995;16(3):301-308. doi:10.1016/8756-3282(94)00042-5

Gupta C, Radhakrishnan P, Palimar V, D’souza AS, Kiruba NL. A quantitative analysis of atlas vertebrae and its abnormalities. Journal of Morphological Sciences. 2013; 30:77–81.

Hazrati Marangalou, J. Patient specific prediction of bone failure using microstructure-enhanced continuum finite element models Eindhoven: Technische Universiteit Eindhoven. 2013. DOI: 10.6100/IR759548

Hermann AP, Brixen K, Andresen J, Mosekilde L. Reference Values for Vertebral Heights in Scandinavian Females and Males. Acta Radiol. 1993; 34(1):48-52.

Jadhav AS, Katti AS, Herekar NG, Jadhav SB. Osteological study of lumbar vertebrae in Western Maharashtra population. Journal of Anatomical Society of India. 2013; 62:10-16.

Johnson KT, Al-Holou WN, Anderson RC, Wilson TJ, Karnati T, Ibrahim M, Garton HJ, Maher CO. Morphometric analysis of the developing pediatric cervical spine. J Neurosurg Pediatr. 2016 Sep;18(3):377–89. doi: 10.3171/2016.3. PEDS1612. Epub 2016 May 27. PubMed PMID: 27231821.

Junno J-A, Niskanen M, Nieminen MT, Maijanen H, Niinima¨ki J, et al. Temporal Trends in Vertebral Size and Shape from Medieval to Modern-Day. PLoS ONE. 2009; 4(3): 4836. doi: 10.1371/journal.pone.0004836

Kathole MA, Joshi RA, Herekar NG, Jadhav SS. Dimensions of Cervical Spinal Canal and Vertebrae and Their Relevance in Clinical Practice. International Journal of Recent Trends in Science and Technology. 2012; 3(2): 54–58.

Katz PR, Reynolds HM, Foust DR, Baum JK. Mid-sagittal dimensions of cervical vertebral bodies. Am J Phys Anthropol. 1975 Nov;43(3):319-26. doi: 10.1002/ajpa.1330430304. PMID: 1211428.

Khomutova IT. Anatomy of the cervical spine of newborns with radiation research methods [Electronic resource]: The dissertation of the candidate of medical sciences: 14.00.19, Omsk 2005. (In Russian)

Kneissel M, Roschger P, Steiner W, et al. Cancellous bone structure in the growing and aging lumbar spine in a historic Nubian population. Calcif Tissue Int. 1997;61(2):95-100. doi:10.1007/s002239900302

Knirsch W, Kurtz C, Häffner N, Langer M, Kececioglu D. Normal values of the sagittal diameter of the lumbar spine (vertebral body and dural sac) in children measured by MRI. Pediatr Radiol. 2005; 35: 419–424. https://doi.org/10.1007/s00247-004-1382-6

[Kósa](https://www.sciencedirect.com/science/article/pii/S0379073804006103#!) F, [Castellana](https://www.sciencedirect.com/science/article/pii/S0379073804006103#!) C. New forensic anthropological approachment for the age determination of human fetal skeletons on the base of morphometry of vertebral column. [Forensic Sci Int.](https://www.ncbi.nlm.nih.gov/pubmed/15694735) 2005 Jan 17;147 Suppl: S69–74.

Mavrych V, Bolgova O, Ganguly P, Kashchenko S. Age-Related Changes of Lumbar Vertebral Body Morphometry. Austin J Anat. 2014;1(3): 1014.

Moussa M. A Comparative Study of the Static Histomorphometry and Marrow Content of Human Vertebral and Iliac Crest Trabecular Bone. Egypt J Histol. 2008; 31:290-300.

Mulder L, van Rietbergen B, Noordhoek NJ, Ito K. The Ability of Flat-Panel Fluoroscopy CT to Quantify Vertebral Trabecular Architecture. 56th Annual Meeting of the Orthopaedic Research Society. Poster No. 1389. 2010.

Newman SL, Gowland RL. The use of non-adult vertebral dimensions as indicators of growth disruption and non-specific health stress in skeletal populations. American journal of physical anthropology. 2015; 158 (1): 155–164.

Ostojić Z, Cvijanović O, Bobinac D, Zoricić S, Sosa I, Marić I, Crncević-Orlić Z, Mihelić R, Ostojić L, Petrović P. Age-related and gender-related differences between human vertebral and iliac crest bone–a histomorphometric study on the population of the Mediterranean Coast of Croatia. Coll Antropol. 2006; 30(1):49–54. PMID: 16617575

Pafundi D. Image-based skeletal tissues and electron dosimetry models for the ICRP reference pediatric age series. A dissertation presented to the graduate schools of the University of Florida in partial fulfillment of the requirements for the degree of doctor of the philosophy. University of Florida. 2009.

Panjabi MM, Duranceau J, Goel V, Oxland T, Takata K. Cervical human vertebrae. Quantitative three-dimensional anatomy of the middle and lower regions. Spine (Phila Pa 1976). 1991; 16(8):861–869.

Panjabi MM, Oxland T, Takata K, Goel V, Duranceau J, Krag M. Articular facets of the human spine. Quantitative three-dimensional anatomy. Spine (Phila Pa 1976). 1993; 18(10):1298–1310.

Panzer M. Numerical Modelling of the Human Cervical Spine in Frontal Impact. 2006 UWSpace. http://hdl.handle.net/10012/2865

Patel MM, Singel TC, Gohil DV, Pandya AM. A Study of Osteometric Measurements of Articular Facets from C3 to S1. Journal of the Anatomical Society of India. 2007; 56(1) (2007-01 - 2007-06)

Peters JR, Chandrasekaran C, Robinson LF, Servaes SE, Campbell RM Jr, Balasubramanian S. Age- and gender-related changes in pediatric thoracic vertebral morphology. Spine J. 2015;15(5):1000-1020. doi:10.1016/j.spinee.2015.01.016

Peters JR, Servaes SE, Cahill PJ, Balasubramanian S. Morphology and growth of the pediatric lumbar vertebrae. Spine J. 2021;21(4):682-697. doi:10.1016/j.spinee.2020.10.029

Ponrartana S, Aggabao PC, Dharmavaram NL, Fisher CL, Friedlich P, Devaskar SU, Gilsanz V. Sexual Dimorphism in Newborn Vertebrae and its Potential Implications. J Pediatr 2015; 167:416–21.

Razo RP, Elizondo-Riojas G, Martinez Garcia JE, Monterrey NL, Monterrey MX. Morphometric assessment of the lumbar pedicle isthmus by reformatted CT: variations according to age and gender. 2016. Poster number: C-1275. DOI: 10.1594/ecr2016/C-1275

Ritzel H, Amling M, Pösl M, Hahn M, Delling G. The thickness of human vertebral cortical bone and its changes in aging and osteoporosis: a histomorphometric analysis of the complete spinal column from thirty-seven autopsy specimens. J Bone Miner Res. 1997; 12(1):89–95.

Schaefer M and Black S. Juvenile Osteology: A Laboratory and Field Manual. Illustrations by Christie A. University of Dundee. Elsevier 2009.

Scheuer L, Black S. The juvenile Skeleton. Elsevier Academic Press London WC1X 8RR, UK 2004.

Schwarz JH. Skeleton Keys: An Introduction to Human Skeletal Morphology, Development and Analysis, 2nd Edition. Oxford University Press: Oxford. 2007; 402. <https://global.oup.com/us/companion.websites/9780195188592/student/part_two/Subadult_Specimens/Subadult_B/>

Seema, Verma P, Singh M. Morphometric study of pedicles of the lumbar vertebrae in adult punjabi males. Int J Anat Res. 2016; 4(2):2401-04. ISSN 2321-4287. DOI:http://dx.doi.org/10.16965/ijar.2016.209

Senegul G, Kodiglu HH. Morphometric anatomy of atlas and diameters vertebra. Turkish Neurosurgery. 2006; 16(2): 69–76.

Sharma N, Jain S.K., Singh P.K., Rohin Garg. A morphometric study of predictors for sexual dimorphism of cervical part of vertebral column in human fetuses. Journal of the Anatomical Society of India. 2017; 66: 135–139.

Shepelkevich AP, Kabak SL, Rogov YuI, Kaban NS, Lebed OA. Morphological changes in bone tissue in type 1 diabetes mellitus. Military medicine. 2011; 4(21): 68–73. (in Russian)

Silva MJ, Keaveny TM, Hayes WC. Load sharing between the shell and centrum in the lumbar vertebral body. Spine (Phila Pa 1976). 1997; 22(2):140-50. PMID: 9122793

Singel TC, Patel MM and Gohil DV. A Study of Width and Height of Lumbar Pedicles in Saurashtra Region. Journal of the Anatomical Society of India. 2004. 53: 4-9.

Singh R, Srivastva SK, Prasath CS, Rohilla RK, Siwach R, Magu NK. Morphometric measurements of cadaveric thoracic spine in Indian population and its clinical applications. Asian Spine J. 2011; 5(1):20–34.

Tager IL. X-ray diagnosis of diseases of the spine. M Medicine. 1983. (In Russian)

Tan SH, Teo EC, Chua HC. Quantitative three-dimensional anatomy of cervical, thoracic and lumbar vertebrae of Chinese Singaporeans. European Spine Journal. 2004;13(2):137–146.

Taylor JR, Twomey LT. Sexual dimorphism in human vertebral body shape. J Anat. 1984 Mar;138 (Pt 2)(Pt 2):281-6. PMID: 6715250; PMCID: PMC1164068.

van Schaik JJ, Verbiest H, van Schaik FD. Morphometry of lower lumbar vertebrae as seen on CT scans: newly recognized characteristics. AJR Am J Roentgenol. 1985 Aug;145(2):327-35. doi: 10.2214/ajr.145.2.327. PMID: 3875234.

[Vijayapalan V](https://www.ncbi.nlm.nih.gov/pubmed/?term=Vijayapalan%20V%5BAuthor%5D&cauthor=true&cauthor_uid=12629671), [Sutton-Smith P](https://www.ncbi.nlm.nih.gov/pubmed/?term=Sutton-Smith%20P%5BAuthor%5D&cauthor=true&cauthor_uid=12629671), [Parkinson IH](https://www.ncbi.nlm.nih.gov/pubmed/?term=Parkinson%20IH%5BAuthor%5D&cauthor=true&cauthor_uid=12629671), [Martin RB](https://www.ncbi.nlm.nih.gov/pubmed/?term=Martin%20RB%5BAuthor%5D&cauthor=true&cauthor_uid=12629671), [Fazzalari NL](https://www.ncbi.nlm.nih.gov/pubmed/?term=Fazzalari%20NL%5BAuthor%5D&cauthor=true&cauthor_uid=12629671). Trabecular rod thickness by direct measurement from 3D SEM anaglyphs. [Anat Rec A Discov Mol Cell Evol Biol.](https://www.ncbi.nlm.nih.gov/pubmed/?term=Vijatapalan+2003) 2003 Apr;271(2):286–90.

Wani BA, Chalkoo AH, Tariq S, Bedar A. Assessment of bone age by cervical vertebral dimensions in lateral cephalometric radiographs. J Oral Med Oral Surg Oral Pathol Oral Radiol 2018;4(3):160–163.

Wolf A, Shoham M, Michael S, Moshe R. Morphometric study of the human lumbar spine for operation-workspace specifications. Spine (Phila Pa 1976). 2001; 26(22):2472-1477.

Xu R, Burgar A, Ebraheim NA, Yeasting RA. The quantitative anatomy of the laminas of the spine. Spine (Phila Pa 1976). 1999; 24(2):107–113.

Yan, Y-B, Qi W, Wang J, Liu L-F, Teo E-C, Tianxia Q, Ba J-J, Lei W, Relationship between architectural parameters and sample volume of human cancellous bone in micro-CT scanning, Medical Engineering & Physics. 2011; 33 (6):764–769.

Zhou SH, McCarthy ID, McGregor AH, Coombs RR. & Hughes SP. Geometrical dimensions of the lower lumbar vertebrae--analysis of data from digitised CT images. Eur. Spine J. 2000; 9(3):242-248.

Zhu YH, Cheng KL, Zhong Z, Li YQ, Zhu QS. Morphologic evaluation of Chinese cervical endplate and uncinate process by three-dimensional computed tomography reconstructions for helping design cervical disc prosthesis. J Chin Med Assoc. 2016 Sep;79(9):500-6

Zhuang Z, Xie Z, Ding S, Chen Y, Luo J, Wang X, Kong K. Evaluation of thoracic pedicle morphometry in a Chinese population using 3D reformatted CT. Clin Anat. 2012; 25(4):461–467.
