# Supplementary material for: Stochastic parametric skeletal dosimetry model for humans: Anatomical-morphological basis and parameter evaluation
Source: PLoS One. 2025 Jul 2;20(7):e0327156. doi: 10.1371/journal.pone.0327156 (PMC12306906; doi:10.1371/journal.pone.0327156)
Supplement: S7 Sacrum — (DOCX) [file pone.0327156.s007.docx]

**sacrum**

**Pre-adults, analysis of published data on sacrum macro-parameters and cortical thickness**

The ossification of sacrum is complex. In different age periods, the number of mineralized components varies, from 21 in the first year of life to one bone after 12–15 years (Fig. S1). The parameters important for modeling are indicated in Fig. S1: width of sacral right-ala to left-ala (*l_1-5_*); S-vertebra transverse body width (*w_t1-5_*); S-vertebra sagittal body width (*w_s1-5_*); S-vertebra height (*h_1-5_*); total sacrum height from superior end-plate of S1 to anterior end-plate of S5 (*h_to_*_t_).


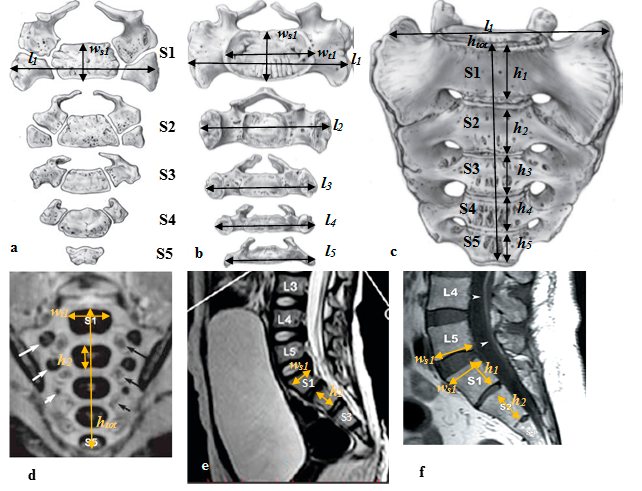


**Fig. S1.** Sacrum vertebrae of different ages; **a-c** – dry mineralized parts of sacrum (Scheuer and Black 2004): **a**- about 1-year-child, superior view on separate S-vertebrae, each vertebra contains several centers of ossification excepting S5; **b**- 5-year-child, superior view on separate S-vertebrae, each vertebra is single fused bone; **c**- 16–18 year youth, sacrum is one fused bone. **d,e,f** –body images: **d**- sacrum of 39-week-fetus similar to a newborn, dark spots are the centers of ossifications (oblique coronal image according to Jian et al. 2019); e- lumbosacral MRI image of 4-year-old child, vertebrae are indicated (sagittal cutch, Shalaby et al. 2015); e- lumbosacral MRI image of teenager, vertebrae are indicated (sagittal cutch, Scott and Neil 2015). Arrows and letters show the main parameters used for modeling (described in text).

Tables S1–S4 present the published data on sacrum-measured parameters. Table S5 contains the averaged measured values assumed for modeling. Table S6 presents the relative sizes of sacrum vertebrae in referent ages.

**Table S1.** Width of sacral right-ala to left-ala (syn. sacral breath) measured at the level of S1 superior endplate (*l_1_*).

| Author | Age | N | Sacral breath, mm | 90% range or SD |
| --- | --- | --- | --- | --- |
| Hresko et al. 2020 | 0 | ^a^ | 40 | 30–50 |
| Hresko et al. 2020 | 1 | ^a^ | 52 | 45–60 |
| Kuznetsov 1994 | 1 | 14 | 51 | 3.7 |
| Kuznetsov 1994 | 2 | 27 | 59 | 5.2 |
| Kuznetsov 1994 | 3 | 23 | 66 | 10 |
| Kuznetsov 1994 | 4 | 18 | 71 | 8.0 |
| Hresko et al. 2020 | 5 | ^a^ | 75 | 65–85 |
| Kuznetsov 1994 | 5 | 15 | 74 | 15 |
| Kuznetzov 1994 | 6 | 21 | 76 | 9.0 |
| Kuznetsov 1994 | 7 | 26 | 81 | 5.0 |
| Kuznetsov 1994 | 8 | 29 | 83 | 11 |
| Kuznetsov 1994 | 9 | 15 | 85 | 8.0 |
| Hresko et al. 2020 | 10 | ^a^ | 90 | 78–103 |
| Kuznetsov 1994 | 10 | 20 | 86 | 13 |
| Kuznetsov 1994 | 11 | 19 | 88 | 13 |
| Kuznetsov 1994 | 12 | 19 | 89 | 9.0 |
| Kuznetsov 1994 | 13 | 14 | 101 | 11 |
| Hresko et al. 2020 | 15 | ^a^ | 105 | 95–118 |

1. Each 5-year age interval was represented by a minimum of N = 67; total number of persons measured =420; the values were derived by us from graphic representation.

**Table S2.** Sagittal (anteroposterior) width of S1-vertebra body (w_s_) at the superior endplate (Hresko et al. 2020) and sagittal width of L5-vertebra body at the level of inferior endplate used as surrogate of w_s_ (Mavrych et al. 2014).

| Author | Age | N | Average *w_s_*, mm | 90% range or SD, mm |
| --- | --- | --- | --- | --- |
| Hresko et al. 2020 | 0 | ^a^ | 7.5 | - |
| Mavrych et al. 2014 | 0 | 23 | 7.8 | 0.4 |
| Hresko et al. 2020 | 1 | ^a^ | 12 | 9.8–15 |
| Mavrych et al. 2014 | 0.5–3 | 21 | 12.95 | 0.95 |
| Hresko et al. 2020 | 5 | ^a^ | 19.5 | 16.5–26 |
| Mavrych et al. 2014 | 3–7 | 7 | 23.1 | 1.4 |
| Hresko et al. 2020 | 10 | ^a^ | 24.5 | 22–29.5 |
| Mavrych et al. 2014 | 8–12 | 6 | 26.5 | 3.6 |
| Mavrych et al. 201 | 13–16 | 8 | 30.6 | 2.4 |
| Hresko et al. 2020 | 15f | ^a^ | 28 | 25–32 |
| Hresko et al. 2020 | 15m | ^a^ | 30.5 | 27–34 |

Note: L5 - fifth lumbar vertebra. Analysis of sagittal width of L5-inferior-endplate and S1-superior-endplate performed Hresko et al. 2020, shown that the values are not significantly differed; authors note “the growth pattern of L5 inferior endplate and superior sacral end plate is proportional and has nearly identical growth curve slope”.

a- Each 5-year age interval was represented by a minimum of N = 67; total number of persons measured =420; the values were derived by us from graphic representation.

**Table S3.** Transverse width of L5 body (at the level of inferior endplate) used as surrogate of S1-body-transverse-width at the level of superior end-plate (*w_t_*).

| Author | Age | N | Average *w_t_*_,_ mm | 90% range or SD, mm |
| --- | --- | --- | --- | --- |
| Mavrych et al. 2014 | 0 | 23 | 14.9 | 0.7 |
| Mavrych et al. 2014 | 0–1 | 9 | 17.7 | 1.3 |
| Hresko et al. 2020 | 1 | a | 28 | 20–35 |
| Mavrych et al. 2014 | 1–3 | 7 | 27.1 | 2.1 |
| Mavrych et al. 2014 | 3–7 | 6 | 34 | 5.1 |
| Hresko et al. 2020 | 5 | a | 37.5 | 28–46 |
| Mavrych et al. 2014 | 8–12 | 8 | 35.3 | 4.8 |
| Hresko et al. 2020 | 10 | a | 45 | 35–54 |
| Mavrych et al. 2014 | 13–16 | 33 | 45.9 | 3.7 |
| Hresko et al. 2020 | 15f | a | 48 | 37–55 |
| Hresko et al. 2020 | 15m | a | 51 | 45–55 |
| Mavrych et al. 2014 | Adults | 12 | 50.4 | 8 |

1. Each 5-year age interval was represented by a minimum of N = 67; total number of persons measured =420; the values were derived from graphic representation by us. L5 - fifth lumbar vertebra

**Table S4.** Age-dependence of total sacrum height according to Kuznetsov 1994 (*h_tot_*).

| Age | N | Average *h_tot_*, mm | SD, mm |
| --- | --- | --- | --- |
| 1 | 14 | 58 | 11 |
| 2 | 27 | 63 | 5.2 |
| 3 | 23 | 71 | 9.6 |
| 4 | 18 | 75 | 8.5 |
| 5 | 15 | 77 | 12 |
| 6 | 21 | 85 | 14 |
| 7 | 26 | 89 | 15 |
| 8 | 29 | 92 | 11 |
| 9 | 15 | 96 | 12 |
| 10 | 20 | 97 | 13 |
| 11 | 19 | 99 | 17 |
| 12 | 19 | 105 | 13 |
| 13 | 14 | 109 | 15 |

Estimates of sacrum length (*h_tot_*) in a newborn vary. Dimeglio 2020 indicates the sacrum length is about 30 mm at birth; and noted that sacrum represents about 15% of sitting height which is about 340–350 mm at birth (15% from this value is about 50 mm); Androneskuy 1970 in the review book shown sacrum length about 42 mm at birth. It should be noted that in the period 0–1 year, the maximum growth rate of all parts of the spine is observed. For modeling, the total height of the sacrum for newborns is taken 40 ± 8 mm (CV = 20% as at the age of 1 year, Table S4). The length of the sacrum in adults is on average 110–120 mm (Dimeglio 2020).

**Table S5.** Averaged sacrum parameters used in modeling.

| Age | Sacrum length (*h_tot_*), mm | | Sacral breath at S1 level (*l_1_*), mm | | S1-body-tranverse width (*w_t1_*), mm | | S1-body-sagittal width (*w_s1_*), mm | |
| --- | --- | --- | --- | --- | --- | --- | --- | --- |
|  | M | SD | M | SD | M | SD | M | SD |
| 0 | 40 | 8 | 40 | 4 | 14.9 | 0.7 | 7.5 | 0.8 |
| 1 | 58 | 11 | 51.5 | 4 | 25.2 | 2.1 | 12.5 | 1.5 |
| 5 | 77 | 12 | 74.5 | 15 | 35.8 | 5.1 | 21.3 | 1.3 |
| 10 | 97 | 13 | 88 | 13 | 40.2 | 4.8 | 25.5 | 1.5 |
| 15 | 110 | 8 | 105 | 5 | 50.5 | 8 | 30 | 2.0 |

Analysis of published images, radiographs, MRI- and CT- scans (Jian et al. 2019; Shalaby et al. 2015; Scott and Neil 2015; Scheuer and Black 2004; Cardoso et al. 2014) allowed us to estimate the relative sizes of sacral vertebrae (relative to S1) depending on age (Table S6)

**Table S6.** Relative sizes (relative to S1) assumed for sacral vertebrae depending on age.

| Vertebra | 0 Y | 1 Y | 5 Y | ≥10 Y |
| --- | --- | --- | --- | --- |
| Body height (*h*) | | | | |
| **S1** | **1** | **1** | **1** | **1** |
| S2 | 1 | 1 | 0.9 | 0.9 |
| S3 | 0.9 | 0.9 | 0.8 | 0.8 |
| S4 | 0.6 | 0.6 | 0.6 | 0.6 |
| S5 | 0.6 | 0.6 | 0.6 | 0.6 |
| Body-transverse width (superior endplate) (*w_t_*) | | | | |
| **S1** | **1** | **1** | **1** | **1** |
| S2 | 0.8 | 0.8 | 0.6 | 0.6 |
| S3 | 0.6 | 0.6 | 0.6 | 0.6 |
| S4 | 0.6 | 0.6 | 0.6 | 0.6 |
| S5 | 0.5 | 0.5 | 0.6 | 0.6 |
| Body-sagittal width (superior endplate) (*w_s_*) | | | | |
| **S1** | **1** | **1** | **1** | **1** |
| S2 | 0.8 | 0.8 | 0.7 | 0.6 |
| S3 | 0.7 | 0.7 | 0.5 | 0.4 |
| S4 | 0.7 | 0.7 | 0.3 | 0.3 |
| S5 | 0.5 | 0.4 | 0.3 | 0.3 |
| Distance from right ala to left ala ends (l) | | | | |
| **S1** | **-** | **1** | **1** | **1** |
| S2 | - | 0.8 | 0.8 | 0.8 |
| S3 | - | 0.7 | 0.7 | 0.7 |
| S4 | - | 0.6 | 0.6 | 0.6 |
| S5 | - | - | 0.3 | 0.5 |

To move from the total height of the sacrum (*h_tot_*) to the height of individual vertebrae, it is necessary to take into account the contribution of the cartilaginous layers between the S-vertebrae. According to Sadofieva 1990, from birth to 3 years of age, the cartilage presents about 1/2 of the vertical and sagittal sizes of vertebral bodies; cartilage contributes a lot to size of the lateral masses and processes. Analysis of radiographs (Sze et al. 1991) shown, the relative contribution of cartilage is quite variable in children of these ages, and ranges from 0.5 to 0.25 of vertebra sizes. The thickness of the cartilaginous layer decreases with age, by 5 years it is no more than 2–2.5 mm (Sadofieva 1990).

For age 0-Y and 1-Y, it is assumed that the contribution of cartilage to the total height (*h_tot_*) of the sacrum is 35%. For an age of 5 years, the thickness of the layer is taken 2.5 mm (4 layers between five S-vertebrae = 10 mm). At age 10, the thickness of the cartilage is not taken into account, because the vertebrae are mostly fused. Table S7 presents the accepted sizes of S1–S5 vertebrae depending on age.

**Table S7.** Dimensions of sacrum vertebrae assumed for BPSs of different ages.

| Vertebra | 0 Y | 1 Y | 5 Y | ≥10 Y |
| --- | --- | --- | --- | --- |
| Vertebra-body height (*h*), mm | | | | |
| **S1** | 6.3 | 9.2 | 17.2 | 22.1 |
| S2 | 6.3 | 9.2 | 15.5 | 19.8 |
| S3 | 5.7 | 8.3 | 13.7 | 17.6 |
| S4 | 3.8 | 5.5 | 10.3 | 13.2 |
| S5 | 3.8 | 5.5 | 10.3 | 13.2 |
| Body-transverse width (*w_t_*) mm | | | | |
| **S1** | 14.9 | 25.2 | 35.8 | 40.2 |
| S2 | 11.9 | 20.2 | 21.5 | 24.1 |
| S3 | 8.9 | 15.1 | 21.5 | 24.1 |
| S4 | 8.9 | 15.1 | 21.5 | 24.1 |
| S5 | 7.5 | 12.6 | 21.5 | 24.1 |
| Body-sagittal width (*w_s_*) mm | | | | |
| **S1** | 7.5 | 12.5 | 21.3 | 25.5 |
| S2 | 6.0 | 10.0 | 14.9 | 15.3 |
| S3 | 5.3 | 8.8 | 10.7 | 10.2 |
| S4 | 5.3 | 8.8 | 6.4 | 7.7 |
| S5 | 3.8 | 5.0 | 6.4 | 7.7 |
| Distance from right ala to left ala ends (*l*) mm | | | | |
| **S1** | **-** | 51.5 | 74.5 | 88.0 |
| S2 | - | 41.2 | 59.6 | 70.4 |
| S3 | - | 36.1 | 52.2 | 61.6 |
| S4 | - | 30.9 | 44.7 | 52.8 |
| S5 | - | - | 22.4 | 44.0 |

Note: the average CV-values for the measurements presented in Table S5 were taken as an estimate of the uncertainty: for *h* CV = 20%, for *w_t_*, *w_s_* and *l* CV=10%.

**Sacrum 0-Y, segmentation and estimation of model parameters**

Only centers of ossification of vertebral bodies are modeled. They are represented by boxes of height *h* and sides *w_t_*, *w_s_*. (Fig. S2); all parameters are presented in Table S7. All boxes are without cortical layer.

**Sacrum 1-Y, segmentation and estimation of model parameters**

Vertebral body of each S-vertebra is modeled by box of height *h* and sides *w_t_*, *w_s_* (Fig. S2, Table S7). In addition, lateral ossification centers of ala are modeled by boxes (two identical boxes to the left and right of the vertebral body). It is assumed that for the ala-BPSs, the value of *w_s_* is equal to those for the vertebral body, and the value of ala-transverse width (*w_ta_*) is calculated as:

*w_ta_* = *l* – *w_t_* – (2×2.5); where 2.5 mm is thickness of cartilaginous layers between body and ala.

Calculated values of *w_ta_* for S1–S4 are presented in Table S8. All boxes are without cortical layer.

**Table S8.** Values of *w_ta_* assumed for S1–S4 (age 1-Y).

| Vertebra | *w_ta_* mm | SD, mm |
| --- | --- | --- |
| S1 | 10.7 | 1.1 |
| S2 | 8.0 | 0.8 |
| S3 | 8.0 | 0.8 |
| S4 | 5.4 | 0.5 |

| BPS | S1  w_s_ | S2 | S3 | S4 | S5 |
| --- | --- | --- | --- | --- | --- |
| Body  0 Y | *h_1_*  w_t_ |  |  |  |  |
| Body 1 Y | *h_1_*  w_t_  w_s_  w_s_ |  |  |  |  |
| Ala  1 Y | *h_1_*  *w_ta_* |  |  |  | _ |

**Fig. S2.** BPSs used for sacrum modeling of newborn and 1-Y child.

**Sacrum 5-Y, segmentation and estimation of model parameters**

At this age, sacrum consists of 5 bone segments (Scheuer and Black 2004), between which are cartilaginous layers with a thickness of about 2–2.5 mm (Sadofieva 1990). Bone segments are modeled by five boxes elongated horizontally (Fig. S3). All parameters are presented in Table S7. The cortical layer covers the two sides: anterior and posterior. The thickness of the cortical layer is taken 0.65 ± 0.23 mm, it is two times thinner than in adults (adult value 1.31 ±0.46 mm is based on Richards et al. 2010 and Peretz et al. 1998).


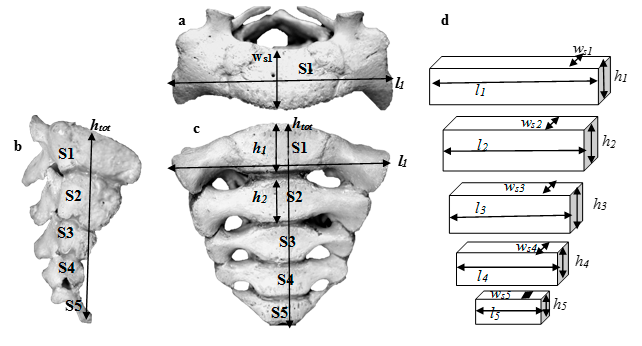


**Fig. S3.** Sacrum of 5-year-children: a – superior view; b- sagittal view; c-posterior view (based Schwarz 2007); (d) – BPSs for 5-Y-sacrum.

**Sacrum 10-Y, segmentation and estimation of model parameters**

At this age, there is a large variability in the number of individual sacral segments: from one, as in adults, to five, as in the previous age group (Cardoso et al. 2014). Cartilaginous layers (if any) are very thin; they were not taken into account evaluating the heights of the vertebral bodies. The size of the vertebrae is smaller than in adults. Sacrum is modeled similarly to the age of 5 years, i.e., with five boxes elongated horizontally (Fig. S3) with a cortical layer on the front, back, upper and lower sides (i.e., on all sides except the ends). The thickness of the cortical layer is taken to be 1.5 times thinner than in adults and is 0.9± 0.31 mm. Linear dimensions of BPSs are in Table S7).

**Sacrum 15-Y, segmentation and estimation of model parameters**

At this age, the formation of the sacrum is largely completed (Sadofieva 1990; Scheuer and Black 2004; Cardoso et al. 2014). The model corresponds to that for adults.

**Adults and 15-Y, analysis of published data on sacrum macro-parameters and cortical thickness**

Adult sacrum (Fig 2.4) was divided into 10 segments; three segments modeled the fused *bodies* of sacrum vertebrae; three segments modeled the *ala* (fused lateral parts of vertebrae, i.e., fused transverse processes); and four segments – *pedicles*.

1. *Body* S1 was described by a box of *h_b1_-*height, *w_b1_-*weight (anteroposterior diameter), *l_b1_*- length (transverse diameter)*.* Cortical layer is located on two (anterior and posterior) sides of BPS.
2. *Body S2-3* was described by a box of *h_sb2-3_* -height (sum of heights of S2 and S3 bodies: *h_b2_+ h_b3_*); *w_b2-3_*-width (averaged over the S2 and S3 bodies); *l_b2-3_*- length (averaged over the S2 and S3 bodies). Cortical layer is located on two (anterior and posterior) sides of BPS.
3. *Body S4-5* was described by a box of *h_sb4-5_* -height (sum of heights of S4 and S5 bodies: *h_b4_+ h_b5_*); width *w_b4-5_* (averaged over the S2 and S3 bodies); and *l_b4-5_*- length (averaged over the S4 and S5 bodies). Cortical layer is located on two (anterior and posterior) sides of BPS.
4. *Ala S1* was described by a box of *h_a1_* – height (equal to the height of S1 body *h_b1_*), *w_a1_* - width (dimension in anteroposterior direction); and *l_a1_* – length (dimension in transverse direction). Value of *l_a1_* was derived from the value of breath of sacrum (BS) and its components: body-S1-transverse dimension (*l_b1_*), pedicle-S1-height (*h_p1_*), ala-S1-length (*l_a1_*). Cortical layer does not cover two sides: side adjacent to S1- pedicle and side adjacent to S2-ala.
5. *Ala S2* was described by a box of *h_a2_* – height (equal to the height of S2-body); *l_a2_* –length; and *w_a2_* - width (dimension in anteroposterior direction that is a sum of S2-body-width *w_b2_* and diameter of sacral canal d_c2_). Cortical layer is located on the anterior, posterior and lateral side.
6. *Ala* S3-4 was described by a right triangular prism (acute angle downwards) with an isosceles triangle at the base; one triangular base is adjacent to *pedicle-S3 and S4*; smaller [rectangular](https://en.wikipedia.org/wiki/Rectangle) side is adjacent to ala-S2. Prism height is *l_a3-4_*. Dimension of triangular base are width - *w_a3-4_* (equal to the sum of body-S3-width and diameter of sacral canal: *w_b3_+* d_c3_*);* height- *h_a3-4_* (sum of heights of S3 and S4 bodies: *h_b3_+* *h_b4_*); the dimension of lateral sides (a=b) was calculated from measured value of *w_a3-4_* and *h_a3-4_*. Cortical layer covers two larger sides of prism and one base.
7. *Pedicle S2* was approximated by elliptic cylinder of axis (diameter) *d*1_p2_ and *d*2_p2_ (equal to the width of ala – S2) and height *h_p2_*; cortical layer is located on the walls of the cylinder.
8. *Pedicle* S1 was approximated by elliptic cylinder of axis (diameter) *d1_p1_* and *d*2_p1_ (equal to the width of body-S1); and height *h_p1_* (distance between the body-S1 and ala-S1); cortical layer is located on the walls of the cylinder.
9. *Pedicle* S3 was approximated by elliptic cylinder of axis (diameter) *d*1_p3_ and *d*2_p3_ (equal to the width of ala – S3) and height *h_p3_*; cortical layer is located on the walls of the cylinder.
10. *Pedicle* S4 was approximated by elliptic cylinder of axis (diameter) *d*1_p4_ and *d*2_p4_ (equal to the sum of body-S4 weight and diameter of sacral canal: *w_b4_+* d_sc4_) and height *h_p_*; cortical layer is located on the walls of the cylinder.

**Fig S4.** Sacrum of adult male. (a) CT-image of coronal section; (b) Superior view; (c) Anterior view; (d) Lateral view and sagittal section. (e) stylized models (BPS) describing sacrum segments: (1) body-S1; (2) body-S2-3; (3) body-S4-5; (4) ala-S1; (5) ala-S2; (6) ala-S3-4; (7) pedicle-S1; (8) pedicle-S2; (9) pedicle-S3; (10) pedicle-S4. Letters are deciphered in the text.

The following BPSs have sex-specific dimensions: the *bodies* of the sacral vertebrae and *ala S1* and *ala S2.* Parameters for *alaS1* and *alaS1* are derived from the value of breath of sacrum (BS, analysis of the literature showed that they are independent of sex), pedicle-S1-height (*h_p1_*) and sex-dependent body transverse diameter (*l_b1_*).

**Table S9.** Sacral body macro-parameters for adult **male**, published data (mean±STD, mm).

| Author | Age (range) | N | *h_b_* | *w_b_* | *l_b_* |
| --- | --- | --- | --- | --- | --- |
| **Body S1** | | | | | |
| Sinha et al. 2013 | Adult | 38 | 28±2 | 29±2 | 46±5 |
| Nisha et al. 2015 | Adult | 83 | - | 29±2 | 48±4 |
| Vasantha et al. 2014 | Adult | 27 | - | - | 49±6 |
| Pradhan et al. 2015 | 40 (20-60) | 25 | 28±2 | 32±2 | - |
| Sachdeva et al. 2011 | Adult | 40 | - | 32±2 | 48±7 |
| Pelin et al. 2005 | 63 (45-81) | 42 | 32±3 | - | - |
| Arman et al. 2009^a^ | Adult | 100 | 30±2 | 31±3 | 49±6 |
| Stolfi et al. 1992 ^a^ | Adult | 4 | - | 27±7 | - |
| Pininski et al. 2014 | 64 (39-89) | 51 | 33±3 | - | 56±6 |
| Zech et al. 2012 | 56 (22-90) | 49 | - | 34±3 | 55±5 |
| **Assumed for BPS (CV%)** | | | **30 (7)** | **31 (10)** | **50 (12)** |
| **Body S2** | | | | | |
| Pradhan et al. 2015 | 40 (20-60) | 25 | - | 20±3 | - |
| Pelin et al. 2005 | 63 (45-81) | 42 | 26±3 | - | - |
| Arman et al. 2009 | Adult | 100 | 26±6 | - | 30±3 |
| Stolfi et al. 1992 | Adult | 4 | - | 18±1 | - |
| Firat et al. 2017 | Adult | 53 | 25±3 | 18±3 | 31±3 |
| Pininski et al. 2014 | 64 (39-89) | 51 | 27±3 | - | - |
| **Assumed for BPS (CV%)** | | | **26 (15)** | **18 (11)** | **30 (10)** |
| **Body S3** | | | | | |
| Pelin et al. 2005 | 63 (45-81) | 42 | 21±3 | - | - |
| Arman et al. 2009 | Adult | 100 | - | - | 28±3 |
| Stolfi et al. 1992 | Adult | 4 | - | 12±1 | - |
| Pininski et al. 2014 | 64 (39-89) | 51 | 20±2 | - | - |
| **Assumed for BPS (CV%)** | | | **20 (15)** | **12 (8)** | **28 (11)** |
| **Body S4** | | | | | |
| Pelin et al. 2005 | 63 (45-81) | 42 | 18±3 | - | - |
| Stolfi et al. 1992 | Adult | 4 | - | 9±1 | - |
| Pininski et al. 2014 | 64 (39-89) | 51 | 19±3 | - | - |
| **Assumed for BPS (CV%)** | | | **18 (17)** | **9 (11)** | **28 (11)^b^** |
| **Body S5** | | | | | |
| Pelin et al. 2005 | 63 (45-81) | 42 | 18±2 | - | - |
| Stolfi et al. 1992 | Adult | 4 | - | 8±1 |  |
| Pininski et al. 2014 | 64 (39-89) | 51 | 18±3 | - | - |
| **Assumed for BPS (CV%)** | | | **18 (17)** | **8 (12)** | **28 (11) ^b^** |

^a^- m+f

^b^- was taken the same as for S3

**Table S10.** Sacral-breadth value used for estimation of ala-S1 parameters for **male**, published data (mean±STD, mm).

| Author | Age | N | Sacral breath (BS) |
| --- | --- | --- | --- |
| Mustafa et al. 2012 | Adult | 24 | 114±6 |
| Maddikunta et al. 2014 | Adult | 27 | 104±7 |
| Sachdeva et al. 2011 | Adult | 40 | 103±8 |
| Punase et al. 2016 | Adult | 77 | 109±7 |
| **Assumed for BPS (CV%)** | **108 (6)** | | |

**Table S11.** Sacral body macro-parameters for adult f**emale**, published data (mean±STD, mm).

| Author | Age (range) | N | *h_b_* | *w_b_* | *l_b_* |
| --- | --- | --- | --- | --- | --- |
| **Body S1** | | | | | |
| Sinha et al. 2013 | Adult | 12 | 28±2 | 30±3 | 47±5 |
| Yadav et al. 2015 | Adult | 57 | - | 27±2 | 41±4 |
| Maddikunta et al. 2014 | Adult | 33 | - | - | 45±6 |
| Pradhan et al. 2015 | 40 (20–60) | 25 | 29±2 | 30±2 | - |
| Sachdeva et al. 2011 | Adult | 10 | - | 29±2 | 46±5 |
| Arman et al. 2009 ^a^ | Adult | 100 | 30±2 | 31±3 | 49±6 |
| Stolfi et al. 1992 ^a^ | Adult | 4 | - | 27±7 | - |
| Pininski and Brits 2014 | 66 (29–93) | 51 | 32±3 | - | 52±6 |
| Zech et al. 2012 | 56 (22–90) | 49 | - | 34±3 | 55±5 |
| **Assumed for BPS (CV%)** | | | **30 (9)** | **28 (7)** | **46 (11)** |
| **Body S2** | | | | | |
| Pradhan et al. 2015 | 40 (20–60) | 25 | - | 18±3 | - |
| Ebraheim et al. 1997 | 68 (54–85) | 20 | 25 | 14 | 29 |
| Arman et al. 2009^1^ | Adult | 100 | 26±6 | - | 30±3 |
| Pininski and Brits 2014 | 66 (29–93) | 51 | 26±2 | - | - |
| **Assumed for BPS (CV%)** | | | **26 (9)** | **16 (19)** | **30 (10)** |
| **Body S3** | | | | | |
| Arman et al. 2009^1^ | Adult | 100 | - | - | 28±3 |
| Stolfi et al. 1992^1^ | Adult | 4 | - | 12±1 | - |
| Pininski and Brits 2014 | 64 (39-89) | 51 | 20±3 | - | - |
| **Assumed for BPS (CV%)** | | | **20 (13)** | **12 (8)** | **28 (11)** |
| **Body S4** | | | | | |
| Stolfi et al. 1992 | Adult | 4 | - | 9±1 | - |
| Pininski and Brits 2014 | 64 (39–89) | 51 | 18±2 | - | - |
| **Assumed for BPS (CV%)** | | | **18 (12)** | **9 (11)** | **28 (11) ^b^** |
| **Body S5** | | | | | |
| Stolfi et al. 1992 | Adult | 4 | - | 8±1 | - |
| Pininski and Brits 2014 | 64 (39–89) | 51 | 17±3 | - | - |
| **Assumed for BPS (CV%)** | | | **17 (17)** | **8 (13)** | **28 (11) ^b^** |

^a^- m+f;

^b^- was taken the same as for S3

*h_b_–*vertebra body height (shortest distance between intravertebral joints)

*w_b–_* anteroposterior diameter of vertebra body

*l_b_–* transverse diameter of vertebra body

**Table S12.** Sacral-breadth value used for estimation of ala-S1 parameters for **female**, published data (mean and ±STD, mm).

| Author | Age | N | Sacral breath (BS) |
| --- | --- | --- | --- |
| Mustafa et al. 2012 | Adult | 24 | 115±9 |
| Maddikunta and Ravinder 2014 | Adult | 27 | 103±6 |
| Sachdeva et al. 2011 | Adult | 40 | 101±7 |
| Punase et al. 2016 | Adult | 77 | 113±6 |
| **Assumed for BPS (CV%) 108 (5)** | | | |

**Table S13.** Sacral-canal-diameter value used for estimation of parameters for ala-BPS (mean±STD, mm), according to Frostel (2016) (male and female).

| Parameter | S1 | S2 | S3 | S4 | S5 |
| --- | --- | --- | --- | --- | --- |
| *d_sc_* | 8±2 | 7±3 | 6±3 | 5±3 | 4±3 |
| N | 129 | 65 | 27 | 15 | 15 |
| **Assumed for BPS (CV%)** | **8 (25)** | **7 (43)** | **6 (50)** | **5 (60)** | **4 (75)** |

**Table S14.** S1-ala width for combined sample of adult male and female, published data (mean±STD, mm).

| Author | Age | N | *w_a_* |
| --- | --- | --- | --- |
| Arman et al. 2009 | Adult | 100 | 54±4* |
| Esenkaya et al. 2003 | Adult | 30 | 51±6 |
| Ebraheim et al. 1998 | Adult | 11 | 46±2 |
| **Assumed for BPS (CV%)** | | | **53 (10)** |

Comments*: w_a_*- the distance between the anterior and posterior borders of the sacral ala neighboring to sacroiliac joint

Sacral ala-S2 length (*l_a2_*) was measured by Esenkaya (2003): N=30, (male and female); *l_a2_*= 23±4 mm.

Sacral ala-S3-4 length (*l_a3-4_*) was measured by us using SUSHPU –collection: N=5; *l_a3-4_*=19±3 (male and female).

Where: *l_a_* – the distance between the lateral walls of the foramina in the direction of transverse line (linea transversalis), and the anterior border of the articular surface on the same horizontal direction, on the ventral surface of the sacrum

**Table S15.** Pedicle macro-parameters for combined sample of adult male and female, published data (mean±STD, mm).

| Author | Age | N | *h_p_^*^* | *d1_p_* | *d2_p_** |
| --- | --- | --- | --- | --- | --- |
| **S1**-pedicle | | | | | |
| Arman et al 2009 | Adult | 100 | 14±2 | 16±2 | 25±4 |
| Okutan et al. 2003 | Adult | 18 | - | 14±3 | 22±3 |
| Sachdeva et al 2011 | Adult | 40 | - | - | 24±3 |
| **Assumed for BPS (CV%)** | | | **14 (14)** | **15 (13)** | **24 (12)** |
| **S2**-pedicle | | | | | |
| Robinson et al. 2016 | Adult | 30 | - | 14±3 | - |
| Arman et al. 2009 | Adult | 100 | 14±2 | 13 ±2 | - |
| **Assumed for BPS (CV%)** | | | **14 (14)** | **14(14)** | **24 (12)**** |
| **S3**-pedicle | | | | | |
| Robinson et al. 2016 | Adult | 30 | - | 13±2 | - |
| **Assumed for BPS (CV%)** | | | **14 (14)**** | **13(15)** | **24 (12)**** |
| S4-pedicle | | | | | |
| Robinson et al. 2016 | Adult | 30 | - | 11±2 | - |
| **Assumed for BPS (CV%)** | | | **14 (14)**** | **11(18)** | **24 (12)**** |

*- measured value for sacral-foramen transverse-diameter that is equal to pedicle height (*h_p_* );

** - was taken the same as for S1 pedicle; *d_1p_* – interforaminal distance

**Table S16**. Cortical thickness of sacrum (averaged over sacral surfaces) for combined sample of male and female.

| Author | Age (range) | | N | Ct.Th |
| --- | --- | --- | --- | --- |
| Richards et al. 2010 | 76 (62-90) | | 6 | 1.52±0.12 |
| **Assumed for BPS (CV%)** |  | **1.52 (8)** | | |

The values of micro-parameters for trabecular structures, their individual variability (CV); and intra-specimen variability (CV_s_) for sacral BPSs were taken the same as for the lumbar vertebra

**Reference for sacrum**

Androneskuy А. Anatomy of a child. Publishing House "Meredian". Bucharest. 1970. (In Russian)

Arman C, Naderi S, Kiray A, Aksu FT, Yilmaz HS, Tetik S, Korman E. The human sacrum and safe approaches for screw placement. J Clin Neurosci. 2009; 16(8):1046–1049.

[Cardoso HF](https://www.ncbi.nlm.nih.gov/pubmed/?term=Cardoso%20HF%5BAuthor%5D&cauthor=true&cauthor_uid=24227049), [Pereira V](https://www.ncbi.nlm.nih.gov/pubmed/?term=Pereira%20V%5BAuthor%5D&cauthor=true&cauthor_uid=24227049), [Rios L](https://www.ncbi.nlm.nih.gov/pubmed/?term=Rios%20L%5BAuthor%5D&cauthor=true&cauthor_uid=24227049). Chronology of fusion of the primary and secondary ossification centers in the human sacrum and age estimation in child and adolescent skeletons. [Am J Phys Anthropol.](https://www.ncbi.nlm.nih.gov/pubmed/?term=Cardoso+sacrum+2014) 2014 Feb;153(2):214–25. doi: 10.1002/ajpa.22422.

Dimeglio A, Bonnel F, Canavese F. The Growing Spine. In: Spinal Anatomy. Modern Concepts. Springer. 2020; 25–52.

Ebraheim NA, Lin D, Xu R, Stanescu S, Yeasting RA. Computed tomographic evaluation of the internal structure of the lateral sacral mass in the upper sacra. Orthopedics. 1999; 22(12):1137–40.

Esenkaya I, Aluçlu MA, Kavakli A, Bulut HT. Radiologic and morphologic evaluation of the lateral sacral mass. Acta Orthop Traumatol Turc. 2003; 37(4):330–339.

Firat A, Alemdaroğlu KB, Özmeriç A, Yücens M, Göksülük D. Morphometric study of the true S1 and S2 of the normal and dysmorphic sacralized sacra. Turk J Med Sci. 2017; 47(3):954–959.

Frostell A, Hakim R, Thelin EP, Mattsson P and Svensson M. A Review of the Segmental Diameter of the Healthy Human Spinal Cord. Front. Neurol. 2016; 7:238. doi: 10.3389/fneur.2016.00238. eCollection 2016.

Hresko AM, Hinchcliff EM, Deckey DG, Hresko MT. Developmental sacral morphology: MR study from infancy to skeletal maturity. Eur Spine J. 2020. https://doi.org/10.1007/s00586-020-06350-6.

[Jian N](https://www.ncbi.nlm.nih.gov/pubmed/?term=Jian%20N%5BAuthor%5D&cauthor=true&cauthor_uid=30607474), [Lin N](https://www.ncbi.nlm.nih.gov/pubmed/?term=Lin%20N%5BAuthor%5D&cauthor=true&cauthor_uid=30607474), [Tian MM](https://www.ncbi.nlm.nih.gov/pubmed/?term=Tian%20MM%5BAuthor%5D&cauthor=true&cauthor_uid=30607474), [Zhang S](https://www.ncbi.nlm.nih.gov/pubmed/?term=Zhang%20S%5BAuthor%5D&cauthor=true&cauthor_uid=30607474), [Li G](https://www.ncbi.nlm.nih.gov/pubmed/?term=Li%20G%5BAuthor%5D&cauthor=true&cauthor_uid=30607474), [Zhao H](https://www.ncbi.nlm.nih.gov/pubmed/?term=Zhao%20H%5BAuthor%5D&cauthor=true&cauthor_uid=30607474), [Xiao LX](https://www.ncbi.nlm.nih.gov/pubmed/?term=Xiao%20LX%5BAuthor%5D&cauthor=true&cauthor_uid=30607474), [Liang WJ](https://www.ncbi.nlm.nih.gov/pubmed/?term=Liang%20WJ%5BAuthor%5D&cauthor=true&cauthor_uid=30607474), [Lin XT](https://www.ncbi.nlm.nih.gov/pubmed/?term=Lin%20XT%5BAuthor%5D&cauthor=true&cauthor_uid=30607474). Normal development of costal element ossification centers of sacral vertebrae in the fetal spine: a postmortem magnetic resonance imaging study. [Neuroradiology.](https://www.ncbi.nlm.nih.gov/pubmed/30607474) 2019 Feb;61(2):183–193. doi: 10.1007/s00234-018-2147–5.

Kuznetsov LE. Pelvic fractures in children (morphology, biomechanics, diagnosis). Moscow, Folium. 1994. (in Russian)

Maddikunta V, Ravinder M. Morphometric study of sacrum in sex determination in Telengana region people. Int J Res Med Sci. 2014; 2(1):164–174.

Mavrych V, Bolgova O, Ganguly P and Kashchenko S. Age-Related Changes of Lumbar Vertebral Body Morphometry. Austin J Anat. 2014;1(3): 7.

Mustafa MS, Mahmoud OM, El Raouf HH, Atef HM. Morphometric study of sacral hiatus in adult human Egyptian sacra: Their significance in caudal epidural anesthesia. Saudi J Anaesth. 2012; 6(4):350–357.

Nisha Y, Kopal S, Kalpana P. Determination of sex using dry adult human sacrum- a morphometric study. IJCRR. 2015; 7(3): 22–28.

Okutan O, Kaptanoğlu E, Solaroğlu I, Beşkonakli E, Tekdemir I. Pedicle morphology of the first sacral vertebra. Neuroanatomy. 2003; 2:16–19.

Pelin C, Duyar I, Kayahan EM, Zağyapan R, Ağildere AM, Erar A. Body height estimation based on dimensions of sacral and coccygeal vertebrae. J Forensic Sci. 2005; 50(2):294–297.

Peretz AM, Hipp JA, Heggeness MH. The internal bony architecture of the sacrum. Spine (Phila Pa 1976). 1998;23(9):971‐974.

Pininski M, Brits D. Estimating stature in South African populations using various measures of the sacrum. Forensic Sci Int. 2014 Jan; 234:182.

Pradhan A, Yadav S. Sexual dimorphism of the sacrum in South Indian population using MRI. International Journal of Pharma and Bio Sciences. 2015; 6 (4). B-398.

Punase VK, Agrawal NL, Shirivastava S.K. A Morphometric Study on Dry Adult Human Sacrum for Determination of Sex. Paripex - indian journal of research. 2016; 5(3).

Richards AM, Coleman NW, Knight TA, Belkoff SM, Mears SC. Bone density and cortical thickness in normal, osteopenic, and osteoporotic sacra. J Osteoporos. 2010:504078. Published 2010 Jun 9. doi:10.4061/2010/504078

Robinson TJ, Roberts SL, Burnham RS, Loh E, Agur AM. Sacro-Iliac Joint Sensory Block and Radiofrequency Ablation: Assessment of Bony Landmarks Relevant for Image-Guided Procedures. Biomed Res Int. 2016. 1432074. DOI: 10.1155/2016/1432074

Sachdeva K, Rajan K S, Gurdeep K, Gaurav S. Role of Sacrum in Sexual Dimorphism-A Morphometric Study. Indian Acad Forensic Med. 2011; 33(3)

Sadofyeva VI. Normal X-ray anatomy of the bone-joint system of children. Leningrad “Medicine” Leningrad branch 1990. (In Russian)

Scheuer L, Black S. The juvenile Skeleton. Elsevier Academic Press London WC1X 8RR, UK 2004.

Schwarz JH. Skeleton Keys: An Introduction to Human Skeletal Morphology, Development and Analysis, 2nd Edition. Oxford University Press: Oxford, 2007; 402. <https://global.oup.com/us/companion.websites/9780195188592/student/part_two/Subadult_Specimens/Subadult_B/>

Scott EF, Neil MB. Imaging Anatomy of the Human Spine. A Comprehensive Atlas Including Adjacent Structures. Demosmedical. Springer Publishing Company. 2015.

Shalaby SA, Eid EM, Saber NA, Ali AM, Gad S. Morphometric (MRI and sonography) study of the human spinal cord in prenatal and postnatal life (from birth to 20 years). Benha Medical Journal. 2015; 32: 146–151.

Sinha MB, Rathore M, Trivedi S, Siddiqui A U. Morphometry of first pedicle of sacrum and its clinical relevance. International J. of Healthcare & Biomedical Research. 2013; 1(4): 234–240.

Stolfi VM, Milsom JW, Lavery IC et al. Newly designed occluder pin for presacral hemorrhage. Dis Colon Rectum. 1992; 35:166–169.

[Sze G](https://www.ncbi.nlm.nih.gov/pubmed/?term=Sze%20G%5BAuthor%5D&cauthor=true&cauthor_uid=1947104), [Baierl P](https://www.ncbi.nlm.nih.gov/pubmed/?term=Baierl%20P%5BAuthor%5D&cauthor=true&cauthor_uid=1947104), [Bravo S](https://www.ncbi.nlm.nih.gov/pubmed/?term=Bravo%20S%5BAuthor%5D&cauthor=true&cauthor_uid=1947104). Evolution of the infant spinal column: evaluation with MR imaging. [Radiology.](https://www.ncbi.nlm.nih.gov/pubmed/1947104) 1991 Dec;181(3):819–27.

Vasantha M, Ravinder M. Morphometric study of sacrum in sex determination in Telengana region people. Int J Res Med Sci. 2014; 2(1): 164–174.

Yadav N, Saini K, Patil K. Determination of sex using dry adult human sacrum- a morphometric study. IJCRR 2015; 7 (3): 22-28.

Zech WD, Hatch G, Siegenthaler L, Thali MJ, Lösch S. Sex determination from os sacrum by postmortem CT. Forensic Sci Int. 2012 Sep 10;221(1-3):39–43.
