# Supplementary material for: Stochastic parametric skeletal dosimetry model for humans: Anatomical-morphological basis and parameter evaluation
Source: PLoS One. 2025 Jul 2;20(7):e0327156. doi: 10.1371/journal.pone.0327156 (PMC12306906; doi:10.1371/journal.pone.0327156)
Supplement: S8 Fibula — (DOCX) [file pone.0327156.s008.docx]

**Fibula**

**Pre-adults, analysis of published data on fibula macro-parameters and cortical thickness**

The shape of the fibula is not significantly dependent on age (Scheuer and Black 2004, White et al. 2011), Fig. Fi1 illustrates the age-changes in the period 0–5 years. For these ages, fibula diaphysis is described by one round cylinder. By the age of 10, in the central part of the fibula diaphysis, the bone marrow is replaced by adipose tissue; only the distal and proximal parts of the diaphysis are described by two identical small cylinders as shown in Fig. Fi1 (f).

**Fig. Fi1.** Fibula, (a – d) radiograph (x-rays) images of fibula and tibia of different ages from (Normal pediatric bone X-ray); (e, f) stylized models (BPS) describing fibula of children aged 0–5 years (e) and 10-Y (f). Fi – Fibula; Ti- tibia; other letter designations are deciphered in the text.

Main measured parameters used in modeling:

- Maximal diaphysis length *(L_mdl_)-* distance between the proximal and distal growth zone (epiphyseal lines), does not include the epiphysis ossified from separate centers (Fig. Fi1);
- Diameters (*D_m_*) in mid-point of diaphysis (Fig. Fi1);
- Cortical thickness (Ct.Th) in the mid-point of diaphysis;

Two BPSs describes fibula of reference ages

0–5-Y:

BPS 1 (body) was described by a round cylinder of height *h_m_* = *L_mdl_* and diameter *d_m =_ D_m_*; cortical layer is located on the walls of the cylinder;

10-Y:

BPS 2 (distal and proximal ends) was described by a small round cylinder of height *h_m_* = 0.17 *L_mdl_* and diameter *d_m=_ D_m_*; cortical layer is located on the walls of the cylinder.

Table Fi1 presents the published data on *L_mdlf_*

**Table Fi1.** Published data on maximal diaphyseal length (*L_mdl_*), mm according to Maresh et al. 1970.

| Age | N | M | SD |
| --- | --- | --- | --- |
| 0.125 | 69 | 66.8 | 4.4 |
| 0.25 | 65 | 77.1 | 4.1 |
| 0.5 | 78 | 84.9 | 5.2 |
| 1 | 81 | 105 | 5.1 |
| 1.5 | 84 | 121.3 | 5.9 |
| 2 | 84 | 136 | 6.8 |
| 2.5 | 82 | 147.9 | 7.1 |
| 3 | 79 | 159.4 | 7.9 |
| 3.5 | 78 | 169.6 | 8.3 |
| 4 | 80 | 179.5 | 9.1 |
| 4.5 | 78 | 189.4 | 10.2 |
| 5 | 80 | 198.6 | 11.1 |
| 5.5 | 74 | 206.5 | 11.7 |
| 6 | 75 | 216 | 12.2 |
| 6.5 | 81 | 224.3 | 13.4 |
| 7 | 86 | 232.1 | 13.4 |
| 7.5 | 83 | 240.8 | 14.5 |
| 8 | 85 | 248.8 | 14.8 |
| 8.5 | 82 | 256.1 | 15.2 |
| 9 | 83 | 263.7 | 16.3 |
| 9.5 | 83 | 272.2 | 17.6 |
| 10 | 84 | 279.4 | 18.3 |
| 10.5 | 75 | 287.2 | 20.4 |
| 11 | 76 | 294.4 | 19.8 |
| 11.5 | 75 | 303.8 | 20.7 |
| 12 | 71 | 311.1 | 20.8 |

Jeanty et al. 1983 presents the data on late fetuses of age=40 w (n=450 total); *L_mdl_*=63; SD=1.5; Demidov et al 1990 (data from Medvedev 2009) also reported data for fetuses of age=40-42 w; *L_mdl_*=63; SD=2.0.

Bernert et al. 2007, on the basis of measurements of the children’s fibula, proposed empirical relationships between the length of the diaphysis (*L_mdl_*) and the maximal (*D_max_*) and minimal (*D_min_*) fibular diameters:

*L_mdl_ =* 22.026 × *D_max_* − 1.688;

*L_mdl_ =* 30.619 *× D_min_* − 18.057.

According to calculations, values *D_max_* and *D_min_* do not differ significantly and they were averaged by us to derive *D_m_*.

Table Fi2 presents the averaged values of *L_mdl_* assumed for models along with calculated values of mid-diaphysis diameters *D_m._* Cortical thickness for all segments was taken as half the thickness for the tibia mid-diaphysis (Appendix Tibia); analysis of images from White et al. (2011) do not contradict our assumption; values of Ct.Th are also presented in Table Fi2.

**Table Fi2.** Assumed values of main measured fibula-parameters used in BPSs modeling, mm.

| Age | *L_mdlf_* | | Ct.Th | | *D_m_* | |
| --- | --- | --- | --- | --- | --- | --- |
|  | M | SD | M | SD | M | SD |
| 0 | 66.8 | 4.4 | 0.9 | 0.15 | 2.9 | 0.2 |
| 1 | 105.0 | 5.1 | 1.5 | 0.20 | 4.4 | 0.5 |
| 5 | 198.6 | 11.1 | 2.0 | 0.35 | 8.1 | 0.5 |
| 10 | 279.4 | 18.3 | 2.1 | 0.25 | 11.2 | 0.7 |

**Table Fi3.** Assumed BPS parameters for fibula.

| BPS | Age | *d_m_* | SD | *h_m_* | SD | Ct.Th | SD |
| --- | --- | --- | --- | --- | --- | --- | --- |
| #1 | 0 | 2.9 | 0.2 | 66.8* | 4.4 | 0.7 | 0.1 |
| #1 | 1 | 4.4 | 0.5 | 105.0* | 5.1 | 1.2 | 0.2 |
| #1 | 5 | 8.1 | 0.5 | 198.6* | 11.1 | 1.5 | 0.3 |
| #2 | 10 | 11.2 | 0.7 | 47.4 | 3.2 | 1.7 | 0.2 |

*- for DFs calculation, *h_m_* is taken 30mm

**Microstructures of fibula trabecular bone**

The trabecular structure of the fibula is assumed to be the same as that of the tibia (see Section Tibia).

**References for fibula**

Bernert Zs, Évinger S, Hajdu T. New data on the biological age estimation of children using bone measurements based on historical populations from the Carpathian Basin. Annales Historico-Naturales Musei Nationalis Hungarici. 2007; 99: 199–206.

Medvedev MV Ed. Ultrasonic Fetometry: Reference Tables and Nomograms Ed. 8th, rev. Moscow: Real time Publisher. 2009; 19–24 (in Russian).

Jeanty P. Fetal limb biometry. Radiology. 1983 May;147(2):601-2. doi:10.1148/radiology.147.2.6836145. PMID: 6836145.

Maresh MM. Measurements from roentgenograms. In: Human Growth and Development (RW. McCammon, Ed.) Springfield, IL: Charles C. Thomas. 1970; 157–200.

Normal pediatric bone X-ray: Accessed at: <https://bonexray.com/>; <http://bones.getthediagnosis.org/>

Scheuer L, Black S. The juvenile Skeleton. Elsevier Academic Press London WC1X 8RR, UK 2004.

White TD, Black MT, Folkens PA. Human osteology: Third edition. Human Osteology: Third Edition. 2011;1–662.
