# Supplementary material for: Stochastic parametric skeletal dosimetry model for humans: Anatomical-morphological basis and parameter evaluation
Source: PLoS One. 2025 Jul 2;20(7):e0327156. doi: 10.1371/journal.pone.0327156 (PMC12306906; doi:10.1371/journal.pone.0327156)
Supplement: S9 Skull — (DOCX) [file pone.0327156.s009.docx]

**Skull (head)**

**Analysis of published data on skull macro-parameters and cortical thickness**

Skull mainly consists of flat bones, they make up the vault (entirely), and partly skull-base and the facial-skull. To describe the skull one BPS describing the flat bones (mainly neurocranium) was modeled. As a result, we describe bones containing about 90% of cranial AM.

**Fig. Sk1.** Scheme of skull: (a) –x-ray images of newborn, children and adult; (b) stylized model BPS.

BPS is a box with parameters *30 × 30 × Sk.Th* (mm), where *Sk.Th* is the average thickness of the skull. The BPS are used for all ages

The results of measurements of skull thickness of pre-adults are presented in Table Sk1. Each author examined a significant number of skull-points located in frontal, parietal and occipital bones.

The degree of severity of cortical layers in the skull bones depends on age. In adults, the flat bones consist of two cortical layers (outer and inner) and intermediate trabecular part (diploe). However, at birth, the lamellar structure of the skull-bones is represented by a single layer (Margulies and Coats 2013; Rodriguez-Florez et al. 2017); three layers are formed with subsequent development. It is accepted that:

For newborns the flat bone is represented by one layer, and this layer is a trabecular bone.

For pre adults of 1-15 years old, the flat bones of the skull have a three-layer structure, the thickness of the cortical layer is the same on the outer and inner sides; in total, both layers make ½ the thickness of the skull (Table Sk1). Table Sk2 presents the BPS-parameters assumed for pre-adult skull

For adults, there is enough data on measuring the thickness of cortical layers and diploe. These data were used for BPS-parameter estimation (Tables Sk3-4).

**Table Sk1.** Thickness of skull vault, published data for pre-adults (mm).

| Author | Age | Average thickness | SD |
| --- | --- | --- | --- |
| De Boer et al. 2015 | 0–0.5 | 1.80 | 0.38 |
| Margulies and Coats 2013 | 0–0.5 | 1.89 | 0.48 |
| Li et al. 2015 | 0–0.5 | 2.37 | 0.64 |
| Margulies and Coats 2013 | 0.5–1 | 2.27 | 0.65 |
| De Boer et al. 2015 | 0.5–1.5 | 2.42 | 1.22 |
| Li et al. 2015 | 1 | 2.99 | 0.84 |
| Margulies and Coats 2013 | 1–2 | 3.25 | 0.53 |
| Li et al. 2015 | 3 | 4.66 | 1.56 |
| Margulies and Coats 2013 | 3–5 | 4.30 | 0.64 |
| De Boer et al. 2015 | 3–6 | 3.63 | 1.03 |
| Margulies and Coats 2013 | 6–7 | 4.49 | 0.89 |
| De Boer et al. 2015 | 7–12 | 4.36 | 1.01 |
| Margulies and Coats 2013 | 8–11 | 4.90 | 0.68 |
| Margulies and Coats 2013 | 12–15 | 5.08 | 0.63 |
| Margulies and Coats 2013 | 16–18 | 5.28 | 0.63 |

Note: Margulies and Coats 2013 presented pooled data from several studies, each group had 32 to 53 people; De Boer et al. 2015 examined 103 skulls from pre-adults, and 54 people from 0 to 5 years old; Li et al. 2015 examined 30 skulls from pre-adults aged 0–3 years

**Table Sk2.** BPS-parameters assumed for pre-adult skull.

| Age | Sk.Th | SD | Ct.Th | SD |
| --- | --- | --- | --- | --- |
| 0 | 2.02 | 0.50 | -^*^ | - |
| 1 | 2.73 | 0.81 | 0.68 | 0.20 |
| 5 | 4.20 | 1.08 | 1.05 | 0.27 |
| 10 | 4.63 | 0.84 | 1.16 | 0.21 |
| 15 | 5.18 | 0.63 | 1.29 | 0.16 |

^*^- whole BPS is represented by trabecular bone

**Table Sk3.** Skull diploe thickness for adults averaged over the frontal, parietal, and occipital bones for a mixed sample of adult male and female, published data (mean ± SD, mm)

| Author | Age | | N | M | SD |
| --- | --- | --- | --- | --- | --- |
| Boruah et al. 2015 | Adults | | 14 | 3.5 | 1.6 |
| Sabanciogullari et al. 2013 | 20–90 | | 220 | 3.5 | 1.0 |
| Alexander et al. 2017 | 76–86 | | 4 | 4 | 1.3 |
| Hatipoglu et al. 2008 | 45±15 | | 107 | 3.6 | 1.3 |
| Lynnerup et al. 2005 | 16–90 | | 161 | 2.5 | 1.2 |
| Hwang et al. 2000 | 55 | | 58 | 3.2 | 1.4 |
| Ruan and Prasad 2001 | Adults | | 7 | 3.6 | 1.0 |
| Sullivan and Smith 1989 | 59–96 | | 37 | 3.4 | 1.4 |
| **Averaged for BPS (CV%)** | | **3.4 (30)** | | | |

**Table Sk4.** The thickness of the outer and inner cortical layer of the flat bones of the brain skull (parietal, frontal and occipital) for a mixed sample of adult men and women (mean ± SD, mm)

| Author | Age | N | Outer layer | | Inner layer | |
| --- | --- | --- | --- | --- | --- | --- |
|  |  |  | M | SD | M | SD |
| Boruah et al. 2015 | 55±9 | 10 | 0.77 | 0.14 | 0.5 | 0.1 |
| Jung et al. 2003 | Adults | 47 | 1.57 | 0.2 | 1.49 | 0.1 |
| Ruan and Prasad 2001 | Adults | 7 | 1.53 | 0.4 | 1.47 | 0.5 |
| Hwang et al. 2000 | 39–79 | 58 | 1.86 | 0.7 | 1.9 | 1.0 |
| Sullivan and Smith 1989 | 59–96 | 37 | 1.7 | 0.3 | 1.4 | 1.0 |
| Lillie et al. 2016 | Adults | 2 | 1.36 | 0.54 | 1.2 | 1.0 |
| Peterson and Dechow 2003 | Adults | 10 | 1.9 | 0.32 | 1.6 | 0.3 |
| Hodgson et al. 1970 | Adults | 14 | 1.96 | 0.46 | 1.8 | 0.4 |
| Sabanciogullari et al. 2013 | 61–90 | 110 | 1.45 | 0.27 | 1.3 | 0.2 |
| Alexander et al. 2017 | 76–86 | 4 | 1.6 | 0.4 | 1.75 | 0.6 |
| Torres-Lagares et al. 2010 | 16–79 | 78 | 1.34 | 0.05 | - | - |
|  |  |  |  |  |  |  |

| **Averaged for BPS (CV%)** | **1.52 (22)** | **1.34 (33)** |
| --- | --- | --- |

**Analysis of published data on skull-bone microstructures**

Table Sk5 presents the published data on trabecular microstructures of adults.

Skull biopsies of the infant were examined in detail by Rodriguez-Florez et al. (2017); biopsies were obtained from patients with isolated sagittal synostosis undergoing spring-assisted cranioplasty (non-syndromic, single suture, n = 18; age from 3.7 to 7.4 month; average age = 5.3 ± 1.1 month). Rodriguez-Florez et al. (2017) note that “the earliest time at which a diploe cavity was seen was at 4.7 months of age, while the latest time at which the bone still had a one layered structure was at 7.2 months. There was no difference in BV/TV between one- and three-layered bones (p = 0.285)”.

Rodriguez-Florez et al. (2017) estimate the value BV/TV to be equal to 0.5±0.1 for infant. A similar BV/TV value was derived from published data for adults (Table Sk4) (0.52±0.1), total number of measured adult-person n=174. Image analysis of histological-sections of pre-adults (García Gil et al. 2016) showed, the value of Tb.Th, with its variability, does not differ significantly from that for adults.

Therefore, it was assumed that trabecular bone parameters are independent of age and correspond to those for adults (Table Sk5)

**Table Sk5.** Published data for skull-bone microstructures for adults

| Author | Age | N | BV/TV | | Tb.Th | | Tb.Sp | |
| --- | --- | --- | --- | --- | --- | --- | --- | --- |
|  |  |  | M | SD | M | SD | M | SD |
| Torres-Lagares et al. 2010 | 16–79 | 78 | 0.54 | 0.09 | - | - | - | - |
| Boruah et al. 2015 | 55±9 | 10 | 0.51 | 0.20 | - | - | - | - |
| Lorc'h-Bukiet et al. 2005 | Adults | 24 | 0.54 | 0.14 | - | - | - | - |
| Alexander et al. 2017 | 76–86 | 4 | 0.40 | 0.03 | - | - | - | - |
| Hwang et al. 2000 | 39–79 | 58 | 0.60 | 0.14 | 0.29 | 0.09 | 0.57 | 0.2 |
| **Averaged for BPS (CV%)** | | | **0.52 (33–71)** | | **0.29 (32)** | | **0.57 (35)** | |

**References for flat bones of skull**

Alexander SL, Rafaels K, Gunnarsson CA, Weerasooriya T. Morphological Characterization of the Frontal and Parietal Bones of the Human Skull. Technical Report. US Army Research Laboratory. Aberdeen Proving Ground, MD 21005-5066. March 2017.

Boruah S, Paskoff GR, Shender BS, Subit DL, Salzar RS, Crandall JR. Variation of bone layer thicknesses and trabecular volume fraction in the adult male human calvarium. Bone. 2015; 77:120–134.

[De Boer HH](https://www.ncbi.nlm.nih.gov/pubmed/?term=De%20Boer%20HH%5BAuthor%5D&cauthor=true&cauthor_uid=26914798), [Van der Merwe AE](https://www.ncbi.nlm.nih.gov/pubmed/?term=Van%20der%20Merwe%20AE%5BAuthor%5D&cauthor=true&cauthor_uid=26914798), [Soerdjbalie-Maikoe VV](https://www.ncbi.nlm.nih.gov/pubmed/?term=Soerdjbalie-Maikoe%20VV%5BAuthor%5D&cauthor=true&cauthor_uid=26914798). Human cranial vault thickness in a contemporary sample of 1097 autopsy cases: relation to body weight, stature, age, sex and ancestry. [Int J Legal Med.](https://www.ncbi.nlm.nih.gov/pubmed/26914798) 2016 Sep;130(5):1371–7. doi: 10.1007/s00414-016-1324-5.

García Gil O, Cambra-Moo O, Audije Gil J, Nacarino-Meneses C, Rodríguez Barbero MA, Rascón Pérez J., González Martín A. Investigating histomorphological variations in human cranial bones through ontogeny. C. R. Palevol. 2016; 15: 527–535. doi: 10.1016/j.crpv.2015.04.006

Hatipoglu HG, Ozcan HN, Hatipoglu US, Yuksel E. Age, sex and body mass index in relation to calvarial diploe thickness and craniometric data on MRI. Forensic Sci Int. 2008; Nov 20;182(1-3):46–51.

Hodgson VR, Greenberg SW, Murray LT, Brinn J. Fracture behavior of the skull frontal bone against cylindrical surfaces. Stapp Car Crash Conference. Fourteenth. Proceedings, SAE, 1970; 341–355.

Hwang K, Hollinger JO, Chung RS, Lee SI. Histomorphometry of parietal bones versus age and race. J Craniofac Surg. 2000; 11(1):17–23.

Image available: http://images.fineartamerica.com/images-medium-large/human-skull-development-d-roberts.jpg

Jung YS, Kim HJ, Choi SW, Kang JW, Cha IH. Regional thickness of parietal bone in Korean adults. Int J Oral Maxillofac Surg. 2003;32(6):638-41.

[Li Z](https://www.ncbi.nlm.nih.gov/pubmed/?term=Li%20Z%5BAuthor%5D&cauthor=true&cauthor_uid=25992998), [Park BK](https://www.ncbi.nlm.nih.gov/pubmed/?term=Park%20BK%5BAuthor%5D&cauthor=true&cauthor_uid=25992998), [Liu W](https://www.ncbi.nlm.nih.gov/pubmed/?term=Liu%20W%5BAuthor%5D&cauthor=true&cauthor_uid=25992998), [Zhang J](https://www.ncbi.nlm.nih.gov/pubmed/?term=Zhang%20J%5BAuthor%5D&cauthor=true&cauthor_uid=25992998), [Reed MP](https://www.ncbi.nlm.nih.gov/pubmed/?term=Reed%20MP%5BAuthor%5D&cauthor=true&cauthor_uid=25992998), [Rupp JD](https://www.ncbi.nlm.nih.gov/pubmed/?term=Rupp%20JD%5BAuthor%5D&cauthor=true&cauthor_uid=25992998), [Hoff CN](https://www.ncbi.nlm.nih.gov/pubmed/?term=Hoff%20CN%5BAuthor%5D&cauthor=true&cauthor_uid=25992998), [Hu J](https://www.ncbi.nlm.nih.gov/pubmed/?term=Hu%20J%5BAuthor%5D&cauthor=true&cauthor_uid=25992998).A statistical skull geometry model for children 0–3 years old. [PLoS One.](https://www.ncbi.nlm.nih.gov/pubmed/25992998) 2015 May 18;10(5):e0127322. doi: 10.1371/journal.pone.0127322. eCollection 2015.

Lillie EM, Urban JE, Lynch SK, Weaver AA, Stitzel JD. Evaluation of Skull Cortical Thickness Changes With Age and Sex From Computed Tomography Scans. J Bone Miner Res. 2016; Feb;31(2):299–307.

Lorc'h-Bukiet LI, Tulasne JF, Llorens A, Lesclous P. Parietal bone as graft material for maxillary sinus floor elevation: structure and remodeling of the donor and of recipient sites. Clin. Oral Implants Res. 2005; 16(2):244–249.

Lynnerup N, Astrup JG, Sejrsen B. Thickness of the human cranial diploe in relation to age, sex and general body build. Head Face Med. 2005; Dec 20; 1:13.

Margulies S. and Coats B. Experimental Injury Biomechanics of the Pediatric Head and Brain. Chapter 4 in: Pediatric Injury Biomechanics Springer Science+Business Media New York 2013; 157–190.

Peterson J, Dechow PC. Material properties of the human cranial vault and zygoma. Anat Rec A Discov Mol Cell Evol Biol. 2003; Sep;274(1):785–97.

[Rodriguez-Florez N](https://www.ncbi.nlm.nih.gov/pubmed/?term=Rodriguez-Florez%20N%5BAuthor%5D&cauthor=true&cauthor_uid=28734753), [Ibrahim A](https://www.ncbi.nlm.nih.gov/pubmed/?term=Ibrahim%20A%5BAuthor%5D&cauthor=true&cauthor_uid=28734753), [Hutchinson JC](https://www.ncbi.nlm.nih.gov/pubmed/?term=Hutchinson%20JC%5BAuthor%5D&cauthor=true&cauthor_uid=28734753), [Borghi A](https://www.ncbi.nlm.nih.gov/pubmed/?term=Borghi%20A%5BAuthor%5D&cauthor=true&cauthor_uid=28734753), [James G](https://www.ncbi.nlm.nih.gov/pubmed/?term=James%20G%5BAuthor%5D&cauthor=true&cauthor_uid=28734753), [Arthurs OJ](https://www.ncbi.nlm.nih.gov/pubmed/?term=Arthurs%20OJ%5BAuthor%5D&cauthor=true&cauthor_uid=28734753), [Ferretti P](https://www.ncbi.nlm.nih.gov/pubmed/?term=Ferretti%20P%5BAuthor%5D&cauthor=true&cauthor_uid=28734753), [Dunaway D](https://www.ncbi.nlm.nih.gov/pubmed/?term=Dunaway%20D%5BAuthor%5D&cauthor=true&cauthor_uid=28734753), [Schievano S](https://www.ncbi.nlm.nih.gov/pubmed/?term=Schievano%20S%5BAuthor%5D&cauthor=true&cauthor_uid=28734753), [Jeelani NUO](https://www.ncbi.nlm.nih.gov/pubmed/?term=Jeelani%20NUO%5BAuthor%5D&cauthor=true&cauthor_uid=28734753). Cranial bone structure in children with sagittal craniosynostosis: Relationship with surgical outcomes. [J Plast Reconstr Aesthet Surg.](https://www.ncbi.nlm.nih.gov/pubmed/28734753) 2017; Nov;70(11):1589–1597. doi: 10.1016/j.bjps.2017.06.017.

Ruan J, Prasad P. The effects of skull thickness variations on human head dynamic impact responses. Stapp Car Crash J. 2001; 45:395–414.

Sabanciogullari V, Salk I, Cimen M. The relationship between total calvarial thickness and diploe in the elderly. Int. J. Morphol. 2013; 31(1):38–44.

Sullivan WG, Smith AA. The split calvarial graft donor site in the elderly: a study in cadavers. Plast Reconstr Surg. 1989; Jul;84(1):29–31.

Torres-Lagares D, Tulasne JF, Pouget C, Llorens A, Saffar JL, Lesclous P. Structure and remodelling of the human parietal bone: an age and gender histomorphometric study. J Craniomaxillofac Surg. 2010; 38(5):325–30.
