# Supplementary material for: Stochastic parametric skeletal dosimetry model for humans: Anatomical-morphological basis and parameter evaluation
Source: PLoS One. 2025 Jul 2;20(7):e0327156. doi: 10.1371/journal.pone.0327156 (PMC12306906; doi:10.1371/journal.pone.0327156)
Supplement: S10 Radius Ulna — (DOCX) [file pone.0327156.s010.docx]

**Radius and Ulna**

Segments describing only one bone (radius) were developed. It was assumed that the ulna has the same characteristics as the radius.

**Analysis of published data on radius macro-parameters and cortical thickness**

The shape and size of the radius are not significantly dependent on age, Fig. Ra1 illustrates the age-changes in the period 0–5 years. For children over 5 years of age, the radius is not modeled, since active hematopoiesis ceases in it.


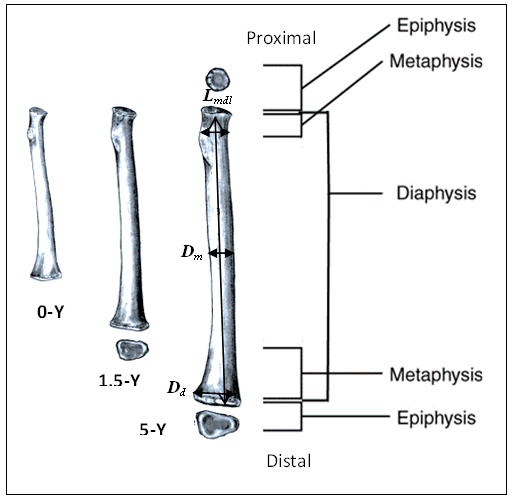


**Fig. Ra1.** Age-changes in radius, bones in natural proportion (Baker et al. 2005); radius of newborn, infant and children of about 5 years are shown; epiphyseal centers of ossification are indicated; divisions into diaphysis, metaphysis, and epiphysis are similar with other tube-bones and diaphysis length (syn. maximal diaphysis length) includes the length of metaphysis (Buikstra and Ubelaker 1994; Maresh 1970). Letter designations are deciphered in the text.

Main measured parameters described in literature which were collected:

- Maximal diaphysis length *(L_mdl_)-* distance between the proximal and distal growth zone (epiphyseal lines), does not include the epiphysis ossified from separate centers (Table Ra1);
- Cortical thickness (Ct.Th) in the mid-point and distal part of diaphysis;
- Diameters (*D_d_*) in distal part of radius (Table Ra2);

**Table Ra1.** Published data on maximal diaphyseal length (*L_mdl_*), mm.

| Author | Age, year | N | M | SD |
| --- | --- | --- | --- | --- |
| Jeanty et al. 1983 | 40wk | 450 | 50 | 3.0 |
| Demidov et al. 1990* | 40-42wk | n/a | 56 | 2.5 |
| Medvedev et al. 1999* | 40wk | n/a | 58 | 2.5 |
| Florence 2007 | 0.0 | 12 | 49.9 | 6.8 |
| Gindhart 1973 | 0.1 | 123 | 54.9 | 2.8 |
| Maresh 1970 | 0.1 | 59 | 58.8 | 3.1 |
| Florence 2007 | 0.2 | 35 | 54.5 | 3.4 |
| Gindhart 1973 | 0.3 | 219 | 61.1 | 3.2 |
| Maresh 1970 | 0.3 | 124 | 64.7 | 3.1 |
| Florence 2007 | 0.4 | 5 | 59.1 | 4.2 |
| Gindhart 1973 | 0.5 | 376 | 68.3 | 3.6 |
| Maresh 1970 | 0.5 | 145 | 69.2 | 3.5 |
| Florence 2007 | 0.8 | 4 | 69.4 | 7.2 |
| Gindhart 1973 | 0.8 | 220 | 74.7 | 4.3 |
| Gindhart 1973 | 1.0 | 367 | 80.9 | 4.6 |
| Maresh 1970 | 1.0 | 153 | 80.8 | 3.7 |
| Gindhart 1973 | 1.5 | 223 | 91.0 | 5.9 |
| Maresh 1970 | 1.5 | 151 | 89.5 | 4.2 |
| Florence 2007 | 2.0 | 3 | 97.3 | 20.0 |
| Gindhart 1973 | 2.0 | 345 | 98.8 | 5.1 |
| Maresh 1970 | 2.0 | 152 | 96.8 | 4.6 |
| Gindhart 1973 | 2.5 | 214 | 105.9 | 5.5 |
| Maresh 1970 | 2.5 | 153 | 103.3 | 4.9 |
| Gindhart 1973 | 3.0 | 345 | 112.6 | 5.9 |
| Maresh 1970 | 3.0 | 150 | 109.7 | 5.3 |
| Gindhart 1973 | 3.5 | 212 | 118.6 | 6.1 |
| Maresh 1970 | 3.5 | 151 | 115.4 | 5.9 |
| Gindhart 1973 | 4.0 | 359 | 124.4 | 6.7 |
| Maresh 1970 | 4.0 | 152 | 121.2 | 5.7 |
| Florence 2007 | 4.5 | 1 | 107.3 |  |
| Gindhart 1973 | 4.5 | 191 | 130.1 | 7.1 |
| Maresh 1970 | 4.5 | 149 | 126.7 | 6.1 |
| Gindhart 1973 | 5.0 | 347 | 135.9 | 7.4 |
| Maresh 1970 | 5.0 | 157 | 132.0 | 6.5 |
| Gindhart 1973 | 5.5 | 165 | 141.5 | 7.8 |
| Maresh 1970 | 5.5 | 147 | 136.8 | 6.8 |

* cited from Medvedev et al. 2009

Bernert et al. 2007, on the basis of measurements of the radius of children, proposed empirical relationships between the length of the diaphysis (*L_mdl_*) and the transverse (*D_tr_*) and anteroposterior (*D_tr_*) diameter:

*L_mdl_ =* 14.319 × *D_tr_ −* 4.74;

*L_mdl_ =* 19.272 *× D_ant_ −* 9.022;

Table Ra2 presents the averaged values of *L_mdl_* assumed for models and calculated value of mid-diaphysis diameters.

**Table Ra2.** Averaged values of *L_mdl_* assumed for models and calculated value of mid-shaft diameters (*D_m_*), mm

| Age | *L_mdl_* | | *D_tr_* | *D_ant_* | *D_m_** | |
| --- | --- | --- | --- | --- | --- | --- |
|  | M | SD | M | M | M | CV% |
| 0 | 57.3 | 3.7 | 4.3 | 3.4 | 3.9 | 20 |
| 1 | 81.1 | 5.0 | 6.0 | 4.7 | 5.3 | 20 |
| 5 | 130.0 | 7.0 | 9.4 | 7.2 | 8.3 | 20 |

*- average between *D_tr_* and *D_ant_* diameters;

**Table Ra3.** Published data on cortical thickness of radius diaphysis, mm

| Author | Point of measurements | Age, year | n | M | SD |
| --- | --- | --- | --- | --- | --- |
| Quick et al. 2006 | Mid-point | 8.2 | 88 | 1.77 | 0.17 |
| O'Berin et al. 2017 | Mid-point | 9.6 | 20 | 1.55 | 0.25 |
| Kawalilak 2017 | Distal metaphysis | 11.3 | 29 | 0.35 | 0.14 |
| Wang et al. 2011 | Distal metaphysis | 12.5 | 61 | 0.55 | 0.28 |
| Farr 2014 | Distal metaphysis | 12 | 108 | 0.77 | 0.07 |

As you can see, there is data on Ct.Th only for older age than necessary for modeling. In order to extrapolate the available data “to the left” from the age of 8–9 years (Table Ra3), we used the age dependence of the increase in cortical thickness for the humerus (Section Humerus). The evaluation results are presented in Table Ra4.

**Table Ra4.** Estimated Ct.Th in mid-shaft and distal part of radius

| Age | Relative Ct.Th in mid-shaft of humerus | Estimated Ct.Th in mid-shaft of radius | CV% | Relative Ct.Th in distal part of humerus | Estimated Ct.Th in distal part of radius | CV% |
| --- | --- | --- | --- | --- | --- | --- |
| 10 | **1** | **1.66 ^a^** | **13 ^a^** | 1 | **0.34 ^a^** | **40 ^a^** |
| 5 | 0.9 | 1.45 | 20 ^b^ | 0.96 | 0.33 | 20 ^b^ |
| 1 | 0.7 | 1.12 | 20 ^b^ | 0.51 | 0.17 | 20 ^b^ |
| 0 | 0.5 | 0.91 | 20 ^b^ | 0.41 | 0.14 | 20 ^b^ |

a -average for children of 8–11 ages from Table Ra3

b- corresponds to CVs for humeral measurements

**Radius segmentation and estimation of model parameters**

The diaphysis is represented by a tube with slightly widened ends. Bone for all ages was modeled by two segments (Fig. Ra2). Table Ra5 summarizes the approaches to BPS parameter derivation.

BPS 1 (body) was described by round cylinder of height *h_m_* and diameter *d_m_*; cortical layer is located on the walls of the cylinder;

BPS 2 (distal end) was described by the truncated cone of height *h_d_*; round base of diameter *d_m_* and elliptical base of diameters *d_m_* and d*_d_*; cortical layer is located on the walls of the cylinder.

| 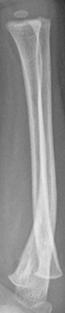a | b 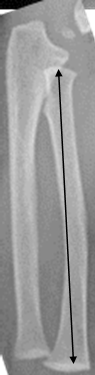 **L_mdl_** | c 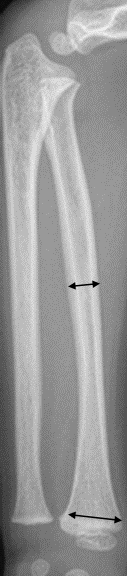 **D_m_**  **d_d_** | d  **d_m_**  **L_mdl_**  **20%L_mdl_**  **d_d_**  **d_m_** 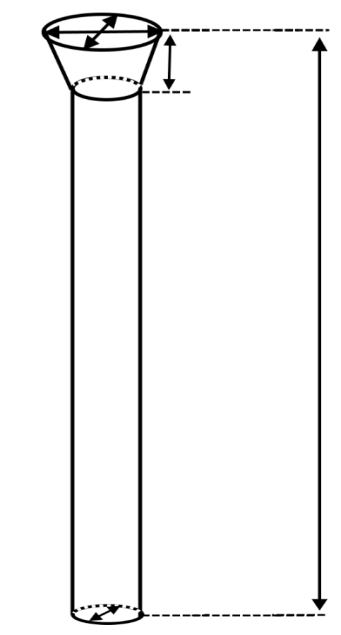 |
| --- | --- | --- | --- |

**Fig. Ra2.** Radius, (a – с) radiograph (x-rays) images (Normal pediatric bone X-ray): (a) 1–Y lateral view; (b) 1-Y anterior view; (d) 5-Y anterior view; (c) stylized models (BPS) describing radius segments; Letter designations are deciphered in the text.

**Table Ra5.** BPS parameter assumed for radius 0–5 Y (mm).

| BPS | Parameter | Rationale | 0-Y | | 1-Y | | 5-Y | |
| --- | --- | --- | --- | --- | --- | --- | --- | --- |
|  |  |  | M | SD | M | SD | M | SD |
| #1, #2 | *d_m_* | = *D_m_* calculated based on *L_mdl_* (Table Ra) | 3.9 | 0.3 | 5.3 | 0.33 | 8.3 | 0.45 |
| #1 | *h_m_* | 80% of maximal diaphysis length *L_mdl_* | 45.7 | 3.0 | 64.9 | 4.0 | 104.0 | 5.6 |
| #1 | *Ct.Th.1* | Estimated based on age-dependence | 0.91 | 0.12 | 1.12 | 0.14 | 1.45 | 0.18 |
| #2 | *h_d_* | 20% of maximal diaphysis length *L_mdl_* | 11.5 | 0.74 | 16.2 | 1.0 | 26.0 | 1.4 |
| #2 | *d_d_* | 1.5×*d_m_* (image analysis)* | 5.8 | 0.38 | 8.0 | 0.5 | 12.5 | 0.7 |
| #2 | *Ct.Th.2* | Estimated based on age-dependence | 0.31 | 0.09 | 0.38 | 0.11 | 0.49 | 0.14 |

* the value of the ratio *d_d_*/ *d_m_* was taken from analysis of x-ray images (normal pediatric bone X-ray).

**Analysis of published data on radius microstructures**

We did not find data on the trabecular microstructure of the radius in children and adolescents evaluated using μCT or histomorphometry. However, a significant number of studies were performed with use of HR-pQCT (resolution 82 μm). As mentioned above, the values obtained on the same sample by HR-pQCT and μCT are significantly different (Zhou 2015). However, HR-pQCT can be used to estimate age-related dynamics, that is, relative changes in the parameters of BV/TV and Tb.Th. We collected HR-pQCT data for children of different ages and compared them with adult data obtained with two methods (μCT and HR-pQCT) (Table Ra6-7).

**Table Ra6.** Published data used for radius data-analysis.

| Author | Method | N | Age±SD (range) | BV/TV, r.u. | SD BV/TV | Tb.Th, mm | SD Tb.Th |
| --- | --- | --- | --- | --- | --- | --- | --- |
| Kirmani et al. 2008 | pQCT | 11 m | 7(6–8) | 0.145 | 0.07 | 0.075 | - |
| Kirmani et al. 2008 | pQCT | 11 f | 7(6–8) | 0.155 | 0.07 | 0.075 | - |
| Mitchel et al. 2018 | pQCT | 3 | 7.5(6–9) | 0.12 | 0.026 | 0.19 | 0.017 |
| Wang et al. 2011 | pQCT | 61 | 12.5 (7–20) | 0.136 | 0.025 | 0.067 | 0.009 |
| Gabel et al. 2017 | pQCT | 166 m | 15.1±2.6 | 0.158 | 0.03 | 0.08 | 0.015 |
| Gabel et al. 2017 | pQCT | 185 f | 14.5±3.4 | 0.141 | 0.026 | 0.072 | 0.01 |
| Farr and Khosla 2014 | pQCT | 58 m | 11.9±0.2 | 0.146 | 0.003 | 0.072 | 0.011 |
| Farr and Khosla 2014 | pQCT | 50 f | 11.9±0.3 | 0.132 | 0.003 | 0.071 | 0.001 |
| Maatta et al. 2015 | pQCT | 74 m | 12.7±1.8 | 0.154 | 0.06 | 0.072 | 0.002 |
| Maatta et al. 2015 | pQCT | 61 f | 11.0±1.8 | 0.142 | 0.06 | 0.068 | 0.002 |
| Kawalilak et al. 2017 | pQCT | 32 | 11.3(8–13) | 0.154 | 0.025 | 0.069 | 0.01 |
| Yang et al. 2017 | pQCT | 20 f | 48.3±14.8 | 0.14 | 0.03 | 0.071 | 0.013 |
| Kocijan et al. 2015 | pQCT | 30 | 43.5 (38.2–51.9) | 0.129 | 0.02 | 0.075 | 0.011 |
| Zhou 2015* | pQCT | 26 | 72±11 | 0.121 | 0.023 | 0.086 | 0.01 |
| Zhou 2015* | μCT | 26 | 72±11 | 0.132 | 0.025 | 0.16 | 0.01 |
| Maquer et al. 2015 | μCT | 3 | Adults | 0.165 | 0.039 | - | - |
| Lochmuller et al. 2008 | μCT | 66 m | 79.7±9.13 | 0.15 | 0.05 | 0.16 | 0.002 |
| Chirchir 2015 | μCT | 38 | Adults | 0.21 | 0.03 | - | - |
| Pafundi 2009 | μCT | 1 m | 18 | 0.196 | - | - | - |
| O'Relly 2018 | μCT | 1 f | 45 | 0.131 | - | 0.22 | - |
| Hough 2011 | μCT | 1 m | 40 | 0.116 | - | 0.198 | - |

***** Measurement of each sample by two methods. m-male; f-female; both sexes if not indicated.

**Table Ra7.** Measured data on trabecular space for adults, mm.

| Author | Age | n | M | SD | Min | Max |
| --- | --- | --- | --- | --- | --- | --- |
| Zhou 2015* | 72±11 | 26 | 0.789 | 0.104 | 0.581 | 0.997 |
| Lochmuller et al. 2008 | 79.7±9.13 | 66m | 0.740 | 0.110 | 0.520 | 0.960 |
| Average |  |  | 0.765 | 0.11 | 0.52 | 0.10 |

As can be seen from Table Ra6, values of BV/TV are independent of age in children ≥5 y. This is also confirmed by a study of Mitchel et al. (2018). In adults, HR-pQCT measurements of BV/TV are generally lower than μCT results, but they are in the range of scatter of the measured values. As for the thickness of the trabeculae, as Mitchel et al. (2018) noted, there is no age-dependence of Tb.Th values in children over 5 y. However, in adults, the HR-pQCT estimates of Tb.Ths are lower than the μCT estimates by a factor of 2 (as is expected taking into account the investigation of Zhou 2015). Thus, for children ≥ 5 years old, values of BV/TV and Tb.Th were taken to be equal to those for adults obtained by μCT.

For children under 5 years, we assume that the dynamics of changes in micro-parameters are similar to those for the humerus. This means that in newborns BV/TV is 1.3 times higher and Tb.Th is 2 times lower than in children ≥5 years; Tb.Th in children of 1 year are 20% lower than in children ≥ 5. The accepted values of the micro-parameters for Radii are presented in Table Ra8.

**Table Ra8**. Parameters assumed for Radius model (all segments).

| Age group | BV/TV, r.u.  (min–max) | SD BV/TV | Tb.Th, mm  (min–max) | SD Tb.Th | Tb.Sp, mm  (min–max) | SD Tb.Sp |
| --- | --- | --- | --- | --- | --- | --- |
| 0 | 0.213  (0.113–0.313) | 0.05 | 0.08  (0.04–0.120) | 0.02 | 0.51  (0.268–0.752) | 0.121 |
| 1 | 0.163  (0.063–0.263) | 0.05 | 0.134  (0.094–0.174) | 0.02 | 0.765  (0.523–1.007) | 0.121 |
| 5 | 0.163  (0.063–0.263) | 0.05 | 0.16  (0.120–0.20) | 0.02 | 0.765  (0.545–0.985) | 0.11 |
| 10 | 0.163  (0.063–0.263) | 0.05 | 0.16  (0.120–0.20) | 0.02 | 0.765  (0.545–0.985) | 0.11 |
| 15 | 0.163  (0.063–0.263) | 0.05 | 0.16  (0.120–0.20) | 0.02 | 0.765  (0.545–0.985) | 0.11 |

**References for radius**

Baker B, Dupras TL, Tocheri MW, Wheeler SM. The osteology of infants and children. Texas A and M University Press. 2005.

Bernert Zs, Évinger S, Hajdu T. New data on the biological age estimation of children using bone measurements based on historical populations from the Carpathian Basin. Annales Historico-Naturales Musei Nationalis Hungarici. 2007; 99: 199–206.

Buikstra JE, Ubelaker D. Standards for data collection from human skeletal remains. Fayetteville, Arkansas: Arkansas archeological survey research series. 1994; 44.

Chirchir H, Kivell TL, Ruff CB, Hublin JJ, Carlson KJ, Zipfel B, Richmond BG. Recent origin of low trabecular bone density in modern humans. Proc Natl Acad Sci U S A. 2015 Jan 13;112(2):366–71. doi: 10.1073/pnas.1411696112. Epub 2014 Dec 22.

Farr JN, Amin S, Melton LJ 3rd, Kirmani S, McCready LK, Atkinson EJ, Müller R, Khosla S. Bone strength and structural deficits in children and adolescents with a distal forearm fracture resulting from mild trauma. J Bone Miner Res. 2014 Mar;29(3):590–9.

Florence JL. Linear and cortical bone dimensions as indicators of health status in subadults from the Milwaukee County Poor Farm Cemetery. M.A., University of Colorado at Denver. 2007.

Gabel L, Macdonald HM, McKay HA. Sex Differences and Growth-Related Adaptations in Bone Microarchitecture, Geometry, Density, and Strength from Childhood to Early Adulthood: A Mixed Longitudinal HR-pQCT Study. J Bone Miner Res. 2017;32(2):250–263. doi:10.1002/jbmr.2982

Gindhart PS. Growth standards for the tibia and radius in children aged one month through eighteen years. American Journal of Physical Anthropology. 1973; 39: 41–48.

[Hough M](https://www.ncbi.nlm.nih.gov/pubmed/?term=Hough%20M%5BAuthor%5D&cauthor=true&cauthor_uid=21427487), [Johnson P](https://www.ncbi.nlm.nih.gov/pubmed/?term=Johnson%20P%5BAuthor%5D&cauthor=true&cauthor_uid=21427487), [Rajon D](https://www.ncbi.nlm.nih.gov/pubmed/?term=Rajon%20D%5BAuthor%5D&cauthor=true&cauthor_uid=21427487), [Jokisch D](https://www.ncbi.nlm.nih.gov/pubmed/?term=Jokisch%20D%5BAuthor%5D&cauthor=true&cauthor_uid=21427487), [Lee C](https://www.ncbi.nlm.nih.gov/pubmed/?term=Lee%20C%5BAuthor%5D&cauthor=true&cauthor_uid=21427487), [Bolch W](https://www.ncbi.nlm.nih.gov/pubmed/?term=Bolch%20W%5BAuthor%5D&cauthor=true&cauthor_uid=21427487). An image-based skeletal dosimetry model for the ICRP reference adult male–internal electron sources. [Phys Med Biol.](https://www.ncbi.nlm.nih.gov/pubmed/21427487) 2011 Apr 21;56(8):2309–46. doi: 10.1088/0031-9155/56/8/001. Epub 2011 Mar 22.

Jeanty P. Fetal limb biometry. Radiology. 1983 May;147(2):601-2. doi: 10.1148/radiology.147.2.6836145. PMID: 6836145

Kawalilak CE, Bunyamin AT, Björkman KM, Johnston JD, Kontulainen SA. Precision of bone density and micro-architectural properties at the distal radius and tibia in children: an HR-pQCT study. Osteoporos Int. 2017 Nov; 28(11):3189–3197. 2011 doi: 10.1007/s00198-017-4185-y

[Kirmani S](https://www.ncbi.nlm.nih.gov/pubmed/?term=Kirmani%20S%5BAuthor%5D&cauthor=true&cauthor_uid=19113916), [Christen D](https://www.ncbi.nlm.nih.gov/pubmed/?term=Christen%20D%5BAuthor%5D&cauthor=true&cauthor_uid=19113916), [van Lenthe GH](https://www.ncbi.nlm.nih.gov/pubmed/?term=van%20Lenthe%20GH%5BAuthor%5D&cauthor=true&cauthor_uid=19113916), [Fischer PR](https://www.ncbi.nlm.nih.gov/pubmed/?term=Fischer%20PR%5BAuthor%5D&cauthor=true&cauthor_uid=19113916), [Bouxsein ML](https://www.ncbi.nlm.nih.gov/pubmed/?term=Bouxsein%20ML%5BAuthor%5D&cauthor=true&cauthor_uid=19113916), [McCready LK](https://www.ncbi.nlm.nih.gov/pubmed/?term=McCready%20LK%5BAuthor%5D&cauthor=true&cauthor_uid=19113916), [Melton LJ 3rd](https://www.ncbi.nlm.nih.gov/pubmed/?term=Melton%20LJ%203rd%5BAuthor%5D&cauthor=true&cauthor_uid=19113916), [Riggs BL](https://www.ncbi.nlm.nih.gov/pubmed/?term=Riggs%20BL%5BAuthor%5D&cauthor=true&cauthor_uid=19113916), [Amin S](https://www.ncbi.nlm.nih.gov/pubmed/?term=Amin%20S%5BAuthor%5D&cauthor=true&cauthor_uid=19113916), [Müller R](https://www.ncbi.nlm.nih.gov/pubmed/?term=M%C3%BCller%20R%5BAuthor%5D&cauthor=true&cauthor_uid=19113916), [Khosla S](https://www.ncbi.nlm.nih.gov/pubmed/?term=Khosla%20S%5BAuthor%5D&cauthor=true&cauthor_uid=19113916). Bone structure at the distal radius during adolescent growth. [J Bone Miner Res.](https://www.ncbi.nlm.nih.gov/pubmed/19113916) 2009 Jun;24(6):1033–42. doi: 10.1359/jbmr.081255.

[Kocijan R](https://www.ncbi.nlm.nih.gov/pubmed/?term=Kocijan%20R%5BAuthor%5D&cauthor=true&cauthor_uid=25956285), [Muschitz C](https://www.ncbi.nlm.nih.gov/pubmed/?term=Muschitz%20C%5BAuthor%5D&cauthor=true&cauthor_uid=25956285), [Haschka J](https://www.ncbi.nlm.nih.gov/pubmed/?term=Haschka%20J%5BAuthor%5D&cauthor=true&cauthor_uid=25956285), [Hans D](https://www.ncbi.nlm.nih.gov/pubmed/?term=Hans%20D%5BAuthor%5D&cauthor=true&cauthor_uid=25956285), [Nia A](https://www.ncbi.nlm.nih.gov/pubmed/?term=Nia%20A%5BAuthor%5D&cauthor=true&cauthor_uid=25956285), [Geroldinger A](https://www.ncbi.nlm.nih.gov/pubmed/?term=Geroldinger%20A%5BAuthor%5D&cauthor=true&cauthor_uid=25956285), [Ardelt M](https://www.ncbi.nlm.nih.gov/pubmed/?term=Ardelt%20M%5BAuthor%5D&cauthor=true&cauthor_uid=25956285), [Wakolbinger R](https://www.ncbi.nlm.nih.gov/pubmed/?term=Wakolbinger%20R%5BAuthor%5D&cauthor=true&cauthor_uid=25956285), [Resch H](https://www.ncbi.nlm.nih.gov/pubmed/?term=Resch%20H%5BAuthor%5D&cauthor=true&cauthor_uid=25956285). Bone structure assessed by HR-pQCT, TBS and DXL in adult patients with different types of osteogenesis imperfecta. [Osteoporos Int.](https://www.ncbi.nlm.nih.gov/pubmed/25956285) 2015 Oct;26(10):2431–40. doi: 10.1007/s00198-015-3156-4. Epub 2015 May 9.

[Lochmüller EM](https://www.ncbi.nlm.nih.gov/pubmed/?term=Lochm%C3%BCller%20EM%5BAuthor%5D&cauthor=true&cauthor_uid=18839046), [Kristin J](https://www.ncbi.nlm.nih.gov/pubmed/?term=Kristin%20J%5BAuthor%5D&cauthor=true&cauthor_uid=18839046), [Matsuura M](https://www.ncbi.nlm.nih.gov/pubmed/?term=Matsuura%20M%5BAuthor%5D&cauthor=true&cauthor_uid=18839046), [Kuhn V](https://www.ncbi.nlm.nih.gov/pubmed/?term=Kuhn%20V%5BAuthor%5D&cauthor=true&cauthor_uid=18839046), [Hudelmaier M](https://www.ncbi.nlm.nih.gov/pubmed/?term=Hudelmaier%20M%5BAuthor%5D&cauthor=true&cauthor_uid=18839046), [Link TM](https://www.ncbi.nlm.nih.gov/pubmed/?term=Link%20TM%5BAuthor%5D&cauthor=true&cauthor_uid=18839046), [Eckstein F](https://www.ncbi.nlm.nih.gov/pubmed/?term=Eckstein%20F%5BAuthor%5D&cauthor=true&cauthor_uid=18839046). Measurement of Trabecular Bone Microstructure Does Not Improve Prediction of Mechanical Failure Loads at the Distal Radius Compared with Bone Mass Alone Calcif Tissue Int. 2008; 83:293–299 DOI 10.1007/s00223-008-9172-z

Määttä M, Macdonald HM, Mulpuri K, McKay HA. Deficits in distal radius bone strength, density and microstructure are associated with forearm fractures in girls: an HR-pQCT study. Osteoporos Int. 2015;26(3):1163–1174. doi:10.1007/s00198-014-2994-9

Maquer G, Musy SN, Wandel J, Gross T, Zysset PK. Bone Volume Fraction and Fabric Anisotropy Are Better Determinants of Trabecular Bone Stiffness Than Other Morphological Variables. J Bone Miner Res. 2015; 30: 1000–1008. doi:[10.1002/jbmr.2437](https://doi.org/10.1002/jbmr.2437)

Maresh MM. Measurements from roentgenograms. In: Human Growth and Development (R.W. McCammon, Ed.). Springfield, IL: Charles C. Thomas. 1970; 157–200.

Medvedev MV Ed. Ultrasonic Fetometry: Reference Tables and Nomograms Ed. 8th, rev. Moscow: Real time Publisher. 2009; 19–24 (in Russian).

Mitchell DM, Caksa S, Yuan A, Bouxsein ML, Misra M, Burnett-Bowie SM. Trabecular Bone Morphology Correlates Wwith Skeletal Maturity and Body Composition in Healthy Adolescent Girls. J Clin Endocrinol Metab. 2018;103(1):336–345. doi:10.1210/jc.2017-01785

Normal pediatric bone X-ray. Accessed at: <https://bonexray.com/>; <http://bones.getthediagnosis.org/>

O'Brien CE, Com G, Fowlkes J, Tang X, James LP. Peripheral quantitative computed tomography detects differences at the radius in prepubertal children with cystic fibrosis compared to healthy controls. PLoS One. 2018 Jan 11;13(1).

[O'Reilly SE](https://www.ncbi.nlm.nih.gov/pubmed/?term=O'Reilly%20SE%5BAuthor%5D&cauthor=true&cauthor_uid=27897136), [DeWeese LS](https://www.ncbi.nlm.nih.gov/pubmed/?term=DeWeese%20LS%5BAuthor%5D&cauthor=true&cauthor_uid=27897136), [Maynard MR](https://www.ncbi.nlm.nih.gov/pubmed/?term=Maynard%20MR%5BAuthor%5D&cauthor=true&cauthor_uid=27897136), [Rajon DA](https://www.ncbi.nlm.nih.gov/pubmed/?term=Rajon%20DA%5BAuthor%5D&cauthor=true&cauthor_uid=27897136), [Wayson MB](https://www.ncbi.nlm.nih.gov/pubmed/?term=Wayson%20MB%5BAuthor%5D&cauthor=true&cauthor_uid=27897136), [Marshall EL](https://www.ncbi.nlm.nih.gov/pubmed/?term=Marshall%20EL%5BAuthor%5D&cauthor=true&cauthor_uid=27897136), [Bolch WE](https://www.ncbi.nlm.nih.gov/pubmed/?term=Bolch%20WE%5BAuthor%5D&cauthor=true&cauthor_uid=27897136). An image-based skeletal dosimetry model for the ICRP reference adult female-internal electron sources. [Phys Med Biol.](https://www.ncbi.nlm.nih.gov/pubmed/27897136) 2016 Dec 21;61(24):8794–8824. Epub 2016 Nov 29.

Pafundi D. Image-based skeletal tissues and electron dosimetry models for the ICRP reference pediatric age series. A dissertation presented to the graduate schools of the University of Florida in partial fulfillment of the requirements for the degree of doctor of the philosophy. University of Florida. 2009.

Quick JL, Ward KA, Adams JE, Mughal MZ. Cortical bone geometry in asthmatic children. Arch Dis Child. 2006;91(4):346–348.

Wang Q, Ghasem‐Zadeh A, Wang X, Iuliano‐Burns S, Seeman E. Trabecular bone of growth plate origin influences both trabecular and cortical morphology in adulthood. J Bone Miner Res. 2011; 26:1577–1583. doi:[10.1002/jbmr.360](https://doi.org/10.1002/jbmr.360)

Yang H, Yu A, Burghardt AJ, Virayavanich W, Link TM, Imboden JB, Li X. Quantitative characterization of metacarpal and radial bone in rheumatoid arthritis using high resolution‐ peripheral quantitative computed tomography. Int J Rheum Dis. 2017; 20: 353–362. doi:[10.1111/1756-185X.12558](https://doi.org/10.1111/1756-185X.12558)

Zhou B. Bone Quality Assessment Using High Resolution Peripheral Quantitative Computed Tomography (HR-pQCT). Theses Doctoral. Columbia University. 2015.
