# Supplementary material for: Stochastic parametric skeletal dosimetry model for humans: Anatomical-morphological basis and parameter evaluation
Source: PLoS One. 2025 Jul 2;20(7):e0327156. doi: 10.1371/journal.pone.0327156 (PMC12306906; doi:10.1371/journal.pone.0327156)
Supplement: S11 Sternum — (DOCX) [file pone.0327156.s011.docx]

**Sternum**

**Adults, analysis of published data on sternum macro-parameters and cortical thickness**

The human sternum consists of *body*, *manubrium* and small xiphoid process (not modeled). Two segments were allocated for sternum: body and manubrium (Fig. St1).

**Fig. St1**. Sternum (a) anterior view, (b) lateral view, (c) stylized models (BPS) describing sternal segments: (1) body, (2) manubrium.

1. *Body* was described by flat box with two sides equal 30 mm and *d_b_*-depth; cortical layer is located on top and bottom sides. Total surface area of sternal body was calculated as product of body length (l_b_) and width (w_sb_): *S_b_=* *l_b_·w_b_*
2. *Manubrium* was described by flat box with two sides equal 30 mm and *d_m_*-depth; cortical layer is located on top and bottom sides. Total surface area of sternal manubrium was calculated as product of manubrium length (l_m_) and width (w_sm_): *S_m_=l_m_·w_m_*

**Table St1.** Sternum macro-parameters for adult male and female according to Selthofer et al. 2006 (M± SD)

| Sex | Age | N | *d_b_* mm | *d_m_* mm | *S_b_* mm^2^ | *S_m_* mm^2^ |
| --- | --- | --- | --- | --- | --- | --- |
| Male | 65 | 55 | 10.0±1 | 13.0±2 | 3480±550 | 2898±348 |
| Female | 65 | 35 | 9.0±0.9 | 11±1.65 | 2614±314 | 2413±193 |

Comments: *d_m_* -the manubrium thickness was measured on two points which represent extreme values of manubrium thickness. The greatest thickness was measured at the intersection of the central longitudinal line and the line joining central points of clavicular notches. The least thickness was determined at the point where the central longitudinal line intersected manubrium at the middle of its length; *d_b_-*mean distance between anterior and posterior surfaces of the sternal body.

The values from Table St1 were assumed for BPSs

**Pre-adults, analysis of published data on sternum macro-parameters and cortical thickness**

The prenatal ossification of the sternum begins at the 5th month from the manubrium (Paterson 1904, Flecker 1932, Noback and Robertson 1951, Last 1973, Fazekas and Kósa 1978, Ogden 1979, England 1990). At the time of birth, the sternum consists of several centers of ossification (one in manubrium and 3−5 in the body) connected by cartilage. The final fusion of ossification centers into a single bone ends during puberty. In this regard, the mineralized parts of the sternum are modeled only starting at the age of 5 years. Only one stylized model was used to describe the sternum both body and manubrium of pre adults. BPS is the box of height *h* (averaged thickness *h_m_* and *h_b_*) and sides 30×30 mm; the cortical layer covers the upper and lower sides. Published data on cortical thickness, manubrium thickness and sternum ossification are summarized in Tables St2-St4.

**Fig. St2.** Development of the sternum (anterior view) according to Cunningham et al. (2016) with modification. Manubrium and body ossification centers are indicted.

**Table St 2.** Cortical thickness of sternum, mm

| Author | Age | N | Body | | Manubrium | |
| --- | --- | --- | --- | --- | --- | --- |
|  |  |  | M | SD | M | SD |
| Jonathon et al. 2017 | 48–92 | 4 | 1.15 | 0.5 | - | - |
| Denst et al. 1950 | 40–80 | 67 | 1.0 | - | - | - |
| Wang et al. 2011 | 17–45 | 64 | - | - | 1.45 | 0.32 |
| **Assumed for BPS (CV%)** | | | **1.1 (42)** | | **1.45 (22)** | |

**Table St3.** Published data on manubrium thickness according to Bayaroğulları et al. (2014), mm.

| Age | Range | N | M | SD |
| --- | --- | --- | --- | --- |
| 2.5 | 0−5 | 50 | 7.0 | 1.1 |
| 8 | 6−10 | 50 | 8.7 | 1.1 |
| 13 | 11−15 | 50 | 11.0 | 1.7 |
| 18 | 16−20 | 50 | 13.5 | 1.9 |

**Table St4.** Published data on sternum ossification area, mm^2^

| Author | Age | n | Sternal area |
| --- | --- | --- | --- |
| Riach 1967 | 0 | 12 | 191.5 |
| Riach 1967 | 1 | 1 | 553.0 |
| Riach 1967 | 3 | 1 | 986.0 |
| Cunningham et al. 2016* | 3 | 1 | 1355.0 |
| Riach 1967 | 4 | 1 | 189.0 |
| Riach 1967 | 6 | 1 | 815.0 |
| Cunningham et al. 2016* | 8 | 1 | 1563.0 |
| Weaver et al. 2014* | 10 | 2 | 3690.0 |

* the images were measured by us.

Values of manubrium thickness for reference ages were obtained by linear interpolation of estimates from Table St3. To obtain the sternum body thickness, it was assumed that the thickness ratio body/manubrium in children is similar to those for adult. Thus, the adult ratio 0.77 was used for estimation of sternal-body thickness. The estimates are shown in Table St5.

**Table St5.** Assumed parameters for sternal BPS, mm.

| Age | Thickness of manubrium (*h_stm_*) | | Thickness of body (*h_stb_*) | | Average thickness *(h)* | | Ct.Th | | Sternal area, mm^2^ |
| --- | --- | --- | --- | --- | --- | --- | --- | --- | --- |
|  | M | SD | M | SD | M | SD | M | SD |  |
| 5 | 7.8 | 1.0 | 6.0 | 0.8 | 6.9 | 0.9 | 0.14 | 0.26 | 1622.5 |
| 10 | 9.6 | 1.5 | 7.4 | 1.2 | 8.5 | 1.3 | 0.65 | 0.4 | 3960.0 |
| 15 | 12.0 | 1.7 | 9.2 | 1.3 | 10.6 | 1.5 | 0.91 | 0.4 | 6378.0* |

*The sternum area for 15-year-olds was taken to be the same as for adults (based on the pictures from Cunningham et al. 2016 and Weaver et al. 2014.

**Analysis of published data on sternum microstructures**

We did not find data on the microstructure of sternum for children, but the trabecular structure of the adult sternum is well studied (Table St6). The following age-dynamic for sternum was assumed (similar with vertebra dynamic): BV/TV has maximal values (twice as high as in adults) in newborns, and then declines by age 1 year to adult levels. In contrast, minimal Tb.Th value (15% lower than adults) is assumed for newborns; and then Tb.Th increases to an adult level by the age of 10 years. Assumed values of trabecular micro-parameters are summarized in Table St7.

**Table St6**. Parameters of microstructure of sternum for combined samples male and female, published data (mean±SD, mm).

| Author | Age | N | Tb.Th | Tb.Sp | BV/TV |
| --- | --- | --- | --- | --- | --- |
| Baur-Melnyk 2012 | Adult | 100 | 0.15±0.05 | - | 0.15±0.04 |
| Bartl et al 1993 | Adult | 100 | 0.155±0.04 | - | 0.15±0.04 |
| Arbabi 2009 | 39 | 2 | 0.224±0.02 | 1.36±0.12 | 0.138 |
|  | 55 | 2 | 0.176±0.01 | 1.5±0.12 | 0.10 |

**Table St7.** Micro-parameters assumed for sternal BPSs.

| Age | BV/TV  (min-max) | SD BV/TV | Tb.Th, mm  (min-max) | SD Tb.Th | Tb.Sp, mm  (min-max) | SD Tb.Sp |
| --- | --- | --- | --- | --- | --- | --- |
| 5 | 0.149  (0.069−0.229) | 0.04 | 0.135  (0.055−0.215) | 0.04 | 1.00  (0.880−1.120) | 0.06 |
| 10 | 0.149  (0.069−0.229) | 0.04 | 0.150  (0.05−0.25) | 0.05 | 1.00  (0.880−1.120) | 0.06 |
| 15 | 0.149  (0.069−0.229) | 0.04 | 0.150  (0.05−0.25) | 0.05 | 1.00  (0.880−1.120) | 0.06 |
| Adults | 0.149  (0.08−0.22) | 0.04 | 0.150  (0.05−0.25) | 0.05 | 1.00  (0.880−1.120) | 0.06 |

**References for sternum**

Arbabi A. A quantitative analysis of the structure of human sternum. J Med Phys. 2009; 34(2): 80–86.

Bartl R, Frisch B. Biopsy of bone in internal medicine – an atlas and sourcebook. Kluwer Academic Publishers, Dordrecht. London.1993.

Baur-Melnyk A. Magnetic Resonance Imaging of the Bone Marrow. Springer Science & Business Media. 2012.

Bayaroğulları H, Yengil E, Davran R, Ağlagül E, Karazincir S, Balcı A. Evaluation of the postnatal development of the sternum and sternal variations using multidetector CT. Diagn Interv Radiol. 2014 Jan-Feb;20(1):82–9.

Cunningham C, Scheuer L, Black S. Developmental Juvenile Osteology. 2 ed. London: Academic Press. 2016; 622.

Denst J, Mulligan RM. The Distribution of Bone Marrow in the Human Sternum. American Journal of Clinical Pathology. 1950; 20(7): 610–613.

England MA. A Colour Atlas of Life Before Birth. London: Wolfe. 1990.

Fazekas IGy, Kósa, F. Forensic Fetal Osteology. Budapest: Akadémiai Kiadó. 1978.

Flecker H. Roentgenographic observations of the human skeleton prior to birth. Medical Journal of Australia. 1932; 19: 640–643.

Jonathon L, Lonnie D, Seligson D, Seid W, Voor M. Anatomy of the Sternum: A Micro-CT Study to Guide the Fixation of Sternal Fractures. Biol Eng Med. 2017; 2(2): 1–4.

Last RJ. Anatomy, Regional and Applied (fifth ed.). Edinburgh: Churchill Livingstone. 1973.

Noback CR, Robertson GG. Sequences of appearance of ossification centres in the human skeleton during the first five prenatal months. American Journal of Anatomy. 1951; 89: 1–28.

Ogden JA. The development and growth of the musculo-skeletal system. In: Scientific Basis of Orthopaedics (J.A. Albright and R. Brands, Eds.). New York: Appleton-Century-Crofts. 1979; 41–103.

Paterson AM. The Human Sternum. London: Williams and Norgate. 1904.

Riach IC. Ossification in the sternum as a means of assessing skeletal age. J Clin Pathol. 1967;20(4):589–590.

Selthofer R, Nikolić V, Mrcela T, Radić R, Leksan I, Rudez I, Selthofer K. Morphometric analysis of the sternum. Coll Antropol. 2006; 30(1):43–47.

Wang GJ, Wang Q, Wang S, Zhong DJ, W Z, Wen R. Feasibility of manubrium sterni cancellous bone plus pyramesh in anterior cervical spinal fusion surgery. Journal of Clinical Rehabilitative Tissue Engineering Research. 2011; 15(9):1698-1701.

Weaver AA, Schoell SL, Nguyen CM, Lynch SK, Stitzel JD. Morphometric analysis of variation in the sternum with sex and age. J Morphol. 2014 Nov; 275(11):1284−99.
