# Supplementary material for: Stochastic parametric skeletal dosimetry model for humans: Anatomical-morphological basis and parameter evaluation
Source: PLoS One. 2025 Jul 2;20(7):e0327156. doi: 10.1371/journal.pone.0327156 (PMC12306906; doi:10.1371/journal.pone.0327156)
Supplement: S12 Scapula — (DOCX) [file pone.0327156.s012.docx]

**Scapula**

**Pre-adults, analysis of published data on scapula macro-parameters and cortical thickness**

The scapula is a flat bone of a triangular shape (Fig. Sc1). The main body of the juvenile scapula is recognizable and close to adult form by 12 to 13 prenatal weeks, and newborns have a scapular bone mineralized to a large extent. At birth, the scapula has cartilage in the region of the process, articular cavity, vertebral edge, and also in the upper and lower corners of the scapula. Thus, the child’s scapula is more rounded than in adults. Fig. Sc1 shows the main parts of the pre-adult scapula for children aged 0 and 5 years: *central part or body; glenoid part; lateral margin; acromion process; coracoid process* (separate center of ossification, not modelled); as well as measurements usually performed during anatomical and morphological studies. The scapula is modeled by a set of geometric models (Fig. Sc1 f, g, h, e).

**Fig. Sc1.** Images of scapula of different ages based on Schaefer et al. (2004); Schwartz (2011), posterior, anterior and lateral view: (a-c) perinatal scapula; (d, e) – children of 5-years; (f-i) stylized models for scapula. Panel (f) shows the box describing lateral margin; panel (g) presents cylinder describing glenoid part; panel (h) shows box describing acromion; panel (i) presents the box describing central part. The main measured parameters are shown in numbers, their designations are deciphered in the text.

The main measured parameters are shown in Fig. Sc1 with numbers, which are deciphered below:

1. Scapular width (syn. breath) (*S_w_*): Distance between the margin of the glenoid fossa and the medial end of the spine;

2. Scapular length (*S_l_*): Distance between the superior and inferior angles of the scapula;

3. Infra-scapular height (*I_h_*): Distance between the point at which the axis of the spine intersects the medial border of the scapula to the inferior angle;

4. Supra-scapular height (*Su_h_*): Distance between the point at which the axis of the spine intersects the medial border of the scapula to the superior angle;

5. Spine length (*Sp_l_*): Maximum distance between medial end of spine and tip of acromion process;

6. Acromial width (*W_a_*): Maximum distance between the anterior and posterior borders of the acromion process, taken perpendicular to the axis of the spine; Acromial length *L_a_* (not shown): distance between the lateral and medial borders at the midpoint of the acromion process

7. Length of glenoidal surface (*D1_g_*): Maximum distance between the superior and inferior borders of the glenoid articular surface (does not include the articular surface for the coracoid);

8. Length of glenoidal mass (*D_1g_*): Distance between the superior border of the articulation site for the coracoid process and the inferior border of the glenoid surface (not used in current study);

9. Middle diameter of the glenoidal surface (*D_2g_*): Distance from the middle of the posterior border of the glenoid rim to the anterior border, perpendicular to the length of glenoidal surface;

10. Thickness of the lateral margin (*W_lm_*): Thickness of the border at the midpoint between the inferior margin of the glenoid prominence and the inferior angle. The measurement should be taken perpendicular to the scapular body.

11 Scapula body thickness (*S_bh_*): mean distance between the anterior and posterior sides of scapulae body (not indicated in the Fig. Sc1).

Stylized models for scapula shown in Fig. Sc1. Set of models for scapula of 0-Y and 1-Y consist of three BPSs: glenoid part, acromion; scapular body. For other ages, 3 stylized models are also used, but the BPS for scapular-body is replaced by the BPS-lateral margin, where active bone marrow remains at this age.

BPS1 *Glenoid*- was stylized by elliptic cylinder of major axes (diameter) *D1_g_*, and *D2_g_*, and height *h_g_* (distance between spino-glenoid notch and articular surface of glenoid). Cortical layer covers the lateral surface (simulated for all ages).

BPS2 *Acromion* was described by a box of *W_a_* (acromion width), *L_a_* (acromion length), *h_a_* (acromion thickness). The cortical layer is located on all sides, except for the side adjacent to the scapula. (simulated for all ages).

BPS3 *Scapula body* was described by a box of height *S_bh_* and sides 30×30 mm. The cortical layer is located on two larger (anterior and posterior) sides). The total flat area of the scapula was also estimated based on the values *S_w_*, *S_l_*, *I_h_*, *Su_h_*, *Sp_l_* (simulated for ages 0-Y and 1-Y).

BPS4 *Lateral margin* was described by a box of width *W_lm_*; height *h_lm_*(30 mm); *l_lm_*-depth. The cortical layer is located on anterior, posterior and lateral sides of BPS (simulated for ages of 5-Y, 10-Y, 15-Y).

Tables Sc1–Sc8 present published data on pre-adult scapula measurements. Table Sc9 summarizes averaged values of pre-adult scapula measurements used for modeling.

**Table Sc1.** Published data on scapular width of pre-adults (mm) (*S_w_)*.

| Author | Age | N | M | SD |
| --- | --- | --- | --- | --- |
| Fazekas et al. 1978 | 40 w | - | 29.5 | - |
| Corrigan 1960 | 0 | 60 | 34.0 | 1.0 |
| Hrdlicka 1942 | 0 | - | 28.6 | - |
| Vallois 1946 | 0 | - | 34.0 | - |
| Saunders et al. 1993 | 0 (0–0.5) | 7 | 31.1 | - |
| Saunders et al. 1993 | 0.75 (0.5–1) | 16 | 37 | - |
| Saunders et al. 1993 | 1.5 (1–2) | 19 | 43.3 | - |
| Cardoso et al. 2017 | <2 | 29 | 32.7 | 6.1 |
| Badr El Dine et al. 2015 | <2 | 13 | 51 | 13.5 |
| Badr El Dine et al. 2015 | 2 | 6 | 52.9 | 8.3 |
| Vallois 1946 | 2 | - | 46.0 | - |
| Rissech et al. 2007 | 2 (0–4) | 15 | 36.6 | 12.1 |
| Saunders et al. 1993 | 2.5 (2–3) | 8 | 59.8 | - |
| Vallois 1946 | 3 (2–4) | - | 45.0 | - |
| Saunders et al. 1993 | 3.5 (3–4) | 5 | 56 | - |
| Badr El Dine et al. 2015 | 4 | 8 | 66.8 | 6.2 |
| Saunders et al. 1993 | 4.5 (4–5) | 3 | 56.8 | - |
| Vallois 1946 | 5 (4–6) | - | 54.0 | - |
| Saunders et al. 1993 | 5.5 (5–6) | 3 | 61.8 | - |
| Badr El Dine et al. 2015 | 6 | 4 | 74.5 | 4 |
| Saunders et al. 1993 | 6.5 (6–7) | 7 | 66.1 | - |
| Rissech et al. 2007 | 6.5 (5–8) | 8 | 55.6 | 6.7 |
| Vallois 1946 | 7 (6–8) | - | 61.0 | - |
| Saunders et al. 1993 | 7.5 (7–8) | 2 | 63.3 | - |
| Badr El Dine et al. 2015 | 8 | 3 | 85.6 | 2.6 |
| Saunders et al. 1993 | 8.5 (8–9) | 1 | 82.5 | - |
| Saunders et al. 1993 | 9.5 (9–10) | 2 | 77.3 | - |
| Badr El Dine et al. 2015 | 10 | 12 | 85.6 | 2.6 |
| Saunders et al. 1993 | 10.5 (10–11) | 2 | 87.3 | - |
| Rissech et al. 2007 | 10.5 (9–12) | 8 | 71.6 | 5.4 |
| Saunders et al. 1993 | 11.5 (11–12) | 1 | 82 | - |
| Badr El Dine et al. 2015 | 12 | 6 | 92.7 | 3.3 |
| Badr El Dine et al. 2015 | 14 | 6 | 92.7 | 3.4 |
| Rissech et al. 2007 | 14.5 (13–16) | 5 | 87.3 | 10 |
| Badr El Dine et al. 2015 | 16 | 9 | 101.6 | 9.4 |
| Rissech et al. 2007 (male) | 18 (17–19) | 6 | 94.1 | 2.8 |
| Rissech et al. 2007 (female) | 18 (17–19) | 6 | 92 | 8.9 |

**Table Sc2.** Published data on scapular length of pre-adults (mm) (*S_l_*).

| Author | Age | N | M | SD |
| --- | --- | --- | --- | --- |
| Fazekas et al. 1978 | 0 | - | 35.5 | - |
| Corrigan 1960 | 0 | 60 | 44.0 | 5.6 |
| Hrdlicka 1942 | 0 | an | 34.8 | - |
| Vallois 1946 | 0 | an | 46.5 | - |
| Saunders et al. 1993 | 0 (0–0.5) | 1 | 39.3 | - |
| Saunders et al. 1993 | 0.75 (0.5–1) | 15 | 49.2 | - |
| Saunders et al. 1993 | 1.5 (1–2) | 19 | 60.4 | - |
| Cardoso et al. 2017 | <2 | 30 | 45.7 | 11.1 |
| Badr El Dine et al. 2015 | <2 | 13 | 74.5 | 15.2 |
| Badr El Dine et al. 2015 | 2 | 6 | 78.7 | 9.5 |
| Vallois 1946 | 2 | - | 61.0 | - |
| Rissech et al. 2007 | 2 (0–4) | 15 | 51.8 | 21.2 |
| Saunders et al. 1993 | 2.5 (2–3) | 10 | 67.8 | - |
| Vallois 1946 | 3 (2–4) | - | 66.5 | - |
| Saunders et al. 1993 | 3.5 (3–4) | 5 | 63.9 | - |
| Badr El Dine et al. 2015 | 4 | 8 | 87.9 | 4.0 |
| Saunders et al. 1993 | 4.5 (4–5) | 3 | 81 | - |
| Vallois 1946 | 5 (4–6) | - | 80.0 | - |
| Saunders et al. 1993 | 5.5 (5–6) | 3 | 91.7 | - |
| Badr El Dine et al. 2015 | 6 | 4 | 94.2 | 4.0 |
| Saunders et al. 1993 | 6.5 (6–7) | 6 | 97.3 | - |
| Rissech et al. 2007 | 6.5 (5–8) | 6 | 82.9 | 10 |
| Vallois 1946 | 7 (6–8) | - | 91.0 | - |
| Saunders et al. 1993 | 7.5 (7–8) | 1 | 94 | - |
| Badr El Dine et al. 2015 | 8 | 3 | 103.6 | 3.1 |
| Saunders et al. 1993 | 8.5 (8–9) | 1 | 117 | - |
| Saunders et al. 1993 | 9.5 (9–10) | 2 | 120 | - |
| Badr El Dine et al. 2015 | 10 | 12 | 103.6 | 3.1 |
| Saunders et al. 1993 | 10.5 (10–11) | 1 | 121 | - |
| Rissech et al. 2007 | 10.5 (9–12) | 5 | 109.2 | 9.5 |
| Saunders et al. 1993 | 11.5 (11–12) | 1 | 121 | - |
| Badr El Dine et al. 2015 | 12 | 6 | 110.7 | 1.4 |
| Badr El Dine et al. 2015 | 14 | 6 | 111.0 | 1.5 |
| Rissech et al. 2007 | 14.5 (13–16) | 5 | 130 | 13.4 |
| Badr El Dine et al. 2015 | 16 | 9 | 126.0 | 10.6 |
| Rissech et al. 2007 (male) | 18 (17–19) | 4 | 149.1 | 7 |
| Rissech et al. 2007 (female) | 18 (17–19) | 5 | 134.9 | 5.6 |

**Table Sc3.** Published data on infra-scapular height of pre-adults (mm) (*I_h_*).

| Author | Age | N | Mean | SD |
| --- | --- | --- | --- | --- |
| Hrdlicka 1942 | 0 | - | 28.6 | - |
| Corrigan 1960 | 0 | 60 | 35.0 | 5.2 |
| Badr El Dine et al. 2015 | <2 | 13 | 60.9 | 15.1 |
| Badr El Dine et al. 2015 | 2 | 6 | 65.3 | 11.1 |
| Rissech et al. 2007 | 2 (0–4) | 15 | 41.6 | 15.2 |
| Badr El Dine et al. 2015 | 4 | 8 | 80.8 | 5.1 |
| Badr El Dine et al. 2015 | 6 | 4 | 84.5 | 4.4 |
| Rissech et al. 2007 | 6.5 (5–8) | 6 | 66.4 | 9 |
| Badr El Dine et al. 2015 | 8 | 3 | 89.1 | 1.5 |
| Badr El Dine et al. 2015 | 10 | 12 | 88.9 | 1.6 |
| Rissech et al. 2007 | 10.5 (9–12) | 5 | 84.5 | 8.3 |
| Badr El Dine et al. 2015 | 12 | 6 | 94.9 | 3.1 |
| Badr El Dine et al. 2015 | 14 | 6 | 95.2 | 3.3 |
| Rissech et al. 2007 | 14.5 (13–16) | 5 | 99.1 | 7.1 |
| Badr El Dine et al. 2015 | 16 | 9 | 105.7 | 5.8 |
| Rissech et al. 2007 (male) | 18 (17–19) | 6 | 114.4 | 4 |
| Rissech et al. 2007 (female) | 18 (17–19) | 5 | 98.8 | 4.1 |

**Table Sc4.** Published data on supra-scapular height of pre-adults (mm) (*Su_h_*).

| Author | Age | N | Mean | SD |
| --- | --- | --- | --- | --- |
| Corrigan 1960 | 0 | 60 | 17.0 | 5.5 |
| Badr El Dine et al. 2015 | <2 | 13 | 17.6 | 3.3 |
| Badr El Dine et al. 2015 | 2 | 6 | 18.8 | 2.2 |
| Rissech et al. 2007 | 2 (0–4) | 15 | 18.7 | 6.6 |
| Badr El Dine et al. 2015 | 4 | 8 | 21.2 | 2.8 |
| Badr El Dine et al. 2015 | 6 | 4 | 24.5 | 4.1 |
| Rissech et al. 2007 | 6.5 (5–8) | 8 | 25.7 | 2 |
| Badr El Dine et al. 2015 | 8 | 3 | 28.8 | 2.3 |
| Badr El Dine et al. 2015 | 10 | 12 | 27.8 | 1.8 |
| Rissech et al. 2007 | 10.5 (9–12) | 7 | 33.4 | 2.2 |
| Badr El Dine et al. 2015 | 12 | 6 | 32.6 | 1.9 |
| Badr El Dine et al. 2015 | 14 | 6 | 32.5 | 1.9 |
| Rissech et al. 2007 | 14.5 (13–16) | 5 | 38.9 | 10.3 |
| Badr El Dine et al. 2015 | 16 | 9 | 40.2 | 3.6 |
| Rissech et al. 2007 (male) | 18 (17–19) | 4 | 43.9 | 4.7 |
| Rissech et al. 2007 (female) | 18 (17–19) | 6 | 45.6 | 6.7 |

**Table Sс5.** Published data on spinal length of pre-adults (mm) (*Sp_l_*).

| Author | Age | N | Mean | SD |
| --- | --- | --- | --- | --- |
| Corrigan 1960 | 0 | 60 | 42.0 | 5.3 |
| Cardoso et al. 2017 | <2 | 23 | 38.3 | 8.3 |
| Badr El Dine et al. 2015 | <2 | 13 | 62.5 | 18.9 |
| Rissech et al. 2007 | 2 (0–4) | 15 | 43.6 | 15.5 |
| Badr El Dine et al. 2015 | 2 | 6 | 69.6 | 12.4 |
| Badr El Dine et al. 2015 | 4 | 8 | 79.8 | 3.9 |
| Badr El Dine et al. 2015 | 6 | 4 | 86.3 | 5.2 |
| Rissech et al. 2007 | 6.5 (5–8) | 8 | 70.9 | 9.3 |
| Badr El Dine et al. 2015 | 8 | 3 | 93.4 | 1.9 |
| Badr El Dine et al. 2015 | 10 | 12 | 92.9 | 1.9 |
| Rissech et al. 2007 | 10.5 (9–12) | 4 | 92.6 | 11.4 |
| Badr El Dine et al. 2015 | 12 | 6 | 107.7 | 3.1 |
| Badr El Dine et al. 2015 | 14 | 6 | 107.3 | 3.3 |
| Rissech et al. 2007 | 14.5 (13–16) | 5 | 116.0 | 12.1 |
| Badr El Dine et al. 2015 | 16 | 9 | 121.6 | 9.9 |
| Rissech et al. 2007 (male) | 18 (17–19) | 6 | 126.7 | 8 |
| Rissech et al. 2007 (female) | 18 (17–19) | 6 | 122.8 | 16.4 |

**Table Sc6.** Published data on acromion width of pre-adults (mm) (*W_a_*).

| Author | Age | N | M | SD |
| --- | --- | --- | --- | --- |
| Corrigan 1960 | 0 | 60 | 16.0 | 3.3 |
| Badr El Dine et al. 2015 | <2 | 13 | 16 | 6.6 |
| Rissech et al. 2007 | 2 (0–4) | 16 | 7.6 | 2.5 |
| Badr El Dine et al. 2015 | 2 | 6 | 17.7 | 4.9 |
| Badr El Dine et al. 2015 | 4 | 8 | 24.4 | 3.6 |
| Badr El Dine et al. 2015 | 6 | 4 | 28 | 2.3 |
| Rissech et al. 2007 | 6.5 (5–8) | 9 | 12.9 | 1.7 |
| Badr El Dine et al. 2015 | 8 | 3 | 30.4 | 0.9 |
| Badr El Dine et al. 2015 | 10 | 12 | 30.4 | 0.8 |
| Rissech et al. 2007 | 10.5 (9–12) | 5 | 18.1 | 5.2 |
| Badr El Dine et al. 2015 | 12 | 6 | 32.9 | 1.7 |
| Badr El Dine et al. 2015 | 14 | 6 | 33.2 | 1.6 |
| Rissech et al. 2007 | 14.5 (13–16) | 5 | 32.3 | 8.7 |
| Badr El Dine et al. 2015 | 16 | 9 | 31.9 | 2.6 |
| Rissech et al. 2007 (male) | 18 (17–19) | 6 | 38.7 | 2.7 |
| Rissech et al. 2007 (female) | 18 (17–19) | 5 | 34.7 | 7.4 |

Acromial length (*L_a_*): distance between the lateral and medial borders at the midpoint of the acromion process was measured by Corrigan 1960 (n=60) for newborn: *L_a_* =13.0±2.2

**Table Sc7.** Published data on scapula lateral-margin-thickness (*W_lm_*) according to Badr El Dine et al. (2015) (mm).

| Age | n | M | SD |
| --- | --- | --- | --- |
| <2 | 13 | 3.0 | 0.4 |
| 2 | 6 | 3.1 | 0.4 |
| 4 | 8 | 3.2 | 0.2 |
| 6 | 4 | 3.1 | 0.2 |
| 8 | 3 | 3.5 | 0.1 |
| 10 | 12 | 3.5 | 0.1 |
| 12 | 6 | 3.3 | 0.1 |
| 14 | 6 | 3.3 | 0.1 |
| 16 | 9 | 3.3 | 0.2 |

**Table Sc8.** Published data on scapula glenoid length (*D_1g_*) and middle diameter (*D_2g_*) of pre-adults (mm).

| Author | Age | N | *D_1g_* | SD | *D_2g_* | SD |
| --- | --- | --- | --- | --- | --- | --- |
| Corrigan 1960 | 0 | 60 | 10 | 2.1 | 7.6 | 1.4 |
| Badr El Dine et al. 2015 | <2 | 13 | 20.2 | 4.4 | 16.3 | 5.6 |
| Cardoso et al. 2017 | <2 | 50 | - | - | 8.7 | 2.3 |
| Cardoso et al. 2017 | <2 | 40 | 16.6 | 4.7 | - | - |
| Rissech et al. 2007 | 2 (0–4) | 19 | 13.9 | 4.3 | 9.7 | 3.7 |
| Badr El Dine et al. 2015 | 2 | 6 | 21.7 | 2.9 | 18.3 | 3.8 |
| Badr El Dine et al. 2015 | 4 | 8 | 26.7 | 2.4 | 18.9 | 0.9 |
| Badr El Dine et al. 2015 | 6 | 4 | 27.9 | 3.2 | 20.1 | 0.8 |
| Rissech et al. 2007 | 6.5 (5–8) | 9 | 21.7 | 2.8 | 15.1 | 1.9 |
| Badr El Dine et al. 2015 | 8 | 3 | 29 | 1.2 | 22.2 | 0.8 |
| Badr El Dine et al. 2015 | 10 | 12 | 28.8 | 1.2 | 22 | 1.6 |
| Rissech et al. 2007 | 10.5 (9–12) | 10 | 26.2 | 1.6 | 17.7 | 1 |
| Badr El Dine et al. 2015 | 12 | 6 | 28.3 | 2.7 | 23.6 | 1.6 |
| Badr El Dine et al. 2015 | 14 | 6 | 28.8 | 2.7 | 23.8 | 4 |
| Rissech et al. 2007 | 14.5 (13–16) | 5 | 31.6 | 3 | 19.7 | 5 |
| Badr El Dine et al. 2015 | 16 | 9 | 31.7 | 4.2 | 23.4 | 3.1 |
| Rissech et al. 2007 | 18 (17–19) | 6 m | 36.3 | 2.5 | 25 | 0.1 |
| Rissech et al. 2007 | 18 (17–19) | 6 f | 32.4 | 2.1 | 21.4 | 1.9 |

**Table Sc9.** Averaged values of measured scapula parameters used in modeling (mm).

| Para-meter | 0-Y | | 1-Y | | 5-Y | | 10-Y | | 15-Y | |
| --- | --- | --- | --- | --- | --- | --- | --- | --- | --- | --- |
|  | M | SD | M | SD | M | SD | M | SD | M | SD |
| *I_h_* | 31.8 | 5.2 | 53.2 | 13.8 | 76.8 | 6.2 | 87.6 | 3.6 | 100.9 | 5.4 |
| *S_l_* | 43.7 | 5.3 | 62.7 | 15.3 | 85.2 | 10 | 108.4 | 6.3 | 122.5 | 8.6 |
| *S_w_* | 33.6 | 1 | 42.7 | 11.3 | 65.4 | 5.1 | 80.4 | 4 | 93.9 | 7.6 |
| *Sp_l_* | 42 | 5.3 | 48.5 | 13 | 77.5 | 6.3 | 92.8 | 4.3 | 115.9 | 8.5 |
| *Su_h_* | 17 | 5.5 | 18.3 | 4.6 | 23.7 | 2.7 | 30 | 1.9 | 37.6 | 4.8 |
| *W_a_* | 16 | 2.2 | 16 | 6.6 | 20.2 | 2.5 | 26.8 | 2.1 | 32.4 | 1.3 |
| *D_1g_* | 10 | 2.1 | 17.5 | 3.1 | 25.4 | 2.8 | 27.5 | 1.4 | 30.7 | 3.3 |
| *D_2g_* | 7.6 | 1.4 | 10.2 | 3 | 18 | 1.2 | 19.85 | 1.3 | 22.3 | 4 |
| *W_lm_* | - | - | - | - | 3.2 | 0.2 | 3.5 | 0.1 | 3.5 | 0.1 |

*Estimation of the area of the flat part of scapula for ages 0 and 1 year*

The body area of the scapula was calculated in the program *IpSquare v5.0*. To do this, we constructed images of the flat part of the scapula (without the glenoid part) in a 1:1 scale based on measurements from Table Sc9. After this, the images were loaded into the program, where the total area of scapular flat part was calculated. It was found:

0-Y Scapular area = 712 mm^2^

1-Y Scapula area = 858 mm^2^

As shown, the actual scapular area is close to the area of the stylized segment used for estimating DFs for large flat bones (30 × 30 = 900 mm^2^). For the calculation of DFs using Monte Carlo method this difference in areas was not considered; however, this difference in areas was considered when assessing the weight contribution of AM.

*Estimation of the thickness of the scapula body for the age of 0 and 1 year (S_bh_)*

We did not find data on the thickness of the scapula body *(S_bh_)* for children. However, the thickness of lateral margin *W_lm_* (Table Sc6) is known for children;

For children of 0–1Y *W_lm_* = 3.0 ± 0.4 mm.

In addition, we know the values of these parameters for adults:

*S_bh_* = 4.1 ± 0.6 mm (Burke et al. 2006; n = 18); *W_lm_* = 4.6 ± 0.5 mm

We hypothesized that the *S_bh_*/*W_lm_* ratio is the same for children and adults. Based on this assumption, we calculated *S_bh_* for children:

For adults, *S_bh_*/*W_lm_* = 4.1 / 4.6 = 0.9

For children, *S_bh_* = 0.9 × *W_lm_* = 0.9 × 3.0 = 2.7 mm

CV for children's *S_bh_* was taken as for *W_lm_* 13%

Table Sc10 presents the BPS-parameters for reference ages.

**Adults, analysis of published data on scapula macro-parameters and cortical thickness**

A significant part of adult scapula is thin and does not contain spongiosa. The following structures containing significant portions of spongiosa were selected as representative for scapula hemopoietic sites (Fig. 2.7): *glenoid, acromion, coracoid, and lateral margin*:

The following segments were allocated for scapula:

1. *Glenoid* was stylized by elliptic cylinder of major axes (diameter) *d1_g_*, and *d2_g_*, and height *h_g_* (distance between spino-glenoid notch and articular surface of glenoid). Cortical layer covers the lateral surface and one base of the cylinder.
2. *Acromion* was described by a box of *l_a_*-length (distance between tip and midpoint of posterior border of acromion process); *w_a_*-width (mean thickness of acromion process); *h_a_*-height (distance between the lateral and medial borders at the midpoint of the acromion process), the cortical layer is located on all sides, except for the side adjacent to the scapula.
3. *Lateral margin* was described by a box of *l_l_*-depth (derived from published CT-images and anatomical pictures); *w_l_*-width (thickness of the border at the midpoint between the inferior margin of the glenoid prominence and the inferior angle); *h_l_*-height =30 mm; total *hl_tot_* was derived from published CT-images and anatomical pictures. The cortical layer is located on anterior, posterior and lateral sides of BPS.

**Table Sc10.** BPS macro-parameters and cortical thickness assumed for scapula (mm).

| BPS | Para-meter | Rationale | 0 Y | | 1 Y | | 5-Y | | 10-Y | | 15-Y | | Adults | |
| --- | --- | --- | --- | --- | --- | --- | --- | --- | --- | --- | --- | --- | --- | --- |
|  |  |  | M | SD | M | SD | M | SD | M | SD | M | SD | M | SD |
| #1 Glenoid | *h_g_* | 16% (0–1 Y); 18% (5–10 Y) of *S_w_* ^a^;=H_g_ for adults | 5.4 | 0.2 | 6.8 | 1.8 | 11.8 | 0.9 | 14.5 | 0.7 | 16.9 | 1.4 | 20 | 2 |
| #1 Glenoid | *D_1g_* | Measured values (Table Sc9) | 10.0 | 2.1 | 17.5 | 3.1 | 25.4 | 2.8 | 27.5 | 1.4 | 30.7 | 3.3 | 36 | 3.2 |
| #1 Glenoid | *D_2g_* | Measured values (Table Sc9) | 7.6 | 1.4 | 10.2 | 3.0 | 18.0 | 1.2 | 19.9 | 1.3 | 22.3 | 4.0 | 26 | 2.6 |
| #2 Acromion | *h_a_* | Based on image analysis ^b^ | 7.0 | 1.3 | 7.0 | 1.3 | 7.6 | 1.4 | 8.2 | 1.5 | 8.8 | 1.6 | 8.8 | 1.6 |
| #2 Acromion | *W_a_* | Measured values (Table Sc9) | 16.0 | 2.2 | 16.0 | 6.6 | 20.2 | 2.5 | 26.8 | 2.1 | 32.4 | 1.3 | 48 | 5.3 |
| #2 Acromion | *L_a_* | Measured data for 0 Y;  For other ages the percentage of *W_a_* ^с^ | 13.0 | 3.3 | 13.0 | 5.4 | 15.7 | 1.9 | 20.9 | 1.6 | 25.2 | 1.0 | 26 | 2.9 |
| #3 Scapula body | *S_bh_* | Derived from child and adult measurements | 2.7 | 0.35 | 2.7 | 0.35 | - | - | - | - | - | - | - | - |
| #4 Lateral marge | *W_lm_* | Measured values (Table Sc9) | - | - | - | - | 3.2 | 0.2 | 3.5 | 0.1 | 3.5 | 0.1 | 4.6 | 1 |
| #4 Lateral marge | *l_lm_* | Image analysis ^a^ | - | - | - | - | 10.0 | 1.2 | 10.0 | 1.2 | 10.0 | 1.2 | 10.0 | 1.2 |
| #4 Lateral marge | *h_lm_* | 85% of scapular length *S_L_* ^a^ | - | - | - | - | 73.5 | 8.6 | 93.5 | 5.4 | 105.6 | 7.4 | 119 |  |
| #1 | Ct.Th | Based on adult estimates:  0–1 years - two times thinner than for adults  5–15 years – the same as for adults | 0.45 | 0.13 | 0.45 | 0.13 | 0.9 | 0.25 | 0.9 | 0.25 | 0.9 | 0.25 | 0.9 | 0.25 |
| #2, #3, #4 | Ct.Th |  | 0.4 | 0.05 | 0.4 | 0.05 | 0.8 | 0.1 | 0.8 | 0.1 | 0.8 | 0.1 | 0.8 | 0.1 |

1. taken from the images provided with a scale (Cunningham et al. 2016; Schwarz 2007; Boneclone images)
2. for 0-Y and 1-Y, the value was estimated based on figures from manual of Cunningham et al. (2016); for other ages, it is assumed that the thickness of the acromion increases uniformly and became equal to that for adults by age 15. Value of CV was assumed to be the same as in adults.
3. percentage (45% of *W_a_*) was estimated based on measured data for 0 years (Corrigan 1960). For children ages 1 and 5 years, CV for *L_a_* is taken to be equal to CV for *W_a_.*

**Fig Sc2.** Scapula (a) lateral view, (b) posterior view, (c) anterior view, (d) stylized models (BPS) describing scapula segments: (1) glenoid; (2) acromion; (3) coracoid; (4) lateral edge. Letters and numbers are deciphered in the text.

The data used for estimation of scapula micro- and macro-parameter values are presented in Tables Sc11-Sc14

**Table Sc11**. Scapula acromion macro-parameters for adult male, published data (mean±STD, mm)

| Author | Age | N | *l_a_* | *w_a_* | *h_a_* |
| --- | --- | --- | --- | --- | --- |
| Badr El Dine et al. 2015 | Adult | 160 | 53±4 | 9±3 | 32±3 |
| Polguj et al, 2011 | Adult | 70 | 45±6 | - | - |
| Paraskevas et al, 2008 | Adult | 45 | 48±6 | 9±1 | 23±3 |
| Collipal et al, 2010 | Adult | 36 | - | 9±2 | 25±3 |
| **Average for BPS (CV%)** | | | **48 (11)** | **9 (18)** | **26 (11)** |

**Table Sc12**. Scapula lateral margin width for adult male, published data (mean±STD, mm)

| Author | Age | N | *w_l_* |
| --- | --- | --- | --- |
| Badr El Dine et al. 2015 | 23 (22-25) | 10 | 5±1 |
| **Average for BPS (CV%)** | | | **5 (13)** |

**Table Sc13**. Scapula glenoid macro-parameters for combined samples of male and female, published data (mean±STD, mm)

| Author | Age | N | *d_1g_* | *d_2g_* | *h_g_* |
| --- | --- | --- | --- | --- | --- |
| Frutos 2002 | Adult | 65 | 36±2 | 26±1 | - |
| Piyawinijwong et al. 2004 | Adult | 50 | 38±2 | 29±3 | - |
| Ozer et al. 2006 | Adult | 47 | 39±3 | 27±2 | - |
| Rajput et al. 2012 | Adult | 100 | 35±3 | 23±3 | - |
| Mamatha et al. 2011 | Adult | 202 | 34±3 | 23±2 | - |
| Gupta et al. 2016 | Adult | 60 | 38±3 | 24±2 | - |
| Chhabra et al. 2015 | Adult | 126 | 39±3 | 25±3 | - |
| Gosavi et al. 2014 | Adult | 229 | 35±4 | 24±3 | - |
| Patil et al. 2014 | Adult | 224 | 33±4 | 24±3 | - |
| Kavita et al. 2013 | Adult | 129 | 35±3 | 25±3 | - |
| Badr El Dine et al. 2015 | Adult | 160 | 39±3 | 28±2 | - |
| Polguj et al. 2011 | Adult | 70 | 38±3 | 28±3 | - |
| Uma et al. 2016 | Adult | 200 | 36±5 | 24±3 | - |
| Terrier et al. 2014 | 34 (18-70) | 19 | - | - | 20±2 |
| **Average for BPS (CV%)** | | | **36 (9)** | **26 (10)** | **20 (9)** |

The total height (*h_ltot_*=119 mm) and depth (*l_l_*=10 mm) of lateral margin were derived from anatomic-atlas images with the specified ratio of bone dimension to the natural bone size (Sinelnikov, 1978); and the images of transverse-sections of scapula (D'Alessandro and Bergman 2018; Armstrong and Murthi, 2016). The CV value for *w_l_* (13%, Table AS4) was taken for *h_ltot_* and *l_l_*.

Cortical thickness of glenoid wall was estimated by us using the published micro-CT-images containing a millimeter scale or images of objects of indicated dimension (Table AS6).

**Table Sc14**. Glenoid cortical thickness for adults (mean±STD, mm)

| Source of images | Age | Number of images (persons) | Ct.Th |
| --- | --- | --- | --- |
| Nyffeler et al. 2006 | Adults | 4 | 0.94±0.27 |
| Wee et al. 2015 | Adults | 8 | 0.82±0.24 |
| Glinnee et al. 2015 | Adults | 1 | 1.4±0.3 |
| **Average for BPS (CV%)** | | | **0.9 (28)** |

Cortical thickness of articular surface of glenoid was taken as =0.3 mm (CV=43%) as typical for bone articular surfaces (head of humerus and femur).

Cortical thickness of acromion and lateral margin of scapula was taken to be 10% lower than for the glenoid at the same CV: Ct.Th=0.8 (CV=29%).

**Analysis of published data on scapula microstructures**

Microstructure measurement data available for adults only. Table Sc15 presents measured values.

**Table Sc15**. Microparameters of the scapula glenoid for adults, published data (mean ± SD, mm)

| Author | n | Age | BV/TV% | SD | Tb.Th | SD | Tb.Sp | SD |
| --- | --- | --- | --- | --- | --- | --- | --- | --- |
| Li et al. 2015 | 8 | 75±13 | 15 | 3 | 0.14 | 0.014 | 0.831 | 0.199 |
| Knowles et al. 2019 | 14 | 67±8 | 25 | 8 | 0.26 | 0.05 | 0.800 | 0.130 |
| Jun et al. 2018 | 3 | 78±14 | 21 | 4 | 0.41 | 0.045 | 2.082 | 0,221 |
| Frich et al. 1998 | 6 | 56 (31-72) | 26 | 8 | - | - |  |  |
| **Average for BPS (CV%)** | | | **22** | **13-31*** | **0.24** | **13** | **0.96** | **14.5** |

*Min-max

We assume that the dynamics of changes in the parameters of the glenoid microstructure corresponds to that for the humerus which forms one joint with glenoid. Thus, it is assumed that the values of BV/TV and Tb.Th for children ages 5 years and older are equal to those for adults. In newborns, BV/TV is 1.3 times higher, and Tb.Th is 2 times lower than in adults; in children of age 1-year Tb.Th is 20% lower than in adults.

The accepted values of the parameters for Scapula are presented in Table Sc16.

**Table Sc16**. Parameters assumed for Scapula model (all segments).

| Age group | BV/TV  (min–max) | SD BV/TV | Tb.Th  (min–max) | SD Tb.Th | Tb.Sp  (min–max) | SD Tb.Sp |
| --- | --- | --- | --- | --- | --- | --- |
| 0 | 0.283  (0.123–0.443) | 0.08 | 0.120  (0.01–0.320) | 0.1 | 0.482  (0.01–0.968) | 0.243 |
| 1 | 0.218  (0.58–0.378) | 0.08 | 0.192  (0.01–0.392) | 0.1 | 0.964  (0.290–2.335) | 0.221 |
| 5 | 0.218  (0.58–0.378) | 0.08 | 0.240  (0.04–0.440) | 0.1 | 0.964  (0.290–2.335) | 0.221 |
| 10 | 0.218  (0.58–0.378) | 0.08 | 0.240  (0.04–0.440) | 0.1 | 0.964  (0.290–2.335) | 0.221 |
| 15 | 0.218  (0.58–0.378) | 0.08 | 0.240  (0.04–0.440) | 0.1 | 0.964  (0.290–2.335) | 0.221 |
| Adults | 0.218  (0.58–0.378) | 0.08 | 0.240  (0.04–0.440) | 0.1 | 0.964  (0.290–2.335) | 0.221 |

**References for scapula**

Armstrong AD and Murthi AM, Anatomic shoulder arthroplasty. Springer Publishing Switzerland. 2016

Bone clone images: [Internet]. Available from: https://boneclones.com/product/human-european-american-male-13-year-old-scapula-and-epiphysis-2pcs-FM-511.

Badr El Dine F, Hassan H. Ontogenetic study of the scapula among some Egyptians: Forensic implications in age and sex estimation using Multidetector Computed Tomography, Egyptian Journal of Forensic Sciences. 2015; 6 (2):56–77.

Cardoso HFV, Spake L, Humphrey LT. Age estimation of immature human skeletal remains from the dimensions of the girdle bones in the postnatal period. Am J Phys Anthropol. 2017 Aug;163(4):772–783. doi: 10.1002/ajpa.23248. Epub 2017 May 24. PubMed PMID: 28542741.

Chhabra N, Prakash S, Mishra BK. An anatomical study of glenoid cavity: its importance in shoulder prosthesis. Int J Anat Res 2015; 3(3):1419–24.

Collipal E, Silva H, Ortegal L, Espinoza E, Martinez C. The acromion and its different forms. Inter J of Morphol. 2010; 28(4): 1189–92.

Corrigan GE. The neonatal scapula. Biol Neonat. 1960 Oct; 2:159–67. PubMed PMID: 13695677.

Cunningham C, Scheuer L, Black S. Developmental Juvenile Osteology: Second Edition. 2016. 1-618 p.

D'Alessandro MP and Bergman RA (curators). Atlas of Human Anatomy in Cross Section: Section 2. Neck, Shoulders, Upper Arm, and Upper Thorax (Lungs) http://www.anatomyatlases.org/HumanAnatomy/2Section/03.shtml accessed 05 April 2018.

Fazekas IGy and Ko´sa F. Forensic Fetal Osteology. Budapest: Akade´miai Kiado´. 1978.

[Frich LH](https://www.ncbi.nlm.nih.gov/pubmed/?term=Frich%20LH%5BAuthor%5D&cauthor=true&cauthor_uid=9752644), [Odgaard A](https://www.ncbi.nlm.nih.gov/pubmed/?term=Odgaard%20A%5BAuthor%5D&cauthor=true&cauthor_uid=9752644), [Dalstra M](https://www.ncbi.nlm.nih.gov/pubmed/?term=Dalstra%20M%5BAuthor%5D&cauthor=true&cauthor_uid=9752644). Glenoid bone architecture [J Shoulder Elbow Surg.](https://www.ncbi.nlm.nih.gov/pubmed/9752644) 1998 Jul-Aug;7(4):356–61.

Frutos LR. Determination of sex from the clavicle and scapula in a Guatemalan contemporary rural indigenous population. Am J Forensic Med Pathol. 2002 Sep;23(3):284–8.

Glennie AR, Giles JW, Johnson JA, Athwal GS, Faber KJ. An in vitro study comparing limited to full cementation of polyethylene glenoid components. Journal of Orthopaedic Surgery and Research.2015; 101:142.

Gosavi S, Jadhav SD, Garud RS. Morphometric study of Scapular glenoid cavity in Indian population. IOSR Journal of Dental and Medical Sciences. 2013; 13: 2279–861.

Gupta C, Palimar V, Akshay BM, Kalthur SG. Morphometric study of suprascapular notch and scapular dimensions with their clinical implications. Research Journal of Pharmaceutical, Biological and Chemical Sciences, 2016; 7(5): 2105–2112.

Hrdlicka A. The scapula: visual observations. Am J Phys Anthropol. 1942; 29:73–94.

[Jun BJ](https://www.ncbi.nlm.nih.gov/pubmed/?term=Jun%20BJ%5BAuthor%5D&cauthor=true&cauthor_uid=28561262), [Vasanji A](https://www.ncbi.nlm.nih.gov/pubmed/?term=Vasanji%20A%5BAuthor%5D&cauthor=true&cauthor_uid=28561262), [Ricchetti ET](https://www.ncbi.nlm.nih.gov/pubmed/?term=Ricchetti%20ET%5BAuthor%5D&cauthor=true&cauthor_uid=28561262), [Rodriguez E](https://www.ncbi.nlm.nih.gov/pubmed/?term=Rodriguez%20E%5BAuthor%5D&cauthor=true&cauthor_uid=28561262), [Subhas N](https://www.ncbi.nlm.nih.gov/pubmed/?term=Subhas%20N%5BAuthor%5D&cauthor=true&cauthor_uid=28561262), [Li ZM](https://www.ncbi.nlm.nih.gov/pubmed/?term=Li%20ZM%5BAuthor%5D&cauthor=true&cauthor_uid=28561262), [Iannotti JP](https://www.ncbi.nlm.nih.gov/pubmed/?term=Iannotti%20JP%5BAuthor%5D&cauthor=true&cauthor_uid=28561262).Quantification of regional variations in glenoid trabecular bone architecture and mineralization using clinical computed tomography images. [J Orthop Res.](https://www.ncbi.nlm.nih.gov/pubmed/28561262) 2018 Jan;36(1):85–96. doi: 10.1002/jor.23620. Epub 2017 Jun 26.

Kavita P, Jaskaran S, Geeta. Morphology of coracoids process and glenoid cavity in adult human scapulae. IJAPBS 2013;2(2):19–22

[Knowles NK](https://www.ncbi.nlm.nih.gov/pubmed/?term=Knowles%20NK%5BAuthor%5D&cauthor=true&cauthor_uid=30366304), [G Langohr GD](https://www.ncbi.nlm.nih.gov/pubmed/?term=G%20Langohr%20GD%5BAuthor%5D&cauthor=true&cauthor_uid=30366304), [Faieghi M](https://www.ncbi.nlm.nih.gov/pubmed/?term=Faieghi%20M%5BAuthor%5D&cauthor=true&cauthor_uid=30366304), [Nelson A](https://www.ncbi.nlm.nih.gov/pubmed/?term=Nelson%20A%5BAuthor%5D&cauthor=true&cauthor_uid=30366304), [Ferreira LM](https://www.ncbi.nlm.nih.gov/pubmed/?term=Ferreira%20LM%5BAuthor%5D&cauthor=true&cauthor_uid=30366304). Development of a validated glenoid trabecular density-modulus relationship. [J Mech Behav Biomed Mater.](https://www.ncbi.nlm.nih.gov/pubmed/30366304) 2019 Feb;90:140–145. doi: 10.1016/j.jmbbm.2018.10.013. Epub 2018 Oct 12.

Li X, Williams P, Curry EJ, Choi D, Craig EV, Warren RF, Gulotta LV, Wright T. Trabecular Bone Microarchitecture and Characteristics in Different Regions of the Glenoid. Orthopedics. 2015; 38(3): 163–168.

Mamatha T, Pai SR, Murlimanju BV, Kalthur SG, Pai MM, Kumar B. Morphometry of glenoid cavity. Online J Health Allied Sciences. 2011; 10(3): 7.

Nyffeler RW, Sheikh R, Atkinson TS, Jacob HA, Favre P, Gerber C. Effects of glenoid component version on humeral head displacement and joint reaction forces: an experimental study. J Shoulder Elbow Surg. 2006; 15:625– 629.

Ozer I, Katayama K, Sagir M, Gulec E. Sex determination using the scapulae in medieval skeletons from east Anatolia. Coll Anthropoll. 2006; 30;415–419.

Paraskevas G, Tzaveas A, Papaziogas B, Kitsoulis P, Natsis K, Spanidou1 S. Morphological parameters of the acromion, Folia Morphol. 2008; 67:255–60.

Patil GV, Kolagi SI, Ramdurg U. Morphometrical Study of Scapular Glenoid Cavities. Global Journal of Medical Research. 2014; 14(2). https://medicalresearchjournal.org/index.php/GJMR/article/view/664; accessed 05 April 2018.

Piyawinijwong S, Sirisathira N, Chuncharunee A. The Scapula: Osseous Dimensions and Gender Dimorphism in Thais. Siriraj Hosp Gaz 2004; 56(7): 356–365.

Polguj M, Jędrzejewski KS, Podgórski M, Topol M. Correlation between morphometry of the suprascapular notch and anthropometric measurements of the scapula. Folia Morphol (Warsz). 2011; 70(2):109–15.

Rajput HB, Vyas KK, Shroff BD. A study of morphological patterns of glenoid cavity of the scapula. National J of Medical Research 2012;2(4): 504–507

Rissech C, Black S. Scapular development from neonatal period to skeletal maturity. A preliminary study. Int J Osteoarchaeol. 2007; 17:451–464.

Saunders S, Hoppa R, Southern R. Diaphyseal growth in a nineteenth-century skeletal sample of subadults from St Thomas’ Church, Belleville, Ontario. International Journal of Osteoarchaeology. 1993; 3: 265–281.

Schaefer M. A summary of epiphyseal union timings in Bosnian males. International Journal of Osteoarchaeology. DOI: 10.1002/oa.959. Copyright. John Wiley & Sons Limited. Reproduced with permission. 2008.

Schwarz JH. Skeleton Keys: An Introduction to Human Skeletal Morphology, Development and Analysis, 2nd Edition. Oxford University Press: Oxford. 2007; 402.

Sinelnikov RD. Atlas of human anatomy. Moscow “Medicina” 1978 (in Russian).

Terrier A, Ston J, Larrea X, Farron A. Measurements of three-dimensional glenoid erosion when planning the prosthetic replacement of osteoarthritic shoulders. Bone Joint J. 2014 Apr;96-B (4):513–8. doi: 10.1302/0301-620X.96B4.32641.

Uma SV, Balasubramanyam V. Morphometry of glenoid using digital photographs and image processing software. Int J Anat Res 2016; 4(3):2720–24.

Vallois HV. L’omoplate humaine. Bulletin de la Sociétié d’Anthropolgie de Paris. 1946; 7: 16–99.

Wee H, Armstrong AD, Flint WW, Kunselman AR, Lewis GS. Peri-implant stress correlates with bone and cement morphology: Micro-FE modeling of implanted cadaveric glenoids. J Orthop Res. 2015;33(11):1671–9.
