# Supplementary material for: Stochastic parametric skeletal dosimetry model for humans: Anatomical-morphological basis and parameter evaluation
Source: PLoS One. 2025 Jul 2;20(7):e0327156. doi: 10.1371/journal.pone.0327156 (PMC12306906; doi:10.1371/journal.pone.0327156)
Supplement: S13 Clavicle — (DOCX) [file pone.0327156.s013.docx]

**Clavicle**

**Pre-adults, analysis of published data on clavicle macro-parameters and cortical thickness**

The s-shape of the clavicle does not change significantly during maturation (McGraw et al. 2009; Cunningham et al. 2016). Fig. C1 illustrates this fact showing prenatal bone (Fig. C1 a) and mature bone (Fig. C1 b, c); the main measured parameters used in modeling are indicated.

The similarity of bone shape across ages allows us to simulate the clavicle with segments of the same shape (Fig. C1 e), i.e., one cylinder for body-shaft and two truncated cones for the acromial and sternal ends. The approaches to determining BPS parameters are also the same for all reference ages. The main parameters are indicated in Fig. C1 and are used in modeling:

**Fig. C1.** Right clavicle according to Cunningham et al. 2016 with modifications: (a) superior view on perinatal clavicle, acromial and sternal ends are indicated; (b) superior view on mature clavicle of similar shape; (c) anterior view on clavicle; (d) schematic images of cross sections in the positions indicated by arrows in the panels (b, c); (e) stylized models (BPSs). Letter designations are deciphered in the text below.

**e**

- Maximum (or total) clavicular length (*L_ml_)* is the greatest distance in a straight line between the sternal and acromial ends of the bone.
- Center-line length (*L_cl_*) corresponds to the length of the curve drawn between the acromial and sternal ends of the clavicle parallel to the surface (Bernat et al. 2014).
- Transverse (maximal) diameter at mid-shaft *D_m1_* (d_1b_).
- Vertical (minimal) diameter at mid-shaft *D_m2_* (d_2b_).
- Transverse diameter of sternal end (*d_1s_*).
- Vertical (minimal) diameter of sternal end (*d_2s_*).
- Transverse (maximal) diameter of acromial end (*d_1a_*).
- Vertical (minimal) diameter of acromial end (*d_2a_*).

BPS 1 (body) was described by cylinder with elliptic base of height *h_b_* (74% of *L_ml_*) and diameters *D_m1_* and *D_m2_*; cortical layer is located on the walls of the cylinder;

BPS 2 (sternal end) was described by the truncated cone of height *h_e_* (13% of *L_ml_*); smaller elliptical base of diameters *D_m1_* and *D_m2;_* larger elliptical base of diameter *d_s1_* and *d_s2_;* and cortical layer located on the walls of the cone;

BPS 3 (acromial end) was described by the truncated cone of height *h_e_* (13% of *L_ml_*); smaller elliptical base of diameters *D_m1_* and *D_m2;_* larger elliptical base of diameter *d_a1=_D_m1_* and *d_a2_;* and cortical layer located on the walls of the cone.

Tables C1–C2 present the measured data on clavicle macro-parameters and cortical thickness of pre-adults. Note that for ages up to 15-Y macro-parameters are age-dependent.

**Table C1.** Published data on the pre-adult clavicle maximal length (*L_ml_*), mm.

| Author | Age (range) | N | Mean | SD |
| --- | --- | --- | --- | --- |
| Fazekas et al. 1978 | 0* | 10 | 44.1 | 1.5 |
| Sherer et al. 2006 | 0* | 1 | 46.9 |  |
| McGraw et al. 2009 | 0 | 53 | 37.7 | 7.0 |
| Corron 2016 | 0 | 21 | 53.2 | 8.2 |
| Black et al. 1996 | 0.2 (0–0.5) | 11 | 44.4 | 5.5 |
| Bleuze et al. 2016 | 0.75 | 13 | 60.2 | 3.2 |
| Corron 2016 | 1 | 22 | 69.8 | 5.8 |
| McGraw et al. 2009 | 1 | 60 | 46.5 | 6.9 |
| Bleuze et al. 2016 | 2.0 (1.5–2.5) | 12 | 64.9 | 2.6 |
| Bleuze et al. 2016 | 4.0 (3.5–4.5) | 5 | 78.3 | 6.7 |
| Corron L 2016 | 5 | 14 | 93.5 | 5.2 |
| McGraw et al. 2009 | 5 | 56 | 85.8 | 13.5 |
| Bleuze et al. 2016 | 6 | 1 | 91.5 | - |
| McGraw et al. 2009 | 10 | 55 | 121.4 | 9.3 |
| Corron 2016 | 10 | 14 | 116.1 | 7.4 |
| McGraw et al. 2009 | 15 f | 29 | 143.4 | 7.0 |
| McGraw et al. 2009 | 15 m | 28 | 150.6 | 13.5 |
| Corron 2016 | 15 m+f | 18 | 143.8 | 8.6 |

* late fetus of 40 weeks; m- male; f- female.

**Table C2.** Published data on mid-diaphysis diameters (*D_m1,_ D_m2_*) of the pre-adult clavicle according to Corron 2016, mm.

| Age | N | *D_m1,_* | SD | *D_m2_* | SD |
| --- | --- | --- | --- | --- | --- |
| 0 | 21 | 5.9 | 1.5 | 4.3 | 1 |
| 1 | 22 | 7.15 | 0.67 | 5.2 | 0.5 |
| 5 | 14 | 8.7 | 0.8 | 6.8 | 0.7 |
| 10 | 14 | 10.9 | 1.3 | 8.3 | 0.8 |
| 15* | 18 | 13.2 | 1.5 | 10.5 | 1.3 |

*-for male and female

To evaluate the whole set of BPS parameters it is necessary to obtain the center-line length of the clavicle (*L_cl_*) which were not found in the literature. According to Bernat et al. (2014), adult *L*_cl_ is about 6% more than *L_ml_*. Thus, we calculated the values *L*_cl_ for reference ages (Table C3).

**Table C3.** Calculated values of center-line length (*L_cl_*) for pre-adults, mm.

| Age | Maximal length *L_ml_* (based on Table C1) | | Calculated center-line length *L_cl_* | |
| --- | --- | --- | --- | --- |
|  | M | SD | M | SD |
| 0 | 42.6 | 6.5 | 45.2 | 6.9 |
| 1 | 53.8 | 6.1 | 57.0 | 6.5 |
| 5 | 87.3 | 11.8 | 92.5 | 12.5 |
| 10 | 120.3 | 8.9 | 127.5 | 9.4 |
| 15 f | 143.4 | 7.0 | 152.0 | 7.4 |
| 15 m | 150.6 | 13.5 | 159.6 | 14.3 |

Taking into account that the shape of the clavicle does not change significantly with age, unmeasured parameters such as *d_s1_, d_s2_, d_a1_, d_a2_* were calculated using the ratios obtained from adult data (parameters for adults were evaluated on the basis of measurements). For adults, the ratios *d_s1_*/*D_m_* = 2.0, *d_s2_*/*D_m_* = 2.2, *d_a1_*/*D_m_* = 1.8, and *d_a1_*=*D_m1_* (note: *D_m_* is the mean value between *D_m1_* and *D_m2_*). These relationships were used to estimate parameters for reference ages (Table C4).

Direct measurements of the cortical thickness of the clavicle in children were not found. However, Fujita et al. 1968 (n = 365; age 10–80 years) note that the cortical thickness in adolescents aged 10–19 years is 20% more than in adults. This suggests a relatively high thickness of the cortical layer in children. Taking into account this information, the following cortical thickness values were taken for the clavicular BPSs (Table C4).

**Table C4.** Clavicle parameters assumed for pre-adult BPS 1 (clavicular body), mm.

| Age | *h_b_* | *SD* | *D_m1,_* | SD | *D_m2_* | SD | *Ct.Th* | SD |
| --- | --- | --- | --- | --- | --- | --- | --- | --- |
| 0 | 33.4 | 5.1 | 5.9 | 1.5 | 4.3 | 1 | 0.8 | 0.20 |
| 1 | 42.2 | 4.8 | 7.2 | 0.7 | 5.2 | 0.5 | 0.9 | 0.09 |
| 5 | 68.5 | 9.3 | 8.7 | 0.8 | 6.8 | 0.7 | 1.1 | 0.10 |
| 10 | 94.4 | 7.0 | 10.9 | 1.3 | 8.3 | 0.8 | 1.8 | 0.47 |
| 15 m | 118.1 | 10.6 | 12.0 | 2.0 | 12.0 | 1.0 | 1.8 | 0.47 |
| 15 f | 112.5 | 5.5 | 10.0 | 0.9 | 10.0 | 0.1 | 1.8 | 0.47 |

**Table C5.** Clavicle parameters assumed for pre-adult BPS 2 (sternal end), mm.

| Age | *h_e_* | *SD* | *d_s1_* | *SD* | *d_s2_* | *SD* | *Ct.Th* | SD |
| --- | --- | --- | --- | --- | --- | --- | --- | --- |
| 0 | 5.9 | 0.9 | 11.6 | 2.8 | 10.4 | 2.5 | 0.33 | 0.08 |
| 1 | 7.4 | 0.8 | 14.1 | 1.4 | 12.7 | 1.2 | 0.40 | 0.04 |
| 5 | 12.0 | 1.6 | 17.6 | 1.7 | 15.9 | 1.5 | 0.49 | 0.05 |
| 10 | 16.6 | 1.2 | 21.8 | 2.4 | 19.6 | 2.1 | 0.77 | 0.20 |
| 15 m | 19.8 | 1.0 | 26.0 | 4.0 | 24.0 | 3.0 | 0.77 | 0.20 |
| 15 f | 20.7 | 19 | 24.0 | 3.8 | 21.0 | 2.9 | 0.77 | 0.20 |

**Table C6.** Clavicle parameters assumed for pre-adult BPS 3 (acromial end), mm.

| Age | *SD* | *d_a1_* | SD | *d_a2_* | SD | *h_e_* | *Ct.Th* | SD |
| --- | --- | --- | --- | --- | --- | --- | --- | --- |
| 0 | 5.9 | 0.9 | 10.0 | 2.4 | 5.9 | 2.9 | 0.33 | 0.08 |
| 1 | 7.4 | 0.8 | 12.1 | 1.2 | 7.2 | 1.4 | 0.40 | 0.04 |
| 5 | 12.0 | 1.6 | 15.1 | 1.5 | 8.7 | 1.6 | 0.49 | 0.05 |
| 10 | 16.6 | 1.2 | 18.8 | 2.0 | 10.9 | 2.5 | 0.77 | 0.20 |
| 15 m | 19.8 | 1.0 | 22.0 | 2.0 | 12.0 | 3.5 | 0.77 | 0.20 |
| 15 f | 20.7 | 19 | 21.0 | 1.2 | 10.0 | 3.2 | 0.77 | 0.20 |

m- male; f- female

**Adults, analysis of published data on clavicle macro-parameters and cortical thickness for**

Measurements of clavicle, which were used in the study:

*d1_bs_-* transverse diameter of sternal end

*d2_bs_-* vertical diameter of sternal end

*d3_b_-* transverse diameter at mid-shaft (*D_m1_*)

*d4_b_-* vertical diameter at mid-shaft (*D_m2_*)

*d1_ba_-* vertical diameter of acromial end

*d2_ba_-* transverse diameter of acromial end

Clavicle consists of cylindrical S-shape *body* and *two ends: sternal (medial) and acromial (lateral)*. The clavicle sizes are sex-dependent. The clavicle (Fig C1) was divided into the following segments: two equal end-segments, and two unequal body-segments:

*1. End segment (paired)* was described by elliptic cylinder of axis (diameter) *d1e= d1bs*, and *d2e=* *d2bs.*  *l_e_* was taken as 20 mm. Cortical layer covers the lateral side of the cylinder*.*

*2. Body* *segment* 1 (*sternal)* was stylized by truncated elliptic cone of axis (diameters) *d1bs, d2bs,* *d3b, d4b* (*d3b, d4b* correspond to the measured mid-diaphysis diameters *D_m1,_ D_m2_*); height of cone is the length of body segment *lb*. Cortical layer covers the lateral surface of cone.

*3. Body* *segment* 2 (*acromial)* was stylized by similar truncated elliptic cone of axis (diameters) *d1ba,* *d2ba (acromial base),* and two axes *d3b, d4b* common to both segments (in the middle of clavicular body *D_m1,_ D_m2_*); height of cone is the same as that of the *body segment* 1 (*lb*). Cortical layer covers the lateral surface of cone.

Measured data on clavicle sizes are presented in Table C1-C4 for male and female.

**Table C7.** Macro-parameters for clavicle for adult **male** (mean±SD, mm), published data.

| Author | N | Age (range) | *d1_bs_* | *d1_ba_* | *d3_b_ (D_m1)_* | *d2_bs_* | *d2_ba_* | *d4_b_*  *(D_m2)_* |
| --- | --- | --- | --- | --- | --- | --- | --- | --- |
| King et al. 2012 | 101 | 49 (16-84) | - | 11±1 | 13±2 | - | - | - |
| Singh et al. 2016 | 195 | 55 (17-94) | - | - | - | 24±3 | 21±3 | 12±1 |
| Bernat et al. 2013 | 17 | 77 (43-99) | 26±4 | 15±2 | 10±1 | 25±3 | 26±4 | 12±1 |
| **Assumed for BPS (CV%)** | | | **26 (16)** | **12 (17)** | **12 (17)** | **24 (13)** | **22 (14)** | **12 (8)** |

**Fig C1.** Clavicle (a) sagittal section, cortical layers and trabecular structures are shown; (b) superior view; (c) stylized models (BPS) describing clavicle segments: (1) clavicle end, (2) body sternal segment, and (3) body acromial segment. Length of the body (*lb*) was derived from the measured data on total curve-length (syn. center-length) of the clavicle *L_cl_*=*2lb+2le*: *l_b_=(L_total_-2l_e_)/2;* center-line length (*L_cl_*) corresponds to the length of the curve drawn between the acromial and sternal ends of the clavicle parallel to the surface. Other letter symbols are described in the text.

**Table C8.** Macro-parameters for clavicle for adult **female** (mean±SD, mm), published data.

| Author | N | Age (range) | *d1_bs_* | *d2_bs_* | *d3_b_*  *(D_m1)_* | *d4_b_*  *(D_m2)_* | *d1_ba_* | *d2_ba_* |
| --- | --- | --- | --- | --- | --- | --- | --- | --- |
| King et al. 2012 | 108 | 48 (17-82) | - | - | 11±1 | - | 9±1 | - |
| Singh and Pathak 2016 | 68 | 17-94 | - | 21±3 | - | 10±1 | - | 19±3 |
| Bernat et al. 2013 | 17 | 77 | 25±4 | 23±3 | 9±1 | 10±1 | 13±1 | 24±3 |
| Daruwalla et al. 2010 | 18 | 54 | - | 16 | - | 10 | - | 20 |
| Parsons 1916 | 52 | adult | 22 | 23 | 10 | 10 | 11 | 23 |
| Andermahr 2006 | 110 | 74 (35-95) | 24±4 | - | 11±1 | - | - | 21±4 |
| **Assumed for BPS (CV%)** | | | **24(16)** | **21(14)** | **10(9)** | **10(10)** | **12(10)** | **21(15)** |

**Table C9.** Total (central) length of clavicle for adult **male** (mean±SD, mm), published data.

| Author | N | Age (range) | *L_cl_* |
| --- | --- | --- | --- |
| King et al. 2012 | 101 | 49 (16-84) | 157±10 |
| Singh et al. 2016 | 195 | 55 (17-94) | 159±9 |
| Bernat et al. 2013 | 17 | 77 (43-99) | 167±7 |
| Boehm et al. 2003 | 18 | 84 (73-97) | 159±7 |
| Parson 1916 | 1 | adult | 161 |
| Andermahr 2006 | 2 | adult | 165±11 |
| Huang 2007 | 2 | adult | 146±11 |
| Jit 1966 | 116 | adult | 156 |
| Kaur et al. 1997 | 748 | adult | 159 |
| Patel et al. 2009 | 107 | adult | 151 |
| McCormick et al. 1991 | 560 | adult | 167 |
| Akhlaghi et al.2012 | 60 | adult | 156 |
| Papaioannou et al. 2012 | 81 | adult | 163 |
| Kralik et al. 2014 | 98 | adult | 163 |
| Alcina et al. 2015 | 45 | adult | 165 |
| **Assumed for BPS (CV%)** | | | **159 (5)** |

**Table C10.** Total (central) length of clavicle for adult **female** (mean±SD, mm), published data.

| Author | N | Age (range) | *L_cl_* |
| --- | --- | --- | --- |
| King et al. 2012 | 108 | 48 (17-82) | 146±8 |
| Bernat et al. 2013 | 17 | 77 | 143±8 |
| Parsons 1916 | 52 | Adult | 138 |
| Andermahr 2006 | 110 | 74 (35-95) | 146 |
| Huang 2007 | 50 | 21-35 | 153±10 |
| Singh and Pathak 2016 | 68 | 17-94 | 137±8 |
| Daruwalla et al. 2010 | 18 | 54 | 149 |
| **Assumed for BPS (CV%)** | | | **143 (6)** |

Table C5 present the published data on cortical thickness for adults

**Table C11.** Cortical thickness of clavicle for combined samples of males and females (mean± SD, mm), published data.

| Author | N | Age | Ct.Th |
| --- | --- | --- | --- |
| Body segments | | | |
| Shah and Routal, 2015 | 30 | 78 (60-89) | 1.6±0.4 |
| Wu and Murrell, 2008 | 20 | Adult | 1.8±1.2 |
| Andermahr* 2006 | 70 | Adult | 1.35±0.3 |
| **Assumed for BPS (CV%)** | | | **1.5 (26)** |
| Clavicle end | | | |
| Milenkovic 2013 | 42 | 70 (20-90) | 0.64±0.12 |
| **Assumed for BPS (CV%)** | | | **0.64 (19)** |

**Analysis of published data on clavicle microstructures**

**Table C12.** Clavicle micro-parameters for adult male, published data (mm)

| Author | N | Age | Tb.Th. | Tb.Sp. | BV/TV |
| --- | --- | --- | --- | --- | --- |
| Hough 2011 | 1 | 40 | 0.27 | 1.1 | 12 |
| Gao et al. 2017 | 1 | adult | 0.15 | - | 17 |
| Pafundi 2009 | 1 | 18 | 0.17 | - | 17 |
| **Assumed for body segments BSPs (CV%)** | | | **0.19 (31)** | **1.1 (13)*** | **15 (8-18)** |
| Milenkovic 2013 | 42 | 70 (20-90) | 0.15±0.02 | - | 29 (15-46) |
| **Assumed for end BSPs (CV%)** | | | **0.15 (13)** | **1.1 (13)*** | **29 (15-46)** |

*CV% was taken equal to rib BPS

For children ages 0 to 1 year, the microparameters were taken to be the same for all clavicular BPSs, both for body (BPS1) and ends (BPS2, 3). These parameters correspond to those for adult clavicular ends. For children ages 5, 10 and 15 years, all parameters are taken to be the same as for adults.

**Reference for clavicle**

Akhlaghi M, Moradi B, Hajibeygi M, Sex determination using anthropometricdimensions of the clavicle in Iranian population. J. Forensic Leg. Med. 2012; 19(7):381-385.

Alcina M, Rissech C, Clavero A, Turbon D. Sexual dimorphism of the clavicle in a modern Spanish sample, Eur. J. Anat. 2015; 19(1): 73-83.

Andermahr J, Jubel A, Elsner A, Johann J, Prokop A, Rehm KE, Koebke J, Anatomy of the clavicle and intermedullar nailing of midclavicular features, Clin. Anat. 2007; 20: 48–56.

Bernat A, Huysmans T, Van Glabbeek F, Sijbers J, Gielen J, Van Tongel A. The anatomy of the clavicle: a three-dimensional cadaveric study. Clin Anat. 2014; 27(5):712–723.

Black SM. and Scheuer JL. Age changes in the clavicle: from the early neonatal period to skeletal maturity. International Journal of Osteoarchaeology. 1996; 6: 425–434.

Bleuze MM, Wheeler SM, Williams LJ, Dupras TL. Growth of the pectoral girdle in a sample of juveniles from the kellis 2 cemetery, Dakhleh Oasis, Egypt. Am J Hum Biol. 2016 Sep 10; 28(5):636–45.

Boehm TD, Kirschner S, Fischer A, Gohlke F. The relation of the coracoclavicular ligament insertion to the acromioclavicular joint: a cadaver study of relevance to lateral clavicle resection. Acta Orthop Scand. 2003; 74(6):718–21.

Corron L. Juvenile age estimation in physical anthropology: A critical review of existing methods and the application of two standardised methodological approaches. Biological anthropology. Aix- Marseille Universite. English. 2016.

Cunningham C, Scheuer L, Black S. Developmental Juvenile Osteology. 2 ed. London: Academic Press. 622. 2016.

Daruwalla ZJ, Courtis P, Fitzpatrick C, Fitzpatrick D, Mullett H. Anatomic variation of the clavicle: A novel three-dimensional study. Clin Anat. 2010 Mar;23(2):199-209.

Fazekas IGy. and Kósa F. Forensic Fetal Osteology. Budapest: Akadémiai Kiadó. 1978.

Fujita T, Orimo H, Ohata M, Yoshikawa M. Changes in the cortical thickness of the clavicle according to age. J Am Geriatr Soc. 1968 Apr;16(4):458–62.

Gao S, Ren L, Qui R, Wu Z, Li C, Li J. Electron absorbed fractions in an image-based microscopic skeletal dosimetry model of Chinese adult male. Radiat Prot Dosimetry. 2017; 175(4):450-459.

Hough M, Johnson P, Rajon D, Jokisch D, Lee C, Bolch W. An image-based skeletal dosimetry model for the ICRP reference adult male—internal electron sources. Phys Med Biol. 2011; 56(8): 2309–2346.

Huang JI, Toogood P, Chen MR, Wilber JH, Cooperman DR. Clavicular anatomy and the applicability of precontoured plates. J Bone Joint Surg Am. 2007; 89(10):2260-2265.

Jit I, Singh S. The sexing of the adult clavicles. Ind J Med Res. 1966; 54:551–571.

Kaur K, Sidhu SS, Kaushal S, Kaur B. Sexing the northwest Indian adult clavicles of Patiala zone. J Anat Soc Ind. 1997; 46(2):121–130.

King PR, Scheepers S, Ikram A. Anatomy of the clavicle and its medullary canal: a computed tomography study. Eur J Orthop Surg Traumatol. 2014; 24(1):37–42.

Kralik M, Urbanova P, Wagenknechtova M. Sex assessment using clavicular measurements: inter- and intra-population comparisons. Forensic Sci Int. 2014; 234:181. 1–15.

McCormick WF, Stewart JH, Green H. Sexing of human clavicles usinglength and circumference measurements, Am. J. Forensic Med. Pathol. 1991; 12:175–181.

McGraw MA, Mehlman CT, Lindsell CJ. and Kirby CL. Postnatal growth of the clavicle: birth to eighteen years of age. Journal of Pediatric Orthopedics. 2009; 29: 937.

Milenković P. Age Estimation Based on Analyses of Sternal End of Clavicle and the First Costal Cartilage Doctoral Dissertation. University OF Belgrade School of Medicine. Belgrade. 2013.

Pafundi D. Image-based skeletal tissues and electron dosimetry models for the ICRP reference pediatric age series. A dissertation presented to the graduate schools of the University of Florida in partial fulfillment of the requirements for the degree of doctor of the philosophy University of Florida. 2009.

Papaionnou VA, Krantioti EF, Joveneaux P, Nathena D, Michalodimitrakis M. Sexual dimorphism of scapula and the clavicle in a contemporary Greek population: applications in forensic identification. Forensic Sci Int. 2012; 217(1-3): 231-237. doi: 10.1016/j.forsciint.2011.11.010. Erratum in: Forensic Sci Int. 2017 Jan; 270:183.

Parsons FG. On the proportions and characteristics of the modern English clavicle. J. Anat. 1916; 51:71–93.

Patel JP, Shah RK, Merchant SP, Nirvan AB, Shah GV. Sexing of the adult human clavicle in Gujarat zone. Gujarat Med. J. 2009; 64:40–46.

Shah VM, Routatal RV. Structure of Clavicle in Relation to Weight Transmission. J Clin Diagn Res. 2015; 9(7):1–4.

Sherer D, Sokolovski M, Dalloul M, Khoury‐Collado F, Osho J, Lamarque M and Abulafia O. Fetal clavicle length throughout gestation: a nomogram. Ultrasound in Obstetrics and Gynecology. 2006; 27: 306–310.

Singh JS, Pathak RK. Variability in anatomical features of human clavicle: Its forensic anthropological and clinical significance. Anthropology Department, Institute of Forensic Science and Criminology, Panjab University, Chandigarh 160014, India. Translational Research in Anatomy. June–September 2016; 3–4: 5–14.

Wu XL. Murrell GAC. The Distal Clavicle Morphology. Techniques in Shoulder & Elbow Surgery. 2008; 9(2):80–84.
