# Supplementary material for: Stochastic parametric skeletal dosimetry model for humans: Anatomical-morphological basis and parameter evaluation
Source: PLoS One. 2025 Jul 2;20(7):e0327156. doi: 10.1371/journal.pone.0327156 (PMC12306906; doi:10.1371/journal.pone.0327156)
Supplement: S14 Hand Foot — (DOCX) [file pone.0327156.s014.docx]

**Hand and foot bones of newborns**

**Analysis of published data on wrist and ankle, hand and foot bone macro-parameters and cortical thickness**

Hand and foot bones include carpal and metacarpal bones, tarsal and metatarsal bones, and phalanges of the fingers and toes. They are very small in newborns, but contain active marrow up to the age of 1 year. Fig. HF1 shows the set of hand and foot bones of a newborn. Together they comprise a significant set of bones, so it was decided to create one generalized model to represent these tubular bones. BPS1 describes bones of hands (averaged parameters for metacarpal, proximal and intermediate phalanxes) and foot (averaged parameters for five metatarsal, five proximal phalanxes and first intermediate phalanx). BPS2 describes the tarsal centers of ossification in the foot (talus and calcaneus).

**Fig. HF1.** Perinatal hand (a, b) and foot bones (c, d); panels (a) and (c) present the set of dry bones (Scheuer and Black 2004); panels (b) and (d) show the radiograph (x-ray) images from (normal pediatric bone x-ray); (c, d) - stylized models (BPSs): (c)- BPS1 describes the tubular bones of the hand and foot; (d)- BPS2 describes the calcaneus and talus bones; *h_i_*- diaphyseal length of *i*-bone; *d_i_* – mid-diameter of *i*-bone; *d_1_* and *d_2_* – diameters of tarsal/calcaneus ossification centers.

Tables HF1 and HF2 presents the results of bones measured for late fetus (40 weeks) that are assumed to be appropriate for newborns.

**Table HF1.** Diaphyseal length (*h_i_)* of first left metatarsal and metacarpal bones according to Fazekas et al. 1978 (late fetus of 40 weeks, n=10), mm.

| Site | M | SD |
| --- | --- | --- |
| First metatarsal | 13.2 | 1.0 |
| First metacarpal | 9.3 | 1.0 |

**Table HF2**. Measured parameters of metacarpal bones and phalanges of hand according to Raziye, et al. 2016 (late fetus of 40 weeks; n=14), mm.

| Bone | *h_i_* | | Proximal diameter | | Distal diameter | |
| --- | --- | --- | --- | --- | --- | --- |
|  | M | SD | M | SD | M | SD |
| Metacarpal bones | | | | | | |
| MC 1 | 10.1 | 0.2 | 4.4 | 0.1 | 4.5 | 0.3 |
| MC 2 | 14.6 | 0.8 | 4.5 | 0.1 | 4.6 | 0.4 |
| MC 3 | 13.7 | 1.0 | 4.1 | 0.1 | 4.6 | 0.4 |
| MC 4 | 11.9 | 0.3 | 4.1 | 0.1 | 4.4 | 0.3 |
| MC 5 | 11.2 | 0.4 | 3.8 | 0.1 | 4.0 | 0.1 |
| Proximal phalanxes | | | | | | |
| PP 1 | 7.4 | 0.2 | 4.2 | 0.1 | 3.2 | 0.1 |
| PP 2 | 7.3 | 0.2 | 5.4 | 0.2 | 3.8 | 0.1 |
| PP 3 | 8.0 | 0.2 | 5.1 | 0.1 | 4.1 | 0.1 |
| PP 4 | 6.4 | 0.2 | 4.6 | 0.1 | 3.9 | 0.1 |
| PP 5 | 5.3 | 0.1 | 4.2 | 0.2 | 3.0 | 0.1 |
| Intermediate (middle) phalanxes | | | | | | |
| IP 2 | 5.8 | 0.3 | 4.6 | 0.2 | 3.4 | 0.2 |
| IP 3 | 6.4 | 0.2 | 4.4 | 0.1 | 3.5 | 0.1 |
| IP 4 | 5.8 | 0.2 | 4.0 | 0.2 | 3.1 | 0.1 |
| IP 5 | 4.6 | 0.2 | 3.2 | 0.1 | 3.1 | 0.1 |
| Averaged | 8.5±3.3 | | 4.1 ±0.6 | | | |

Note: values of variability of bone sizes were taken as variability between bones of different position

We have estimated the relative sizes of the foot bones (Table HF 3), and then the actual sizes (Table HF 4) based on images from the manual of Cunningham et al. (2016). The relative diameter of tubular foot bones *d_i_* (relative diaphyseal length) was estimated on the basis of image analysis from the manual of Cunningham et al. (2016); normal pediatric bone x-ray; Scheuer and Black (2004).

**Table HF3.** Diaphyseal length of foot bones relative to first metatarsal bone (row from first to fifth), rel. units.

| Level | #1 | #2 | #3 | #4 | #5 |
| --- | --- | --- | --- | --- | --- |
| Metatarsal | **1*** | 1.09 | 1.11 | 1.07 | 1.0 |
| Proximal phalanx | 0.44 | 0.49 | 0.49 | 0.33 | 0.38 |
| Intermediate phalange | 0.44 | - | - | - | - |

*Value 13.2 mm from Table HF1 was taken as unit

**Table HF4.** Calculated diaphyseal length of foot-bones (row from first to fifth, based on Table HF4 and HF5), mm.

| Level | #1 | #2 | #3 | #4 | #5 |
| --- | --- | --- | --- | --- | --- |
| Metatarsal | 13.20 | 14.37 | 14.67 | 14.08 | 13.20 |
| Proximal phalanx | 5.87 | 6.45 | 6.45 | 4.40 | 4.99 |
| Intermediate phalanx | 5.87 | - | - | - | - |
| Average *h_i_* | 9.4±4.4 | | | | |

**Assumed BPS1 parameters (CV%): *h*=8.9 (43); *d*=3.8 (42)**

Images from Cunningham et al. 2016 were also used for evaluation of sizes of tarsal bones (Table HF5**)**

**Table HF5.** Talus and calcaneus bone sizes (based on Cunningham et al. 2016), mm.

| Bone | *d1* | *d2* |
| --- | --- | --- |
| Calcaneus | 8.5 | 13.2 |
| Talus | 7.04 | 11.1 |
| **Averaged for BPS2** | **7.8±1.1** | **12.2±1.4** |

The cortical thickness of the tubular bones was taken to be the doubled thickness of the trabeculae for calcaneus (see below) **Ct.Th= 0.120×2= 0.24 mm (CV= 25%)**.

**Analysis of published data on microstructures of foot and hand bones**

We do not have data on direct measurements of the trabecular structures of wrist and hand bones in children up to 1 year. Comparative analysis of the trabecular structures of various adult bones (Chircher et al. 2015, Table HF6) showed that wrist and hand bones, and ankle and foot bones have close BV/TV ratios.

**Table HF6.** Trabecular fraction in different bone of adults (according to Chircher et al. 2015).

| Bone | N | BV/TV | SD BV/TV |
| --- | --- | --- | --- |
| Distal radius | 38 | 0.21 | 0.03 |
| Proximal metacarpal | 30 | 0.23 | 0.03 |
| Distal metatarsal | 35 | 0.21 | 0.03 |

Trabecular microstructures are most studied in calcaneus. We have used the data of Saers (2017), who investigated the age-dynamics of trabecular structures of calcaneus, for derivation of trabecular bone parameters for ankle and foot bones.

Thus, for children under one year old, it is accepted that wrist and hand bones have the same trabecular microstructure as calcaneus (Table HF7).

**Table HF7**. Parameters of microstructures assumed for ankle, foot, and hand bones (based on Saers 2017) for ages of 0–1 year.

| BV/TV  (min–max)* | SD BV/TV | Tb.Th  (min–max)* | SD Tb.Th | Tb.Sp  (min–max)* | SD Tb.Sp |
| --- | --- | --- | --- | --- | --- |
| 0.221  (0.01**–0.501) | 0.14 | 0.120  (0.056–0.184) | 0.032 | 0.248  (0.01**–0.506) | 0.129 |

*-M±2SD; **- Accepted instead of estimated negative value

**References for hand and foot bones**

[Chirchir H](https://www.ncbi.nlm.nih.gov/pubmed/?term=Chirchir%20H%5BAuthor%5D&cauthor=true&cauthor_uid=28101969), [Ruff CB](https://www.ncbi.nlm.nih.gov/pubmed/?term=Ruff%20CB%5BAuthor%5D&cauthor=true&cauthor_uid=28101969), [Junno JA](https://www.ncbi.nlm.nih.gov/pubmed/?term=Junno%20JA%5BAuthor%5D&cauthor=true&cauthor_uid=28101969), [Potts R](https://www.ncbi.nlm.nih.gov/pubmed/?term=Potts%20R%5BAuthor%5D&cauthor=true&cauthor_uid=28101969). Low trabecular bone density in recent sedentary modern humans. [Am J Phys Anthropol.](https://www.ncbi.nlm.nih.gov/pubmed/28101969) 2017 Mar;162(3):550–560. doi: 10.1002/ajpa.23138. Epub 2017 Jan 19.

Cunningham C, Scheuer L, Black S. Developmental Juvenile Osteology. Second Edition. Elsevier Academic Press. 2016.

Fazekas IGy, Kósa F. Forensic Fetal Osteology. Budapest: Akadémiai Kiadó. 1978.

Normal pediatric bone X-ray, available in: <https://bonexray.com/>; <http://bones.getthediagnosis.org/>; <http://bonepit.com/>

Raziye D, Ceren U, Kadir D, Osman S, Mehmed Ali M. A Radiological Investigation on the Hand Development in Human Fetuses Throughout the Fetal Period and an Evaluation Performed in Terms of its Clinical Importance Hand Development. International Journal of Morphology. International Journal of Morphology; 2016;34: 1539–1552. doi:10.4067/s0717-95022016000400057

Saers J. Ontogeny and functional adaptation of trabecular bone in the human foot. Doctoral Thesis. University of Cambridge. 2017.

Scheuer L, Black S. The juvenile Skeleton. Elsevier Academic Press London WC1X 8RR, UK. 2004.
