# Supplementary material for: Stochastic parametric skeletal dosimetry model for humans: Anatomical-morphological basis and parameter evaluation
Source: PLoS One. 2025 Jul 2;20(7):e0327156. doi: 10.1371/journal.pone.0327156 (PMC12306906; doi:10.1371/journal.pone.0327156)
Supplement: Attachment — Submitted filename: Responses to reviewers_20250327.docx [file pone.0327156.s016.docx]

Response to Reviewers

The following is our response to the Reviewers’ comments on the paper Tolstykh et al. **"Stochastic parametric skeletal dosimetry model for humans: Anatomical-morphological basis and parameter evaluation "**, which was submitted to PLOS ONE

*Journal requirements:*

*When submitting your revision, we need you to address these additional requirements.*

*1. Please ensure that your manuscript meets PLOS ONE's style requirements, including those for file naming.*

Confirmed, this has been verified.

*2. Thank you for stating the following financial disclosure:*

*[This work was funded by Federal Medical-Biological Agency of Russia (MOD, EIT, EAS, PAS, VIZ) and the U.S. Department of Energy’s Office of International Health Programs (MAS, BAN) in the framework of joint US-Russia JCCRER Project 1.1 (*[*https://www.energy.gov/ehss/russian-health-studies-program*](https://www.energy.gov/ehss/russian-health-studies-program)*).].*

*Please state what role the funders took in the study.  If the funders had no role, please state: "The funders had no role in study design, data collection and analysis, decision to publish, or preparation of the manuscript." If this statement is not correct you must amend it as needed. Please include this amended Role of Funder statement in your cover letter; we will change the online submission form on your behalf.*

The funders had no role in study design, data collection and analysis, decision to publish, or preparation of the manuscript. This statement has been added to a revised cover letter.

*3. Please include a caption for figure 5, 6, 7, 8.*

The captions have been bolded and separated from the main text by adding line spaces to make them more conspicuous.

Reviewers' comments:

Reviewer's Responses to Questions

**Comments to the Author**

*1. Is the manuscript technically sound, and do the data support the conclusions?

The manuscript must describe a technically sound piece of scientific research with data that supports the conclusions. Experiments must have been conducted rigorously, with appropriate controls, replication, and sample sizes. The conclusions must be drawn appropriately based on the data presented.*

*Reviewer #1: Yes*

*Reviewer #2: Partly*

*Reviewer #3: No*

For greater clarity, we have prepared a flowchart of the SPSD modeling approach and added it to the manuscript as new Fig 1. In addition, please see our responses to reviewer #2's comments.

*2. Has the statistical analysis been performed appropriately and rigorously?*

*Reviewer #1: Yes*

*Reviewer #2: No*

*Reviewer #3: I Don't Know*

We have included the necessary links and explanations in the text. See also our responses to reviewer #2's comments

*3. Have the authors made all data underlying the findings in their manuscript fully available?

The* [*PLOS Data policy*](http://track.editorialmanager.com/CL0/http:%2F%2Fwww.plosone.org%2Fstatic%2Fpolicies.action%23sharing/1/010f0194ee52247a-f9e7a6d2-9e8c-42eb-a745-43f9287dadbf-000000/ICD8zzdjI9cfKHMZOJ0oh0YI-w0qaCTv3PqUUJdd88A=197) *requires authors to make all data underlying the findings described in their manuscript fully available without restriction, with rare exception (please refer to the Data Availability Statement in the manuscript PDF file). The data should be provided as part of the manuscript or its supporting information, or deposited to a public repository. For example, in addition to summary statistics, the data points behind means, medians and variance measures should be available. If there are restrictions on publicly sharing data—e.g. participant privacy or use of data from a third party—those must be specified.*

*Reviewer #1: Yes*

*Reviewer #2: Yes*

*Reviewer #3: Yes*

Doesn't require any explanation

*4. Is the manuscript presented in an intelligible fashion and written in standard English?

PLOS ONE does not copyedit accepted manuscripts, so the language in submitted articles must be clear, correct, and unambiguous. Any typographical or grammatical errors should be corrected at revision, so please note any specific errors here.*

*Reviewer #1: Yes*

*Reviewer #2: Yes*

Reviewer #3: Yes

Doesn't require any explanation

5. Review Comments to the Author

*Reviewer #1: the text provides a good overview of the purpose of the study, but it could benefit from explicitly stating its primary objective earlier. For example, clearly define whether the focus is methodological innovation, parameter evaluation, or both.*

We have included the following explanation at the end of the Introduction section: “Therefore, the focus of the study is on methodological innovation and SPSD model parameter evaluation”.

*Reviewer #2: Dear Authors,
Thank you for your submission to PLOS ONE. Your manuscript presents a well-structured and methodologically sound study on Stochastic Parametric Skeletal Dosimetry (SPSD) for modeling radiation exposure to bone marrow. The approach is innovative and provides a robust framework for radiation risk assessment, particularly for bone-seeking radionuclides. However, I have noted some areas for improvement, particularly regarding statistical analysis, model validation, and clarity in the discussion of limitations.

(1) Include confidence intervals (CIs) or sensitivity analysis for key parameters. The Monte Carlo approach incorporates variability, but confidence intervals for trabecular thickness (Tb.Th), bone volume fraction (BV/TV), and cortical thickness (Ct.Th) are not explicitly reported. Providing 95% confidence intervals, standard deviations, or a bootstrapping approach would improve statistical robustness.*

The aim of our study was to evaluate the key morphometric parameters based on literature data. The characteristics of their variability (SD, CV, max-min) are presented in summary tables and Supporting information. The Monte Carlo method was not used for variability evaluation. It is possible the reviewer has addressed questions related to the next steps of our study, and their questions concern the generated phantoms. To clarify the steps of the SPSD modeling, we have included a flowchart with explanations (Fig 1). See answer to question 2.

*(2) Perform a sensitivity analysis to evaluate how variations in key parameters influence dose estimations. A one-at-a-time (OAT) sensitivity analysis or variance-based sensitivity analysis (e.g., Sobol indices) could provide valuable insights into parameter impact.
Provide empirical validation or benchmark comparisons. The study relies on literature-based data, but additional validation would strengthen credibility. If direct experimental validation is not possible, consider benchmarking results against ICRP reference phantoms or other computational dosimetry models (e.g., UF/NCI hybrid phantoms). A comparison table summarizing differences in dose estimations would be helpful*.

The reviewer makes valid points, however the objective of current study was not to estimate doses. A separate paper will be devoted to the evaluation of dosimetric coefficients. To clarify this situation, we have included a flowchart of SPSD modeling (Fig. 1) and the following text in the Introduction section:

“The development and implementation of the SPSD approach is a complex, multi-stage study that cannot be fully covered within a single paper. In this regard, we prepared a series of the papers that describe various aspects of SPSD modeling. General approaches were described by Degteva et al. [5,6]; mathematical models and software were described in [7,8]; the current paper presents the description of the anatomical-morphological basis for the SPSD model; and another publication (Sharagin et al., 2025) summarizes the data on phantoms generated for reference ages. The methods for evaluation of dose uncertainty and sensitivity were discussed in detail (Shishkina et al., 2023, Shishkina et al., 2024; Shishkina et al., 2024); a paper on dose coefficients (final modeling results) is currently in preparation. To clarify the overall picture, we have included a flowchart of SPSD modeling (Fig 1). The current paper describes the methodology and results of the initial steps (Fig 1 a-d), as well as the place of these results in the overall scheme of SPSD modeling.”

**Fig 1. Flowchart of SPSD-modeling**. Stages **a-c** are performed with use of published data: **a**- selection of skeleton bones with active marrow for reference ages; **b**- study of bone shapes, collection of morphometric parameters; (**c**) selection of bone segments with measured parameters and assignment of suitable geometric shapes to them (e.g., box, cylinder, cone). **d** – averaging and accepting parameters for bone segments and their geometric analogues (shapes): thickness of cortical layer (Ct.Th); trabecular thickness (Tb.Th), trabecular separation (Tb.Sp), fraction of bone volume in total volume of spongiosa (BV/TV), bone segment dimensions (a,b,c,d…) and characteristics of parameter variability. Stages **e-f** are performed using “Trabecila” software for generation of parametric phantoms in voxel representation. **e - Step 1** - spongiosa model is generated as an isotropic 3-D-grid of rod trabeculae with bone marrow in the space between them; Ct.Th and other parameters correspond to population-averaged values for bone segment. **e- Step 2** - stochastic 3D-deformation of trabeculae (in the range of intra- specimen variability CV_IS_) is carried out to make a model equivalent (BPS) of a real trabecular structure. **f -** generation of a set of 12 supplementary phantoms (SPS), whose parameters for Step 1 are randomly selected in the range SD (d). Stages **g-h** are performed using MCNP software for Monte Carlo simulation of electron/photon transport in the generated voxel phantoms. **g**- calculation of the population-average values of DF for each BPS; **h**- calculation of DF for each supplementary phantom (12 DFs for each bone-segment), estimation of SD for BPS-DF. **i** – estimation of skeletal average DF and its uncertainty for each reference age for radionuclide source in cortical and trabecular media separately.

Additional references:

Sharagin PA, Shishkina EA, Tolstykh EI, Smith MA, Napier BA. Stochastic parametric skeletal dosimetry model for humans: Pediatric and adult computational skeleton phantoms for internal bone marrow dosimetry. Submitted to PLoS ONE, PONE-D-25-02823 (01/22/2025)

Shishkina EA, Sharagin PA, Tolstykh EI. Effect of variability of human bone morphometric parameters on the uncertainty of internal bone marrow doses due to ^90^Sr. Radiatsionnaya Gygiena = Radiation Hygiene. 2024;17(1):60-68. (In Russ.) <https://doi.org/10.21514/1998-426X-2024-17-1-60-68>

Shishkina EA, Sharagin PA, Tolstykh EA. The uncertainty of estimation of doses to the bone marrow from 89,90Sr due to the variability of the chemical composition and bone density. Radiatsionnaya Gygiena = Radiation Hygiene. 2023;16(2):32-43. (In Russ.) <https://doi.org/10.21514/1998-426X-2023-16-2-32-43>

Shishkina EA, Sharagin PA, Tolstykh EI, Smith MA, Napier BA, Degteva MO. Uncertainty of stochastic parametric approach to bone marrow dosimetry of 89,90Sr. Heliyon. 2024 Feb 10;10(4):e26275. doi: 10.1016/j.heliyon.2024.e26275. PMID: 38420372; PMCID: PMC10900932.

*(3) Verify assumptions on parameter distributions. The manuscript assumes normal distributions for some trabecular bone parameters but does not test this assumption. A Kolmogorov-Smirnov (K-S) test or Shapiro-Wilk test should be performed to confirm normality. If non-normal, a log-normal or gamma distribution may be more appropriate.*

We used the published data, which in most cases contained mean parameter values and standard deviations (not raw data). For macro-parameters (dimensions), normal distribution was reported by the authors of the studies (for example, Krogman WM.1962). Regarding the trabecular bone parameters, the authors (Aaron et al. 1987; Kothari et al. 1999; Wakamatsu and Sissons 1969) pointed out some asymmetry in the distribution of Tb.Th. In particular, the data provided by Wakamatsu and Sissons (1969) best fit the 4-parameter Weibull distribution function (r2adj=0.994; W Statistic= 0.9137), but they also do not contradict the normal distribution (r2adj=0.942; W Statistic= 0.8938). The parameters Tb.Sp and Tb.Th are negatively correlated, and a normal distribution was also assumed for Tb.Sp.We added the following explanation in Section “Statistical methods used”:

“Bone parameters were assumed to be normally distributed. For dimensional parameters this is indicated by the authors of the studies (for example, Krogman WM.1962). For micro-parameters (trabecular thickness), authors indicated some asymmetry of distributions (Aaron 1987; Kothari 1999, Wakamatsu 1969), however, the normal distribution can also be applied for data description.”

References:

Krogman WM. The Human Skeleton in Forensic Medicine: C.C. Thomas; 1962.

Aaron JE, Makins NB, Sagreiya K. The microanatomy of trabecular bone loss in normal aging men and women. Clin Orthop Relat Res. 1987 Feb;(215):260-71. PMID: 3802645.

Kothari M, Keaveny TM, Lin JC, Newitt DC, Majumdar S. Measurement of intraspecimen variations in vertebral cancellous bone architecture. Bone. 1999 Aug;25(2):245-50. doi: 10.1016/s8756-3282(99)00161-1. PMID: 10456392.

Wakamatsu E, Sissons HA. The cancellous bone of the iliac crest. Calcif Tissue Res 4:147–161; 1969, doi:10.1007/bf02279116

*(4) Clarify segmentation methodology. The segmentation of hematopoietic sites into simple geometric shapes is described, but the rationale behind specific segmentations is not clear. A brief explanation of why certain segmentations were chosen, particularly for heterogeneous bone structures, would improve transparency.*

Segmentation methodology is described in detail in Supplementary information files for each specific bone. We also included the explanation in flowchart (Fig 1 с)

(*5) Improve readability in the methodology section. Some descriptions, especially in parameter selection and stochastic modeling, are complex and may be difficult for non-specialists to follow. Simplifying descriptions and adding a schematic diagram or flowchart of the SPSD modeling workflow would enhance clarity.*

As recommended by the reviewer, we have included a flowchart. See answer to question 2.

*(6) Address minor grammatical issues. While the manuscript is generally well-written, refining sentence structures and improving conciseness would improve readability.*

We made grammatical corrections

(7) These revisions will improve the statistical rigor, validation strength, and clarity of the manuscript. Addressing these concerns will enhance the study’s impact and ensure its findings are transparent and reproducible. Looking forward to the revised version.

This is a summary of the comments, see specific answers above

Reviewer #3: Thank you for massive data you gathered and the manuscript, but I didn't understand your introduction, your method and finally your discussion and I didn't find coordination between different parts of your manuscript. your abstract can not convey your work and is too long.
generally it is not obvious that 3. I think it needs total and basic rewriting and revision.

To clarify the manuscript, we have included a flowchart of the studies (Fig 1) and included several explanations in the Introduction section and “Statistical methods used”.
